# Impaired flux of bile acids from the liver to the gut reveals microbiome-immune interactions associated with liver damage

**DOI:** 10.1038/s41522-023-00398-0

**Published:** 2023-06-07

**Authors:** Howell Leung, Ling Xiong, Yueqiong Ni, Anne Busch, Michael Bauer, Adrian T. Press, Gianni Panagiotou

**Affiliations:** 1 Microbiome Dynamics, Leibniz Institute for Natural Product Research and Infection Biology - Hans Knöll Institute, Jena, Germany; 2 Jena University Hospital, Department of Anesthesiology and Intensive Care Medicine, Jena, Germany; 3 Friedrich Schiller University, Theoretical Microbial Ecology, Institute of Microbiology, Faculty of Biological Sciences, Jena, Germany; 4 Friedrich Schiller University, Medical Faculty, Jena, Germany; 5 Friedrich Schiller University Jena, Institute of Microbiology, Faculty of Biological Sciences, Jena, Germany

**Keywords:** Metagenomics, Microbiome

## Abstract

Currently, there is evidence that alteration in the gut ecosystem contributes to the development of liver diseases, however, the complex mechanisms involved are still unclear. We induced cholestasis in mice by bile duct ligation (BDL), mirroring the phenotype of a bile duct obstruction, to understand how gut microbiota alterations caused by an impaired flow of bile acid to the gut contribute to the pathogenesis and progression of liver disease. We performed longitudinal stool, heart, and liver sampling using mice receiving BDL and controls receiving sham operation (ShamOP). Shotgun metagenomics profiling using fecal samples taken before and on day 1, day 3, and day 7 after surgery was performed, and the cytokines and clinical chemistry profiles from heart blood, as well as the liver bile acids profile, were measured. The BDL surgery reshaped the microbiome of mice, resulting in highly distinct characteristics compared to the ShamOP. Our analysis of the microbiome pathways and ECs revealed that BDL reduces the production of hepatoprotective compounds in the gut, such as biotin, spermidine, arginine, and ornithine, which were negatively associated with inflammatory cytokines (IL-6, IL-23, MCP-1). The reduction of the functional potential of the gut microbiota in producing those hepatoprotective compounds is associated with the decrease of beneficial bacteria species from *Anaerotruncus*, *Blautia*, *Eubacterium*, and *Lachnoclostridium* genera, as well as the increase of disease-associated bacteria e.g., *Escherichia coli* and *Entercoccus faecalis*. Our findings advances our knowledge of the gut microbiome-bile acids-liver triangle, which may serve as a potential therapeutic strategy for liver diseases.

## Introduction

Globally, liver disease (LD) accounts for over two million fatalities annually, or 3.5% of all fatalities, and it has become more prevalent in aging populations^1,2^. The complicated etiology of LD encompasses several interconnected risk factors, including obesity, advanced age, and excessive alcohol consumption^3^. Non-alcoholic fatty liver disease (NAFLD) is the most common chronic liver disease that affects up to 25% of the population worldwide^4^. It is a complex disease linked to metabolic dysfunction, altered gut microbiome, and immune dysregulation^5–7^. Different therapeutic approaches for NAFLD targeting the host’s metabolism, the gut microbiota, and the immune system have been explored^8^. However, due to the complex interplay of these factors in the diseases, an integrated approach is required to investigate those elements.

Increasing evidence suggests that gut microbiota affect the development of NAFLD in multiple ways. Gut barrier disruption, bacterial translocation, and inflammatory response in the liver are some potential mechanisms that have been suggested in how the gut microbiome may influence NAFLD and non-alcoholic steatohepatitis (NASH)^9^. Furthermore, NAFLD and NASH patients exhibited significantly higher serum primary and secondary bile acids (BAs) than healthy individuals^10^. BAs are important metabolites in liver diseases^11^. Regulating secondary BAs metabolism is one of the known functions carried out by the gut microbiota. The elevation of secondary BAs production was associated with the increase of taurine- and glycine-metabolizing bacteria^12,13^. Therefore, a rise in secondary BAs may exacerbate NAFLD.

Nevertheless, it was reported that the microbiota regulates farnesoid X receptor (FXR) via secondary BAs^14^. FXR has an anti-inflammatory role and protects against cholestasis, NAFLD, and liver inflammation associated with NASH. It could be activated by the secondary BAs lithocholic acid and deoxycholic acid^15–17^, suggesting the secondary BAs have also a protective role against NAFLD progression.

Cytokines are involved in the pathogenesis of liver diseases in both disease-promoting and beneficial ways. Hence, their role remains controversial. For example, tumor necrosis factor-α (TNF-α) has a dichotomous role in the liver. In addition to acting as a mediator of cell death, it induces hepatocyte proliferation and liver regeneration^18^, whereas IL-6 also acts as both liver regeneration promoter and inflammation inducer^19^. In addition, inflammation could negatively impact the development of NAFLD depending on the disease stage, where inflammation contributes to liver injury in the early stage, and becomes a critical driver of host defense against pathogen infection and liver regeneration in the late stage^20^. Finally, the microbiota could regulate cytokine production in humans, mainly through the release of common intermediate mediators such as tryptophan, palmitoleic acid, and short-chain fatty acids, instead of exerting direct communication between microbes and immune cells^21,22^.

Cholestasis is a condition where bile flow from the liver is decreased or blocked, and depending on the mechanism, it could be classified into intrahepatic or extrahepatic cholestasis. Interestingly, some primary pathophysiological mechanisms are shared between cholestatic liver disease and NAFLD^23^. Bile duct ligation (BDL) is a standard method for inducing fibrosis and cirrhosis in mice by introducing obstructive cholestatic injury^24^. The morphological changes caused by BDL are similar to those observed in human biliary cirrhosis, allowing researchers to study the underlying pathophysiological mechanism and develop possible treatments^25^. Though BDL is a cholestatic liver disease model, it has also been successfully used as a tool in preclinical NAFLD and NASH research^26–28^. Recent studies have explored the microbiome taxonomic differences between BDL and control mice using 16S rRNA sequencing, and have proposed specific bacterial genera associated with the host hepatic gene expression response that may protect the liver during acute cholestasis^29^. Yet, the functional mechanisms of the gut–liver interplay in this mouse model system have remained largely unexplored.

To broaden our understanding of the gut–liver immune axis in liver diseases, we performed feces and blood longitudinal sampling in mice with BDL and sham operation (ShamOP). Our comprehensive and integrative analysis of the shotgun metagenomics profile with the host cytokines, liver bile acids, and biochemical markers provided further insights into possible mechanisms of the gut microbiome in modulating the severity of liver injury.

## Results

### Changes in biochemical markers, cytokines, and bile acids in BDL mice confirm the liver injury

**Fig. 1:**
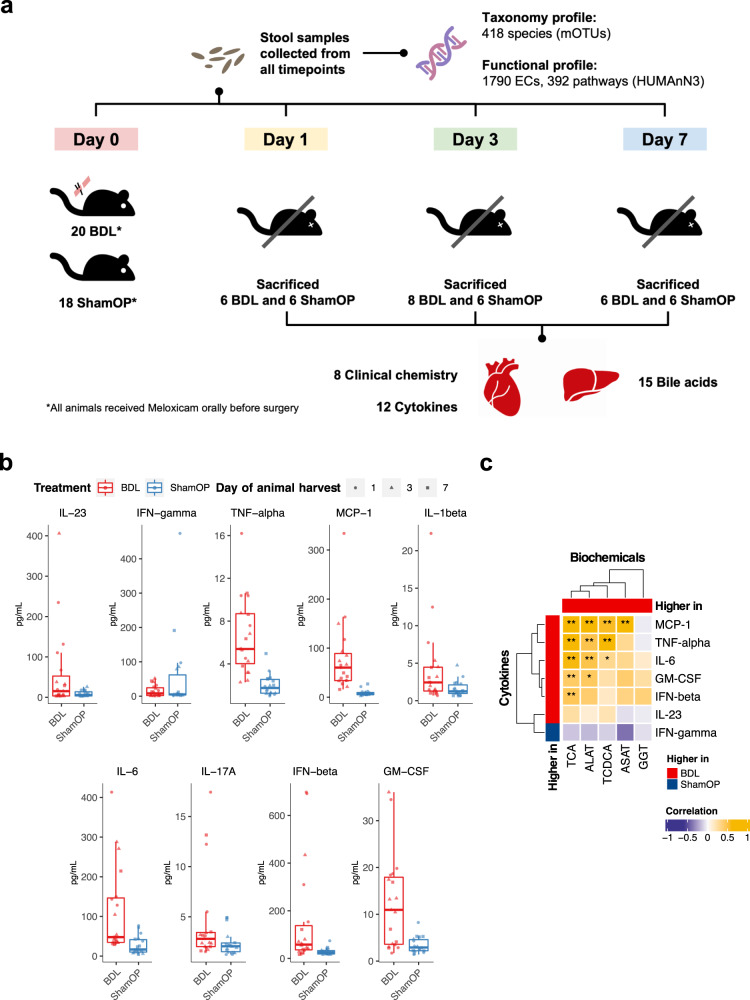
Study design and comparison of cytokines and biochemical profiles. **a** A graphical illustration of the study design, data collecting, and data generating procedures. **b** Box plots of significantly different cytokines between BDL and ShamOP mice (*P*-value < 0.05). The centre line indicates the median of the data, the bounds of the box represent the interquartile range, and the whiskers indicate the range of the data, excluding outliers. **c** Correlations between significantly different cytokines and significantly different biochemical markers, using data from all time points (day 1, day 3, and day 7). The significance of correlations is indicated by asterisk symbols, where a single asterisk (*) indicates *P*-value < 0.1 and a double asterisk (**) indicates *P*-value < 0.05.

A total of 38 mice were randomly assigned to receive BDL (*n* = 20) and ShamOP (*n* = 18) (Fig. 1a). The mice were sacrificed in batches on day 1, day 3, and day 7 after the surgery, and their heart blood, and liver samples were collected. We profiled 8 biochemical markers and 12 cytokines from the heart blood samples and 15 bile acids from the liver samples (Supplementary Table 1). Stool samples were obtained at all time points, from baseline (prior to surgery) to day 1, day 3, and day 7 before the mice were sacrificed. To confirm the liver damage induced by BDL, we examined the hematoxylin and eosin (H&E) images and compared the biochemical, inflammatory, and bile acid profiles between the BDL and ShamOP mice. Our histopathological examination of liver tissue through H&E staining confirmed the success of the BDL surgery in the BDL group (Supplementary Fig. 1). Also, the overall liver bile acid concentration was significantly increased in BDL mice, suggesting the bile flow was blocked as expected (Supplementary Fig. 2, generalized linear model, *P*-value < 0.05). We observed that the serum markers related to liver damage, including aspartate aminotransferase (ASAT), alanine aminotransferase (ALAT), gamma-glutamyl transferase (GGT), and triglyceride (TG), were significantly higher in BDL compared to ShamOP mice (Supplementary Fig. 2, generalized linear model, *P*-value < 0.05). The levels of ASAT and ALAT showed the largest difference between the two study groups. Our analysis also showed that the inflammatory cytokines IL-23, TNF-α, MCP-1, IL-1β, IL-6, IL-17A, GM-CSF, and IFN-β were significantly increased in BDL mice. In contrast, IFN-γ was significantly decreased (Fig. 1b & Supplementary Fig. 2, generalized linear model, *P*-value < 0.05). TNF-α, MCP-1, and IL-6 were the top differential cytokines between the BDL and ShamOP mice and were significantly positively correlated with ALAT and ASAT (Fig. 1c, Spearman correlation, *P*-value < 0.05).

### Blocking the flow of bile acids induced significant alterations in the gut microbiome composition

**Fig. 2:**
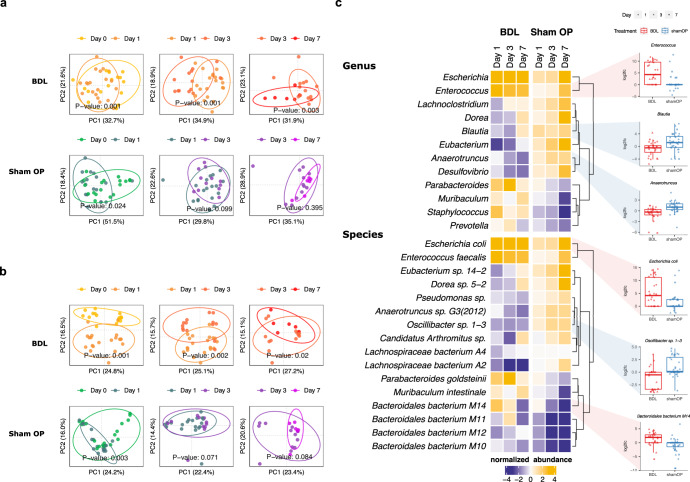
Comparison of the gut microbiome taxonomy profiles of BDL and ShamOP mice. Principal coordinate analysis (PCoA) of Bray–Curtis dissimilarity between gut microbiome abundance profiles at day 0, day 1, day 3, and day 7, at **a** genus and **b** species level. **c** Heatmap of significant differentially abundant genera and species (linear model, FDR < 0.1). Colors of cells indicate the direction and magnitude of the normalized abundance. Box plots on the side show the abundance of some selected taxa. The centre line indicates the median of the data, the bounds of the box represent the interquartile range, and the whiskers indicate the range of the data, excluding outliers. Colors and shapes of dots within box plots indicate the types of treatment and days of sample collection.

The gut microbiome structure of the BDL mice and ShamOP mice was assessed via shotgun metagenomics sequencing of all fecal samples. In total, we processed 110 stool samples and sequenced 770.4 gigabase pairs with an average of 46,689,954 high-quality reads per sample. Using mOTUs for taxonomic profiling^30^, we identified 60 genera and 418 species. Chao1, Shannon, and Simpson alpha diversity indices showed no significant differences between BDL and ShamOP mice when using the samples from all time points for comparison (Supplementary Table 2, linear mixed model, *P*-value > 0.05). Bray–Curtis distances were calculated to compare the beta diversity between and within groups. The beta diversity at the genus and species level between BDL and ShamOP at baseline had no significant difference (PERMANOVA, *P*-value > 0.05). However, it was significantly different when comparing the two mice groups on day 1, day 3, and day 7 (except for genus level, which did not reach statistical significance on day 3) (Supplementary Table 2, PERMANOVA, *P*-value < 0.05). Notably, significant differences were detected at both genus and species levels when comparing BDL-day 0 vs. BDL-day 1 and ShamOP-day 0 vs. ShamOP-day 1, suggesting abdominal incision would promote changes in the microbiota, regardless of the manipulation of the biliary system. Both genus and species composition continued to change significantly on day 3 and day 7 in BDL mice (PERMANOVA, *P* < 0.05) but not in the ShamOP mice (PERMANOVA, *P*-value > 0.05) (Fig. 2a, b).

By analyzing the taxonomic profile of the microbiome on each sampling day (normalized to baseline; detailed in Methods), we found 19 genera (12 having the exact genus annotation) significantly different in abundance between BDL and ShamOP mice (Fig. 2c and Supplementary Table 3, linear model, FDR < 0.05). From the 19 significant genera, nine were enriched in the BDL group, while ten were enriched in the ShamOP mice. *Escherichia* and *Eubacterium* were the top enriched genera in BDL and ShamOP mice (mean normalized abundance of the three post-surgery days = 5.23 and 2.22, respectively) (Supplementary Table 3). Besides *Escherichia*, when we looked specifically at genera with exact genus annotation, five genera had significantly higher relative abundance in BDL compared to ShamOP mice (linear model, FDR < 0.05), including *Enterococcus* and *Prevotella*, previously reported in mice and human studies to promote liver disease or inflammation^31,32^. In contrast, six genera significantly enriched in ShamOP mice included known butyrate-producing bacteria, such as *Anaerotruncus*, *Blautia*, *Eubacterium*, and *Lachnoclostridium* (linear model, FDR < 0.05)^33,34^. Some genera showed clear trends in the changes in relative abundance. For example, the relative abundance of *Anaerotruncus* continued decreasing in BDL mice until day 7 after surgery (Fig. 2c and Supplementary Table 3).

At the species level, we found in total 128 species (16 having exact species annotation) significantly different in abundance between the BDL and ShamOP mice (Fig. 2c and Supplementary Table 4, linear model, FDR < 0.05). Of those species, 22 and 106 were found to be significantly enriched in the BDL and ShamOP mice, respectively. *Escherichia coli* and *Enterococcus faecalis* were the top enriched ones in BDL mice (mean normalized abundance of the three post-surgery days = 5.24 and 4.26, respectively). Species belonging to *Clostridiales* and *Lachnospiraceae* were the top enriched ones in ShamOP mice (mean normalized abundance of the three post-surgery days = 3.35 and 3.33, respectively) (Supplementary Table 4). Several significantly different species have been reported to be negatively or positively associated with the development of liver diseases. An increased abundance of *E. coli* was observed in NAFLD patients with advanced fibrosis and might contribute to pro-inflammatory activity^35^. *Enterococcus faecalis* has been reported to exacerbate alcoholic hepatitis in mice^36^. A mice study revealed a higher abundance of *Bacteroidales* bacteria in the alcoholic fatty liver disease (AFLD) group compared to the control group^37^. Also in our study, *Bacteroidales* species were higher in BDL compared to ShamOP mice (Fig. 2c and Supplementary Table 4).

On the other hand, butyrate-producing bacteria such as *Lachnospiraceae* bacterium *A2*, *Lachnospiraceae* bacterium *A4*, and *Anaerotruncus sp G3 (2012)* were significantly higher in ShamOP mice (Fig. 2c, linear model, FDR < 0.05)^11^. *Lachnospiraceae bacterium A2* exhibited a significant drop in relative abundance in BDL mice on all three post-surgery days, while its relative abundance did not change in ShamOP mice. Bacteria belonging to the *Oscillibacter* genus have been reported to be more abundant in healthy individuals than in individuals with NAFLD^38^. On the contrary, *Oscillibacter* was also reported to be associated with liver steatosis in non-diabetic obese women^39^. A member of this genus, *Oscillibacter sp 1-3*, was found in higher abundance in ShamOP compared to BDL mice (linear model, FDR < 0.05). However, its relative abundance continuously decreased in BDL mice. Interestingly, another beneficial bacteria species, *Parabacteroides goldsteinii*, has shown anti-inflammatory properties in high-fat-diet-induced obesity^40^, and was found in higher relative abundance in BDL compared to ShamOP mice (linear model, FDR < 0.05).

In summary, BDL caused significant changes in the gut community structure, as shown by beta diversity comparisons with the ShamOP mice. In addition, many species were affected by the block of bile acid flow in the gut, as evidenced by a reduction of known butyrate producers and an increase of bacteria mainly associated with liver disease.

### BDL decreases intestinal production of hepatoprotective compounds

The shotgun metagenomics analysis of the stool samples applied here allowed us to subsequently annotate the pathways and enzyme profiles of the microbial communities in the two mice groups. We identified 392 pathways and 1790 ECs using HUMAnN3^41^. The analysis of the pathway profiles revealed the effect of BDL in changing not only the microbiome composition but also the functional profile of the community. In contrast to the taxonomy, a significant difference was observed at the functional level when we compared the community alpha diversities between BDL mice and ShamOP mice, measured as Shannon, Simpson, and Chao1 indices (linear mixed model, *P*-value < 0.05, Supplementary Table 5). In addition, the beta diversity of the pathway profiles between BDL and ShamOP groups pooling samples from all time points was significantly different (PERMANOVA, *P*-value < 0.05). The pathway composition changed significantly from baseline to day 1 after surgery in both BDL and ShamOP mice (PERMANOVA, *P*-value < 0.05). Likewise, in the microbiome taxonomic profile our results suggest that abdominal incision would also cause alterations in the microbiota’s functional potential. These significant changes continued until day 3 in BDL mice (PERMANOVA, *P*-value < 0.05) but not in the ShamOP mice (PERMANOVA, *P*-value > 0.05). Even though in the BDL mice, the taxonomy composition continued to change significantly from day 3 to day 7 (Fig. 2a, b), the community functional profile did not follow (Supplementary Table 5, PERMANOVA, *P*-value > 0.05).

**Fig. 3:**
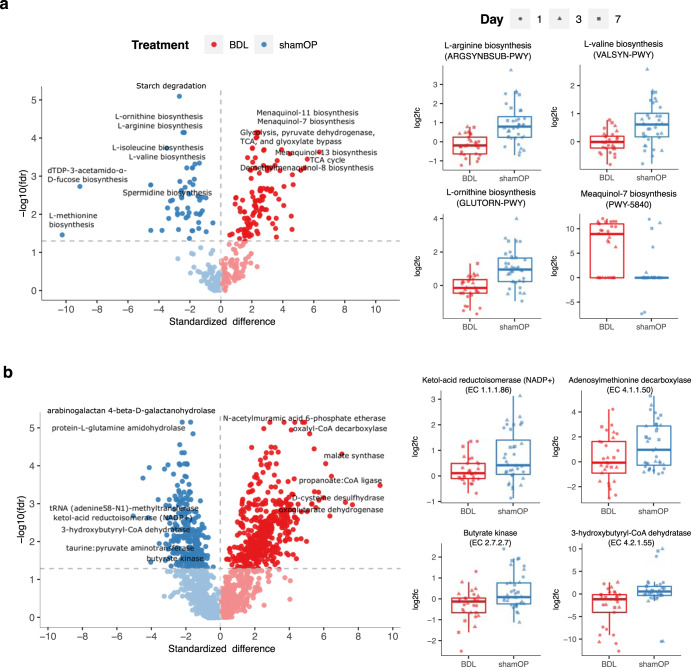
Comparison of the gut microbiome functional profiles of BDL and ShamOP mice. Volcano plots showing the differential **a** pathways and **b** EC fold-change profiles in the two groups, BDL and ShamOP. The horizontal dashed lines represent the −log10 adjusted *P*-value at FDR at 0.05 (linear model). Several pathways and ECs we discuss in the main text are shown as box plots, the centre line within indicates the median of the data, the bounds of the box represent the interquartile range, and the whiskers indicate the range of the data, excluding outliers.

We normalized the abundance of pathways and enzymes to their baseline and compared the functional profiles between the BDL and ShamOP mice. We observed that 195 functional pathways and 762 ECs were significantly different in relative abundance between the BDL and ShamOP mice after surgery (Fig. 3, linear model, FDR < 0.05); 139 and 56 pathways and 483 and 279 genes were enriched in BDL and ShamOP mice, respectively (Supplementary Tables 6 and 7). The biotin biosynthesis pathway (PWY-5005) was significantly lower in relative abundance in the BDL mice (linear model, FDR < 0.05), and a gene encoding the enzyme 6-carboxyhexanoate–CoA ligase (EC 6.2.1.14), which is involved in the biotin biosynthesis pathway, was significantly lower in BDL mice (linear model, FDR < 0.05). Biotin reduces hepatotoxicity and oxidative stress in liver of diabetic mice, and a biotin deficiency enhances the secretion of pro-inflammatory cytokines in human study^42^. The biosynthetic pathways of two branched-chain amino acids (BCAAs), isoleucine and valine (ILEUSYN-PWY and VALSYN-PWY), were significantly higher in ShamOP mice compared to BDL mice (Fig. 3a and Supplementary Table 6, linear model, FDR < 0.05). NAFLD patients were found to have increased levels of isoleucine and valine as the disease progressed^43^. However, it was also reported that isoleucine and valine could reduce hepatic fat accumulation at the early stage of NAFLD and prevent acute liver injury in mice, and restore immune function in advanced cirrhosis patients^44,45^. Several ECs that contribute to isoleucine and valine biosynthesis had significantly higher abundance in ShamOP mice (Fig. 3b and Supplementary Table 7, linear model, FDR < 0.05), including ketol-acid reductoisomerase (NADP+) (EC 1.1.1.86), acetolactate synthase (EC 2.2.1.6), and dihydroxy-acid dehydratase (EC 4.2.1.9). The biosynthetic pathways of arginine (ARGSYN-PWY and ARGSYNBSUB-PWY) were also significantly higher in ShamOP mice (Fig. 3a, linear model, FDR < 0.05). A mice study suggested an arginine and bile acid conjugate could be a possible treatment for NAFLD^46^. The microbial functional potential in producing ornithine (GLUTORN-PWY), a compound that has a hepatoprotective effect in NAFLD^47^, was also significantly lower in BDL mice (Fig. 3a, linear model, FDR < 0.05). Moreover, spermidine alleviates acute liver injury^48^, and the spermidine biosynthesis pathway (PWY-6834) was significantly lower in BDL mice compared to ShamOP mice (Fig. 3a, linear model, FDR < 0.05). This pathway had a clear decreasing trend in BDL mice, while its relative abundance only slightly fluctuated during the post-surgery period in ShamOP mice. Within the spermidine biosynthesis pathway, two ECs N1-aminpropylagmatine ureohydrolase (EC 3.5.3.24) and adenosylmethionine decarboxylase (EC 4.1.1.50) were significantly higher in ShamOP mice compared to BDL mice (Fig. 3b and Supplementary Table 7 linear model, FDR < 0.05). A gene encoding taurine–pyruvate aminotransferase (EC 2.6.1.77), which produces alanine that promotes restoration of a damaged liver^49^, was also significantly higher in ShamOP mice (Fig. 3b, linear model, FDR < 0.05). Furthermore, the relative abundance of the peptidoglycan biosynthesis pathway (PWY-5265) in BDL mice was significantly higher than in ShamOP mice (Supplementary Table 6, linear model, FDR < 0.05), while peptidoglycan was a pro-inflammatory factor found to induce the progression of steatohepatitis^50^. EC 2.3.2.17, a gene encoding an enzyme involved in peptidoglycan biosynthesis, was significantly higher in BDL mice (Supplementary Table 7, linear model, FDR < 0.05).

On the contrary, the biosynthesis pathways of menaquinone (PWY-5838, PWY-5840, PWY-5861, PWY-5897, and PWY-5899) had a significantly higher relative abundance in BDL mice compared to ShamOP mice (Fig. 3a and Supplementary Table 6 linear model, FDR < 0.05). Furthermore, it has been reported that NAFLD patients with fibrosis have a higher potential for bacterial menaquinone production than NAFLD patients without fibrosis^51^. Last but not least, we investigated whether limiting bile acid flow would impact the gut microbiome’s ability to produce butyrate. Even though at the taxonomy level, we found significant differences in the abundance of butyrate producers, none of the butyrate-producing pathways in our dataset (PWY-5022, PWY-5676, P162-PWY, and CENTFERM-PWY) were significantly different in relative abundance between the two study groups (Supplementary Table 6, linear model, *P*-value = 0.16-1). Yet, the relative abundance of two ECs involved in these butyrate-producing pathways, butyrate kinase (EC 2.7.2.7) and 3-hydroxybutyryl-CoA dehydratase (EC 4.2.1.55), were significantly lower in BDL mice compared to ShamOP mice (Fig. 3b, linear model, FDR < 0.05).

Overall, the functional potential of the microbiome in BDL mice was greatly affected, including a reduction in the production of anti-inflammatory and hepatoprotective compounds (biotin, isoleucine, valine, ornithine, arginine, and spermidine) and increased production of menaquinone. Furthermore, specific ECs participating in butyrate biosynthesis were also found to be decreased in BDL mice.

### Gut bacterial compound production promotes pro-inflammatory cytokines production

We next sought to study how the reshaping of the gut microbiome induced by BDL is associated with the cytokine profile. We performed a three-way correlation analysis between the cytokines profile, functional profile, and taxonomy profile to determine which taxa and functions were associated with the difference in the levels of the measured cytokines. To determine which taxa/functions may contribute to liver injury upon limiting bile acid flow, we focused on the cytokines, genera, species, pathways, and ECs that were significantly different in abundance between the BDL and Sham OP groups after normalizing to baseline values (generalized linear model or linear model, FDR < 0.05).

**Fig. 4:**
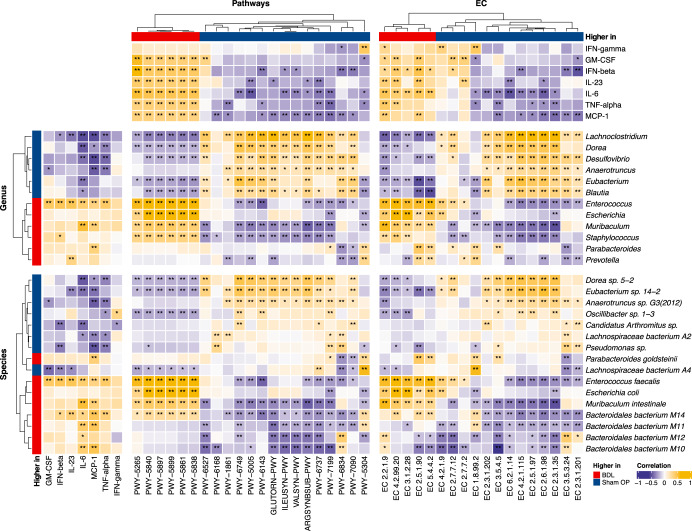
Heatmap showing correlations between important cytokines and significant microbial features. The genera and species shown significantly differ between BDL and ShamOP mice (linear model, FDR < 0.05). The pathways shown are significantly different in BDL mice compared with ShamOP mice (linear model, FDR < 0.05). The ECs shown are part of the significant pathways and significantly changed in BDL compared with ShamOP mice. Pathways that do not have at least one significant correlation (Spearman correlation, *P*-value < 0.05) with the significant taxa (linear model, FDR < 0.05) and cytokines (generalized linear model, FDR < 0.05) were removed. The significance of correlations is indicated by asterisk symbols, where a single asterisk (*) indicates *P*-value < 0.1 and a double asterisk (**) indicates *P*-value < 0.05.

As shown in Fig. 4, butyrate-producing genera *Anaerotruncus*, *Blautia*, *Eubacterium*, and *Lachnolostridium* that were found lower in BDL mice were significantly positively correlated (Spearman correlation, *P*-value < 0.05) with the biosynthesis pathway of biotin (PWY-5005), which was also significantly lower in BDL mice. Two species from these butyrate-producing genera, *Eubacterium sp. 14-2* and *Anaerotruncus sp. G3 (2012)* were also significantly positively correlated with this biosynthesis pathway. In contrast, two genera that were higher in BDL mice, *Enterococcus* and *Prevotella*, and two species that were also higher in BDL mice, *Enterococcus faecalis* and *Bacteroidales bacterium M14*, had significant negative correlations with the biotin production pathway (Fig. 4, Spearman correlation, *P*-value < 0.05). In addition, the functional potential of the microbiota synthesizing biotin was significantly negatively correlated with pro-inflammatory cytokines IL-6 and IL-23 (Fig. 4, Spearman correlation, *P*-value < 0.05). A high IL-6 level was found to increase the risk of NAFLD^52^, and IL-23 was elevated in NASH patients and was suggested to be indirectly linked with hepatic steatosis and pro-inflammatory response in NAFLD^53^.

*Anaerotruncus*, *Blautia*, *Eubacterium*, and *Lachnolostridium* also had positive correlations with the gut microbiota biosynthesis of two amino acids, ornithine and arginine (GLUTORN-PWY and ARGSYNBSUB-PWY) (Fig. 4, Spearman correlation, *P*-value < 0.05). These two biosynthesis pathways were lower in BDL mice and were also strongly positively linked with *Eubacterium sp. 14-2* and *Anaerotruncus sp. G3 (2012)*. Oppositely, significant negative correlations were shown between the ornithine production pathway and *Prevotella* and the biosynthesis pathway of arginine and *Enterococcus* (Fig. 4, Spearman correlation, *P*-value < 0.05). Bacteroidales bacteria that were found higher in BDL mice (*Bacteroidales bacterium M10*, *Bacteroidales bacterium M11*, *Bacteroidales bacterium M12*, and *Bacteroidales bacterium M14*) were correlated negatively with the two amino acids biosynthesis pathways (Fig. 4, Spearman correlation, *P*-value < 0.05). In addition, the ornithine and arginine biosynthesis pathways were negatively correlated with MCP-1, a cytokine that contributes to hepatic steatosis and fibrosis^54,55^.

Moreover, we found that the functional capability for microbiota synthesizing spermidine (PWY-6834) significantly positively correlated with *Anaerotruncus*, *Blautia*, *Eubacterium*, and *Lachnolostridium* (Fig. 4, Spearman correlation, *P*-value < 0.05). On the other hand, the spermidine biosynthesis pathway was lower in BDL mice. Two species that were lower in BDL mice, *Anaerotruncus sp. G3 (2012)* and *Lachnospiraceae bacterium A2*, were also positively correlated with the spermidine production pathway. On the other hand, *Enterococcus*, *Prevotella*, *Bacteroidales bacterium M11*, and *Bacteroidales bacterium M14* were negatively correlated with this pathway (Fig. 4, Spearman correlation, *P*-value < 0.05). In addition, the spermidine biosynthesis pathway negatively correlated with IFN-beta and MCP-1 (Fig. 4, Spearman correlation, *P*-value < 0.05).

We also observed lower levels of isoleucine and valine production pathways (ILEUSYN-PWY and VALSYN-PWY) in BDL mice. These pathways were positively correlated with *Lachnolostridium* and *Eubacterium sp. 14-2,* and negatively correlated with *Bacteroidales bacterium M10*, *Bacteroidales bacterium M11* and *Bacteroidales bacterium M12* (Fig. 4, Spearman correlation, *P*-value < 0.05). Moreover, pro-inflammatory cytokines IL-6 and MCP-1 showed significant negative correlations with the functional potential of microbiota synthesizing isoleucine and valine (Fig. 4, Spearman correlation, *P*-value < 0.05).

In addition, the menaquinone biosynthesis of the microbiota (including PWY-5840, PWY 5899, PWY-5897, PWY-5861, and PWY-5838) was found to be higher in BDL and had significant positive correlations with the two most enriched species in BDL, *E. coli* and *E. faecalis*. These biosynthesis pathways were negatively correlated with *Eubacterium sp. 14-2* and *Oscillibacter sp. 1-3* (Fig. 4, Spearman correlation, *P*-value < 0.05). GM-CSF, IFN-β, IL-23, IL-6, MCP-1, and TNF-α have a strong positive link with the functional ability of the microbiota to produce menaquinone (Fig. 4, Spearman correlation, *P*-value < 0.05).

Our analysis investigated the link between the host inflammatory state and the gut microbiome, and it highlights several possible mechanisms of gut microbiota contributions in liver injury when bile acid flow stoppage into the gut. The taxonomic changes induced by BDL were associated with a reduction in the biosynthesis of beneficial compounds (biotin, ornithine, arginine, spermidine, isoleucine, and valine) and increased menaquinone production in the gut, which led to the promotion of pro-inflammatory cytokines (such as IL-6 and MCP-1) and exacerbated liver damage, as shown by their correlation with liver damage markers (such as ALAT and ASAT).

## Discussion

The gut–liver axis comprises the intestine, which supplies roughly 70–75% of the blood flow to the liver^56^. The liver is a crucial component of the immune system and acts as the focal point of communication between the gut microenvironment and the host metabolism. As a result, the liver is exposed to numerous bacterial components, metabolites, and signals originating from the microbiome. According to recent research, bile acids function as pleiotropic signaling molecules that mediate the crosstalk between the gut and liver^57,58^. The intricate interaction between bile acids and the gut flora (which involves mutual dependency and inhibition) is crucial for maintaining mammalian homeostasis. Bile acids that have not been conjugated can balance the pH difference across the cell membrane. Cell membrane damage can result directly from the proton pump’s subsequent absence of bioenergy. Bile acids eventually prevent some bacteria from growing and participate in the gut microbiome’s development^59^. In parallel, bile acids are essential molecules for bi-directional regulation between the liver and the gut, and two signaling pathways activate them. A signaling molecule binds to the G-protein-coupled bile acid receptor 1 (GPBAR1 or TGR5), which regulates hepatic steatosis and the inflammatory response, and activates the expression of the FXR; this then controls the balance of energy metabolism, controls hepatic steatosis and the inflammatory response, and influences the composition of the intestinal tract. As a result, the pathophysiology of liver illnesses may be affected by the gut microbiome’s usage of bile acids as signaling molecules^12,60–62^. New treatments may be based on a better understanding of the precise areas of action of the gut microbiome and bile acids in various signaling pathways in liver disorders.

The need to look into the molecular mechanisms involved in the interplay of the gut microbiota, bile acids, and liver diseases was further highlighted by a recent mice study^29^, where they performed BDL on germ-free (GF) mice and altered-Schaedler-flora-colonized mice. Acute cholestasis induced more severe liver injury in GF compared to mice with limited gut microbial load, which, as the authors have shown, was associated with changes in the hepatic gene expression involved in tissue repair and several metabolic and immune functions with hepatoprotective effects. Nevertheless, the role of individual microbial species and the functional mechanisms for the microbial-induced differences that modulate liver injury remained unclear. To fill this gap and suggest targeted gut microbiota modifications that may be beneficial in attenuating liver injury, we performed here shotgun metagenomics sequencing in the BDL mice model and ShamOP mice with longitudinal sampling. In addition, we investigated how the changes in microbial species composition and biosynthetic potential in response to the stoppage of bile acids from the liver to the gut were associated with biochemical and inflammatory profiles.

The BDL surgery reshaped the microbiome of mice, resulting in highly distinct characteristics compared to the ShamOP control. Like the taxonomy, abdominal incision changed the microbiome functional ability of both BDL and ShamOP mice, while the alterations were only maintained in BDL mice. Our analysis of the pathways and ECs revealed the microbiota’s functional role in pathogenesis during acute liver damage. BDL reduces the production of hepatoprotective compounds in the gut, such as biotin, spermidine, arginine, ornithine, isoleucine, and valine^42,44–46,48,63^. BDL induces bacterial menaquinone production. It may increase damage to the liver by promoting vitamin K-dependent proteins, which play an essential role in chronic liver fibrosis^64^. Abundances of ECs for butyrate production were significantly dropped in BDL mice, suggesting a possible role of bile acid in keeping the level of butyrate in the gut stable again^65^. The reduction of the functional potential of the gut microbiota in producing several hepatoprotective compounds (biotin, ornithine, arginine, spermidine, isoleucine, and valine) is associated with the decrease of beneficial bacteria *Anaerotruncus*, *Blautia*, *Eubacterium*, and *Lachnoclostridium* and the increase of harmful bacteria in liver disease (*E. coli*, *E. faecalis*, and *Bacteroidales* species). The biosynthesis of these compounds was negatively associated with inflammatory cytokines (IL-6, IL-23, and MCP-1) linked with hepatic steatosis and fibrosis in NAFLD^52–55^. In contrast, the higher menaquinone production in the gut microbiome of BDL mice is linked to elevated *E. coli* and *E. faecalis* and reduced butyrate-producing bacteria. Menaquinone production was positively associated with inflammatory cytokines (GM-CSF, IFN-β, IL-23, IL-6, MCP-1, and TNF-α). These results highlight the association between cytokine profiles and the gut microbiota and reveal the potential mechanism by which the gut microbiome contributes to inflammation and liver injury.

According to Gergiev et al., most injury induced by BDL occurs within the first 5 to 7 days, where acute inflammatory processes are observed till day 3, and significant bile-duct proliferation is found after day 5^66^. Repair mechanism and bile-duct proliferation determine the molecular adaptation in the second week, and restore partial bile-flow which impacts the microbiome. Hence, we focused on the early days (days 1, 3, and 7) after surgery in our study to avoid the potential bias induced by bile-flow restoration at late time points. Moreover, our study also has limitations. The longitudinal sampling lasted for 7 days, while more severe liver injury (liver fibrosis) induced by BDL takes 20 days to fully develop^24^. We could only examine the role of the gut microbiome in liver injury development in the initial phase. The composition of individual bacterial species and their respective functions might continue to change and show a different pattern as the disease progresses. The observed alterations in this study may contribute to the initial effects of BDL and liver injury development, but may be not directly related to severe liver disease. These cholestasis-related and BA-dependent modifications in the gut microbiota and biochemistry during the first 7 days of the study may serve as precursors and promote the changes related to more severe liver conditions. Future studies may prolong the experiment to at least 20 days to provide a complete overview of the gut microbiota contribution during ongoing fibrogenesis. There are also some differences between bile acid pools of humans and mice which may limit the transferability of our findings to humans. The bile acid composition is different between mice and humans where hydrophilic 6-hydroxylated muricholic acids comprise half of the BA pool in mice. Moreover, cholestasis has different effect on BA production in mice and humans, where BDL increases BA synthesis in mice, while in humans, cholestasis reduces BA synthesis^67,68^. Future studies may use humanized mice models to address these limitations and improve our understanding of the link between bile acids, gut microbiomes, and human health.

Nevertheless, overall our results reveal the contribution of gut microbiota and its potential molecular mechanism during impaired bile acid flow from the liver to the intestine in hepatic inflammation, or even ongoing fibrogenesis, by targeting the relationship between the metabolism, the gut microbiome, and the immune system. Our findings also advance our knowledge of the role of bile acids as a cornerstone of the immune axis between the liver and the gut microbiome, and their optimal utilization as potential therapeutic targets.

## Methods

### Ethical statement

All experimental animal procedures in this study were evaluated through the Independent Ethical Advisory Committee and approved by the Local Government Authority of Thuringia, Thüringer Landesverwaltungsamt, per European and German laws and regulations under the license UKJ-19-010. For all experiments, male and female FVB/NRj mice (Janvier Laboratory, France) were housed under specific pathogen-free conditions and bred in the Jena University Hospital (Germany) animal facility. All animals were given LASQ diet (Rod 16, Auto) from Altromin company, which was composed of cereals, vegetable by-products, minerals, oils, fats, and yeast, and provided mice crude nutrients with 16.9% crude protein, 4.3% crude fat, 4.3% crude fiber, and 7.0% crude ash.

### Bile duct ligation

All animals were weighed, scored, and received 1 mg kg^−1^ body weight (BW)^−1^ Meloxicam orally (Meloxicam, CP-Pharma Handelsgesellschaft mbH, Burgdorf, Germany, 0.5 mg mL^−1^ oral suspension) 1 day and 1 h before surgery. The pre-surgical preparation, surgical procedures under anesthesia (Isoflurane, CP-Pharma Handelsgesellschaft mbH, Burgdorf, Germany, 1.5–3% in 100 mL min^−1^ O_2_ stream inhalation), and post-surgical treatment were described previously^24,69^. The bile duct was ligated with two pre-cut 6-0 braided silk sutures (Teleflex Medical GmbH, Fellbach, Germany, sterilized and soaked in 70% ethanol in advance) (BDL group). In contrast, the sham operation group (ShamOP) received the same surgical procedures except for the bile duct ligation. Afterward, the abdominal layers were closed and sewn up with 4-0 antibacterial sutures (Johnson & Johnson Medical GmbH Ethicon, Norderstedt, Germany, Vicryl Plus with pre-attached 17 mm 1/2c RB-1 plus needle). A 2 to 4 μg g^−1^ BW^−1^ Bupivacaine solution (PUREN Pharma GmbH, München, Germany, 2.5 mg mL^−1^) administered as an intra-incisional injection during suturing strengthened post-surgical analgesia. After surgery, the animals were weighed again and received 20 µL g^−1^ BW^−1^ Ringer acetate (Berlin Chemie AG, Berlin, Germany) subcutaneously. The animals recovered separately in single cages with free access to water and soft food under a warming lamp. Cholestasis manifested in all animals of the BDL group (but not in the ShamOP group) as urine discoloration and yellowing of paws or cornea circa 12 h after the surgery. Animals were followed up for 1, 3, or 7 days. Body weight was measured mornings and evenings. All animals received fluid resuscitation twice daily until the body weight was approximately at the pre-surgical level. Furthermore, oral analgesia and scoring were processed three times per day.

### Stool samples collection

Fresh stools were collected 1 h before surgery and 1, 3, and 7 days after. The stools were picked up with sterilized tweezers and transferred into a 2 mL micro-tube, where it was frozen immediately in liquid nitrogen for microbial DNA extraction.

### Plasma and liver sampling

At the end of the experiment (1, 3, or 7 days after surgery), animals were sacrificed with an anesthetic overdose of Xylazine (Rompun, Bayer Vital GmbH, Leverkusen, Germany, 80 mg kg^−1^ BW^−1^) and Ketamin (bela-pharm GmbH, Vechta, Germany, 500 mg kg^−1^ BW^−1^). After animals reached surgical tolerance, the abdomen was opened and heart blood was taken into a sterile 1 mL EDTA syringe connected to a 24 G needle via a final cardiac puncture. Afterward, the liver and spleen were removed, frozen in liquid nitrogen, and stored in micro-tubes at −80 °C for further analysis.

### Cytokine

The heart blood was centrifuged at 15,000 × *g* for 15 min at room temperature (RT) to obtain plasma. Then, 25 μL plasma from each animal was processed with LEGENDplex Mouse Inflammation Panel (13-plex) (BioLegend, Koblenz, Germany, #740150) to quantify the concentrations of 13 inflammatory cytokines. The LEGENDplex Mouse Inflammation Panel is a cytokine bead array quantifying specific cytokines that are identified based on their different forward scatter and fluorescence. The assay was carried out, measured on a BD Accuri C6 Plus flow cytometer (BD Bioscience, Heidelberg, Germany), that was validated by the manufacturer for this assay. As a result, IL-23, IL-1α, IFN-γ, TNF-α, MCP-1, IL-1β, IL-6, IL-27, IL-17A, IFN-β, GM-CSF, and anti-inflammatory cytokines IL-12p70 and IL-10 were identified by flow cytometry and quantified against a standard curve. The data analysis was performed according to the manufacturer’s protocol using the Lot- and assay-specific calibrated online analysis tool provided by BioLegend. The gating strategy for the different beads is manufacturer specific and provided with the cytokine bead array kit.

### Bile acid analysis

Concentrations of 15 bile acids were determined in isolated hepatocytes using an LC-MS/MS quantification method: taurocholic acid (TCA), glycocholic acid (GCA), glycochenodeoxycholic acid (GCDCA), taurochenodeoxycholic acid (TCDCA), taurolithocholic acids (TLCA), glycolithocholic acid (GLCA), taurodeoxycholic acid (TDCA), glycodeoxycholic acid (GDCA), cholic acid (CA), chenodeoxycholic acid (CDCA), ursodeoxycholic acid (UDCA), deoxycholic acid (DCA), tauroursodeoxycholic acid (TUDCA), glycoursodeoxycholic acid (GUDCA), and lithocholic acid (LCA). Pre-weighed samples were mixed with 3-fold (w/v) ethanol-phosphate buffer (15% 0.01 mol/L phosphate buffer solution pH 7.5, 85% ethanol), followed by a homogenizing step in a pebble mill (QiaShredder) and a centrifugation step (5 min, 16,000 × *g*). 270 µL of 85% aqueous methanol was added to 30 µL of the sample supernatant in a Thomson Single Step® Filter Vial (PES membrane 0.2 µM, Thomson Instrument Company, California). This solution was mixed for 20 s, centrifuged at 200 × *g* for 1 min, filtered, and placed in the autosampler. An Agilent 1200 high-performance liquid chromatography (HPLC) system (Agilent Technologies GmbH, Böblingen, Germany) with a CTC-PAL autosampler coupled to an API 4000 Triple Quadrupole mass spectrometer with electrospray ionization source (AB Sciex, Darmstadt, Germany) was used throughout. All chromatographic separations were performed with a reverse-phase Agilent Zorbax Eclipse XDB-C18 (3.5 µm, 100 × 3 mm) analytical column equipped with a guard column (C18, 4 × 3 mm; Phenomenex, Aschaffenburg, Germany). The mobile phase consisted of water (A) and methanol (B), containing 0.012% formic acid and 5 mM ammonium acetate, at a total flow rate of 300 µL min^−1^.

### Clinical chemistry

The Jena University Hospital routine clinical laboratory quantified eight biochemical markers: albumin, aspartate transaminase (ASAT), alanine transaminase (ALAT), glutamate dehydrogenase (GLDH, GDH), gamma-glutamyltransferase (Gamma-GT), triglyceride, lactate dehydrogenase (LDH), and alkaline phosphatase (ALP) when plasma remained.

### DNA extraction and sequencing for stool samples

All stool samples (about ~200 mg per sample) were processed by Novogene (UK). DNA was extracted using the following protocol: Stool samples were thoroughly mixed with 900 μL of CTAB lysis buffer. All samples were incubated at 65 °C for 60 min before being centrifuged at 12,000 × *g* for 5 min at 4 °C. Supernatants were transferred to fresh 2 mL microcentrifuge tubes and 900 μL of phenol:chloroform:isoamyl alcohol (25:24:1, pH = 6.7; Sigma-Aldrich) was added for each extraction. Samples were mixed thoroughly before incubating at room temperature for 10 min. Phase separation occurred by centrifugation at 12,000 × *g* for 15 min at 4 °C, and the upper aqueous phase was re-extracted with a further 900 μL of phenol:chloroform:isoamyl alcohol. Next, samples were centrifuged at 12,000 × *g* for 10 min at 4 °C, and the upper aqueous phases were transferred to fresh 2 mL microcentrifuge tubes. The final extraction was performed with 900 μL of chloroform:isoamyl alcohol (24:1), and layer separation occurred by centrifugation at 12,000 × *g* for 15 min at 4 °C. Precipitation of DNA was achieved by adding the upper phase from the last extraction step to 450 μL of isopropanol (Sigma-Aldrich) containing 50 μL of 7.5 M ammonium acetate (Fisher). Samples were incubated at –20 °C overnight, although shorter incubations (1 h) produced lower DNA yields. Samples were centrifuged at 7500 × *g* for 10 min at 4 °C, and supernatants were discarded. Finally, DNA pellets were washed in 1 mL of 70% (v/v) ethanol (Fisher). The final pellet was air-dried and re-suspended in 200 μL of 75 mM TE buffer (pH = 8.0; Sigma-Aldrich). Sequencing library was generated based on Illumina technologies and following manufactures’ recommendations. The DNA fragments were end-polished, A-tailed, and ligated with the full-length adapters of Illumina sequencing, followed by further PCR amplification with P5 and indexed P7 oligos. The PCR products as the final construction of the libraries were purified with the AMPure XP system. Then libraries were checked for size distribution by Agilent 2100 Bioanalyzer (Agilent Technologies, CA, USA), and quantified by real-time PCR (to meet the criteria of 3 nM). The qualified libraries are fed into Illumina sequencers (NovaSeq system).

### Metagenomics

#### Quality control and removal of host-derived reads

For quality control of raw reads, we removed all Illumina primer/adaptor/linker sequences in the first step. Subsequently, we used BWA version 0.7.4 to align all reads to the mouse genome (version: GRCm39), followed by the removal of reads with >90% coverage and 95% identity. We then performed pairwise comparisons of paired-end reads to eliminate potential PCR duplicates, using the criterion of 25 bp that consecutively matched from both ends of the forward and reverse reads. Finally, we trimmed any low-quality terminal regions in each read, defined as consecutive bases with a Phred quality score of <20^70^.

#### Taxonomy profiling and functional annotation

Taxonomic annotation of the high-quality reads was performed by mOTUs2 with default settings, generating taxonomic relative abundances^30^. For further analysis, bacterial community profiles were constructed at the family, genus, and species level for further analysis. Functional annotation after quality control was done by HUMAnN3^41^. The quantified pathway and gene family abundances in the units of RPKs (read per kilobase) were then normalized to copies per million (CPM) units. Gene families were then regrouped to EC domain for further analysis.

### Statistical analysis

#### Software

All analyses were performed using R Statistical Software (v4.1.1; R Core Team 2021).

#### Data pre-processing

Prevalence filtering was done to remove low-prevalence features, prevalence <10%, before differentially abundant analysis and diversity analysis.

#### Differentially abundant analysis and correlation analysis

Clinical data were analyzed by generalized linear model, adjusted by the harvest day and the body weight of the mice (clinical data ~ group + harvest day + body weight). Taxonomic and functional data were normalized to baseline (log2 fold change [log2FC] of follow-up with respect to baseline) before analysis and were analyzed by linear model, adjusted by the harvest day of feces from the mice (log2FC abundance ~ group + harvest day). Metagenomics data were centered log ratio (CLR) transformed using R package microbiome prior to correlation analysis^71^. Spearman’s correlations between data were analyzed with function rcorr from R package Hmisc^72^. *P*-value < 0.05 was considered statistically significant. The false-discovery rate (FDR) was calculated to adjust *P*-values for multiple hypotheses testing by applying the Benjamini–Hochberg procedure.

#### Diversity analysis for metagenomics data

The alpha diversity (Shannon, Simpson, and Chao1 indexes) was calculated using the R package vegan^73^. The linear mixed model was applied to compare the alpha diversity between groups. Bray–Curtis distances were calculated using the R package vegan for estimating community dissimilarities. Finally, permutational multivariate analysis of variance using function adonis from R package vegan was carried out to analyze the beta diversity between groups. *P*-value < 0.05 was considered statistically significant.

### Reporting summary

Further information on research design is available in the Nature Research Reporting Summary linked to this article.

## Supplementary information

Supplementary Information

Reporting Summary

## Data Availability

Raw metagenomic sequencing data for all samples of this study have been deposited in European Nucleotide Archive under accession ID PRJEB57214. Further information and requests for resources and reagents should be directed to G.P. (Gianni.Panagiotou@leibniz-hki.de).

## References

[1] Asrani SK, Devarbhavi H, Eaton J, Kamath PS (2019). Burden of liver diseases in the world. J. Hepatol..

[2] Kim IH, Kisseleva T, Brenner DA (2015). Aging and liver disease. Curr. Opin. Gastroenterol..

[3] Younossi ZM (2019). Non-alcoholic fatty liver disease—a global public health perspective. J. Hepatol..

[4] Younossi Z (2019). Global perspectives on nonalcoholic fatty liver disease and nonalcoholic steatohepatitis. Hepatology.

[5] Eslam M (2020). A new definition for metabolic dysfunction-associated fatty liver disease: an international expert consensus statement. J. Hepatol..

[6] Sharpton SR, Schnabl B, Knight R, Loomba R (2021). Current concepts, opportunities, and challenges of gut microbiome-based personalized medicine in nonalcoholic fatty liver disease. Cell Metab..

[7] Hotamisligil GS (2017). Inflammation, metaflammation and immunometabolic disorders. Nature.

[8] Tilg H, Adolph TE, Dudek M, Knolle P (2021). Non-alcoholic fatty liver disease: the interplay between metabolism, microbes and immunity. Nat. Metab..

[9] Kolodziejczyk, A. A., Zheng, D., Shibolet, O. & Elinav, E. The role of the microbiome in NAFLD and NASH. *EMBO Mol. Med*. **11**, 10.15252/emmm.201809302 (2019). 10.15252/emmm.201809302 PMC6365925 30591521

[10] Bechmann LP (2013). Free fatty acids repress small heterodimer partner (SHP) activation and adiponectin counteracts bile acid-induced liver injury in superobese patients with nonalcoholic steatohepatitis. Hepatology.

[11] Biddle A, Stewart L, Blanchard J, Leschine S (2013). Untangling the genetic basis of fibrolytic specialization by lachnospiraceae and ruminococcaceae in diverse gut communities. Diversity.

[12] Jiao N (2018). Suppressed hepatic bile acid signalling despite elevated production of primary and secondary bile acids in NAFLD. Gut.

[13] Ferslew BC (2015). Altered bile acid metabolome in patients with nonalcoholic steatohepatitis. Dig. Dis. Sci..

[14] Sayin SI (2013). Gut microbiota regulates bile acid metabolism by reducing the levels of tauro-beta-muricholic acid, a naturally occurring FXR antagonist. Cell Metab..

[15] Rizzo G, Renga B, Mencarelli A, Pellicciari R, Fiorucci S (2005). Role of FXR in regulating bile acid homeostasis and relevance for human diseases. Curr. Drug Targets Immune Endocr. Metab. Disord..

[16] Clifford BL (2021). FXR activation protects against NAFLD via bile-acid-dependent reductions in lipid absorption. Cell Metab..

[17] Armstrong LE, Guo GL (2017). Role of FXR in liver inflammation during nonalcoholic steatohepatitis. Curr. Pharm. Rep..

[18] Schwabe RF, Brenner DA (2006). Mechanisms of Liver Injury. I. TNF-alpha-induced liver injury: role of IKK, JNK, and ROS pathways. Am. J. Physiol. Gastrointest. Liver Physiol..

[19] Schmidt-Arras D, Rose-John S (2016). IL-6 pathway in the liver: from physiopathology to therapy. J. Hepatol..

[20] Gao B, Tsukamoto H (2016). Inflammation in alcoholic and nonalcoholic fatty liver disease: friend or foe. Gastroenterology.

[21] Vinolo MA, Rodrigues HG, Nachbar RT, Curi R (2011). Regulation of inflammation by short chain fatty acids. Nutrients.

[22] Schirmer M (2016). Linking the human gut microbiome to inflammatory cytokine production capacity. Cell.

[23] Trauner M, Fuchs CD (2022). Novel therapeutic targets for cholestatic and fatty liver disease. Gut.

[24] Tag, C. G. et al. Bile duct ligation in mice: induction of inflammatory liver injury and fibrosis by obstructive cholestasis. *J. Vis. Exp*. 10.3791/52438 (2015). 10.3791/52438 PMC4354634 25741630

[25] Kountouras J, Billing BH, Scheuer PJ (1984). Prolonged bile duct obstruction: a new experimental model for cirrhosis in the rat. Br. J. Exp. Pathol..

[26] Kluwe J (2010). Modulation of hepatic fibrosis by c-Jun-N-terminal kinase inhibition. Gastroenterology.

[27] Wang X (2017). A20 attenuates liver fibrosis in NAFLD and inhibits inflammation responses. Inflammation.

[28] Gabbi C (2012). Effects of bile duct ligation and cholic acid treatment on fatty liver in two rat models of non-alcoholic fatty liver disease. Dig. Liver Dis..

[29] Juanola O (2021). Intestinal microbiota drives cholestasis-induced specific hepatic gene expression patterns. Gut Microbes.

[30] Milanese A (2019). Microbial abundance, activity and population genomic profiling with mOTUs2. Nat. Commun..

[31] Llorente C (2017). Gastric acid suppression promotes alcoholic liver disease by inducing overgrowth of intestinal Enterococcus. Nat. Commun..

[32] Kwan SY (2022). Gut microbiome features associated with liver fibrosis in Hispanics, a population at high risk for fatty liver disease. Hepatology.

[33] Louis P, Flint HJ (2009). Diversity, metabolism and microbial ecology of butyrate-producing bacteria from the human large intestine. FEMS Microbiol. Lett..

[34] Vital M, Howe AC, Tiedje JM (2014). Revealing the bacterial butyrate synthesis pathways by analyzing (meta)genomic data. mBio.

[35] Loomba R (2017). Gut microbiome-based metagenomic signature for non-invasive detection of advanced fibrosis in human nonalcoholic fatty liver disease. Cell Metab..

[36] Duan Y (2019). Bacteriophage targeting of gut bacterium attenuates alcoholic liver disease. Nature.

[37] Yan AW (2011). Enteric dysbiosis associated with a mouse model of alcoholic liver disease. Hepatology.

[38] Jiang W (2015). Dysbiosis gut microbiota associated with inflammation and impaired mucosal immune function in intestine of humans with non-alcoholic fatty liver disease. Sci. Rep..

[39] Hoyles L (2018). Molecular phenomics and metagenomics of hepatic steatosis in non-diabetic obese women. Nat. Med..

[40] Wu TR (2019). Gut commensal Parabacteroides goldsteinii plays a predominant role in the anti-obesity effects of polysaccharides isolated from Hirsutella sinensis. Gut.

[41] Beghini, F. et al. Integrating taxonomic, functional, and strain-level profiling of diverse microbial communities with bioBakery 3. *Elife* **10**, 10.7554/eLife.65088 (2021). 10.7554/eLife.65088 PMC8096432 33944776

[42] Agrawal S, Agrawal A, Said HM (2016). Biotin deficiency enhances the inflammatory response of human dendritic cells. Am. J. Physiol. Cell Physiol..

[43] Lake AD (2015). Branched chain amino acid metabolism profiles in progressive human nonalcoholic fatty liver disease. Amino Acids.

[44] Iwao M (2020). Supplementation of branched-chain amino acids decreases fat accumulation in the liver through intestinal microbiota-mediated production of acetic acid. Sci. Rep..

[45] Tajiri K, Shimizu Y (2018). Branched-chain amino acids in liver diseases. Transl. Gastroenterol. Hepatol..

[46] Voloshin I, Hahn-Obercyger M, Anavi S, Tirosh O (2014). L-arginine conjugates of bile acids-a possible treatment for non-alcoholic fatty liver disease. Lipids Health Dis..

[47] Canbay A, Sowa JP (2019). L-Ornithine L-aspartate (LOLA) as a novel approach for therapy of non-alcoholic fatty liver disease. Drugs.

[48] Zhou S (2018). Spermine alleviates acute liver injury by inhibiting liver-resident macrophage pro-inflammatory response through ATG5-dependent autophagy. Front Immunol..

[49] Maezono K (1996). Alanine protects liver from injury caused by F-galactosamine and CCl4. Hepatology.

[50] Jin M (2020). Effects of peptidoglycan on the development of steatohepatitis. Biochim. Biophys. Acta Mol. Cell Biol. Lipids.

[51] Rodriguez-Diaz C (2022). Microbiota diversity in nonalcoholic fatty liver disease and in drug-induced liver injury. Pharm. Res..

[52] Duan Y (2022). Association of inflammatory cytokines with non-alcoholic fatty liver disease. Front Immunol..

[53] Tang Y (2011). Interleukin-17 exacerbates hepatic steatosis and inflammation in non-alcoholic fatty liver disease. Clin. Exp. Immunol..

[54] Kanda H (2006). MCP-1 contributes to macrophage infiltration into adipose tissue, insulin resistance, and hepatic steatosis in obesity. J. Clin. Invest..

[55] Seki E (2009). CCR2 promotes hepatic fibrosis in mice. Hepatology.

[56] Abdel-Misih SR, Bloomston M (2010). Liver anatomy. Surg. Clin. North Am..

[57] de Aguiar Vallim TQ, Tarling EJ, Edwards PA (2013). Pleiotropic roles of bile acids in metabolism. Cell Metab..

[58] Wahlstrom A, Sayin SI, Marschall HU, Backhed F (2016). Intestinal crosstalk between bile acids and microbiota and its impact on host metabolism. Cell Metab..

[59] Ciocan D (2018). Bile acid homeostasis and intestinal dysbiosis in alcoholic hepatitis. Aliment Pharm. Ther..

[60] Ma, C. et al. Gut microbiome-mediated bile acid metabolism regulates liver cancer via NKT cells. *Science* **360**, 10.1126/science.aan5931 (2018). 10.1126/science.aan5931 PMC6407885 29798856

[61] Hartmann P (2018). Modulation of the intestinal bile acid/farnesoid X receptor/fibroblast growth factor 15 axis improves alcoholic liver disease in mice. Hepatology.

[62] Wehr A (2013). Chemokine receptor CXCR6-dependent hepatic NK T Cell accumulation promotes inflammation and liver fibrosis. J. Immunol..

[63] Butterworth RF, Canbay A (2019). Hepatoprotection by L-ornithine L-aspartate in non-alcoholic fatty liver disease. Dig. Dis..

[64] Smirne C (2019). Gas6/TAM signaling components as novel biomarkers of liver fibrosis. Dis. Markers.

[65] Sheng L (2017). Hepatic inflammation caused by dysregulated bile acid synthesis is reversible by butyrate supplementation. J. Pathol..

[66] Georgiev P (2008). Characterization of time-related changes after experimental bile duct ligation. Br. J. Surg..

[67] Inagaki T (2005). Fibroblast growth factor 15 functions as an enterohepatic signal to regulate bile acid homeostasis. Cell Metab..

[68] Schaap FG, van der Gaag NA, Gouma DJ, Jansen PL (2009). High expression of the bile salt-homeostatic hormone fibroblast growth factor 19 in the liver of patients with extrahepatic cholestasis. Hepatology.

[69] Tag CG (2015). Induction of experimental obstructive cholestasis in mice. Lab Anim..

[70] Li J (2016). Probiotics modulated gut microbiota suppresses hepatocellular carcinoma growth in mice. Proc. Natl Acad. Sci. USA.

[71] Lahti, L. & Shetty, S. *microbiome R package*, https://bioconductor.org/packages/microbiome/ (2019).

[72] Harrell, F. E. Jr & Dupont, C. *Hmisc: Harrell Miscellaneous*, https://CRAN.R-project.org/package=Hmisc (2022).

[73] Oksanen, J. et al. *vegan: Community Ecology Package*, https://CRAN.R-project.org/package=vegan (2020).

