# Supplementary material for: Impaired flux of bile acids from the liver to the gut reveals microbiome-immune interactions associated with liver damage
Source: NPJ Biofilms Microbiomes. 2023 Jun 7;9:35. doi: 10.1038/s41522-023-00398-0 (PMC10247725; doi:10.1038/s41522-023-00398-0)
Supplement: Supplementary file 1 — Supplementary Information [file 41522_2023_398_MOESM1_ESM.pdf]

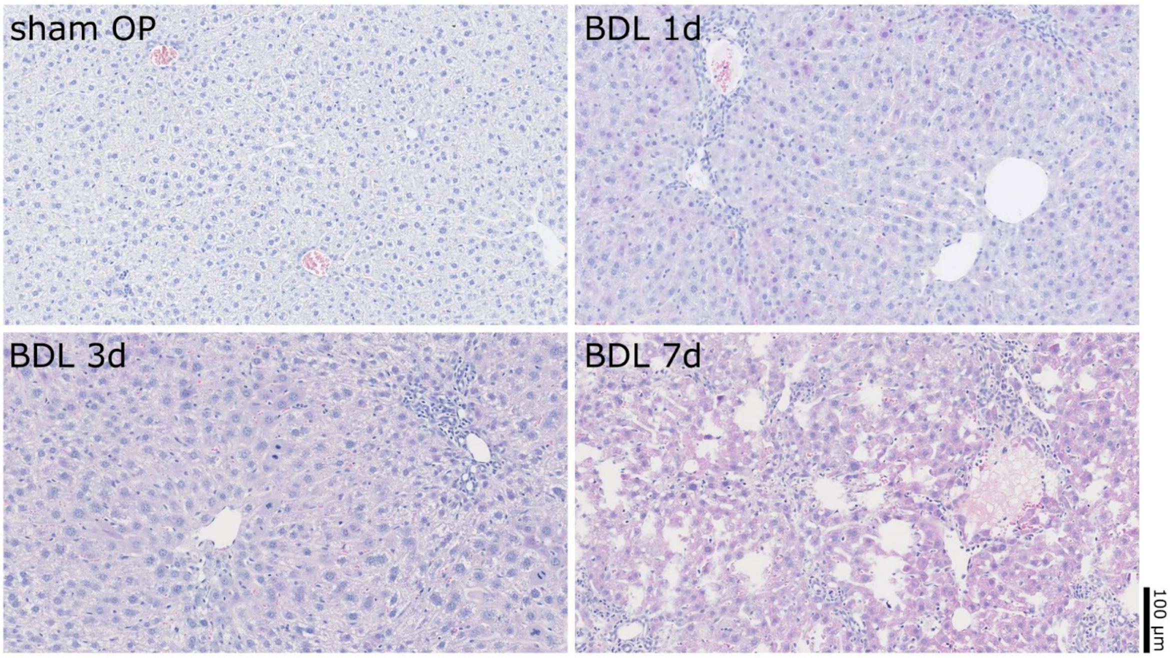

Supplementary Fig. 1. Comparison of H&E images of the liver of BDL and ShamOP mice.

H&E images assessing the degree of liver damage in BDL and ShamOP mice.

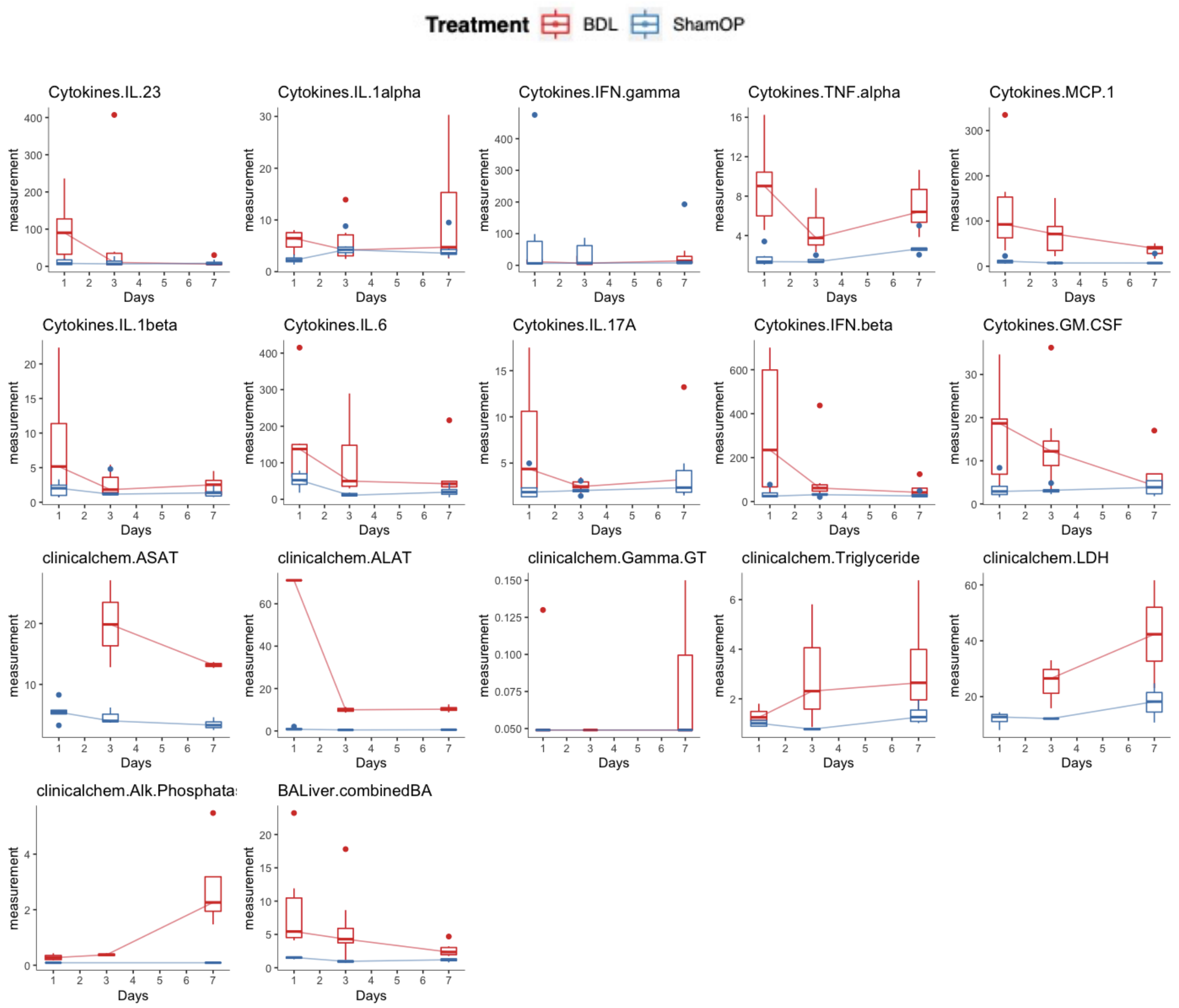

Supplementary Fig. 2. Comparison of biochemical markers and bile acids profiles of BDL and ShamOP mice. Boxplots of significantly different cytokines, bile acids and clinical chemicals. The centre line indicates the median of the data, the bounds of the box represent the interquartile range, and the whiskers indicate the range of the data, excluding outliers.

Supplementary Table 1. Biochemical markers, cytokines and bile acids.

treatment	day.feces.harvest	day.animals.harvest	seq.id	mouse.id	Cytokines-IL-23	Cytokines-IL-1α	Cytokines-IFN-γ	Cytokines-TNF-α	Cytokines-MCP-1	Cytokines-IL-1β	Cytokines-IL-10	Cytokines-IL-6	Cytokines-IL-27	Cytokines-IL-17A	Cytokines-IFN-β	Cytokines-GM-CSF
BDL	1	1	103	73965	8.1	8.05	2.58	10.42	164.56	0.76	4.98	38.39	58.69	1.59	21.33	1.85
BDL	1	1	104	73966	236.19	5.8	18.16	7.63	66.45	22.35	164.9	415.03	2405.91	5.56	156.4	18.82
BDL	1	1	105	73967	111.7	7.11	14.02	10.43	334.7	2.51	39.54	130.1	5138.39	3.17	700.57	34.65
BDL	1	1	112	73877	132.94	4.42	33.4	5.44	61.47	12.55	68.99	145.24	3643.21	17.49	694.47	18.55
BDL	1	1	113	73878	20.5	7.69	3.75	16.24	118.71	1.54	13.87	36.75	66.27	2.01	36.57	3
BDL	1	1	114	73879	68.78	3.13	8.96	4.57	35.03	7.82	51.54	151.51	1839.9	12.29	312.56	19.95
BDL	3	3	79	73847	16.51	13.92	53.35	8.81	150.84	1.4	276.42	289.54	259.99	2.43	437.08	13.57
BDL	3	3	80	73848	4.7	4.17	23.76	3.23	67.72	1.6	26.2	35.56	94.9	2.1	64.88	13.34
BDL	3	3	81	73849	4.7	6.99	2.44	5.44	94.13	1.11	50.98	44.12	41.95	1.77	31.41	3.16
BDL	3	3	82	73959	33.7	4.2	9.7	2.5	22.25	5.41	146.9	55.71	258.46	3.26	83.24	17.55
BDL	3	3	83	73960	4.7	3.25	2.24	2.33	35.01	1.33	19.92	29.86	89.13	3.46	45.56	3.8
BDL	3	3	84	73961	407.01	7.51	43.5	4.29	74.83	4.58	12.37	106.26	202.57	2.87	47.88	11.08
BDL	3	3	89	73825	4.7	2.47	3.23	6.87	86.02	3.27	47.69	272.49	670.43	2.49	73.48	10.59
BDL	3	3	90	73958	39.27	2.52	2.89	3.23	34.27	2.04	217.63	35.94	268.45	2.41	57.56	36.24
BDL	7	7	37	73826	30.2	15.31	4.76	10.66	50.71	3.16	32.61	34.12	77.95	3.23	41.34	17
BDL	7	7	38	73827	no blood sam	no blood sam	no blood sam	no blood sam	no blood sam	no blood sam	no blood sam	no blood sam	no blood sam	no blood sam	no blood sam	no blood sam
BDL	7	7	45	73832	4.7	4.02	7.52	3.85	16.88	4.51	12.64	42.49	1245.96	13.22	124.53	6.96
BDL	7	7	46	73957	4.7	2.57	14.27	5.35	39.74	1.09	22.62	33.22	13.46	2.08	19.22	2.94
BDL	7	7	47	73982	8.56	30.3	45.94	8.68	43.44	2.53	56.88	216.31	183.26	3.56	60.88	4.25
BDL	7	7	48	73983	6.09	4.74	28.12	6.4	28.35	1	39.03	49.64	45.04	1.67	26.86	3.65
sham OP	1	1	106	73968	4.7	2.23	475.08	1.19	13.04	3.3	101.81	39.46	37.28	1.3	21.45	1.97
sham OP	1	1	107	73850	4.7	2.2	3.17	1.18	6.51	2.51	14.14	18.23	126.68	2.37	43.13	2.99
sham OP	1	1	108	73851	20.9	3.35	6.41	3.4	22.71	2.33	12.95	78.49	1323.01	2.19	27.1	4.44
sham OP	1	1	109	73852	11.17	2.78	5.91	1.93	10.16	1.72	197.33	45.45	6423.92	4.98	77.13	8.38
sham OP	1	1	110	73853	4.7	1.83	98.81	1.05	5.84	0.74	1.8	59.6	16.85	<1.43	21.9	1.53
sham OP	1	1	111	73876	19.4	1.37	4.75	1.51	12.11	0.71	99.65	73.31	29.73	1.53	20.1	2.8
sham OP	3	3	77	73845	5.5	3.56	6.27	2.01	7.87	1.08	41.47	17.68	58.69	1.97	31.65	3.21
sham OP	3	3	78	73846	27.23	8.8	8.09	1.65	10.77	1.03	8.77	18.45	21.24	1.45	20.77	3.1
sham OP	3	3	85	73962	7.79	3.16	86.86	1.26	6.18	4.8	23.88	11.23	405.79	3.08	36.69	2.26
sham OP	3	3	86	73963	4.7	4.86	4.13	1.34	8.27	1.33	37.54	7.62	78.5	1.88	39.7	2.67
sham OP	3	3	87	73964	16.23	4.07	80.34	1.3	6.74	1.19	48.58	9.06	79.6	2.15	31.04	4.83
sham OP	3	3	88	73880	4.7	4.44	2.74	1.32	3.64	1.14	7.8	11.26	117.85	2.1	29.24	3.28
sham OP	7	7	39	73843	9.95	9.5	7.01	2.45	7.27	1.32	448.28	13.72	150.89	4.97	26.74	5.4
sham OP	7	7	40	73844	11.25	3.32	6.02	2.66	7.58	1.4	22.52	14.34	203.29	2.48	23.49	5.44
sham OP	7	7	41	73828	6.2	3.49	13.6	2.05	5.8	0.75	5.82	44.62	37.28	1.73	19.11	2.34
sham OP	7	7	42	73829	18.09	2.75	192.8	2.65	6.27	2.41	70.09	25.26	250.84	2.21	33.48	5.33
sham OP	7	7	43	73830	4.7	3.63	5.06	2.74	7.14	0.76	7.83	5.55	70.97	1.5	20.77	1.79
sham OP	7	7	44	73831	4.7	4.49	8.39	5.01	28.09	1.54	3.01	27.1	398.34	4.76	49.18	2.38

mouse.id	clinicalchemistry-Albumin	clinicalchemistry-ASAT	clinicalchemistry-ALAT	clinicalchemistry-GLDH	clinicalchemistry-Gamma-GT	clinicalchemistry-Triglyceride	clinicalchemistry-LDH	clinicalchemistry-Alk. Phosphatase
73965	27	nd	nd	6	<0.05	1.1	nd	nd
73966	22	nd	nd	nd	<0.05	1.56	nd	0.35
73967	26	nd	71.07	nd	<0.05	1.28	nd	0.20
73877	20	nd	nd	nd	<0.05	1.22	nd	0.17
73878	24	nd	nd	nd	nd	1.80	nd	0.43
73879	19	nd	nd	nd	0.13	1.1	nd	0.27
73847	nd	nd	nd	nd	nd	nd	nd	nd
73848	nd	nd	nd	nd	nd	nd	nd	nd
73849	nd	nd	nd	nd	nd	nd	nd	nd
73959	45	19.85	8.62	948	<0.05	2.31	>32.54	0.32
73960	33	27.09	11.30	6,141	nd	5.81	26.54	nd
73961	nd	nd	nd	nd	nd	nd	nd	nd
73825	26	12.83	nd	nd	<0.05	0.86	15.87	0.43
73958	nd	nd	nd	nd	nd	nd	nd	nd
73826	nd	12.70	10.46	5,704	<0.05	6.78	nd	5.48
73827	no blood sam	no blood sam	no blood sam	no blood sam	no blood sam	no blood sam	no blood sam	no blood sam
73832	43	nd	nd	nd	0.15	1.14	nd	1.47
73957	44	13.67	8.76	nd	<0.05	3.06	23.05	2.42
73982	39	nd	12.51	nd	nd	2.23	61.63	2.1
73983	nd	nd	10.04	nd	nd	nd	nd	nd
73968	nd	5.22	0.67	nd	nd	nd	nd	nd
73850	26	5.12	1.18	1,850	<0.05	1.14	12.12	<0.09
73851	nd	8.28	0.78	nd	nd	nd	nd	nd
73852	27	5.80	0.79	624	<0.05	0.88	13.38	nd
73853	27	5.66	2.06	1,954	<0.05	0.90	14.44	<0.09
73876	23	3.27	0.65	790	<0.05	1.12	8.07	<0.09
73845	nd	nd	nd	nd	nd	nd	nd	nd
73846	nd	nd	nd	nd	nd	nd	nd	nd
73962	nd	3.72	0.47	nd	nd	nd	nd	nd
73963	nd	6.21	nd	nd	nd	0.81	nd	nd
73964	nd	4.01	nd	294	nd	0.74	12.16	nd
73880	nd	nd	nd	nd	nd	nd	nd	nd
73843	38	4.61	0.67	nd	nd	1.95	nd	<0.09
73844	nd	nd	nd	nd	nd	nd	nd	nd
73828	38	3.59	0.58	271	<0.05	1.11	24.77	<0.09
73829	42	2.51	0.43	219	<0.05	1.02	18.23	<0.09
73830	nd	nd	nd	nd	nd	nd	nd	nd
73831	42	3.03	0.53	158	<0.05	1.41	10.76	<0.09

mouse.id	BALiver-TCA	BALiver-GCA	BALiver-GCDCA	BALiver-TCDCA	BALiver-TLCA	BALiver-GLCA	BALiver-TDCA	BALiver-GDCA	BALiver-CA	BALiver-CDCA	BALiver-UDCA	BALiver-DCA	BALiver-TUDCA	BALiver-GUDCA	BALiver-LCA
73965	5.4	0.05	0.05	0.134	0.05	0.05	0.06	0.05	0.05	0.05	0.05	0.025	0.0509	0.03	0.06
73966	3.97	0.05	0.05	0.116	0.05	0.05	0.06	0.05	0.05	0.05	0.05	0.025	0.05	0.03	0.06
73967	3.8	0.05	0.05	0.0453	0.05	0.05	0.06	0.05	0.05	0.05	0.05	0.025	0.05	0.03	0.06
73877	10.9	0.05	0.05	0.23	0.05	0.05	0.0986	0.05	0.162	0.05	0.05	0.025	0.05	0.03	0.06
73878	21.7	0.05	0.05	0.344	0.05	0.05	0.213	0.05	0.451	0.05	0.05	0.025	0.0505	0.03	0.06
73879	3.45	0.05	0.05	0.04	0.05	0.05	0.06	0.05	0.05	0.05	0.05	0.025	0.05	0.03	0.06
73847	7.98	0.05	0.05	0.04	0.05	0.05	0.06	0.05	0.05	0.05	0.05	0.025	0.05	0.03	0.06
73848	4.03	0.05	0.05	0.04	0.05	0.05	0.06	0.05	0.05	0.05	0.05	0.025	0.05	0.03	0.06
73849	3.23	0.05	0.05	0.04	0.05	0.05	0.06	0.05	0.05	0.05	0.05	0.025	0.05	0.03	0.06
73959	0.519	0.05	0.05	0.04	0.05	0.05	0.06	0.05	0.0697	0.05	0.05	0.025	0.05	0.03	0.06
73960	3.17	0.05	0.05	0.0744	0.05	0.05	0.06	0.05	0.05	0.05	0.05	0.025	0.05	0.03	0.06
73961	2.68	0.05	0.05	0.04	0.05	0.05	0.06	0.05	0.05	0.05	0.05	0.025	0.05	0.03	0.06
73825	4.17	0.05	0.05	0.127	0.05	0.05	0.06	0.05	0.121	0.05	0.05	0.025	0.05	0.03	0.06
73958	16.8	0.05	0.05	0.202	0.05	0.05	0.06	0.05	0.227	0.05	0.05	0.025	0.05	0.03	0.06
73826	1.74	0.05	0.05	0.845	0.05	0.05	0.06	0.05	0.05	0.05	0.05	0.025	0.05	0.03	0.06
73827	3.1	0.05	0.05	0.957	0.05	0.05	0.06	0.05	0.05	0.05	0.05	0.025	0.05	0.03	0.06
73832	1.09	0.05	0.05	0.04	0.05	0.05	0.06	0.05	0.05	0.05	0.05	0.025	0.05	0.03	0.06
73957	0.989	0.05	0.05	0.597	0.05	0.05	0.06	0.05	0.05	0.05	0.05	0.025	0.05	0.03	0.06
73982	0.585	0.05	0.05	0.656	0.05	0.05	0.06	0.05	0.05	0.05	0.05	0.025	0.0614	0.03	0.06
73983	1.02	0.05	0.05	0.8	0.05	0.05	0.06	0.05	0.05	0.05	0.05	0.025	0.104	0.03	0.06
73968	0.986	0.05	0.05	0.04	0.05	0.05	0.06	0.05	0.0569	0.05	0.05	0.025	0.05	0.03	0.06
73850	0.899	0.05	0.05	0.04	0.05	0.05	0.06	0.05	0.05	0.05	0.05	0.025	0.05	0.03	0.06
73851	0.961	0.05	0.05	0.04	0.05	0.05	0.06	0.05	0.05	0.05	0.05	0.025	0.05	0.03	0.06
73852	0.727	0.05	0.05	0.04	0.05	0.05	0.06	0.05	0.05	0.05	0.05	0.025	0.05	0.03	0.06
73853	0.598	0.05	0.05	0.04	0.05	0.05	0.06	0.05	0.05	0.05	0.05	0.025	0.05	0.03	0.06
73876	0.857	0.05	0.05	0.04	0.05	0.05	0.06	0.05	0.05	0.05	0.05	0.025	0.05	0.03	0.06
73845	0.44	0.05	0.05	0.04	0.05	0.05	0.06	0.05	0.05	0.05	0.05	0.025	0.05	0.03	0.06
73846	0.398	0.05	0.05	0.04	0.05	0.05	0.06	0.05	0.05	0.05	0.05	0.025	0.05	0.03	0.06
73962	0.146	0.05	0.05	0.04	0.05	0.05	0.06	0.05	0.05	0.05	0.05	0.025	0.05	0.03	0.06
73963	0.0856	0.05	0.05	0.04	0.05	0.05	0.06	0.05	0.05	0.05	0.05	0.025	0.05	0.03	0.06
73964	0.279	0.05	0.05	0.04	0.05	0.05	0.06	0.05	0.05	0.05	0.05	0.025	0.05	0.03	0.06
73880	0.303	0.05	0.05	0.04	0.05	0.05	0.06	0.05	0.05	0.05	0.05	0.025	0.05	0.03	0.06
73843	0.387	0.05	0.05	0.04	0.05	0.05	0.06	0.05	0.05	0.05	0.05	0.025	0.05	0.03	0.06
73844	0.129	0.05	0.05	0.04	0.05	0.05	0.06	0.05	0.05	0.05	0.05	0.025	0.05	0.03	0.06
73828	0.412	0.05	0.05	0.04	0.05	0.05	0.06	0.05	0.05	0.05	0.05	0.025	0.05	0.03	0.06
73829	0.646	0.05	0.05	0.04	0.05	0.05	0.06	0.05	0.05	0.05	0.05	0.025	0.05	0.03	0.06
73830	0.686	0.05	0.05	0.04	0.05	0.05	0.06	0.05	0.05	0.05	0.05	0.025	0.05	0.03	0.06
73831	0.669	0.05	0.05	0.04	0.05	0.05	0.06	0.05	0.05	0.05	0.05	0.025	0.05	0.03	0.06

Supplementary Table 2. Comparison of gut microbiome taxonomy profiles of BDL and ShamOP mice. Alpha and beta diversities of genus and species were compared. A linear mixed model was used to compare alpha diversities. Permutational multivariate analysis of variance was used to analyze beta diversities. P-values are shown.

	Alpha diversity		
	**Shannon**	**Simpson**	**Richness**
**Genus**	0.067	0.093	0.171
**Species**	0.276	0.076	0.612

	Beta diversity			
	**Day 0**	**Day 1**	**Day 3**	**Day 7**
**Genus**	0.142	0.001	0.132	0.014
**Species**	0.168	0.001	0.019	0.011

Supplementary Table 3. Linear model (LM) result for the genus. A positive coefficient indicates BDL mice > ShamOP mice, negative indicates the opposite. P-values and FDR-adjusted P-values are shown.

**feature**	**mean normalized abundance BDL day1**	**mean normalized abundance BDL day3**	**mean normalized abundance BDL day7**	**mean normalized abundance shamOP day1**	**mean normalized abundance shamOP day3**	**mean normalized abundance shamOP day7**	**coef**	**P-value**	**FDR**
Lachnospiraceae gen. incertae sedis	-1.7917417	-0.654831	-0.0577765	0.39072269	1.21689034	2.73181832	-2.1456089	4.73E-07	2.27E-05
Enterococcus	7.38297572	2.7392937	2.65393202	0.22986979	0.43967063	2.46556051	4.56627459	8.05E-06	0.00013149
Muribaculaceae gen. incertae sedis	0.03593368	-0.2644604	0.28507115	-0.8544997	-1.2497825	-2.00303	1.09502382	8.22E-06	0.00013149
Eubacterium	-2.726488	-2.0244285	-0.157235	0.81728803	2.02333939	3.84531766	-3.7733468	3.46E-05	0.00034572
Staphylococcus	2.41158716	0.42115441	0.99293733	-0.905655	-1.1784298	-3.1595991	2.82381459	4.24E-05	0.00034572
Parabacteroides	2.04499915	3.40061505	0.55618745	0.26115564	-0.5353145	-1.1363287	2.52622116	4.32E-05	0.00034572
Anaerotruncus	-0.0882918	-1.287707	-1.9230292	0.58272334	1.46108709	1.50602345	-1.7300003	0.00010615	0.0007279
Lachnoclostridium	-1.095187	0.36678083	0.39962503	0.64548145	1.31852184	2.52948746	-1.5077485	0.00019082	0.00106281
Desulfovibrio	-0.3857422	-1.668732	-1.9726168	0.58843736	0.76000555	2.66376372	-1.951931	0.00019928	0.00106281
Escherichia	6.19576934	3.58261048	5.92068526	1.08381202	1.31373876	3.45857002	3.76538159	0.00109494	0.00525572
Ruminococcaceae gen. incertae sedis	-0.9396093	0.13290824	-1.1185995	0.32802055	0.8337334	2.02392869	-1.298236	0.00232203	0.01013249
Blautia	-0.6447944	-1.323957	0.81774051	1.31579085	1.06772035	1.66754612	-1.9867416	0.00278504	0.01091475
Dorea	-1.0406605	-0.7815334	1.08619598	0.3406971	0.99752678	3.40628066	-1.6468817	0.00295608	0.01091475
Desulfovibrionaceae gen. incertae sedis	-0.3313752	-0.2849169	-0.5354272	0.68226905	0.54153964	0	-0.8864486	0.00332015	0.01138338
Erysipelotrichaceae gen. incertae sedis	-0.2034384	-2.2484874	-2.1735628	0.53168063	0.13973975	0.96525384	-1.6293734	0.0059634	0.01908288
Muribaculum	0.7322264	-0.0867621	1.37403629	-0.1353524	-0.4631504	-2.6654705	1.09593388	0.00769944	0.02309833
Prevotella	0.16261397	0.91391364	-0.7764757	-0.3713651	-1.163636	-1.8785218	1.14271282	0.01009706	0.0284931
Candidatus Gastranaerophilales gen. incertae sedis	0.5132002	2.64364874	0.01310828	0.14710264	-1.4218018	0	1.59316628	0.01068491	0.0284931
Alphaproteobacteria gen. incertae sedis	1.45926709	2.03458848	-0.6814115	0.6334311	-0.8993039	0	1.35300927	0.01271036	0.03211038
Pseudomonas	-0.2121613	-0.6428649	-1.0356597	0.21470054	0.60184312	1.30146023	-0.9500227	0.02665596	0.06219077
Prevotellaceae gen. incertae sedis	0.9801303	0.26269538	0.72036995	0.24833584	-0.4163649	0	0.6964101	0.02720846	0.06219077
Ruminococcus	0.46595065	0.09356984	-0.3964017	0.0700493	-1.4951098	-2.1203204	0.95919101	0.03031416	0.06613999
Bacteroidaceae gen. incertae sedis	1.21851853	1.96094387	0	0.08460983	-0.8537587	-1.4280008	1.75387175	0.04580446	0.09559191
Azospirillum	1.7018857	1.57192001	-2.6420825	0.68890926	-1.3410804	0	1.19062141	0.05809717	0.11619434
Bifidobacterium	0.06784615	1.41620993	5.17879697	1.36977568	4.27606607	5.64965445	-1.7129663	0.06309771	0.12114761
Oscillibacter	-0.588493	-0.0302142	-0.8469406	0.09615462	0.12806274	1.79269322	-0.7518591	0.07606645	0.14043037
Clostridium	-0.9098455	-0.0687351	0.44242696	-0.0144619	0.13542313	1.52774668	-0.6834106	0.09661222	0.17175506
Burkholderiales gen. incertae sedis	1.17171399	-0.7386321	2.16653443	0.1126729	-1.1355571	-0.4637562	0.98932618	0.14394669	0.24676576
Clostridiales Family XIII. Incertae Sedis gen. incertae sedis	1.1703459	-0.9829694	0.28905333	-1.1077277	-0.1543992	0.81776915	0.8418748	0.16837778	0.27087881
Akkermansia	0.10644709	0.29713317	0	0.29469129	-1.4506684	-2.4727941	0.80201507	0.17183588	0.27087881
Clostridiaceae gen. incertae sedis	-0.2063868	-1.9431691	2.64436139	-0.6393792	1.06928618	2.6484644	-0.8248414	0.17494256	0.27087881
Erysipelatoclostridium	-0.1351818	-0.698055	-1.5040812	0.21986363	-0.3501822	0	-0.5064045	0.18116105	0.27174157
Firmicutes gen. incertae sedis	-0.7621451	-1.9812804	-0.8640285	-0.5721319	-1.0585974	1.52765753	-0.7505816	0.27568094	0.40099046
Enterorhabdus	2.10538482	1.2505789	4.83971608	1.02403643	1.88475708	3.5705744	0.49703927	0.29631899	0.41833269
Cutibacterium	-0.2647761	-0.7122948	-1.498887	-0.3579895	-0.6981196	1.2810381	-0.3202917	0.31192775	0.42778663
Hungateiclostridiaceae gen. incertae sedis	-0.0601961	2.14535081	2.02071405	0.14869296	0.49851092	1.79192971	0.49684777	0.35621901	0.46930227
Olsenella	1.31938858	-0.4982465	-1.2870126	0.42080959	-0.3770654	-2.181458	0.53870301	0.36175383	0.46930227
Bacteroides	0.77436903	1.41911406	0.21099305	0.50357989	0.47213485	0.71696859	0.41239167	0.38882088	0.47432135
Bacteria gen. incertae sedis	-0.0046977	-1.6360335	-1.2864121	-0.3022116	-0.1753298	0	-0.5186955	0.39330719	0.47432135
Clostridiales gen. incertae sedis	0.46902973	-0.1022326	-1.245798	-0.0278302	0.47806026	2.29539965	-0.3866626	0.39526779	0.47432135
Eggerthellaceae gen. incertae sedis	0.76934917	0.11154244	1.56527936	0.57245623	1.16746489	1.8531371	-0.3018464	0.4283229	0.49276961
Candidatus Melainabacteria gen. incertae sedis	-0.5131887	-0.496619	-1.0400191	-0.626435	0.26836698	1.76340838	-0.5527288	0.43117341	0.49276961
Lactobacillus	-0.120286	0.32148584	3.89988744	-1.4295795	1.09367643	3.76940721	0.44833447	0.44377143	0.49537275
Clostridia gen. incertae sedis	-0.2610018	0.72128188	-0.1168038	0.27020973	0.38626689	0.6517118	-0.2537724	0.47637873	0.51968589
Roseburia	-0.5720198	-0.2114647	0.82872678	0.04307812	-0.446118	2.99354172	-0.5427952	0.50199303	0.53545923
Bacteroidales gen. incertae sedis	-0.1928542	-0.2247586	-1.0113649	-0.076808	0.455672	-1.4960958	-0.2158668	0.57467899	0.59966504
Odoribacter	0.23087095	-1.0913625	-0.2487996	0.51285298	-1.2662812	-2.0817712	0.11502885	0.86661867	0.88505736
Alistipes	0.29369074	-0.0307659	-2.0813666	-0.0274491	-0.4807745	0.34335077	0.01505917	0.9603041	0.9603041

Supplementary Table 4. Linear model (LM) result for species, comparing BDL and ShamOP mice. A positive coefficient indicates BDL mice > ShamOP mice, negative indicates the opposite. P-values and FDR-adjusted P- values are shown. Additional Wilcox-test result comparing species abundance at baseline and post surgery. P-values are shown.

feature	mean normalized abundance BDL day1	mean normalized abundance BDL day3	mean normalized abundance BDL day7	mean normalized abundance shamOP day1	mean normalized abundance shamOP day3	mean normalized abundance shamOP day7	coef	LM P-value	LM FDR	Wilcox-test P-value (BDL day0 vs day1)	Wilcox-test P-value (BDL day 0 vs day 3)	Wilcox-test P-value (BDL day 0 vs day 7)	Wilcox-test P-value (shamOP day 0 vs day 1)	Wilcox-test P-value (shamOP day 0 vs day 3)	Wilcox-test P-value (shamOP day 0 vs day 7)
[Clostridium] cocleatum [ref_mOTU_v25_11811]	-0.1356535	-0.699611	-1.5067418	0.22030038	-0.3508401	0	-0.507549	0.18093175	0.27707499	0.93264664	0.85513214	1	0.42267807	1	NA
Akkermansia muciniphila [ref_mOTU_v25_03591]	0.09736494	0.29714215	0	0.29992234	-1.4447497	-2.4754087	0.79284001	0.17645427	0.27337986	1	1	NA	0.90569544	0.14221324	0.37109337
Alistipes species incertae sedis [ext_mOTU_v26_17309]	1.88899871	0.69475911	-2.9728392	1.04135304	0.12055195	-1.631969	0.4817729	0.23922575	0.33736964	3.05E-05	0.16015625	0.25	0.0384903	0.73339844	0.09375
Alistipes species incertae sedis [ext_mOTU_v26_18761]	-0.5802653	-1.082784	-0.8795062	-0.342592	-0.5313053	0.00703902	-0.4363822	0.52039128	0.59981302	0.03353882	0.00976563	0.75	0.63969421	0.56933594	0.0625
Alistipes species incertae sedis [ext_mOTU_v26_18779]	-0.8686512	-1.645031	-4.0685154	-1.148734	-4.2857347	-4.8326178	1.12532705	0.10809561	0.18873837	0.00628662	0.13085938	0.25	0.04827881	0.00927734	0.03125
Alistipes species incertae sedis [ext_mOTU_v26_18785]	1.53607318	0.66460522	-2.3797179	-0.3649248	-3.6269738	-4.3051331	2.69948025	8.18E-05	0.00087095	0.00057983	0.04882813	0.25	0.70188141	0.00683594	0.0625
Alistipes species incertae sedis [ext_mOTU_v26_18827]	-1.3268535	-4.3696626	-5.8407628	0.99290956	-1.1221593	0.62509394	-3.2089485	0.00037453	0.00240758	0.01578549	0.01781719	0.25	0.33876302	0.47719662	0.4375
Alistipes species incertae sedis [ext_mOTU_v26_18865]	0.39470501	0.28945999	-0.4967092	0.62247989	-0.3317885	0	0.02034466	0.94592057	0.95168837	0.04231527	0.28071267	1	0.10564543	0.18144921	NA
Alistipes species incertae sedis [ext_mOTU_v26_18944]	0.39377148	-0.4459451	-0.3580961	0.48487999	-0.0617738	0	-0.2377621	0.42386223	0.5161422	0.04231527	0.41849223	1	0.28071267	0.42267807	NA
Alphaproteobacteria species incertae sedis [ext_mOTU_v26_27739]	1.45940582	2.0348163	-0.6815748	0.63389321	-0.8993536	0	1.35290896	0.01274106	0.03593633	0.01426619	0.05905823	1	0.05905823	0.18144921	NA
Anaerotruncus sp. G3(2012) [ref_mOTU_v25_07507]	-0.0883656	-1.288691	-1.9257563	0.58242423	1.46132155	1.50630061	-1.7306732	0.00010685	0.00100749	0.49542236	0.01953125	0.75	0.04827881	0.02099609	0.03125
Azospirillum species incertae sedis [ext_mOTU_v26_18867]	1.70248093	1.57284175	-2.6448533	0.68940355	-1.3424476	0	1.19110073	0.05820629	0.11930482	0.01426619	0.28071267	1	0.05917207	0.10034825	NA
Bacteria species incertae sedis [ext_mOTU_v26_23316]	-0.0700118	-1.1807657	-1.4918654	-0.0299501	0.21530337	0	-0.6921142	0.30632234	0.40113639	0.89390372	0.10564543	1	0.35254214	0.20124262	NA
Bacteria species incertae sedis [ext_mOTU_v26_33444]	-0.1046366	-1.7667812	-0.7596886	-0.0877816	-0.1704659	0	-0.6702904	0.19457922	0.29186883	0.82409817	0.07592696	1	0.28071267	0.42267807	NA
Bacteroidaceae species incertae sedis [meta_mOTU_v25_13034]	1.21965839	1.96343693	0	0.08425334	-0.8556931	-1.4305773	1.75651665	0.04580758	0.09690065	0.08313114	0.10831938	NA	0.6834809	0.52936811	0.78926803
Bacteroidales bacterium M1 [ref_mOTU_v25_10008]	-2.1666499	-1.9544224	2.10859126	-1.127729	-0.1896198	0.92330941	-1.0167219	0.05583922	0.11589272	0.00335693	0.19335938	0.25	0.0384903	0.42382813	0.4375
Bacteroidales bacterium M10 [ref_mOTU_v25_10015]	-0.2172124	-0.6845162	-1.5211949	-1.4559031	-2.8177697	-6.9860246	2.0861945	0.00088536	0.0047124	0.52816772	0.43164063	0.5	0.01040649	0.00244141	0.03125
Bacteroidales bacterium M11 [ref_mOTU_v25_10016]	-0.6661007	1.14514048	-2.058403	-1.3515988	-3.6212302	-5.5291021	2.44924501	8.76E-05	0.00089569	0.40374756	0.19335938	0.75	0.01383259	0.00385729	0.03125
Bacteroidales bacterium M12 [ref_mOTU_v25_10017]	-1.5912621	-0.776435	-0.0152283	-1.3715333	-3.4668705	-5.8167522	1.53291856	0.01335563	0.03735048	0.00100708	0.23242188	1	0.00336456	0.01611328	0.03125
Bacteroidales bacterium M14 [ref_mOTU_v25_10018]	2.28195605	0.8341672	-2.1159069	0.17600429	-1.819113	-3.0949035	2.13788928	0.00057357	0.00332065	0.00166169	0.10831938	0.5	0.62483487	0.01426619	0.10034825

Bacteroidales bacterium M6 [ref_mOTU_v25_10012]	0.22515335	-0.468375	2.92260855	-0.2222017	0.08084163	-1.2338783	0.56582754	0.242938	0.33970144	0.37545776	0.4921875	0.25	0.93228149	0.46972656	0.09375
Bacteroidales species incertae sedis [ext_mOTU_v26_18778]	-0.0267024	0.10582273	-1.1984802	0.35685003	0.36987109	-0.5419409	-0.3664609	0.47760752	0.56694418	0.59658813	0.43164063	0.5	0.19638824	0.56933594	0.6875
Bacteroidales species incertae sedis [ext_mOTU_v26_18918]	0.29673502	-0.4152786	-1.1164509	-0.4930777	-1.3352461	-3.0409673	0.97517827	0.05710166	0.11777217	0.34838867	0.19335938	0.5	0.76602936	0.96972656	0.03125
Bacteroidales species incertae sedis [ext_mOTU_v26_18943]	-1.32304	-2.1106945	-1.9735239	-0.7403648	-0.5005705	-2.9626193	-0.7281595	0.21615889	0.31289063	0.00213623	0.00390625	1	0.30379486	0.38037109	0.03125
Bacteroides acidifaciens [ref_mOTU_v25_03478]	1.16970372	1.59575897	0.28785179	0.62112799	0.38304655	0.3476925	0.70537611	0.12221165	0.20576452	0.00057983	0.02734375	1	0.08976746	0.73339844	1
Bacteroides caecimuris [ref_mOTU_v25_03476]	-0.0199944	0.63161152	1.06362409	0.1114543	1.06264624	1.46221065	-0.2571285	0.57844519	0.65596877	0.88884068	0.625	1	0.77638843	0.73339844	0.03125
Bacteroides sp. [ref_mOTU_v25_03475]	-1.5480956	-0.1158703	-1.0792399	-0.0585647	0.07769526	0.85085509	-1.0888884	0.09412562	0.16973357	0.01285124	0.40694122	1	0.47553266	0.78740649	0.37109337
Bacteroides species incertae sedis [ext_mOTU_v26_17311]	0.6596688	0.48034149	-0.8794759	0.12191043	-0.6206866	0	0.54509909	0.19774719	0.29394852	0.13190409	0.72628615	1	0.62483487	0.41849223	NA
Bacteroides species incertae sedis [ext_mOTU_v26_18754]	-0.5634428	0.34848259	-0.1512394	0.44928832	-0.082313	0	-0.4058872	0.25901107	0.35763035	0.06835303	0.28071267	1	0.40167817	0.42267807	NA
Bacteroides species incertae sedis [ext_mOTU_v26_33493]	0.86788532	1.71767858	-0.5905748	0.76093085	0.39167674	0	0.45020559	0.4587865	0.54854908	0.05966047	0.23395283	1	0.26640242	1	NA
Bifidobacterium pseudolongum [ref_mOTU_v25_03808]	0.06830528	1.41776639	5.18420845	1.37066519	4.27819031	5.6550504	-1.7133977	0.06321465	0.12746923	0.42267807	0.37109337	0.37109337	0.05191296	0.05905823	0.03125
Blautia species incertae sedis [ext_mOTU_v26_18824]	-0.6454314	-1.3262897	0.81778609	1.31674882	1.06854556	1.66781085	-1.9887189	0.00279165	0.01196422	0.34868808	0.55664063	0.37109337	0.08976746	0.12939453	0.09375
Burkholderiales bacterium YL45 [meta_mOTU_v25_13139]	-0.4998577	-0.4615547	1.871426	-0.2194605	-0.7651067	-1.4398059	0.36300041	0.62268266	0.69420702	0.94994449	0.63558612	0.5	1	0.56933594	0.15625
Burkholderiales bacterium YL45 [ref_mOTU_v25_10100]	1.26751347	-0.5478692	2.27837877	0.11552142	-1.019254	-0.2927061	1.05858758	0.11761805	0.20041513	0.00628662	0.375	0.25	0.96611786	0.73339844	0.6875
Candidatus Arthromitus sp. [ref_mOTU_v25_00944]	-0.4352636	-2.2937556	1.17932561	-0.101219	0.70899453	1.46319094	-1.265554	0.00922912	0.02928472	0.05917207	0.05905823	1	0.83393541	0.36131043	0.37109337
Candidatus Gastranaerophilales species incertae sedis [ext_mOTU_v26_18840]	0.51322504	2.64453206	0.01310871	0.1471359	-1.4225061	0	1.59370515	0.01071981	0.03215942	0.10732756	0.05905823	1	1	0.10034825	NA
Candidatus Melainabacteria species incertae sedis [ext_mOTU_v26_18847]	-0.5132049	-0.4974453	-1.0400617	-0.6272937	0.26781349	1.76235001	-0.5522503	0.43186425	0.52395295	0.175354	0.921875	0.5	0.41711426	0.62207031	0.15625
Clostridia species incertae sedis [ext_mOTU_v26_18828]	1.11037137	1.84190959	1.51554461	0.63981552	-0.0771693	0.8176491	0.99106919	0.04084883	0.08966792	0.04149109	0.02249427	0.25	0.26640242	0.67498671	0.42267807
Clostridia species incertae sedis [ext_mOTU_v26_18950]	-0.4317578	-0.6280696	-1.1841139	0.35473303	0.24252094	0	-0.8648558	0.0103993	0.03148413	0.05870741	0.17752985	1	1	0.78926803	NA
Clostridia species incertae sedis [ext_mOTU_v26_23357]	-0.1763752	0.30902491	1.27158219	-0.3755543	-0.5753974	0	0.56478435	0.14850415	0.24211323	0.67498671	0.36131043	1	0.20124262	0.18144921	NA
Clostridia species incertae sedis [ext_mOTU_v26_27808]	-0.8776656	0.79255067	-0.0984044	0.21967804	0.30923938	0.6996777	-0.5026494	0.20869949	0.30609258	0.08325195	0.03710938	1	0.26452637	0.56933594	0.15625
Clostridia species incertae sedis [ext_mOTU_v26_33428]	0.20213132	0.77967097	-0.1802276	-0.4611234	-0.4057203	0	0.7422265	0.08706523	0.16141307	0.44120852	0.28071267	1	0.29450739	1	NA
Clostridia species incertae sedis [ext_mOTU_v26_33471]	-0.1686795	-0.3692225	0.71258216	-0.4764464	-0.2847577	-1.2100561	0.37824227	0.55054051	0.62864487	1	0.83393541	1	0.35254214	1	0.37109337
Clostridiaceae species incertae sedis [ext_mOTU_v26_18986]	0.22922655	0.3480763	1.46777571	-0.5397654	0.36059596	1.18713294	0.44086636	0.2571591	0.35656513	0.58963855	1	0.37109337	0.41849223	0.28071267	0.10034825

Clostridiales Family XIII. Incertae Sedis species incertae sedis [ext_mOTU_v26_23391]	1.17143363	-0.9845974	0.28904857	-1.1091635	-0.154465	0.81912761	0.84248628	0.16875268	0.26518279	0.03603169	0.36272651	1	0.02019367	0.83846378	0.4375
Clostridiales species incertae sedis [ext_mOTU_v26_18730]	1.07509078	-0.3803342	-1.2440561	0.12307813	-0.2564574	0	0.29286394	0.55423758	0.63068414	0.02249427	0.58388242	1	1	1	NA
Clostridiales species incertae sedis [ext_mOTU_v26_18757]	-0.8495907	0.17471646	0.63041805	0.13532016	1.45097067	2.63057942	-1.1998134	0.00867846	0.02873773	0.93988037	0.55664063	0.5	0.56999607	0.00683594	0.0625
Clostridiales species incertae sedis [ext_mOTU_v26_18759]	-0.3647601	-1.1575867	-2.6790282	0.26739385	-0.1566652	0.36666039	-1.070615	0.09826655	0.1746889	0.52861213	0.14221324	0.37109337	0.37867469	0.72228296	0.21875
Clostridiales species incertae sedis [ext_mOTU_v26_18760]	-0.5521651	-3.2727562	-2.5372108	-0.1317495	0.75222708	3.45685316	-2.3885097	0.00122159	0.0059283	0.05065918	0.32226563	0.25	0.79870605	0.02685547	0.0625
Clostridiales species incertae sedis [ext_mOTU_v26_18764]	-0.0157252	-0.3301161	1.74484129	0.4946044	0.18813979	1.02955971	-0.3756683	0.51738716	0.59981302	0.93210694	0.6953125	0.75	0.70490705	0.75976583	0.36131043
Clostridiales species incertae sedis [ext_mOTU_v26_18769]	0.35237952	-0.6612197	-0.9496036	0.14432476	0.32759023	0.12621816	-0.3682562	0.35136986	0.44914858	0.17752985	1	1	0.41849223	0.58388242	0.78926803
Clostridiales species incertae sedis [ext_mOTU_v26_18775]	0.28604339	-0.1112758	0	0.08656423	-0.4750601	0.3211487	0.17703564	0.68145341	0.74959875	0.58963855	1	NA	0.82409817	0.58963855	0.37109337
Clostridiales species incertae sedis [ext_mOTU_v26_18798]	0.06383694	0.68154097	1.7667422	-0.2035739	0.25866951	-0.1507552	0.53641599	0.30216017	0.39967664	0.41019833	0.09720109	0.5	0.81289688	0.17626953	0.84375
Clostridiales species incertae sedis [ext_mOTU_v26_18813]	2.24691727	1.4542251	-1.6971436	0.29588696	-0.5314957	-2.2835124	1.79690775	0.04130159	0.08966792	0.0008919	0.09720109	0.5	0.04544695	0.44120852	0.10564543
Clostridiales species incertae sedis [ext_mOTU_v26_18814]	0.36198484	2.24287594	0	0.25737304	0.21513826	0	0.77410576	0.11032626	0.19161929	0.6834809	0.04231527	NA	0.79984611	0.20124262	NA
Clostridiales species incertae sedis [ext_mOTU_v26_18817]	-0.0707664	0.14877332	-2.3191589	0.1718613	-0.1411119	0	-0.3152513	0.45300303	0.54435628	0.72628615	0.78740649	1	0.28071267	0.78926803	NA
Clostridiales species incertae sedis [ext_mOTU_v26_18819]	-1.1756302	-3.8623984	-0.242036	-0.832419	1.3913497	3.99943621	-2.5441925	0.00037938	0.00240758	0.55629846	0.02249427	0.75	0.26452637	0.02099609	0.03125
Clostridiales species incertae sedis [ext_mOTU_v26_18823]	-1.2365935	-0.9005571	-0.4334073	-0.0036064	0.02874827	0	-1.0266016	0.00201605	0.00911365	0.14148212	0.17752985	1	1	1	NA
Clostridiales species incertae sedis [ext_mOTU_v26_18831]	-0.5199608	-0.0619587	-1.0009677	-0.3983013	-0.1073366	-0.4887519	-0.1019183	0.82802224	0.86197898	0.07592696	1	1	0.67498671	1	1
Clostridiales species incertae sedis [ext_mOTU_v26_18837]	0.98316071	-0.0112286	0	0.65799243	-0.5478451	-0.0935027	0.34654129	0.60164525	0.67762093	0.07592696	0.78926803	NA	0.35895144	0.2084128	0.85513214
Clostridiales species incertae sedis [ext_mOTU_v26_18839]	-0.1056449	0.28529621	-0.35131	0.35903609	0.5660582	0	-0.3770485	0.14730623	0.24211323	0.58963855	1	1	0.18144921	0.37109337	NA
Clostridiales species incertae sedis [ext_mOTU_v26_18844]	1.02377052	-0.3328684	-0.7356551	0.1096697	0.40770228	0	0.13461153	0.77003701	0.83043207	0.07592696	0.78926803	1	1	1	NA
Clostridiales species incertae sedis [ext_mOTU_v26_18853]	-1.6651994	-1.031008	0.14842072	0.02701856	0.81052893	3.0897884	-1.9044557	0.00040567	0.00250449	0.00057983	0.13085938	0.5	0.46829224	0.06396484	0.03125
Clostridiales species incertae sedis [ext_mOTU_v26_18862]	-0.3742502	-0.8528661	-0.8237371	0.91116081	0.68516955	1.33499681	-1.4915911	0.01525477	0.0402726	0.5633178	0.40694122	1	0.05249174	0.04149109	0.28071267
Clostridiales species incertae sedis [ext_mOTU_v26_18869]	1.64313656	0.54221301	0.65450042	0.71497845	0.24407235	0	0.66534749	0.08473542	0.15975317	0.02086258	0.28071267	1	0.05905823	0.18144921	NA
Clostridiales species incertae sedis [ext_mOTU_v26_18870]	-0.6870696	-3.6089982	-1.0355782	0.31152747	0.08923221	0	-1.9624542	0.0006979	0.00383842	0.08005792	0.05905823	1	0.78740649	0.78926803	NA
Clostridiales species incertae sedis [ext_mOTU_v26_18882]	-0.3483788	-1.444901	-0.2239225	0.79639797	-0.0396118	1.69596527	-1.3619287	0.01471687	0.03948428	0.04149109	0.20124262	1	0.05870741	0.29450739	0.36131043
Clostridiales species incertae sedis [ext_mOTU_v26_18884]	0.45894206	1.78867003	-0.2064864	-0.035055	-2.1298538	-0.0441392	1.57149907	0.00220721	0.00977307	0.18740119	0.00915169	0.75	0.30379486	0.26640242	0.78740649

Clostridiales species incertae sedis [ext_mOTU_v26_18897]	0.08838678	-1.2034518	-3.2447958	0.58110237	0.23807305	0	-1.1700255	0.02390213	0.05757448	0.78740649	0.37109337	1	0.18144921	1	NA
Clostridiales species incertae sedis [ext_mOTU_v26_18927]	-1.3853799	-1.3109335	-0.3448128	1.11053402	1.77835672	4.16170393	-2.9617068	1.73E-05	0.00036292	0.24429364	0.16015625	1	0.04179671	0.00488281	0.03125
Clostridiales species incertae sedis [ext_mOTU_v26_18928]	-1.2916145	-0.6988851	0.54054846	-0.0581957	0.3025811	1.85853716	-1.1689422	0.02178887	0.05488799	0.10732756	0.52936811	1	0.78740649	0.36131043	0.20124262
Clostridiales species incertae sedis [ext_mOTU_v26_18945]	-0.5064987	0.43104546	-0.438072	0.04905629	0.03252127	1.1508657	-0.3543741	0.35835404	0.44964575	0.66854858	0.43164063	0.5	1	0.50487987	0.03125
Clostridiales species incertae sedis [ext_mOTU_v26_18948]	-0.424228	0.98431042	0.1750025	-0.0738649	-1.3264253	0.31569096	0.58153598	0.35659675	0.44914858	0.75565774	0.81270369	1	0.39830459	0.72228296	0.85513214
Clostridiales species incertae sedis [ext_mOTU_v26_18953]	-0.3014295	-1.235993	0.5451439	0.36235446	0.24616647	0	-0.8053565	0.01228319	0.03494356	1	0.05905823	1	0.05905823	0.78926803	NA
Clostridiales species incertae sedis [ext_mOTU_v26_18954]	0.27312682	-0.2193526	0	1.02512742	1.46483096	0	-0.9694562	0.05560991	0.11589272	0.67498671	0.85513214	NA	0.05905823	0.18144921	NA
Clostridiales species incertae sedis [ext_mOTU_v26_18967]	-0.0826675	-1.3798165	-3.7603962	0.72942759	0.60506665	2.45365103	-1.9079853	0.00566388	0.0210009	0.41849223	0.41849223	0.37109337	0.25534036	0.14148212	0.15625
Clostridiales species incertae sedis [ext_mOTU_v26_18988]	-0.2415645	0.00477182	-1.066805	0.45238892	0.41666865	0.60872983	-0.7166688	0.11147337	0.19259797	0.6603461	0.63558612	1	0.23613697	0.02997397	0.37109337
Clostridiales species incertae sedis [ext_mOTU_v26_18993]	-0.3800248	-2.5134966	-1.3972127	-0.3884028	-0.2173472	4.18520823	-1.5287316	0.02228924	0.05530413	0.41849223	0.05905823	1	1	0.78926803	0.28071267
Clostridiales species incertae sedis [ext_mOTU_v26_19011]	-0.8094373	-0.3717972	0.06914023	-0.1038799	-0.1865869	0	-0.4293844	0.35543296	0.44914858	0.10831938	1	1	0.58963855	1	NA
Clostridiales species incertae sedis [ext_mOTU_v26_23321]	0.02923093	-0.1723119	-1.5146434	-0.19598	-0.4788836	-0.9059911	0.15060872	0.67726724	0.74748558	0.78926803	0.37109337	1	0.78740649	0.37109337	0.37109337
Clostridiales species incertae sedis [ext_mOTU_v26_23340]	-1.6808419	-2.465007	-1.0563475	-0.7423146	0.07417425	0.43720735	-1.5623723	0.00768513	0.02640466	0.00258448	0.03710938	1	0.45125897	0.68908375	0.58963855
Clostridiales species incertae sedis [ext_mOTU_v26_23356]	-0.9382541	-0.8241724	0.17314404	0.56338978	1.14955953	1.72847046	-1.6702342	0.00015213	0.00122572	0.01426619	0.28071267	1	0.19553886	0.05870741	0.05905823
Clostridiales species incertae sedis [ext_mOTU_v26_23378]	-1.3432598	0.43356344	0.78009595	0.40386911	0.46141007	1.40379895	-1.0077181	0.06763555	0.13365108	0.18740119	0.72228296	1	0.26640242	0.47719662	0.18144921
Clostridiales species incertae sedis [ext_mOTU_v26_27766]	-0.3857607	-0.9559255	-0.3431744	0.59799854	0.76595715	2.72720472	-1.5152028	0.00806332	0.02715199	0.6701287	0.63558612	0.75	0.04827881	0.02685547	0.0625
Clostridiales species incertae sedis [ext_mOTU_v26_27793]	-1.2177091	-0.3785055	-0.4020107	0.3885006	1.75376743	3.9566446	-2.1252741	0.00013642	0.00115436	0.23481129	0.47719662	1	0.26806676	0.01846609	0.03125
Clostridiales species incertae sedis [ext_mOTU_v26_27799]	-0.0138104	-1.2074615	-0.0624286	0.45682349	0.34636876	1.0092771	-0.9357219	0.011237	0.03310903	0.67260382	0.10034825	1	0.10732756	0.20124262	0.37109337
Clostridiales species incertae sedis [ext_mOTU_v26_27801]	-1.5868061	0.24877637	-0.5331382	0.81713097	1.99063143	3.93466162	-2.4188566	7.10E-06	0.0002132	0.05005797	0.43164063	0.5	0.02367401	0.00048828	0.03125
Clostridiales species incertae sedis [ext_mOTU_v26_27804]	-0.4246476	0.25533533	0.24743084	-0.1303635	0.92505042	3.97001877	-0.8556487	0.21712713	0.31289063	0.44323108	0.921875	1	0.9000858	0.05224609	0.03125
Clostridiales species incertae sedis [ext_mOTU_v26_27807]	-0.6398314	1.22604446	1.09371404	0.58340628	0.15699721	0.20061265	-0.1597772	0.77877654	0.83440343	0.6834809	0.12353425	1	0.16219923	0.94418251	0.58388242
Clostridiales species incertae sedis [ext_mOTU_v26_27811]	-0.9908491	0.84790666	0.98800155	2.06382695	2.05094797	5.92381614	-2.663574	0.00020756	0.00156006	0.57610354	0.375	1	0.00837385	0.04544695	0.03125
Clostridiales species incertae sedis [ext_mOTU_v26_27813]	-0.9211589	0.98377358	-0.4567639	0.10709907	0.26047801	0	-0.332839	0.45363023	0.54435628	0.40167817	0.05917207	1	0.36131043	1	NA
Clostridiales species incertae sedis [ext_mOTU_v26_27821]	-0.7372132	-0.0431429	0.14550014	-0.105402	0.11120816	0	-0.3626491	0.23685115	0.33545441	0.18343062	1	1	0.28071267	0.42267807	NA

Clostridiales species incertae sedis [ext_mOTU_v26_27822]	-0.7095123	-1.4323766	0.28503482	0.14333913	-0.4528938	0.94623887	-0.8969359	0.06334835	0.12746923	0.20928655	0.10732756	1	1	0.93264664	0.37109337
Clostridiales species incertae sedis [ext_mOTU_v26_27826]	-0.0235893	0.63070449	2.44301656	0.33602821	1.82122798	3.30692491	-0.7027166	0.30399647	0.39967664	1	0.921875	0.37109337	0.36360697	0.02099609	0.09375
Clostridiales species incertae sedis [ext_mOTU_v26_33426]	-0.506086	-0.8966955	0.98627992	1.02183617	0.50256539	0.95937908	-1.3048906	0.00879549	0.02873773	0.23002425	0.03461056	1	0.06921297	0.10831938	0.37109337
Clostridiales species incertae sedis [ext_mOTU_v26_33434]	-0.4995858	-1.199954	-1.3535849	-0.5806034	-0.1919318	-0.3608681	-0.4280279	0.42054943	0.51400486	0.41849223	0.18144921	1	0.39830459	0.52861213	1
Clostridiales species incertae sedis [ext_mOTU_v26_33467]	-1.4869873	-1.052483	-1.2969007	0.09664071	0.74372674	0.75411246	-1.704955	0.00107875	0.00555298	0.01905889	0.10564543	1	1	0.55411313	1
Clostridiales species incertae sedis [ext_mOTU_v26_33487]	-1.6814254	0.28870596	0.29356776	1.47573626	1.89618358	4.51895747	-2.7548162	4.97E-05	0.00060803	0.23296606	0.375	0.75	0.00902006	0.00804524	0.03125
Clostridiales species incertae sedis [ext_mOTU_v26_33501]	-0.8161031	1.67411958	2.6170976	-0.4479089	-0.1814673	0.32765083	0.74541563	0.30315374	0.39967664	0.55629846	0.03710938	0.5	0.6701287	1	0.58388242
Clostridiales species incertae sedis [ext_mOTU_v26_33502]	-0.422588	-0.2279976	0	0.06838664	-0.052054	0	-0.3217817	0.21678179	0.31289063	0.37109337	1	NA	0.78926803	1	NA
Clostridium sp. ASF502 [ref_mOTU_v25_03467]	-0.4249591	1.24844905	1.45721388	1.33558485	0.65882474	2.14302194	-0.8212673	0.08568579	0.15975317	0.22401538	0.02249427	0.25	0.01444518	0.09720109	0.05905823
Clostridium species incertae sedis [ext_mOTU_v26_18731]	-0.6633742	-0.154743	-0.1942966	0.46532861	0.21932359	0	-0.7492743	0.02635469	0.06212178	0.14148212	0.58963855	1	0.10564543	0.18144921	NA
Clostridium species incertae sedis [ext_mOTU_v26_18763]	-1.9215948	-1.1963221	-1.9177914	0.75960373	1.25203613	0.56246669	-2.5594176	9.24E-07	6.10E-05	0.00076294	0.84570313	0.25	0.01793752	0.00244141	0.21875
Clostridium species incertae sedis [ext_mOTU_v26_18792]	-0.4150847	-2.6010406	-3.3805284	-1.5571122	-2.334909	-4.7243572	0.67530308	0.40263702	0.49764126	0.67260382	0.10034825	1	0.03098405	0.72628615	0.4375
Clostridium species incertae sedis [ext_mOTU_v26_18820]	-0.6805974	-0.7926308	0.58993249	0.17752579	-0.1107498	0	-0.6242401	0.04197096	0.09052561	0.01426619	0.58963855	1	0.58963855	0.42267807	NA
Clostridium species incertae sedis [ext_mOTU_v26_18861]	-0.3250972	-3.4628225	0.28756542	0.1685299	0.1116255	2.18240326	-1.7757477	0.01031515	0.03148413	0.34838867	0.00585938	0.75	0.26452637	0.67724609	0.09375
Clostridium species incertae sedis [ext_mOTU_v26_18883]	-1.4406051	-0.5437051	-1.5043825	0.71374589	-1.0684573	0.22391215	-1.1957743	0.22326565	0.31919279	0.06835303	1	0.75	0.09008206	0.55361699	0.3125
Clostridium species incertae sedis [ext_mOTU_v26_18888]	-0.4138343	0.17757787	-1.2396101	0.01731387	0	0	-0.3149321	0.40791894	0.500421	0.58388242	1	1	1	NA	NA
Clostridium species incertae sedis [ext_mOTU_v26_18914]	-0.4489545	-0.3398221	1.27115812	0.0464942	-0.1570866	0	-0.173105	0.61200721	0.68466004	0.20489389	0.41849223	1	1	0.78926803	NA
Clostridium species incertae sedis [ext_mOTU_v26_18932]	-0.6957338	0.38243764	-2.3029838	0.7916284	0.63078175	0.44419609	-1.2040993	0.00920157	0.02928472	0.09720109	0.78740649	1	0.02439024	0.05905823	0.37109337
Clostridium species incertae sedis [ext_mOTU_v26_18961]	-1.1877049	1.15726883	3.4445357	-0.218705	1.77065603	2.89725958	-0.6321911	0.37385834	0.46555944	0.08061281	0.09720109	0.25	0.85638273	0.00146484	0.0625
Clostridium species incertae sedis [ext_mOTU_v26_18974]	-0.8921109	-0.7688492	0.83584664	0.54381771	0.70663315	2.0870298	-1.4365423	0.0033243	0.01364152	0.0289917	0.6953125	0.25	0.06653595	0.02685547	0.03125
Clostridium species incertae sedis [ext_mOTU_v26_18980]	-0.3853452	0.76914264	-0.4370821	0.25442556	0.08551336	0	-0.1483922	0.71899035	0.78826184	0.44120852	0.17752985	1	0.41849223	1	NA
Clostridium species incertae sedis [ext_mOTU_v26_18994]	-1.1270076	-0.3480003	0.94146829	0.93059409	1.59482938	2.66729347	-1.9740401	2.14E-05	0.00039239	0.00268555	1	0.5	0.01576792	0.02099609	0.03125
Clostridium species incertae sedis [ext_mOTU_v26_19010]	-1.2223563	0.57939891	0.69599965	0.78950763	1.72198969	2.10946778	-1.6176706	0.00119606	0.00589106	0.03231291	0.27189871	0.75	0.02307267	0.00146484	0.03125
Clostridium species incertae sedis [ext_mOTU_v26_23317]	-0.0458907	0.89695242	-0.6787484	-0.3766833	-0.5143649	0	0.58285373	0.11326316	0.19467105	0.62406453	0.28071267	1	0.17752985	0.18144921	NA

Clostridium species incertae sedis [ext_mOTU_v26_23345]	0	0	0	-0.6868105	-1.6279928	-2.97004	1.29528846	0.02879885	0.0655422	NA	NA	NA	0.44687282	0.2084128	0.10034825
Clostridium species incertae sedis [ext_mOTU_v26_23387]	-0.1071099	0.46703594	-0.4589882	0.05977186	-0.2278873	0	0.09552457	0.7693539	0.83043207	1	0.14221324	1	0.44687282	0.42267807	NA
Clostridium species incertae sedis [ext_mOTU_v26_27738]	-0.7079609	-0.7804359	-0.1966429	-0.354173	0.39824422	-0.5834702	-0.533389	0.30996624	0.40396817	0.41011699	0.67498671	1	0.6834809	0.15078557	1
Clostridium species incertae sedis [ext_mOTU_v26_27794]	0.70228959	0.46882139	0	0.03162663	-0.7107148	0	0.74945667	0.01636655	0.04252725	0.18144921	1	NA	0.42267807	0.37109337	NA
Clostridium species incertae sedis [ext_mOTU_v26_27800]	0.03967446	0.30289725	-2.6218115	0.42727693	1.06748038	1.81665775	-1.0129743	0.09045419	0.16521754	0.6701287	0.55664063	0.25	0.1873117	0.05592968	0.03125
Clostridium species incertae sedis [ext_mOTU_v26_27810]	-1.2008557	-1.6578781	-1.4100341	0.15910861	-0.1511085	0	-1.4248639	0.00040983	0.00250449	0.02249427	0.58963855	1	0.58963855	0.78926803	NA
Clostridium species incertae sedis [ext_mOTU_v26_33441]	-0.4802708	0.64083044	0.11483366	0.01454789	0.03007239	0	-0.0279648	0.9182316	0.93235824	0.20489389	0.05905823	1	0.78740649	1	NA
Clostridium species incertae sedis [ext_mOTU_v26_33448]	1.07311887	1.58649578	0	-0.0756433	0.05867081	0	1.14509515	0.02341282	0.05681052	0.03603169	0.17752985	NA	0.58388242	1	NA
Clostridium species incertae sedis [ext_mOTU_v26_33449]	0.34977572	-0.7723519	-2.5745063	0.03086501	-0.707182	-0.8657461	-0.0772074	0.86977523	0.89151318	0.78740649	1	1	1	1	1
Clostridium species incertae sedis [ext_mOTU_v26_33463]	-0.5581016	0.14302574	1.12000405	-0.1524321	0	0	-0.0216517	0.96029732	0.96029732	0.18144921	1	1	0.37109337	NA	NA
Cutibacterium acnes [ref_mOTU_v25_00800]	-0.2655717	-0.7142897	-1.5032042	-0.3590623	-0.7002428	1.28369038	-0.3210157	0.31215722	0.40396817	0.85513214	0.18144921	0.37109337	0.17752985	0.10034825	0.10034825
Desulfovibrio species incertae sedis [ext_mOTU_v26_18866]	-0.3857835	-1.6697221	-1.972886	0.58804152	0.75941109	2.66395726	-1.9519528	0.00020094	0.00156006	0.32250977	0.10546875	0.25	0.09873962	0.06396484	0.03125
Desulfovibrionaceae species incertae sedis [ext_mOTU_v26_18835]	-0.2846768	-0.5475354	-1.3404809	0.6827397	0.54155817	0	-1.0539674	0.00171312	0.00796239	0.18343062	0.41849223	1	0.10564543	0.18144921	NA
Dorea sp. 5-2 [ref_mOTU_v25_07503]	-1.0425854	-0.7822903	1.0866436	0.34109279	0.99834662	3.41049549	-1.6491385	0.00295634	0.01250761	0.02019367	0.625	0.5	0.48513443	0.09228516	0.03125
Eggerthellaceae species incertae sedis [ext_mOTU_v26_15443]	0.02619335	-0.2556422	0.78494431	-0.1425037	0.48928138	0.73502438	-0.1605415	0.52298367	0.59981302	1	1	1	1	0.37109337	0.37109337
Eggerthellaceae species incertae sedis [ext_mOTU_v26_18906]	0.94070027	0.47152089	1.07292128	0.65719307	0.96736007	0.01897721	0.11564669	0.86001157	0.88688693	0.61029866	1	1	0.23002425	0.10831938	1
Eggerthellaceae species incertae sedis [ext_mOTU_v26_18907]	-0.7465125	-1.0001194	-1.6745793	0.05393231	-0.2255869	-0.6148762	-0.8245315	0.3110777	0.40396817	0.23120117	0.4921875	0.5	0.33876302	0.50487987	0.6875
Eggerthellaceae species incertae sedis [ext_mOTU_v26_27735]	1.31071695	0.60692548	3.06716254	0.92317187	1.62147038	2.90675264	-0.1359379	0.77829179	0.83440343	0.00335693	0.6953125	0.25	0.03036499	0.00683594	0.03125
Enterococcus faecalis [ref_mOTU_v25_00318]	7.38931207	2.74442142	2.65670312	0.22919493	0.4393102	2.47060143	4.57156694	8.00E-06	0.00022009	0.00166169	0.02249427	1	0.85513214	0.85513214	0.10034825
Enterorhabdus caecimuris [ref_mOTU_v25_02234]	2.10740422	1.04102345	3.81562976	1.56243757	2.43631529	4.4820525	-0.293361	0.59303375	0.67020937	6.10E-05	0.4921875	0.25	0.02081299	0.01220703	0.03125
Enterorhabdus mucosicola [ref_mOTU_v25_02233]	2.12816469	1.30026149	4.92650777	0.94080116	1.76406137	3.44129295	0.63875971	0.1879286	0.28447908	0.00015259	0.08398438	0.25	0.08976746	0.05224609	0.03125
Erysipelotrichaceae species incertae sedis [ext_mOTU_v26_15367]	-0.4659121	0.06937171	-1.9467782	0.80880988	0.51566737	-1.7293105	-0.8371669	0.19021901	0.28663138	0.10034825	1	1	0.50487987	0.52936811	0.85513214
Erysipelotrichaceae species incertae sedis [ext_mOTU_v26_18913]	-0.908124	-1.4105089	1.14761499	-0.1729414	-0.1395199	1.36146174	-0.8736399	0.06654502	0.13228829	0.10000062	0.05917207	0.37109337	0.67260382	0.78926803	0.37109337
Erysipelotrichaceae species incertae sedis [ext_mOTU_v26_19009]	0.61058989	-3.3239491	-2.7152449	-0.7894912	-1.2527654	-1.0902564	-0.2097614	0.79244395	0.84356936	0.48985385	0.05802402	1	0.48985385	0.41482307	0.6875
Erysipelotrichaceae species incertae sedis [ext_mOTU_v26_33494]	-0.1461817	-0.5857618	-2.548985	0.329143	-0.4662076	0.34046472	-0.6662217	0.27203283	0.37249308	0.81270369	0.67498671	0.5	0.90569544	0.58388242	0.42267807

Escherichia coli [ref_mOTU_v25_00095]	6.2004636	3.58739006	5.92613876	1.08511322	1.31459288	3.46474823	3.768404	0.00109377	0.00555298	0.00592154	0.02249427	0.37109337	0.29362154	0.2084128	0.05905823
Eubacterium sp. 14-2 [ref_mOTU_v25_07499]	-1.5310935	-0.3683508	0.47111526	0.82961249	1.60446104	4.67212089	-2.4621827	7.75E-05	0.00087095	0.03603169	0.79984611	0.5	0.07592696	0.03603169	0.03125
Eubacterium species incertae sedis [ext_mOTU_v26_18752]	-2.1943948	-2.1447422	1.07281454	0.79202033	1.63508754	3.00280499	-3.1351677	0.00036256	0.00239293	0.00669228	0.02439024	0.37109337	0.03603169	0.30806323	0.21875
Firmicutes bacterium ASF500 [ref_mOTU_v25_08057]	-0.2957476	-0.1402033	-0.397793	-0.1109341	0.46485305	2.43358244	-0.6652089	0.25639023	0.35656513	0.55411313	0.78926803	1	0.62406453	0.52936811	0.3125
Firmicutes species incertae sedis [ext_mOTU_v26_18791]	-0.4181727	-1.6371281	-0.8684695	0.29235554	-0.3225016	0	-0.9574667	0.02600441	0.06207465	0.20489389	0.05905823	1	0.58388242	0.42267807	NA
Firmicutes species incertae sedis [ext_mOTU_v26_19006]	-0.1936805	-0.7375302	0.09551012	0.16309566	-0.196941	0	-0.374487	0.11936408	0.20200076	1	0.58963855	1	0.78740649	0.78926803	NA
Firmicutes species incertae sedis [ext_mOTU_v26_23322]	-0.0609302	0.22955314	0.9715279	0.18866003	0.11784347	0.7124148	-0.0657222	0.84762322	0.87685161	1	1	1	0.58388242	1	1
Firmicutes species incertae sedis [ext_mOTU_v26_23325]	0.11905456	-0.2468082	-1.4227736	0.05086512	-0.2271205	0	-0.1525452	0.67188833	0.74403741	0.44120852	0.28071267	1	1	0.78926803	NA
Firmicutes species incertae sedis [ext_mOTU_v26_23336]	-0.6044777	-0.7676969	-2.3148432	-0.6890288	-1.6733452	-1.168077	0.20277489	0.73545536	0.80364327	0.44687282	0.58963855	0.37109337	0.2084128	0.05905823	0.36131043
Firmicutes species incertae sedis [ext_mOTU_v26_27776]	-0.2670881	-0.1869844	0	-1.0582873	-0.800366	0	0.62874681	0.12729313	0.21323215	0.78926803	1	NA	0.03603169	0.37109337	NA
Firmicutes species incertae sedis [ext_mOTU_v26_27784]	-0.194459	-2.3954874	-1.2237349	0.27492109	-0.0926937	0	-1.2184921	0.00875355	0.02873773	0.36272651	0.05905823	1	0.78740649	0.78926803	NA
Firmicutes species incertae sedis [ext_mOTU_v26_33447]	-0.7037697	-1.5643985	1.48037691	0.03152007	-0.4339669	0	-0.6156479	0.20150295	0.29685703	0.17752985	0.17752985	1	1	1	NA
Hungateiclostridiaceae bacterium KB18 [ref_mOTU_v25_10098]	-0.0601849	2.14641003	2.02090747	0.14787011	0.49795785	1.79216878	0.49784744	0.35566175	0.44914858	0.66854858	0.04882813	0.25	0.18146515	0.26611328	0.03125
Lachnoclostridium species incertae sedis [ext_mOTU_v26_18770]	-0.8695387	0.29392692	-0.0288392	0.83745689	1.51491288	2.98232575	-1.6956858	0.00045128	0.00270765	0.00265508	0.23242188	1	0.01383259	0.01220703	0.03125
Lachnoclostridium species incertae sedis [ext_mOTU_v26_18786]	-1.0536006	-0.0590899	-0.1357374	0.36932801	0.80709477	0.87016933	-1.167494	0.01225934	0.03494356	0.09344482	0.921875	1	0.26625059	0.16816683	0.15625
Lachnoclostridium species incertae sedis [ext_mOTU_v26_18936]	-0.6724813	0.13077866	0.03868019	0.16586489	-0.6212589	0	-0.1867871	0.61204458	0.68466004	0.09349248	0.58388242	1	0.28071267	0.42267807	NA
Lachnoclostridium species incertae sedis [ext_mOTU_v26_18985]	-1.8699254	-0.43491	0.74629575	0.95545831	-0.7915783	-1.8853009	-1.0505009	0.08874964	0.16361665	0.14221324	0.63558612	0.75	0.03027641	0.55411313	0.18144921
Lachnoclostridium species incertae sedis [ext_mOTU_v26_18998]	-2.3554007	-1.0348531	0.98577253	0.32230803	0.78920434	2.67344643	-2.2623331	2.62E-05	0.00041214	0.00209764	0.10546875	0.75	0.39355135	0.1535764	0.03125
Lachnoclostridium species incertae sedis [ext_mOTU_v26_18999]	-0.9247744	-0.3551129	1.68546415	0.23270686	0.69583022	1.58795003	-0.9656252	0.03956594	0.08762927	0.14385986	0.6953125	0.5	0.17059877	0.06396484	0.09375
Lachnoclostridium species incertae sedis [ext_mOTU_v26_19003]	-0.5519114	0.62855719	0.16843029	1.42088629	0.17649968	-1.2321999	-0.7099133	0.22343495	0.31919279	0.52816772	0.10546875	1	0.00336456	0.26611328	0.6875
Lachnoclostridium species incertae sedis [ext_mOTU_v26_27798]	-0.8688681	0.50224093	1.3126889	0.85880274	0.65873341	1.83881493	-1.0377446	0.05099266	0.10718202	0.05905823	0.58388242	0.75	0.04401098	0.40694122	0.20124262
Lachnoclostridium species incertae sedis [ext_mOTU_v26_27803]	-1.8181103	-1.0323075	-0.4998367	0.49858649	0.93386696	3.15584161	-2.3685053	4.45E-05	0.00058573	0.00021362	0.921875	1	0.15556599	0.42382813	0.03125
Lachnospiraceae bacterium 10-1 [ref_mOTU_v25_07505]	-0.006039	0.77964825	3.6996826	0.38282075	0.10473071	-1.5271291	0.68565692	0.18183852	0.27707499	1	1	1	0.10034825	0.78926803	0.37109337
Lachnospiraceae bacterium 28-4 [ref_mOTU_v25_11106]	-0.4447485	1.57012365	-0.863991	1.39468443	0.92294438	1.24340885	-1.0026013	0.18161943	0.27707499	0.72676781	0.05802402	0.5	0.0045073	0.17626953	0.15625

Lachnospiraceae bacterium 3-2 [ref_mOTU_v25_07504]	-1.1214433	-0.1948659	-0.0503562	-0.3723624	-0.020464	1.40986645	-0.6390622	0.17526189	0.27281332	0.07592696	1	1	0.55411313	0.72628615	0.36131043
Lachnospiraceae bacterium A2 [ref_mOTU_v25_11108]	-1.6957296	-3.2942168	-3.2408231	-0.0325158	0.18894801	1.54914926	-2.6991193	0.00696403	0.02471109	0.10551372	0.19251757	0.75	0.9000858	0.94418251	0.84375
Lachnospiraceae bacterium A4 [ref_mOTU_v25_11109]	-1.0592515	-0.2432379	-0.0509192	0.05645954	0.03979434	0	-0.6902115	0.0140853	0.03873457	0.05905823	1	1	0.42267807	1	NA
Lachnospiraceae bacterium COE1 [ref_mOTU_v25_07500]	-0.6544817	0.88836954	-0.3526502	0.31449024	0.03009923	0	-0.2509456	0.50710293	0.59553013	0.14148212	0.10564543	1	0.41849223	1	NA
Lachnospiraceae bacterium M18-1 [ref_mOTU_v25_03466]	0.15907941	1.6860772	4.45064814	1.06432713	1.83593296	1.34377488	-0.1280896	0.82716096	0.86197898	0.2084128	0.23613697	0.25	0.18514372	0.10732756	0.15625
Lachnospiraceae species incertae sedis [ext_mOTU_v26_17313]	-0.7981748	-0.4427596	0	0.43661931	0.73081748	0	-1.0502	0.02614659	0.06207465	0.37109337	1	NA	0.18144921	0.37109337	NA
Lachnospiraceae species incertae sedis [ext_mOTU_v26_18732]	-2.0294772	-0.8746454	-0.7435582	1.32345524	-0.216254	4.54242613	-2.7041748	0.00035364	0.00238168	0.01578549	0.81270369	0.25	0.04316711	0.5633178	0.0625
Lachnospiraceae species incertae sedis [ext_mOTU_v26_18735]	-0.7651333	0.54080458	-0.2547464	0.19953229	0.08969816	0	-0.3773266	0.32447166	0.41663676	0.23395283	0.28071267	1	0.58963855	0.78926803	NA
Lachnospiraceae species incertae sedis [ext_mOTU_v26_18736]	-0.3809247	0.36442637	0.07986361	1.50028448	2.30856394	3.78336767	-2.1256089	0.0011346	0.005673	0.81270369	0.14221324	1	0.01448328	0.02997397	0.10034825
Lachnospiraceae species incertae sedis [ext_mOTU_v26_18739]	-0.4772265	-0.1803562	-0.225523	-0.0400617	-0.0157651	0.56619436	-0.388752	0.29930279	0.39967664	0.28071267	0.58388242	1	1	1	1
Lachnospiraceae species incertae sedis [ext_mOTU_v26_18751]	-3.4688526	-5.7281205	-2.457212	-0.5175299	0.07901821	3.15722237	-4.3057886	8.26E-07	6.10E-05	0.00237684	0.00195313	0.5	0.14056241	0.23002425	0.0625
Lachnospiraceae species incertae sedis [ext_mOTU_v26_18758]	-0.3705671	0.98916931	1.49604655	1.41645047	1.44652978	2.13859026	-1.1813189	0.04126396	0.08966792	1	0.04882813	0.5	0.00104523	0.03417969	0.03125
Lachnospiraceae species incertae sedis [ext_mOTU_v26_18762]	-2.0528512	-0.9976058	1.47024381	0.01702048	-1.1450695	1.92344648	-1.1336216	0.14859792	0.24211323	0.00258448	0.6953125	0.25	0.51365564	0.75976583	0.4375
Lachnospiraceae species incertae sedis [ext_mOTU_v26_18766]	-0.6433385	-0.1712304	1.74599811	0.70088453	-1.4327405	-1.948878	0.16346688	0.80846678	0.8498855	0.08005792	0.78740649	0.37109337	0.03603169	1	0.09375
Lachnospiraceae species incertae sedis [ext_mOTU_v26_18767]	-1.0992376	0.16054691	0.34566376	0.30430388	0.36391398	1.85640658	-1.0036195	0.09463932	0.16973357	0.12353425	0.15078557	0.75	0.32787693	0.2863206	0.10034825
Lachnospiraceae species incertae sedis [ext_mOTU_v26_18771]	-0.8088115	0.27163577	-0.4924411	0.6210895	1.85039174	3.01639139	-1.7261012	0.00606563	0.02199623	0.34838867	0.19335938	0.75	0.05993652	0.01220703	0.03125
Lachnospiraceae species incertae sedis [ext_mOTU_v26_18772]	-0.3146324	-0.109806	-1.7114637	1.01219269	1.5066099	1.91865016	-1.7066485	0.00131169	0.0061837	0.28071267	1	1	0.04544695	0.01781719	0.09375
Lachnospiraceae species incertae sedis [ext_mOTU_v26_18773]	-1.4382572	-1.3694817	-1.889593	0.56038151	1.33083112	2.39008556	-2.522529	1.73E-06	7.46E-05	0.03863525	0.13085938	0.25	0.34654999	0.04248047	0.03125
Lachnospiraceae species incertae sedis [ext_mOTU_v26_18782]	-1.4099574	-1.5620403	-1.8701174	0.79551757	0.67303658	3.4504395	-2.6242645	1.29E-05	0.00032711	0.00515747	0.08398438	0.5	0.0538559	0.09228516	0.03125
Lachnospiraceae species incertae sedis [ext_mOTU_v26_18784]	-1.1971508	1.02076058	0.89454379	0.72832243	1.46629208	5.97951161	-1.8151573	0.00303671	0.01268498	0.34637328	0.10546875	0.75	0.18501631	0.56933594	0.03125
Lachnospiraceae species incertae sedis [ext_mOTU_v26_18787]	-1.1000432	0.61493617	2.92927434	0.05534105	-0.2667765	1.36632859	-0.1206339	0.80867893	0.8498855	0.00196553	0.10546875	0.25	0.10838318	1	0.03125
Lachnospiraceae species incertae sedis [ext_mOTU_v26_18788]	0.03159183	2.29582966	5.21396812	1.65970925	2.38664582	4.56693998	-0.8111169	0.30374979	0.39967664	0.55411313	0.10034825	0.37109337	0.25534036	0.14148212	0.18144921
Lachnospiraceae species incertae sedis [ext_mOTU_v26_18793]	-2.8285976	-5.3752847	-4.3303376	-0.3972387	-0.0905711	0.06470998	-3.6786835	1.76E-05	0.00036292	0.00042725	0.00390625	0.25	0.88706869	0.23339844	0.15625
Lachnospiraceae species incertae sedis [ext_mOTU_v26_18796]	-2.5500932	-3.4305863	-2.2804755	-0.7414969	1.12048266	2.38944467	-3.1091593	0.00390976	0.01554482	0.00680165	0.52861213	0.37109337	0.29338289	0.05224609	0.15625

Lachnospiraceae species incertae sedis [ext_mOTU_v26_18797]	-0.7074618	-0.8238052	0.14852313	-0.4169851	-0.8862076	0	-0.1276775	0.75846369	0.82333229	0.2084128	0.42267807	1	0.58388242	0.18144921	NA
Lachnospiraceae species incertae sedis [ext_mOTU_v26_18802]	-4.5741097	-6.6334499	-7.7296086	-0.7008857	0.39783503	2.36759065	-5.7526659	1.38E-09	4.55E-07	0.00072651	0.00195313	0.25	0.37947136	0.10291754	0.4375
Lachnospiraceae species incertae sedis [ext_mOTU_v26_18803]	-2.2364413	-3.0690448	-1.0477689	1.06291803	1.39220095	0.74582487	-3.5149009	9.33E-09	1.54E-06	0.00166169	0.01367188	1	0.01578549	0.03667129	0.78740649
Lachnospiraceae species incertae sedis [ext_mOTU_v26_18811]	-1.2248195	-0.2358023	-0.7255439	0.39984374	0.77054795	2.02954826	-1.5487483	0.0049934	0.01938615	0.11655196	0.47719662	1	0.70642712	0.44980379	0.17752985
Lachnospiraceae species incertae sedis [ext_mOTU_v26_18812]	-3.3262635	-4.0874147	-3.9018005	-1.2139611	-0.6267915	0.56386378	-2.8784648	0.00015229	0.00122572	0.00258448	0.01426619	0.25	0.69815238	0.89390372	0.6875
Lachnospiraceae species incertae sedis [ext_mOTU_v26_18815]	-1.2787119	-1.2797242	-3.0223587	0.55880426	0.60157108	0.63816482	-2.0760343	0.00124338	0.00594661	0.14385986	0.6953125	1	0.32472992	0.03417969	0.6875
Lachnospiraceae species incertae sedis [ext_mOTU_v26_18822]	-1.6576224	-1.9195341	0.4817888	1.28959888	0.42802397	2.74749686	-2.6867327	7.06E-07	6.10E-05	6.10E-05	0.03710938	0.75	0.00193024	0.15136719	0.03125
Lachnospiraceae species incertae sedis [ext_mOTU_v26_18829]	-1.694208	-2.9894012	-0.16534	0.49492397	1.18549367	1.70816447	-2.8462763	1.76E-06	7.46E-05	0.00072651	0.00915169	1	0.14753242	0.12627893	0.10564543
Lachnospiraceae species incertae sedis [ext_mOTU_v26_18830]	-0.5629181	-0.8753188	-0.2243215	0.70489159	0.41221663	2.01322475	-1.4128228	0.00510841	0.01957097	0.01426619	1	1	0.02382415	0.15513157	0.0625
Lachnospiraceae species incertae sedis [ext_mOTU_v26_18832]	-1.4297685	-0.4859974	-0.4152808	0.29677758	-0.4267763	0	-0.9916913	0.06182144	0.12593256	0.05905823	0.37109337	1	0.67498671	0.78926803	NA
Lachnospiraceae species incertae sedis [ext_mOTU_v26_18836]	-1.4304695	0.0524165	0.01980242	0.34098462	0.11527047	0	-0.9496669	0.0294121	0.06647941	0.01082692	0.78740649	1	0.52936811	0.42267807	NA
Lachnospiraceae species incertae sedis [ext_mOTU_v26_18845]	0.16596786	1.32990454	1.12976893	0.46618504	-0.4562248	-1.7498745	0.82367974	0.07599653	0.14496448	0.37109337	0.37109337	1	0.20124262	0.58388242	0.37109337
Lachnospiraceae species incertae sedis [ext_mOTU_v26_18848]	-1.2542007	0.77570709	-0.3500001	0.63946992	1.63355557	2.98332043	-1.6976395	0.00352529	0.01418716	0.1166687	0.06445313	0.75	0.01619648	0.01611328	0.0625
Lachnospiraceae species incertae sedis [ext_mOTU_v26_18850]	-0.7705353	0.56126466	0.85020348	0.27803235	0.76484339	2.32596437	-0.8067836	0.07309638	0.14106319	0.10732756	0.55411313	1	0.20489389	0.79984611	0.10034825
Lachnospiraceae species incertae sedis [ext_mOTU_v26_18851]	-0.7358564	-1.6045171	-0.3086121	0.75121221	0.34564383	-0.001761	-1.5105056	0.00095392	0.00499673	0.29450739	0.10564543	1	0.10291754	1	1
Lachnospiraceae species incertae sedis [ext_mOTU_v26_18852]	-0.5072223	0.21356032	0.09185211	-0.4481435	-0.8348553	-3.9402512	0.85508457	0.15309899	0.24525566	0.78926803	0.42267807	1	0.32787693	0.72228296	0.21875
Lachnospiraceae species incertae sedis [ext_mOTU_v26_18854]	-1.2310453	-1.5311774	-0.465916	0.22463724	1.52966099	3.20618024	-2.2877028	8.96E-05	0.00089569	0.12973022	0.04882813	0.25	0.05993652	0.00488281	0.03125
Lachnospiraceae species incertae sedis [ext_mOTU_v26_18856]	-0.8093472	-1.500427	-0.548501	0.39441102	0.38644913	0.70793691	-1.4551333	0.00776137	0.02640466	0.08296041	0.62406453	1	0.06654572	0.35254214	1
Lachnospiraceae species incertae sedis [ext_mOTU_v26_18872]	-2.1841043	-0.9512164	-0.7257288	0.4640131	1.53182973	1.88931855	-2.5700064	5.31E-05	0.00062543	0.04377232	0.375	0.75	0.12973785	0.03417969	0.03125
Lachnospiraceae species incertae sedis [ext_mOTU_v26_18875]	-0.6480728	0.87830298	2.16108688	0.49539476	0.18574361	-0.4099113	-0.0380838	0.94489895	0.95168837	0.09383864	0.04882813	0.5	0.19638824	0.73339844	0.84375
Lachnospiraceae species incertae sedis [ext_mOTU_v26_18881]	-0.8224859	-0.2385736	-1.4882634	0.43005551	1.46694738	2.456071	-1.7334022	0.01010294	0.03115861	0.28958742	0.47719662	0.5	0.0787915	0.03231291	0.15625
Lachnospiraceae species incertae sedis [ext_mOTU_v26_18886]	-0.2970526	0.54818616	3.8255031	-0.0053112	1.3644465	2.36598675	-0.2476419	0.64301326	0.71445918	0.78926803	0.37109337	0.37109337	0.94418251	0.07592696	0.15625
Lachnospiraceae species incertae sedis [ext_mOTU_v26_18889]	0.52914769	0.58570203	1.18256023	0.85253727	0.98498968	3.54556842	-0.6203896	0.28758545	0.38578536	0.55411313	0.58963855	1	0.02109797	0.10732756	0.03125
Lachnospiraceae species incertae sedis [ext_mOTU_v26_18891]	-0.3757413	-1.5288763	-1.6030162	0.62705348	0.9380009	1.99188336	-1.8428661	0.01453384	0.03940428	1	0.20489389	0.5	0.06835303	0.07556057	0.09375

Lachnospiraceae species incertae sedis [ext_mOTU_v26_18894]	-0.9686233	-1.3570388	-1.3625733	-0.1302643	0.96979392	4.22136999	-1.9564466	0.00067105	0.00375333	0.25222778	0.27539063	0.25	0.69815238	0.14236818	0.03125
Lachnospiraceae species incertae sedis [ext_mOTU_v26_18896]	-2.0440188	-1.4899327	-0.9974437	0.17913647	-0.1392312	1.19392617	-1.9275074	0.00521892	0.01957097	0.01285124	0.40167817	1	0.83393541	0.58963855	0.37109337
Lachnospiraceae species incertae sedis [ext_mOTU_v26_18898]	-1.1963953	-1.8859308	-2.8803448	0.86800477	0.93822328	3.30980943	-2.8587474	0.000119	0.00109086	0.05164622	0.02439024	0.37109337	0.08323017	0.04544695	0.09375
Lachnospiraceae species incertae sedis [ext_mOTU_v26_18902]	-0.0710375	-0.9303797	0.00363737	-0.3559668	-1.4488731	-1.3988196	0.4850272	0.27609299	0.37568227	0.19251757	0.36131043	1	0.41482307	0.29450739	0.37109337
Lachnospiraceae species incertae sedis [ext_mOTU_v26_18919]	-1.0549109	-0.5342409	0.84061385	-0.3071149	-1.6277803	1.41914922	-0.1247208	0.80064925	0.84956351	0.23120117	1	1	0.79870605	0.04248047	0.03125
Lachnospiraceae species incertae sedis [ext_mOTU_v26_18920]	-0.1973579	0.0188592	0	0.30651365	0.21228539	0	-0.331945	0.06456065	0.12912131	0.42267807	1	NA	0.37109337	1	NA
Lachnospiraceae species incertae sedis [ext_mOTU_v26_18934]	-0.8020734	-1.0213598	0.22006167	-0.6494708	-2.1758813	-4.5275666	0.90484007	0.08558514	0.15975317	0.14148212	0.58963855	1	0.94428467	0.00915169	0.03125
Lachnospiraceae species incertae sedis [ext_mOTU_v26_18941]	-3.0085095	-2.3535114	1.83469635	0.3366744	-0.0635038	2.51524205	-2.6744697	1.59E-05	0.00036292	0.00072651	0.07556057	0.25	0.34376	1	0.0625
Lachnospiraceae species incertae sedis [ext_mOTU_v26_18949]	-0.9670971	2.63838074	2.48078	1.70875524	2.10859463	5.59145537	-1.6172359	0.01984978	0.05077852	0.02938254	0.04401098	0.25	0.01149007	0.04149109	0.05905823
Lachnospiraceae species incertae sedis [ext_mOTU_v26_18956]	-1.8225331	-3.3823811	-3.7654427	0.53921267	1.01572226	0.55635167	-3.3108772	7.11E-06	0.0002132	6.10E-05	0.625	1	0.10750801	0.03667129	0.28071267
Lachnospiraceae species incertae sedis [ext_mOTU_v26_18957]	-0.2892401	0.19916213	1.20643183	1.15881472	1.75874984	3.52507568	-1.5992315	0.02725352	0.06328108	0.67498671	0.23242188	0.5	0.03027641	0.02337639	0.03125
Lachnospiraceae species incertae sedis [ext_mOTU_v26_18958]	-1.4061066	-2.752725	-1.8464705	0.52173021	1.5064801	2.1060188	-2.9897513	2.04E-06	7.46E-05	0.00697954	0.04401098	0.75	0.39273834	0.07714844	0.03125
Lachnospiraceae species incertae sedis [ext_mOTU_v26_18959]	-1.7206025	-1.6808636	0.57944148	-0.3956074	1.10285519	2.88370442	-1.9510387	0.0001282	0.00114345	0.01098633	0.02734375	0.5	0.89857483	0.07714844	0.03125
Lachnospiraceae species incertae sedis [ext_mOTU_v26_18960]	-2.3199272	-0.5980797	1.97733277	-0.1810472	-0.0462091	2.6517	-1.41855	0.01456764	0.03940428	0.02486786	0.921875	0.25	0.51804649	0.75565774	0.03125
Lachnospiraceae species incertae sedis [ext_mOTU_v26_18962]	-1.0266943	0.2544914	-1.0694652	0.48952749	0.42910175	1.40539432	-1.16814	0.09846102	0.1746889	0.18740119	0.32226563	0.75	0.48985385	0.50487987	0.17752985
Lachnospiraceae species incertae sedis [ext_mOTU_v26_18965]	-1.9430978	-1.891198	-0.2141701	0.18157231	-0.573786	0.58411328	-1.6995649	0.00517694	0.01957097	0.00286559	0.13085938	0.5	0.26452637	0.79101563	0.6875
Lachnospiraceae species incertae sedis [ext_mOTU_v26_18968]	-0.3123326	-0.0216628	-1.154476	-0.0115272	-0.2648535	0	-0.2195181	0.40550595	0.49931703	0.18144921	1	1	0.78926803	1	NA
Lachnospiraceae species incertae sedis [ext_mOTU_v26_18969]	-0.6904441	-0.7007474	-1.2019908	1.82254277	2.63398229	5.37472788	-3.3119282	2.32E-05	0.00040271	0.61029866	0.40167817	1	0.00317266	0.01781719	0.0625
Lachnospiraceae species incertae sedis [ext_mOTU_v26_18971]	-1.0993896	-0.2550866	3.3773055	-0.0046854	0.40463412	1.21493725	-0.5413503	0.19562582	0.29211096	0.01426619	0.29450739	0.25	1	0.10564543	0.37109337
Lachnospiraceae species incertae sedis [ext_mOTU_v26_18978]	-1.3045464	-1.5699593	0.39197741	-0.1820056	1.04058273	2.15920282	-1.7169769	0.00595549	0.02183679	0.23481129	0.13085938	0.5	0.77610522	0.01846609	0.15625
Lachnospiraceae species incertae sedis [ext_mOTU_v26_18984]	-1.4433235	0.08376447	-0.200598	0.32418086	0.36530542	1.36931853	-1.2250405	0.00944104	0.02967185	0.02800782	0.375	1	0.14224517	0.04248047	0.03125
Lachnospiraceae species incertae sedis [ext_mOTU_v26_18997]	-1.6312959	-0.5902082	0.78426917	0.42389143	0.77606131	0.86418155	-1.5636667	0.0092285	0.02928472	0.00418091	0.625	0.25	0.12981814	0.42382813	0.21875
Lachnospiraceae species incertae sedis [ext_mOTU_v26_19000]	-0.9956341	-0.8384626	0.70298978	0.79974961	-0.2496932	-1.9513994	-0.826088	0.15795243	0.25180822	0.05191296	0.52936811	1	0.04455332	0.36272651	0.17752985
Lachnospiraceae species incertae sedis [ext_mOTU_v26_19001]	-1.215382	0.1960624	-0.2787545	0.30359365	-0.3690455	-0.8809081	-0.5281204	0.28170166	0.37943489	0.05802402	1	0.75	0.11655196	0.94418251	0.84375

Lachnospiraceae species incertae sedis [ext_mOTU_v26_19002]	-0.8470422	-0.9982013	-1.4194333	0.15124607	0	0	-1.052528	0.01083011	0.03219763	0.2084128	0.41849223	1	1	NA	NA
Lachnospiraceae species incertae sedis [ext_mOTU_v26_19004]	-1.0586893	-0.4562352	1.6449303	0.29529144	0.94169403	3.12053666	-1.3922228	0.03002272	0.06739794	0.03667129	0.67260382	0.37109337	0.44880444	0.09228516	0.03125
Lachnospiraceae species incertae sedis [ext_mOTU_v26_23286]	-0.6128795	-1.7827591	-1.4563849	1.09527282	1.59337695	1.64830606	-2.4618685	0.00029434	0.0020236	0.72676781	0.04231527	0.37109337	0.0787915	0.11972241	0.0625
Lachnospiraceae species incertae sedis [ext_mOTU_v26_23287]	-1.6313217	-2.1079178	-1.2423093	0.56090053	0.74780707	2.01330899	-2.5653896	2.56E-05	0.00041214	0.08061281	0.05802402	1	0.20928655	0.15513157	0.10034825
Lachnospiraceae species incertae sedis [ext_mOTU_v26_23289]	-0.4393689	0.33643273	-0.7780063	0.41318346	0.46596368	0	-0.5823109	0.26671494	0.36673304	0.78740649	0.58388242	1	0.85513214	0.78926803	NA
Lachnospiraceae species incertae sedis [ext_mOTU_v26_23292]	-1.2565121	1.39157289	0.55189821	0.70124484	1.68769954	1.56276639	-1.2347584	0.07024615	0.13716705	0.06868016	0.13085938	1	0.14056241	0.00488281	0.09375
Lachnospiraceae species incertae sedis [ext_mOTU_v26_23295]	-0.7785733	-1.3838432	1.8254908	0.12598062	0.47454205	0.31140016	-0.9384121	0.16318088	0.25765401	0.14221324	0.55361699	0.75	0.42285039	0.14236818	0.78926803
Lachnospiraceae species incertae sedis [ext_mOTU_v26_23302]	-1.1151276	-0.0895651	0	0.17515282	-0.1373589	0	-0.6648733	0.03643209	0.08123372	0.05917207	1	NA	0.36131043	0.18144921	NA
Lachnospiraceae species incertae sedis [ext_mOTU_v26_23332]	-2.3238208	-1.7424281	0.56863426	0.37418361	0.98137041	2.84293966	-2.6608833	0.00022913	0.0016803	0.02139282	0.4921875	0.5	0.11870575	0.07714844	0.0625
Lachnospiraceae species incertae sedis [ext_mOTU_v26_23338]	-0.6649178	-0.7517705	0.03058793	0.42182373	1.19058278	1.75473189	-1.4593895	0.00028622	0.00200965	0.11972241	0.14221324	1	0.26452637	0.02099609	0.05905823
Lachnospiraceae species incertae sedis [ext_mOTU_v26_23339]	-0.2990006	0	0	0.28992702	-0.5776968	0.64343823	-0.2083105	0.50412974	0.59415291	0.37109337	NA	NA	0.18144921	1	1
Lachnospiraceae species incertae sedis [ext_mOTU_v26_23341]	-1.3622582	-1.8127618	-2.5855014	0.06955255	0.92122817	0.51204981	-2.0786858	0.00084797	0.00458739	0.01187344	0.12353425	0.5	0.20120399	0.09228516	0.41849223
Lachnospiraceae species incertae sedis [ext_mOTU_v26_23351]	-2.5573632	-2.4107665	0.24748086	-0.4017512	0.87227313	3.2538742	-2.6577649	0.00013205	0.00114677	0.00021362	0.16015625	0.75	0.96611786	0.17626953	0.03125
Lachnospiraceae species incertae sedis [ext_mOTU_v26_23352]	-0.0179413	-0.4619147	-4.0069475	-0.3518291	0.21529751	2.70110195	-0.8992025	0.11781981	0.20041513	1	1	0.37109337	0.85513214	0.36131043	0.10034825
Lachnospiraceae species incertae sedis [ext_mOTU_v26_23364]	-0.7410605	-0.1894704	-1.2702565	0.01369341	-0.3143378	0	-0.5148839	0.07575698	0.14496448	0.35254214	1	1	1	1	NA
Lachnospiraceae species incertae sedis [ext_mOTU_v26_23370]	-0.9788731	0.80576168	0.54211809	0.83287681	-0.8557432	0.81935494	-0.4377393	0.46069382	0.54884101	0.03027641	0.10732756	1	0.14056241	0.40694122	0.58388242
Lachnospiraceae species incertae sedis [ext_mOTU_v26_23371]	-0.2282283	0.26777201	0.18392847	0.08477138	-0.2594653	-3.4648701	0.49226459	0.27663876	0.37568227	1	0.42267807	1	0.50487987	0.72628615	0.0625
Lachnospiraceae species incertae sedis [ext_mOTU_v26_27740]	-0.8356916	1.22029186	0.29422225	-0.0563906	0.02742773	0	0.05252777	0.90383213	0.92341982	0.09349248	0.20124262	1	0.42267807	0.78926803	NA
Lachnospiraceae species incertae sedis [ext_mOTU_v26_27756]	-0.1977223	0.06818062	0.85803644	1.14648156	1.06928496	2.00685774	-1.2083043	0.01518681	0.0402726	0.18343062	0.28071267	0.37109337	0.02019367	0.23613697	0.03125
Lachnospiraceae species incertae sedis [ext_mOTU_v26_27764]	-0.109944	1.69443545	0	0.26023036	0.34488477	0	0.28883077	0.51363355	0.59981302	0.78740649	0.10564543	NA	1	1	NA
Lachnospiraceae species incertae sedis [ext_mOTU_v26_27772]	-1.1796699	0.03463736	-0.005268	0.57309418	1.45096276	3.06818348	-1.794256	0.00055523	0.00327187	0.12973022	0.375	1	0.06653595	0.05224609	0.03125
Lachnospiraceae species incertae sedis [ext_mOTU_v26_27774]	-0.6891157	-0.1669213	0.30078584	1.18076063	1.73038893	3.61527096	-2.0635427	0.00231192	0.01003859	0.29450739	0.44120852	0.37109337	0.03315388	0.04149109	0.0625
Lachnospiraceae species incertae sedis [ext_mOTU_v26_27779]	-0.7607446	-0.7623495	-2.5668323	-0.3183041	-0.1241714	0.67743017	-0.8588057	0.10086771	0.17705502	0.18144921	0.78926803	1	1	1	1
Lachnospiraceae species incertae sedis [ext_mOTU_v26_27785]	-0.2812361	-0.35021	-0.3288323	0.6465003	0.87683235	3.98423731	-1.4718856	0.02252395	0.05542521	0.44980379	0.23395283	1	0.20489389	0.14221324	0.10034825

Lachnospiraceae species incertae sedis [ext_mOTU_v26_27795]	-1.177445	-0.0655885	0.91781806	0.77325725	1.31840753	3.19269131	-1.7965813	0.00180903	0.00829137	0.00697954	0.375	0.75	0.08834899	0.00341797	0.05905823
Lachnospiraceae species incertae sedis [ext_mOTU_v26_27796]	-1.1856133	-0.6817806	-0.2827133	0.88945754	1.64847399	2.74673155	-2.2787514	3.88E-05	0.0005339	0.13229866	0.08398438	0.5	0.08142853	0.01611328	0.03125
Lachnospiraceae species incertae sedis [ext_mOTU_v26_27805]	-0.8771789	0.31933697	2.33938739	0.30734014	1.31637316	2.42563107	-0.9755325	0.06984524	0.13716705	0.35254214	0.72628615	0.5	0.30806323	0.02086258	0.10034825
Lachnospiraceae species incertae sedis [ext_mOTU_v26_27812]	-0.3797006	0.61624721	-0.3021967	-0.2733582	-0.5346271	-3.1130959	0.71454593	0.17083476	0.26718232	1	0.37109337	1	0.72228296	0.94418251	0.10564543
Lachnospiraceae species incertae sedis [ext_mOTU_v26_27814]	-0.6542522	0.10333898	-0.1315788	0.22371487	0.52453814	0	-0.6148239	0.09122602	0.16540982	0.07592696	0.28071267	1	0.85513214	0.42267807	NA
Lachnospiraceae species incertae sedis [ext_mOTU_v26_27825]	-0.6835128	-1.4561294	0.68764603	0.43368491	0.31007958	2.18612514	-1.412425	0.01885223	0.04860341	0.03603169	0.14148212	1	0.83172607	0.38037109	0.09375
Lachnospiraceae species incertae sedis [ext_mOTU_v26_33451]	-1.5601694	-0.5243145	1.10353994	0.04306481	0.79090764	2.76251732	-1.5122253	0.01382828	0.03834732	0.02337639	0.36272651	1	0.32025538	0.10291754	0.03125
Lachnospiraceae species incertae sedis [ext_mOTU_v26_33495]	-0.4720428	0.80127741	-1.1907007	0.76192497	-0.7388366	-0.7340481	-0.1824733	0.7546575	0.82190421	0.44323108	0.04882813	0.5	0.10551372	0.56933594	1
Lactobacillus animalis/murinus [ref_mOTU_v25_03470]	1.27514337	1.73800503	4.80428915	-1.0546904	1.09186175	4.36551847	1.51062711	0.02195585	0.05488962	0.15905762	0.19335938	0.25	0.04316711	0.17626953	0.03125
Lactobacillus johnsonii [ref_mOTU_v25_01040]	-0.9862736	-1.2673196	3.32514078	-1.5761482	0.2540466	2.8438481	-0.1618483	0.82171549	0.8608448	0.19281006	0.63558612	0.25	0.00769043	0.33935547	0.09375
Lactobacillus reuteri [ref_mOTU_v25_04085]	-1.1014147	-0.3869152	2.92301404	-2.9382294	0.5560679	4.36487991	0.47823148	0.51968697	0.59981302	0.01309204	0.32226563	0.25	7.63E-05	0.30126953	0.03125
Lactobacillus taiwanensis [ref_mOTU_v25_01041]	-0.4976428	-2.2239051	3.87444304	-1.7924132	0.32520611	3.28610246	-0.1421432	0.86990074	0.89151318	0.49542236	1	0.25	0.00041962	0.46972656	0.0625
Muribaculaceae species incertae sedis [ext_mOTU_v26_18165]	1.27115148	-0.4702979	0.11069471	-0.0494405	-1.1845388	-3.2801408	1.35803569	0.0049853	0.01938615	0.00418091	0.625	1	0.93228149	0.09228516	0.03125
Muribaculaceae species incertae sedis [ext_mOTU_v26_18726]	0.95820196	0.62506522	-0.8475172	-0.1641956	-0.9480456	-1.405591	1.2031394	7.94E-05	0.00087095	0.00021362	0.19335938	1	0.79870605	0.15136719	0.03125
Muribaculaceae species incertae sedis [ext_mOTU_v26_18727]	-0.1213632	-0.2189514	4.65754514	-0.1276867	0.41723202	0.90874496	0.242698	0.52166739	0.59981302	0.59658813	0.375	0.25	0.21214294	0.56933594	0.15625
Muribaculaceae species incertae sedis [ext_mOTU_v26_18734]	0.08956417	-0.0507304	1.57371594	1.07518843	1.06595532	3.73271486	-1.1994055	0.01614676	0.04228915	0.89993286	0.43164063	0.25	0.01387024	0.00683594	0.03125
Muribaculaceae species incertae sedis [ext_mOTU_v26_18738]	-0.4363455	-0.2033783	0.20448135	-0.4439333	-0.5682754	-2.4297288	0.47022362	0.28159389	0.37943489	0.04431152	1	0.75	0.44229889	0.10986328	0.03125
Muribaculaceae species incertae sedis [ext_mOTU_v26_18747]	-0.7976355	0.73372864	-0.1178404	-2.1756413	-2.7761608	-4.6629274	2.5273099	1.82E-06	7.46E-05	0.03863525	0.16015625	1	0.00019073	0.00146484	0.03125
Muribaculaceae species incertae sedis [ext_mOTU_v26_18755]	-3.6992507	-4.6966558	-2.3642073	-2.6335882	-4.4645765	-3.8489879	-0.4953102	0.52347318	0.59981302	0.00021362	0.06445313	0.25	0.00053406	0.00341797	0.4375
Muribaculaceae species incertae sedis [ext_mOTU_v26_18776]	0.90122243	1.21791335	0.64901758	-0.0805402	-1.3658837	-2.9458827	1.86080142	0.00020801	0.00156006	0.00918579	0.00976563	0.75	0.46829224	0.38037109	0.03125
Muribaculaceae species incertae sedis [ext_mOTU_v26_18780]	0.91647655	-2.2950787	0.69383939	-0.0396401	0.04461565	2.59177198	-0.5819249	0.44893627	0.54267022	0.0289917	0.01367188	0.5	1	0.17626953	0.03125
Muribaculaceae species incertae sedis [ext_mOTU_v26_18783]	-0.7365781	-1.4820199	2.07492556	-0.9314121	-0.2700174	1.30769153	-0.2355444	0.48329426	0.57163837	0.00628662	0.00976563	0.25	0.00336456	0.56933594	0.09375
Muribaculaceae species incertae sedis [ext_mOTU_v26_18843]	0.54266185	1.13621435	1.2804004	0.28729059	-0.2097027	0	0.75672379	0.00629923	0.02259508	0.29362154	0.05905823	1	0.05905823	0.18144921	NA
Muribaculaceae species incertae sedis [ext_mOTU_v26_18915]	-0.7074713	-1.2509452	1.20490973	-0.409396	0.08387478	2.86877094	-0.848457	0.04474272	0.0952587	0.00268555	0.01953125	0.25	0.26452637	0.23339844	0.03125

Muribaculaceae species incertae sedis [ext_mOTU_v26_18917]	0.50810597	0.93108815	2.67088601	-0.4492246	-0.391633	-0.739725	1.39150485	3.01E-05	0.00044108	0.21142578	0.03710938	0.25	0.12973785	0.96972656	0.3125
Muribaculaceae species incertae sedis [ext_mOTU_v26_18981]	-0.0054451	-1.0529211	-0.0682775	0.23900428	0.08990482	-0.3760814	-0.4934948	0.07237585	0.1404943	0.9000858	0.44120852	1	0.26349418	0.93264664	0.37109337
Muribaculaceae species incertae sedis [ext_mOTU_v26_18996]	0.70505866	-0.8584445	-0.0848984	-0.7365163	-1.4382629	-4.2759055	1.48590466	0.01211	0.03494356	0.56188965	0.23242188	0.5	0.05993652	0.01611328	0.03125
Muribaculaceae species incertae sedis [ext_mOTU_v26_23390]	-1.1506158	-1.4825344	-1.1803287	-1.0732368	-1.1309362	-2.2497164	-0.0251545	0.95900646	0.96029732	0.00515747	0.375	0.75	0.00041962	0.00927734	0.03125
Muribaculum intestinale [ref_mOTU_v25_10099]	0.73223944	-0.0867953	1.37406345	-0.1353607	-0.463214	-2.6657455	1.09599373	0.0076991	0.02640466	0.08325195	0.76953125	0.25	0.5508728	0.62207031	0.03125
Odoribacter species incertae sedis [ext_mOTU_v26_18781]	0.78103451	-0.0502997	0.44207914	0.61187903	-1.3244501	-2.0833083	0.82204093	0.2426195	0.33970144	0.00918579	0.6953125	0.75	0.00336456	0.38037109	0.4375
Odoribacter species incertae sedis [ext_mOTU_v26_18805]	-0.2757183	-0.8778721	0.19863189	0.49525335	-0.1526611	0	-0.6486211	0.15087485	0.24406225	0.36272651	0.17752985	1	0.52936811	0.85513214	NA
Olsenella species incertae sedis [ext_mOTU_v26_15401]	1.32133839	-0.4990509	-1.2873678	0.42200229	-0.3777632	-2.1843411	0.53935728	0.36178203	0.45222754	0.07556057	0.58388242	0.5	0.30655627	1	0.58963855
Oscillibacter sp. 1-3 [ref_mOTU_v25_07502]	-1.2341361	-1.4762289	-1.2971187	0.58287295	1.49396488	1.53556799	-2.3343443	1.97E-05	0.00038197	0.10797321	0.36272651	1	0.5720817	0.01426619	0.18144921
Oscillibacter species incertae sedis [ext_mOTU_v26_18744]	-0.4579066	0.03650747	-1.3102785	0.80623183	-0.1638332	0.38433469	-0.8199983	0.23346902	0.33208955	0.67498671	0.81270369	0.37109337	0.02536986	0.75565774	0.58963855
Oscillibacter species incertae sedis [ext_mOTU_v26_18816]	-0.7591179	-0.3148844	-1.7542873	-0.3104203	-1.265713	-0.0993401	-0.1278092	0.80766944	0.8498855	0.175354	1	0.25	0.6705246	0.05224609	0.5625
Oscillibacter species incertae sedis [ext_mOTU_v26_18818]	-0.8807982	-1.3011943	-0.8067453	0.32313036	0.66251337	1.9540781	-1.6692386	0.00024894	0.00178589	0.06654572	0.10831938	0.25	0.15513157	0.07592696	0.10034825
Oscillibacter species incertae sedis [ext_mOTU_v26_18825]	-0.7520039	-0.2766567	-0.5299986	0.14229504	-0.0142767	3.00625676	-1.0235178	0.09061932	0.16521754	0.46374512	0.6953125	0.75	0.32472992	0.51855469	0.03125
Oscillibacter species incertae sedis [ext_mOTU_v26_18880]	0.53777492	-0.5471752	-1.8380205	0.07479951	-0.236285	0.02156591	-0.1035125	0.84327957	0.87510144	0.10458374	1	0.5	0.14224517	1	0.6875
Oscillibacter species incertae sedis [ext_mOTU_v26_18885]	-0.9123823	0.04434315	0.87933844	1.09221758	1.1543732	3.48355622	-1.7805679	0.0006655	0.00375333	0.52816772	0.375	0.5	0.01593018	0.00927734	0.03125
Oscillibacter species incertae sedis [ext_mOTU_v26_18942]	-0.4193747	-1.7799032	-2.2802062	-0.0027829	0.22450833	2.21872136	-1.4928552	0.02267395	0.05542521	0.97994995	0.05802402	0.25	0.32472992	0.33935547	0.15625
Oscillibacter species incertae sedis [ext_mOTU_v26_18975]	-0.2570808	-0.1509538	-0.3474497	0.01397629	-0.4460748	0.04560789	-0.0978479	0.78332587	0.83656161	0.67260382	0.58963855	1	0.78740649	0.78926803	1
Oscillibacter species incertae sedis [ext_mOTU_v26_18979]	0.2251487	0.49166015	0	0.43664995	0.57646824	1.19197409	-0.2897449	0.35257919	0.44914858	0.42267807	0.18144921	NA	0.19251757	0.28071267	0.37109337
Oscillibacter species incertae sedis [ext_mOTU_v26_23349]	-0.7541834	-0.4199779	-0.9028075	0.85165841	1.58367806	2.81420606	-2.0034603	9.60E-05	0.00093181	0.09383864	0.84570313	1	0.04316711	0.00683594	0.03125
Oscillibacter species incertae sedis [ext_mOTU_v26_23389]	-1.1851893	-2.4072167	-0.7966137	-0.1017526	-0.960281	-1.98094	-0.9403403	0.14578062	0.24174676	0.05592968	0.10732756	0.37109337	0.72676781	0.10000062	0.3125
Oscillibacter species incertae sedis [ext_mOTU_v26_23392]	-0.6710546	0.38418871	-0.0801325	0.1730091	-0.0513578	0	-0.301578	0.21664936	0.31289063	0.18343062	0.17752985	1	0.78740649	0.78926803	NA
Oscillibacter species incertae sedis [ext_mOTU_v26_27820]	-1.3401131	-0.5396891	0.41814657	0.59417357	0.76424664	3.03573643	-1.8116998	0.00222115	0.00977307	0.06868016	0.83363488	0.75	0.04650424	0.05592968	0.03125
Oscillibacter species incertae sedis [ext_mOTU_v26_33432]	-0.0269113	0.3500488	1.24014527	-0.2312768	1.99967775	5.11676959	-0.940723	0.15184449	0.2444326	0.70642712	0.47719662	0.75	0.89714249	0.03667129	0.03125
Parabacteroides goldsteinii [ref_mOTU_v25_01679]	2.04549	3.40117521	0.55639963	0.23713094	-0.5115624	-1.1377491	2.53209247	4.61E-05	0.00058573	0.00509932	0.02086258	1	0.22401538	0.14148212	0.37109337

Prevotella species incertae sedis [ext_mOTU_v26_18740]	0.16261722	0.91393721	-0.776525	-0.3713746	-1.1637246	-1.8786718	1.14277072	0.01009798	0.03115861	0.35895144	0.05905823	1	0.5633178	0.10831938	0.37109337
Prevotellaceae species incertae sedis [ext_mOTU_v26_18833]	0.98015837	0.26270914	0.72042916	0.24877529	-0.4164123	0	0.69621481	0.0273175	0.06328108	0.01426619	0.41849223	1	1	0.18144921	NA
Pseudomonas sp. [ref_mOTU_v25_00201]	-0.4027215	-0.6443543	-1.0381198	0.2151315	0.6031172	1.30406388	-1.051552	0.01147123	0.03350006	0.37109337	0.37109337	1	0.58963855	0.36131043	0.18144921
Roseburia species incertae sedis [ext_mOTU_v26_18838]	-1.0240899	-1.4735327	0.85387384	0.02317637	-0.2052426	3.27807827	-1.3288647	0.14893632	0.24211323	0.09383864	0.84570313	0.25	0.11825088	0.09228516	0.03125
Roseburia species incertae sedis [ext_mOTU_v26_23385]	-0.0159432	1.99025941	-0.3739439	0.03280298	-0.9291549	-1.1464785	1.08827463	0.0274218	0.06328108	0.94418251	0.03603169	1	0.72628615	0.40167817	0.37109337
Ruminococcaceae species incertae sedis [ext_mOTU_v26_18745]	-0.5917742	-1.6058907	-0.7093094	0.84016235	1.33579266	2.06321937	-2.1286876	0.00334837	0.01364152	0.86026001	0.63558612	0.5	0.0384903	0.01611328	0.0625
Ruminococcaceae species incertae sedis [ext_mOTU_v26_18846]	-0.7015631	-1.1463863	-1.6111116	0.16694943	-0.6314985	0	-0.853814	0.04270254	0.09150543	0.05870741	0.17752985	1	0.41849223	0.18144921	NA
Ruminococcaceae species incertae sedis [ext_mOTU_v26_18940]	-0.3327214	0.69037409	-0.1409718	0.59763223	0.20955392	2.71374086	-0.6951394	0.1821978	0.27707499	0.41011699	0.10732756	1	0.19553886	0.27189871	0.10034825
Ruminococcaceae species incertae sedis [ext_mOTU_v26_18990]	-2.2105148	-2.1575397	-1.4281917	0.0758128	-0.3303574	1.080346	-2.1704243	3.07E-05	0.00044108	6.10E-05	0.00195313	0.25	0.22875214	0.33935547	0.03125
Ruminococcaceae species incertae sedis [ext_mOTU_v26_18992]	-0.5173049	-1.14201	-1.7032317	0.23652085	0.76077828	1.51010172	-1.4596193	0.02020116	0.05127986	0.90632939	0.35254214	0.37109337	0.1147685	0.26640242	0.4375
Ruminococcaceae species incertae sedis [ext_mOTU_v26_23310]	-0.4398219	1.17273158	-1.3262632	-0.0204127	-0.130678	0.18439127	0.05236592	0.92305098	0.93437676	0.2621926	0.15078557	0.5	0.29338289	0.17626953	0.3125
Ruminococcaceae species incertae sedis [ext_mOTU_v26_23393]	-0.4559926	1.57108477	-0.0936407	0.02028852	-0.541956	0	0.47314438	0.20102003	0.29685703	0.03603169	0.10564543	1	0.28071267	0.42267807	NA
Ruminococcaceae species incertae sedis [ext_mOTU_v26_27743]	-0.3502074	0.42458962	-1.3116915	0.20574999	-0.2974717	1.40292774	-0.3915964	0.31781276	0.40968051	0.78926803	0.36131043	1	0.18144921	0.78926803	0.37109337
Ruminococcaceae species incertae sedis [ext_mOTU_v26_27750]	-0.7499842	-1.112836	-2.5216644	0.25849697	-0.9185491	-1.1642796	-0.7813427	0.07709933	0.14622286	0.05191296	0.18144921	0.37109337	0.72628615	0.18144921	0.37109337
Ruminococcaceae species incertae sedis [ext_mOTU_v26_27777]	4.71E-05	0.72877384	1.39910355	-0.4123763	0.28035847	1.25751429	0.3955562	0.38480086	0.47738453	1	0.37109337	1	0.20124262	0.58388242	0.58388242
Ruminococcaceae species incertae sedis [ext_mOTU_v26_27824]	-1.030197	1.35389663	0.76947175	0.82121614	0.27315605	2.88986366	-0.8783651	0.13740269	0.22900449	0.04231527	0.04231527	1	0.34637328	0.81270369	0.0625
Ruminococcaceae species incertae sedis [ext_mOTU_v26_33465]	-0.2194059	0	0	0.12372791	0.33315565	0.02906958	-0.2947673	0.1005616	0.17705502	0.42267807	NA	NA	1	1	1
Ruminococcaceae species incertae sedis [ext_mOTU_v26_33488]	-0.8308448	-0.1187849	-3.094202	0.57448965	0.44076549	3.49892906	-1.7707678	0.0283342	0.06493253	0.75361035	0.19251757	0.5	0.47765974	0.35895144	0.0625
Ruminococcaceae species incertae sedis [ext_mOTU_v26_33503]	-1.2396879	0.50737671	-0.8711065	0.68774258	-0.6326627	1.03774781	-0.876455	0.15873712	0.25184254	0.43319702	0.84570313	0.75	0.08834899	0.41482307	0.10564543
Ruminococcus species incertae sedis [ext_mOTU_v26_18826]	1.41527538	0.11473266	0.09802094	0.41704703	-1.641279	-2.1230646	1.38464884	0.00760589	0.02640466	0.01426619	0.58963855	1	0.03461056	0.14221324	0.37109337
Ruminococcus species incertae sedis [ext_mOTU_v26_23318]	-0.1124347	-0.1793278	-0.4580842	0.03874332	-0.4883834	0	-0.0393048	0.91127657	0.92815206	0.36272651	0.28071267	1	0.10564543	0.18144921	NA

Supplementary Table 5. Comparison of gut microbiome functional profiles of BDL and ShamOP mice. Alpha and beta diversities of pathways and ECs were compared. A linear mixed model was used to compare alpha diversities. Permutational multivariate analysis of variance was used to analyze beta diversities. P-values are shown.

	**Alpha diversity**		
	**Shannon**	**Simpson**	**Richness**
**Pathways**	0.00016924	6.42E-05	0.00095087
**EC**	0.00069026	8.54E-04	0.0012079

	**Beta diversity**			
	**Day 0**	**Day 1**	**Day 3**	**Day 7**
**Pathways**	0.12	0.002	0.559	0.213
**EC**	0.096	0.005	0.709	0.078

		**Beta diversity**					
		**day0-day1**	**day0-day3**	**day0-day7**	**day1-day3**	**day1-day7**	**day3-day7**
**Pathways**	**BDL**	0.001	0.009	0.016	0.012	0.129	0.43
	**ShamOP**	0.004	0.008	0.006	0.278	0.115	0.92
**EC**	**BDL**	0.001	0.017	0.013	0.014	0.29	0.139
	**ShamOP**	0.001	0.007	0.009	0.281	0.208	0.908

Supplementary Table 6. Linear model result for pathways. A positive coefficient indicates BDL mice > ShamOP mice, negative indicates the opposite. P-values and FDR-adjusted P- values are shown.

feature	mean normalized abundance BDL day1	mean normalized abundance BDL day3	mean normalized abundance BDL day7	mean normalized abundance shamOP day1	mean normalized abundance shamOP day3	mean normalized abundance shamOP day7	coef	P-value	FDR
PWY.6737	-0.6959318	-0.1286163	0.10824142	0.58338142	0.69631448	1.37680175	-1.121039	2.22E-08	8.07E-06
PWY.7184	0.60455562	0.21650132	0.35836625	-0.3443234	-0.6849217	-1.8592874	1.09190392	7.28E-07	7.13E-05
PWY.6125	0.49295138	0.25436287	0.53124248	-0.2782282	-0.5565139	-1.6160092	0.95831075	7.67E-07	7.13E-05
PWY.7197	0.65301419	0.24036808	0.34133747	-0.3351123	-0.692325	-1.8589561	1.12102838	8.78E-07	7.13E-05
PWY.7228	0.52913818	0.19923146	0.45416909	-0.3662271	-0.7046243	-1.9439687	1.08749505	1.35E-06	7.13E-05
PWY0.166	0.40227237	0.21691922	0.20682679	-0.2074067	-0.4481499	-1.2110545	0.73082448	1.41E-06	7.13E-05
PWY.7400	-0.3705939	0.11613115	0.31235602	0.63312505	0.81055898	1.50312544	-0.9200551	1.86E-06	7.13E-05
ARGSYN.PWY	-0.3718348	0.11895988	0.31303613	0.63785971	0.81785587	1.51648515	-0.9263267	1.93E-06	7.13E-05
PWY.5840	8.50870582	4.06882326	3.80427041	0.21125743	1.0215874	1.67995773	5.68963904	2.26E-06	7.13E-05
ARGSYNBSUB.PWY	-0.4448661	0.13200295	0.31290987	0.67819496	0.88834938	1.61433561	-1.0177125	2.49E-06	7.13E-05
GLUTORN.PWY	-0.4940335	0.16226993	0.37481461	0.7795073	1.03237393	1.76275166	-1.1466344	2.54E-06	7.13E-05
PWY.5897	8.57410599	4.04813206	3.8820114	0.23135069	1.11289458	1.67299541	5.68616746	2.75E-06	7.13E-05
PWY.5898	8.57410599	4.04813206	3.8820114	0.23135069	1.11289458	1.67299541	5.68616746	2.75E-06	7.13E-05
PWY.5899	8.57410599	4.04813206	3.8820114	0.23135069	1.11289458	1.67299541	5.68616746	2.75E-06	7.13E-05
PWY.7208	0.48901094	0.16571737	0.48063219	-0.3039308	-0.628177	-1.7182918	0.9694781	3.88E-06	9.40E-05
PWY.5791	7.77070386	3.41538109	3.72737892	0.29172335	1.04040929	1.48314992	5.03878109	4.64E-06	9.90E-05
PWY.5837	7.77070386	3.41538109	3.72737892	0.29172335	1.04040929	1.48314992	5.03878109	4.64E-06	9.90E-05
PWY.5838	8.57198247	3.47161253	3.87710654	0.63890134	1.12814088	1.65945924	5.25664042	9.05E-06	0.00018242
PWY.7199	-0.5148022	-0.2908673	-0.7888051	0.22842023	0.10865017	0.5406516	-0.6992229	9.59E-06	0.00018328
PWY.5861	8.28784271	3.30624705	3.80169672	0.6311633	1.10599962	1.59580415	5.05995853	1.03E-05	0.00018725
PWY0.862	7.02620595	3.10915489	3.42203457	0.72301901	-1.6202433	-3.4486178	5.79024594	1.22E-05	0.0002019
PYRIDNUCSAL.PWY	6.32323819	3.92802688	5.58487794	-1.3127795	-0.7855346	1.56866589	6.14582111	1.22E-05	0.0002019
PWY.5989	6.83811955	3.03724176	3.34595305	0.71389873	-1.6162351	-3.605818	5.68129967	1.33E-05	0.00020997
GLYCOLYSIS.TCA.GLYOX.BYPASS	6.32909179	4.87107204	6.00431482	0.32470188	1.21504715	1.7021397	4.97530599	1.54E-05	0.00023319
REDCITCYC	5.94109611	3.40539061	4.78488028	0.55199085	0.92155477	0.22136306	4.27148921	1.79E-05	0.00025988
PWY.5367	7.57619969	2.11779747	3.50530286	0.3851682	0.92804955	1.3381	4.45554782	2.35E-05	0.00032808
PWY.5863	7.03761792	2.74351389	3.5291816	0.26326172	0.35085599	1.43586485	4.64064268	2.46E-05	0.0003302
TCA.GLYOX.BYPASS	6.08728429	4.39455868	5.58807689	0.34744766	1.19239243	1.63695878	4.63267509	2.77E-05	0.00035944
FUC.RHAMCAT.PWY	6.20639918	4.20959048	7.96137622	0.94689375	1.17132272	1.55820477	4.61346321	3.13E-05	0.00039218
PWY.841	0.34872211	0.12783356	0.14793276	-0.1994681	-0.4586432	-1.3064394	0.6757077	3.57E-05	0.00043162
PWY.6151	-0.2740889	-0.1466916	0.60848062	0.31274602	0.59476018	1.10002053	-0.6294049	3.90E-05	0.00045672
PWY0.1296	-0.3364777	0.08308274	0.56128119	0.58161617	0.77875094	1.87157948	-0.8935066	4.27E-05	0.00048441
PRPP.PWY	7.09566947	4.28281324	6.85899699	-0.2101161	1.89649731	1.88313117	5.31479612	4.75E-05	0.00052299
PWY.7254	6.71245881	2.93263754	3.63538911	-0.0665193	0.9088548	0.34786224	4.68877435	4.94E-05	0.00052775
PWY.6282	5.91229932	3.06696787	3.42370984	0.73157902	-1.6086248	-3.5414889	5.19643404	6.08E-05	0.00060702
PWY.7388	5.87474336	2.98385553	3.60154503	0.73961183	-1.457346	-3.298289	5.08325354	6.12E-05	0.00060702
FASYN.INITIAL.PWY	5.82512018	2.98787204	3.54105678	0.7357965	-1.4998634	-3.3522076	5.07438376	6.44E-05	0.00060702
METH.ACETATE.PWY	-2.147563	0.10947696	-0.3924587	1.80558507	2.55407304	6.7380268	-3.8318142	6.49E-05	0.00060702
PWY.6803	5.84845408	3.37728968	5.23143813	0.0450508	1.08392684	2.06081978	4.2471074	6.66E-05	0.00060702
PWY.7664	5.87651216	3.1361407	3.45446676	0.74774952	-1.6942144	-3.662937	5.24125023	7.07E-05	0.00060702
GLYOXYLATE.BYPASS	5.78557056	4.99100254	5.74933125	0.34246651	1.28265523	2.34108978	4.5864531	7.10E-05	0.00060702
PWYG.321	5.90566561	3.1724754	3.54338245	0.76325734	-1.7102803	-3.6886046	5.280525	7.11E-05	0.00060702
ILEUSYN.PWY	-0.1738923	0.09018687	0.47671074	0.451463	0.65906478	1.12219023	-0.6082378	7.36E-05	0.00060702
VALSYN.PWY	-0.1738923	0.09018687	0.47671074	0.451463	0.65906478	1.12219023	-0.6082378	7.36E-05	0.00060702

FASYN.ELONG.PWY	5.88526562	3.19976844	3.48018199	0.76302344	-1.7678036	-3.7753055	5.302147	7.56E-05	0.00060986
PWY.7328	4.71478524	1.70105242	5.11724455	-0.3203843	0.78905582	1.33342011	3.43605085	7.78E-05	0.00061428
PWY.6519	5.57908703	3.11574446	3.39868047	0.74468509	-1.7139426	-3.772469	5.09407686	8.77E-05	0.00067715
PWY.5971	7.99683149	2.80514007	4.00149717	0.37017595	1.33663621	1.76501561	4.79797397	9.18E-05	0.00068629
PWY.6113	7.77793378	2.71220697	3.96339646	0.3699357	1.2470129	1.70561341	4.68317138	9.26E-05	0.00068629
P105.PWY	5.36111575	4.05813531	5.68082527	0.23883198	1.07887256	1.63135763	4.24162076	9.69E-05	0.00070027
PWY.6284	7.31994895	2.45506684	3.78786856	0.37374437	1.09877434	1.55031617	4.39788889	9.84E-05	0.00070027
PWY.6630	5.77433596	3.29832849	5.50583862	-0.070413	0.51392573	1.46392746	4.54172439	0.00010385	0.00071284
BIOTIN.BIOSYNTHESIS.PWY	5.54671829	3.20647911	3.39007542	0.76344672	-1.8493093	-3.9583037	5.16687114	0.00010408	0.00071284
PWY0.1415	5.4389277	2.44210675	3.35587191	-0.1587525	0.96591124	1.3373552	3.71562864	0.00012478	0.00083883
PWY.561	5.42425608	3.76146239	5.57614294	0.30291408	1.1343466	1.5822501	4.11035202	0.00013899	0.00091733
PWY.5138	7.10504435	3.64532362	3.62730707	1.24514577	2.34297109	2.10930569	3.73474281	0.00017701	0.00114738
PWY.7111	-0.1420624	0.1323931	0.53393864	0.4710298	0.65906478	1.12219023	-0.5802007	0.00018908	0.00120416
PWY.6121	-0.157906	-0.0590299	-0.3615022	0.26951875	0.04190751	0.34367876	-0.3524636	0.00021991	0.00137636
PWY.6478	-1.3867207	-1.529032	0	1.15049926	0.72518904	4.37787409	-2.7020673	0.00023436	0.00144188
CITRULBIO.PWY	6.08923719	4.10161979	5.95933781	0.88736302	1.50571018	1.80335934	4.15888325	0.00028126	0.00167617
PWY.7332	-3.2341452	0.30588783	-0.0315984	2.02877435	1.17818455	5.84950753	-3.839402	0.00028167	0.00167617
PWY.6122	-0.1709285	-0.1004329	-0.21134	0.21891961	0.0783204	0.25062688	-0.3278938	0.00029626	0.00170701
PWY.6277	-0.1709285	-0.1004329	-0.21134	0.21891961	0.0783204	0.25062688	-0.3278938	0.00029626	0.00170701
PWY.6953	-1.6825891	-1.2272653	-1.8904974	0.8561751	1.33180221	1.03043001	-2.5818141	0.00033184	0.00187336
PWY.5675	6.2440479	3.70123451	5.32356177	0.8485011	1.17794542	1.61740747	4.17305432	0.00033545	0.00187336
PWY.7219	-0.2238651	-0.1602353	0.06552765	0.16398706	0.21427596	0.53558813	-0.3951381	0.00035939	0.00197664
GLYCOCAT.PWY	5.75467465	2.33504547	5.83525834	0.29401574	0.90003826	1.44312065	3.90967441	0.00039325	0.00210558
PWY.5083	6.09777367	3.4561397	5.79166305	0.40092211	2.04413982	2.40070766	3.92221918	0.00039443	0.00210558
P4.PWY	4.80568378	3.81260763	7.39164982	0.58672132	1.97258832	0.52494643	3.73786032	0.0004065	0.00213852
PWY.4702	6.16620137	3.17854463	5.32596205	0.96015183	1.21297614	1.53790246	3.88605821	0.00041761	0.00216559
PWY.6731	5.38649118	2.14660178	5.36829132	0.30983641	0.84565325	1.29942355	3.62119685	0.00044161	0.00224262
PWY0.781	4.80727571	3.75994513	7.40874994	0.56460294	1.99392144	0.56975172	3.72121751	0.00044482	0.00224262
PWY.5747	5.04711905	2.81412423	3.39422228	0.38701857	0.99977998	1.26049173	3.35094396	0.00048402	0.00239234
HEME.BIOSYNTHESIS.II	6.48224626	2.81130106	3.82013799	0.87700024	1.15673022	0.59468416	3.92406711	0.00049292	0.00239234
PWY.2723	5.32296408	2.08836849	5.22650683	0.31343591	0.84654509	1.27988611	3.55028004	0.00050075	0.00239234
PWY.6609	-0.2620808	-0.1367332	-0.0981951	0.15782357	0.06004629	0.43652158	-0.359961	0.00050088	0.00239234
PWY0.42	5.13156257	2.80923615	3.45392021	0.39329742	1.03157882	1.2821087	3.38405206	0.00051607	0.00243292
PWY.7090	-0.5529404	-0.4661634	0	1.77283702	0.42693824	2.41444422	-1.8702169	0.00055228	0.00257025
PWY.5918	6.94292007	2.97064638	3.82715293	0.93121902	2.05742505	1.51416754	3.77699163	0.0005774	0.00265309
PWY.7211	5.64870497	3.90595684	3.82435521	0.27782005	1.67630028	1.64853836	3.89141837	0.00063712	0.00289091
ARO.PWY	-0.248476	-0.1590263	0.23549533	0.30565228	0.27129695	0.56865121	-0.486402	0.00065339	0.00292815
PWY.6317	-0.1560841	0.26114849	0.47495799	0.51851806	0.57790718	1.55709093	-0.6053475	0.00075018	0.00331554
NAD.BIOSYNTHESIS.II	5.30563251	2.46713852	3.56754214	0.36353172	0.94637754	1.3585326	3.40188329	0.0007581	0.00331554
PWY.7046	5.32772626	2.55080525	3.61504714	0.37716537	1.0773154	1.32595257	3.40287278	0.00078471	0.00338879
HCAMHPDEG.PWY	5.4154581	2.37904817	5.06941076	0.44515336	1.22838718	1.39046961	3.4714227	0.00080285	0.00338879
PWY.6690	5.4154581	2.37904817	5.06941076	0.44515336	1.22838718	1.39046961	3.4714227	0.00080285	0.00338879
PWY.6527	-0.1522091	0.30514813	0.57504701	0.53532542	0.69839178	1.68596858	-0.6409893	0.00082894	0.00345869
TRPSYN.PWY	-0.3128199	-0.1095719	0.30429334	0.59653099	0.79092264	1.45650304	-0.9384072	0.00084397	0.00347526
METHGLYUT.PWY	5.78809127	2.57714882	3.67980963	0.41726376	1.41239424	2.10787306	3.42677123	0.00085206	0.00347526
PWY.6612	3.58961886	2.03548532	6.59440379	-2.2715425	-0.0770836	5.184418	3.98019388	0.00089282	0.00359155
PWY.5005	-2.5664235	0.46576672	2.9111886	1.75808941	2.33859467	5.53292012	-3.265249	0.00090036	0.00359155
PWY.5304	-0.9447173	-1.4323641	-0.1870377	0.7900549	0.78165749	0	-1.708115	0.0009331	0.003674
PWY.6470	-1.6079478	1.0507266	3.23586349	3.37890563	2.30570511	2.09407858	-2.9308186	0.00094127	0.003674
FOLSYN.PWY	3.68727317	2.18911144	6.8955052	-2.4395682	-0.0800745	5.55266613	4.16608929	0.00096648	0.00373225
PWY.7210	5.27688139	3.54251438	3.65841617	0.63970974	1.5603396	1.52347588	3.4065479	0.0010472	0.0040014

GLUCOSE1PMETAB.PWY	5.41767203	2.81479099	5.42471749	0.61727357	1.15122533	2.18892055	3.49782993	0.00110528	0.00417934
PWY.6834	-2.1533437	-3.9418753	-5.1245994	1.0895836	-0.9158746	0.86236105	-3.5531755	0.00112729	0.00421861
PWY.6285	7.08849668	1.20016461	3.88031361	0.36926089	1.17605486	1.6129823	3.81248446	0.00115194	0.00422025
PWY0.1338	4.35125336	1.63724666	4.09624955	0.52337409	0.68962651	1.04728578	2.71200287	0.00115867	0.00422025
PWY66.422	-0.1363174	0.26492391	0.56382049	0.51851806	0.57962879	1.55709093	-0.5833405	0.0011626	0.00422025
ORNDEG.PWY	5.81183034	2.20927109	3.82947529	0.37745296	1.07489391	1.39798168	3.55266137	0.00126103	0.00445403
PWY.6629	6.00489272	2.87492703	3.82902561	0.25793476	1.23800572	1.7244764	3.85766562	0.00126986	0.00445403
ARGDEG.PWY	5.61262637	2.09118536	3.71728599	0.3296021	1.03922687	1.37848877	3.43298601	0.00128559	0.00445403
ORNARGDEG.PWY	5.61262637	2.09118536	3.71728599	0.3296021	1.03922687	1.37848877	3.43298601	0.00128559	0.00445403
DTDPRHAMSYN.PWY	-0.2690627	-0.1031113	-0.0742443	0.11230097	0.20479962	0.35942225	-0.3614911	0.00128836	0.00445403
PWY.7391	5.33114917	0.66689177	1.78605212	0.51300504	0.62091315	1.69136141	2.54141261	0.00156107	0.00534593
PWY.6531	4.73068333	3.17497375	5.08702526	0.56318299	1.37500087	1.38923955	3.28546175	0.00175007	0.00584804
PWY.5656	5.42118172	2.11894631	3.63407904	0.40882339	1.14299685	1.3922747	3.25377343	0.00175352	0.00584804
PWY.5173	5.45146662	1.96393873	3.70196204	0.08844667	1.01645308	1.42362128	3.4336621	0.00175602	0.00584804
PWY0.1277	5.53582222	2.20797098	3.80854562	0.43520146	1.21621596	1.45998323	3.31950827	0.00195306	0.00644511
X3.HYDROXYPHENYLACETATE.DEGRADATION.PWY	5.29293123	2.02613595	4.76826872	0.61018522	1.4218996	1.33179428	3.10041099	0.00208738	0.00676775
GALACTARDEG.PWY	5.61313983	2.68291465	6.04796894	0.61941691	1.52689825	1.51118718	3.59445318	0.00210701	0.00676775
GLUCARGALACTSUPER.PWY	5.61313983	2.68291465	6.04796894	0.61941691	1.52689825	1.51118718	3.59445318	0.00210701	0.00676775
COMPLETE.ARO.PWY	-0.1385076	-0.0346205	0.12450697	0.36225371	0.37335983	0.60544084	-0.4673081	0.00212541	0.00676775
BRANCHED.CHAIN.AA.SYN.PWY	0.09850235	0.34593188	0.30856805	0.55940292	0.80883528	1.38278125	-0.5374402	0.00214536	0.00677187
PWY.7288	5.6319056	2.35964576	2.16186846	1.06254977	2.23811844	1.53818763	2.53707685	0.00219832	0.00687923
PWY.6876	-0.9600308	-1.1282223	-1.7573189	0.52871894	0.41177897	2.42379647	-1.8565888	0.00230732	0.00715862
GLUCARDEG.PWY	5.26550682	2.56369102	5.68493599	0.58060623	1.45787129	1.47096869	3.37473856	0.0023797	0.00732061
GLYCOL.GLYOXDEG.PWY	4.41041967	2.21208654	3.13530915	0.54637367	1.16644911	1.09194448	2.65413739	0.00246478	0.00751348
ASPASN.PWY	0.47467921	0.08235288	0.25343232	-0.1786115	-0.1433889	-0.1133648	0.46709276	0.00248379	0.00751348
PWY.6549	-2.2070842	-0.7688924	1.51913051	0.47074517	0.85734212	3.66143005	-2.2575221	0.00267644	0.00802932
PWY.7187	0.31915264	0.30797086	0.27988248	0.00951206	-0.1175248	-0.5549345	0.41665308	0.00272787	0.00811654
PWY.5845	5.99655638	1.14587855	3.78066895	-0.1314485	1.06920577	1.64032113	3.51191623	0.00288445	0.0085021
PWY66.391	6.04874539	2.70260044	2.48287645	1.18212411	2.55483841	1.83503707	2.70850052	0.0029043	0.0085021
PWY.5862	5.80521312	1.13920267	3.68121968	-0.0793187	1.04113637	1.57391544	3.38698039	0.00299087	0.00868548
AST.PWY	4.96025868	1.91785373	3.40790543	0.58707661	1.23992771	1.2040335	2.81020027	0.00302873	0.00872562
PWY.6749	-2.7672488	-0.5006604	-0.6668675	0.48087385	1.42528015	4.23603093	-2.9907103	0.0031439	0.00898612
PWY.5097	-0.1804228	0.05609433	0.5456917	0.33138508	0.49385076	0.84642768	-0.4599508	0.00331783	0.00938345
PWY.7316	-1.4006281	-1.1034428	-2.5390363	1.14601106	0.52495007	3.44464482	-2.6791528	0.00333819	0.00938345
KDO.NAGLIPASYN.PWY	4.26001318	1.61245274	2.92545927	0.49164734	1.12205884	1.04226957	2.38800273	0.00336047	0.00938345
PWY.6168	-0.2811925	-0.3010865	-0.0200098	0.2678157	0.19407329	0.76598382	-0.5652889	0.00345065	0.00956172
PWY.6163	-0.2450889	-0.1880962	0.1999501	0.19947644	0.12948984	0.30594158	-0.3608655	0.00349584	0.00961355
PWY.4981	0.14439358	0.1095388	0.70845981	0.59937421	0.5687106	1.15186933	-0.4606973	0.00373775	0.01020153
PWY.6837	3.75891753	1.54813779	2.87121848	0.48235377	0.99306803	0.53873658	2.20983693	0.00381595	0.0103111
PWY.6386	-0.1111632	-0.0271543	0.08843773	0.2964702	0.43900975	0.77076267	-0.4624423	0.00383471	0.0103111
DENOVOPURINE2.PWY	0.29206071	0.15660062	0.19747951	-0.0174167	-0.1345149	-0.6103578	0.36617475	0.00399473	0.0106624

PWY.5154	1.03347312	1.12287246	1.51075586	0.172526	0.01175143	0.39866905	0.97412871	0.00422198	0.0111764
PWY.7446	3.4829718	1.21214884	2.74888318	0.46064742	0.61470597	0	2.13800825	0.00424888	0.0111764
PWY.5103	0.15656325	0.38886386	0.25148991	0.57267834	0.82671481	1.42606734	-0.5175767	0.00429869	0.01122608
PWY.1861	-0.8002807	1.62620634	-1.9392166	2.64907806	2.69782689	2.26535864	-2.6950832	0.00478279	0.0124011
PWY.6147	1.51156687	1.11753795	1.96318599	-0.130394	0.47733749	0.54314662	1.27001688	0.00489406	0.01259034
PWY.7539	1.49899495	1.10539805	1.94867665	-0.1369201	0.47081383	0.53112992	1.26460695	0.00492515	0.01259034
POLYAMINSYN3.PWY	1.69062442	3.42730728	4.25404617	0.21386657	0.38273938	2.33593469	2.07200772	0.00497602	0.01263142
ENTBACSYN.PWY	5.81594319	1.12581805	5.8035116	0.64542201	1.49263012	1.66928494	3.09549137	0.00505895	0.01274998
ARGININE.SYN4.PWY	1.43251415	1.05033881	0.75477466	0.24246744	-0.3917466	-0.3704344	1.26006419	0.00509297	0.01274998
PWY.5686	-0.2576556	-0.1628447	-0.0548344	0.1453231	0.06932501	0.22853356	-0.3305463	0.00517505	0.01286673
PWY.5705	4.18286646	1.22833653	2.89006526	0.4993863	1.03845553	0.96806199	2.24021941	0.00560911	0.01385107
PWY.7204	4.76379425	1.81081277	3.49649032	0.60135905	1.23429293	1.3280471	2.65818415	0.00663133	0.01625526
PWY.5415	4.09393658	1.14138724	2.06728038	0.56342143	0.69110969	0.65531459	2.1823848	0.00667227	0.01625526
PWY.7315	-0.4183564	0.46773393	0.40909325	0.58678153	0.64250999	1.83864715	-0.7725533	0.00680167	0.01646005
RIBOSYN2.PWY	-0.2870812	-0.2279203	0.96745055	0.25843948	0.26665028	0.74667696	-0.4376163	0.00699549	0.01681697
PWY.5189	3.78182444	2.02536463	4.25649012	-0.0439866	0.80577428	0.43290186	2.92058575	0.00738319	0.01763221
PWY.6891	5.52649488	2.03761822	6.17531014	0.91587705	1.28726002	0.69930294	3.36105074	0.00769429	0.01825508
PWY.5180	4.97551999	1.55470455	3.78826119	0.64612803	0.89843721	1.44565187	2.78448531	0.00784114	0.01836344
PWY.5182	4.97551999	1.55470455	3.78826119	0.64612803	0.89843721	1.44565187	2.78448531	0.00784114	0.01836344
PWY.5692	3.57386284	0.85330331	2.70382788	0.46199413	0.61913602	0.78900724	1.94713561	0.00827444	0.01913135
URDEGR.PWY	3.57386284	0.85330331	2.70382788	0.46199413	0.61913602	0.78900724	1.94713561	0.00827444	0.01913135
PWY.7094	4.55814447	1.45760469	3.33201649	0.5651259	1.28050245	1.11607836	2.43759662	0.00951051	0.02185009
PWY0.1479	5.00331689	4.65980888	6.75106259	1.45699434	2.31081127	2.80458135	3.1819192	0.01031684	0.02355354
PWY.5505	-2.2282421	-0.5747758	0.05944527	0.41848158	-0.2207366	1.98367296	-1.7766368	0.01038303	0.02355651
HEMESYN2.PWY	4.84753529	3.36447164	6.755244	1.39875643	1.8518481	3.93838777	2.67519207	0.01089589	0.02456651
PWY.4984	2.90209706	3.79061233	4.27423434	0.3127902	0.4986699	1.37995089	2.87109301	0.01113451	0.02494955
PWY.6385	-0.0092034	-0.0430472	0.08038348	0.28615374	0.30794899	0.64056304	-0.3500569	0.01133176	0.02512172
GLUCONEO.PWY	-0.0241319	0.13165639	0.31346768	-0.2026517	-0.1054754	0.09984845	0.203486	0.0115589	0.02512172
PWY.5855	4.44484093	0.99014293	3.28404672	0.55995344	1.19337876	1.18729691	2.22886948	0.01162658	0.02512172
PWY.5856	4.44484093	0.99014293	3.28404672	0.55995344	1.19337876	1.18729691	2.22886948	0.01162658	0.02512172
PWY.5857	4.44484093	0.99014293	3.28404672	0.55995344	1.19337876	1.18729691	2.22886948	0.01162658	0.02512172
PWY.6708	4.44484093	0.99014293	3.28404672	0.55995344	1.19337876	1.18729691	2.22886948	0.01162658	0.02512172
PEPTIDOGLYCANSYN.PWY	-0.1806399	-0.1449436	-0.0678298	0.11493287	0.14991895	0.26217226	-0.2999118	0.01229196	0.02640225
PWY.6387	-0.1847821	-0.1473588	-0.0526041	0.11361293	0.16859714	0.30832442	-0.3125405	0.01239169	0.0264599
PWY.6143	-2.2596564	-0.8912404	-0.8044202	0.09551691	-0.623352	1.00427164	-1.5757523	0.01275257	0.02694136
PWY.821	4.56136862	3.6166272	6.05413231	-0.599756	2.54581462	4.99009266	3.24389693	0.01281828	0.02694136
PWY.2942	0.00595667	0.06655611	0.38549019	0.3630619	0.45186576	0.70207157	-0.3627648	0.01283982	0.02694136
PWY.6895	5.4588686	2.17081571	6.72981073	1.04036754	1.12316005	0.45413161	3.45949534	0.01326777	0.02767931
PWY0.41	3.92088774	0.9052465	2.87898299	0.4918043	1.05176069	0.87721869	1.99901496	0.01335356	0.0276991
COA.PWY.1	-0.2157555	-0.035202	0.46508041	0.14867751	0.32115587	0.55601819	-0.3270861	0.01386856	0.02860391
ECASYN.PWY	3.76655961	0.91865697	3.10824295	0.52398209	1.14915589	0	2.01698764	0.01535968	0.03150036
PWY.7269	4.88257435	1.35822422	3.4870157	0.59624659	1.84556384	1.26108781	2.36999037	0.01551004	0.03163003
PWY3O.355	4.48032436	1.05345236	3.49137597	0.59149662	1.13386204	1.26235332	2.28787186	0.01586941	0.0321821
PWY66.389	4.12429159	2.22523804	5.57378893	0.96460357	1.48224635	0.70042255	2.52966699	0.01597724	0.03222077
PWY.5030	0.14625907	0.27646648	-1.3550616	-0.1511945	-0.9352949	-1.7149052	0.62203409	0.0164423	0.03297544
PWY.6071	3.95515988	0.97244842	3.08851925	0.53879879	1.0605323	1.04842874	2.01565735	0.01711216	0.03413029
PWY0.321	3.88571548	0.95835691	3.05069332	0.53233176	1.05073249	1.02901854	1.97895402	0.01754664	0.03465141
HSERMETANA.PWY	-0.1312265	-0.0713447	-0.1005407	0.30576752	0.23966239	0.32581064	-0.3929795	0.01756435	0.03465141
PWY.5860	4.78146755	1.13920267	3.68121968	0.65271293	0.90095645	1.45025286	2.51766903	0.01767904	0.03468915
PWY.7616	1.79141282	1.66254811	3.83466648	0.39049828	1.1849078	0	1.39650721	0.01802025	0.03498336
PWY.5850	4.90682926	1.14587855	3.78066895	0.66363324	0.93304226	1.5285894	2.57110177	0.01819337	0.03498336
PWY.5896	4.90682926	1.14587855	3.78066895	0.66363324	0.93304226	1.5285894	2.57110177	0.01819337	0.03498336
PWY.7385	1.65382453	2.17857646	2.59981566	-0.0440523	0.60059339	1.85469817	1.5389421	0.01825817	0.03498336

PWY.6263	1.59105924	-0.1916535	-0.4302764	-1.2584248	-1.4717913	1.33195661	1.70005504	0.01831085	0.03498336
UBISYN.PWY	4.17713199	1.00319417	3.3251868	0.56670376	1.21581137	1.20529556	2.08509059	0.02000943	0.0379724
PWY.5345	5.0421707	4.12139942	6.56099335	0.22353198	2.27337413	4.1452571	3.49146392	0.02008458	0.0379724
PWY.7013	3.47958033	3.09594932	4.51498364	0.84291046	0.97873075	1.9711776	2.43497887	0.02103027	0.03955435
PWY.7312	0	1.10695313	0	1.20305582	2.29790666	3.76575966	-1.5065436	0.02294966	0.04294188
PWY.5265	6.66938559	0.89630552	2.68304572	1.81263071	1.04430679	-1.271612	2.98033724	0.02335506	0.04347635
PWY.6823	3.85273075	0.98979878	3.17580858	0.55324301	1.15512278	1.1020653	1.93303601	0.02773648	0.05136909
LPSSYN.PWY	3.8583486	0.92782302	3.15106854	0.5379774	1.14284202	1.19207734	1.91145166	0.02830289	0.05215202
PWY0.881	4.85592885	1.15484912	3.77033465	0.66023309	1.54990217	1.45046717	2.35621165	0.03122772	0.05725083
PWY.7209	4.3421462	1.56859007	4.68317125	0.09636353	1.46155839	3.6844744	2.38630234	0.0327608	0.05975966
PWY.6606	2.02298188	0.9011048	1.54601223	0.0465264	0.87247364	2.16421038	0.97167552	0.03379478	0.06133753
PWY.6700	-0.1573223	-0.2821175	-0.2042897	-0.2493957	-0.632927	-1.1949106	0.29205198	0.03596238	0.06494698
THRESYN.PWY	0.84974177	0.61113956	0.27835216	0.24224882	0.40742587	0.29337004	0.39207147	0.03831943	0.06886115
P562.PWY	-1.6049466	0.78413122	0.38931654	0.69506507	1.17906408	4.87798752	-1.9174341	0.04197306	0.07505528
POLYISOPRENSYN.PWY	2.04701366	0.26416493	5.0524995	-1.7464176	1.05382855	2.47677763	2.05747591	0.04617587	0.08216589
PWY.6608	2.53851011	1.17854032	1.46783612	0.84338093	0.74445232	1.80898325	0.98782079	0.04844545	0.08578389
PWY.5100	-0.3817548	0.47197229	1.27915819	0.28642395	0.64422584	1.63051289	-0.4560632	0.05007578	0.08824034
PWY0.1586	2.0324855	1.04010248	2.79758659	0.76090822	0.88548746	1.93623459	0.81864187	0.05992084	0.10507857
PWY.724	0.3487462	0.42456903	0.21265535	0.53382649	0.75574875	1.27215719	-0.3446401	0.06054011	0.10565414
PWY.7220	0.30275706	0.42268159	0.81254001	0.13713958	0.20764524	0.00120439	0.26531163	0.06217532	0.10747448
PWY.7222	0.30275706	0.42268159	0.81254001	0.13713958	0.20764524	0.00120439	0.26531163	0.06217532	0.10747448
HISTSYN.PWY	-0.1090264	0.26866069	0.84966224	0.28872902	0.62565515	0.84640324	-0.3301942	0.0629052	0.10822079
PWY.5104	0.26836858	0.37367246	0.42717814	0.75125485	0.80310362	1.83750727	-0.5857862	0.06459999	0.11061225
SO4ASSIM.PWY	4.7461374	3.19319577	4.58849687	1.16703407	2.09761024	3.58711421	2.38675715	0.07650005	0.13037333
SER.GLYSYN.PWY	0.44048307	0.02476458	-1.3065313	0.07916254	-0.7833277	-0.8589141	0.405957	0.08510554	0.14436127
TRNA.CHARGING.PWY	-0.1898442	-0.1329492	-0.1082226	0.04481623	-0.0574874	-0.0251404	-0.1617713	0.0864314	0.14592836
P221.PWY	2.38813577	0.69935516	2.47169037	0.74379993	0.82332797	0.56686436	1.05357223	0.08805143	0.14797532
ANAEROFRUCAT.PWY	0.46883763	0.50597976	0.82150793	0.0983934	0.1860806	0.8234732	0.30284539	0.09275096	0.15515484
POLYAMSYN.PWY	3.81557687	2.62139236	5.7902081	0.43221212	1.92351181	4.26105191	2.210558	0.09508466	0.15832905
SALVADEHYPOX.PWY	2.16914564	1.06949138	2.27729919	0.58937365	0.88897255	2.3726451	0.8710884	0.09717053	0.16106348
PWY.5136	2.30309381	1.04554681	2.57950716	-1.4401229	2.0900815	4.97940797	1.33102262	0.09891975	0.16321759
SULFATE.CYS.PWY	4.68825336	3.53072764	4.60964628	1.23427627	2.21970024	4.02902219	2.34418297	0.11146336	0.18308236
PWY.6353	1.71534865	0.87285975	1.66019649	0.39217112	0.77744068	1.91091423	0.69221635	0.1122884	0.18360671
FAO.PWY	2.08638662	1.07311588	2.98111154	-1.5875936	2.19840377	5.21762894	1.28956486	0.1161979	0.18914725
ARG.POLYAMINE.SYN	3.71980257	2.69108583	6.22758037	1.11620803	2.08990976	1.69484963	2.15132452	0.11676236	0.18921757
PWY0.162	1.3067131	0.12507966	-1.6562088	0.28760291	-0.0082373	-0.7106802	0.4655337	0.11896787	0.19193483
HEXITOLDEGSUPER.PWY	1.33538889	1.24531497	2.4273234	0.64603999	0.24817672	1.85281865	0.76324685	0.12348365	0.19833878
PWY.6897	0.59195995	0.5114629	1.59926438	0.06667747	0.356687	0.94072693	0.41034663	0.12628751	0.20194875
PWY.5695	0.24173186	-0.0166462	0.44713376	-0.0428458	-0.1216122	0.09367603	0.2261331	0.12762137	0.20318665
HOMOSER.METSYN.PWY	2.04188299	1.06068043	2.18127672	-0.5053291	1.53616562	3.06136829	1.07892446	0.12838085	0.20350327
PWY.5088	-0.8855737	0.08914949	1.46536121	0.85775305	0.10323871	2.93043677	-1.1347443	0.13331509	0.21040599
PWY.1042	-0.089278	0.08249337	-0.4525412	0.03521239	-0.4483335	-0.690487	0.14568967	0.13974087	0.2195928
FUCCAT.PWY	3.30057108	2.35383889	5.80722687	1.60416963	0.70277961	4.06478776	1.64070265	0.14275706	0.22336558
PWY.6471	5.07233096	3.59134735	5.1111236	3.50230007	1.58157693	1.23716231	1.97751285	0.15097524	0.23521035

MET.SAM.PWY	1.76659232	1.00419972	2.14204531	-0.8379793	1.57344662	3.2880078	1.04796593	0.15209524	0.23594261
PWY.6703	0.1555729	-0.1366861	0.1408278	0.00842906	-0.3342877	-0.5196408	0.2235483	0.15708442	0.24160504
PWY.4242	-0.0862254	-0.022308	0.54734167	0.1411582	0.20613692	0.42446454	-0.1856039	0.15767099	0.24160504
PWY.6270	0.00339825	1.33644037	3.34458606	0.97813415	3.42300615	3.45809082	-1.2204246	0.15774213	0.24160504
PWY.5676	0.89605207	0.29782311	-1.2517043	1.21510871	1.17275053	2.59108339	-0.9610169	0.15876176	0.24214504
KETOGLUCONMET.P WY	2.92335157	1.52182482	5.00029712	0.22500803	1.35984425	2.99085317	1.72112789	0.15961048	0.24242095
PWY.5347	1.7097233	0.94136698	1.8427745	-0.7692354	1.44443379	2.87311535	1.01889226	0.16479473	0.24925203
P461.PWY	1.92054818	1.54573617	1.49001712	1.11652356	0.38384879	1.92520784	0.75253169	0.16594391	0.24959798
NONMEVIPP.PWY	0.1081168	1.51820516	3.64490025	1.21003009	3.6000332	3.75453252	-1.2868244	0.16639865	0.24959798
GLYCOLYSIS.E.D	1.52527421	1.40382337	2.66021607	0.01371817	0.48924212	3.76239566	0.9593913	0.17620758	0.26322367
METSYN.PWY	1.81709139	0.97924373	1.97993244	-0.6970229	1.57253777	3.18779988	0.98291369	0.18262796	0.27169652
PWY.5188	0.04948069	0.62081965	1.05878637	0.73699415	0.67352338	1.20371051	-0.4019218	0.19210547	0.28462974
COBALSYN.PWY	2.57982971	1.07982926	3.56607372	0.93405723	0.77655183	1.551504	1.20590769	0.20793863	0.30648178
HISDEG.PWY	-0.4264049	-0.4464597	-0.7353767	-0.3668841	-1.1251483	-1.9180398	0.34785917	0.2085427	0.30648178
PWY.7560	0.17092335	1.42763953	3.59772836	1.04153338	3.41948715	3.41206812	-1.0965263	0.21069886	0.30840195
PWY.6123	-0.0613885	-0.1624703	-0.4870512	0.07551694	-0.0347565	-0.0281828	-0.1751031	0.21244088	0.30970297
PWY.2941	-0.0695572	-0.1213356	1.27896436	0.06263788	0.60381763	1.67204059	-0.3740721	0.21637026	0.31416961
PWY.5121	0.08722519	1.17588662	3.28651945	0.30636003	3.49822917	4.57574923	-1.0487272	0.22202448	0.32109517
PWY.6595	0.98478134	-0.7048506	1.72096041	-1.4881001	0.19313825	2.8451908	0.84426179	0.2247038	0.32368047
UNMAPPED	-0.2787733	-0.1558511	-0.5579049	-0.1058678	-0.1567258	-0.1325295	-0.1434011	0.22864693	0.32805863
PWY.5022	0.48227615	0.28217194	1.2579696	-1.4084887	0.4803163	3.28072297	0.67977316	0.23109252	0.33026214
UNINTEGRATED	0.14496032	0.11823992	0.47446184	0.0392144	0.11623717	0.15247899	0.09653649	0.23377623	0.33278734
PWY.6588	1.69564304	0.79773401	1.39442241	0.03589507	0.02110054	1.60280929	1.10385064	0.24206596	0.34324197
GLYCOLYSIS	-0.0198102	0.17235104	0.59450716	0.06511738	0.41664069	0.99537026	-0.1790069	0.24333097	0.34369316
PWY.7229	0.07917784	0.09591507	0.17046596	0.18059198	0.23568849	0.35949238	-0.1258587	0.24444085	0.34392259
PWY0.1297	1.13526616	0.71431195	3.48992511	-1.0183791	1.60739701	3.03685259	0.89957008	0.24559644	0.34421431
P441.PWY	4.01813588	4.01716603	-0.5489215	0.98081645	2.31629199	5.21850751	1.4827513	0.24843444	0.3468527
PWY.6628	3.92576059	1.23813257	6.27109771	1.71229634	1.6231376	1.6883404	1.58134991	0.25136092	0.34959392
THISYNARA.PWY	0.11919157	0.0327552	0.53032281	0.18086418	0.50353137	1.23296317	-0.2858758	0.25411764	0.35207902
PWY.7198	-0.3315059	1.10605076	0.54890735	0.61506338	1.0655836	2.223315	-0.6884209	0.26430051	0.36479501
PWY.6859	0.54672094	-0.2948507	3.01408937	-2.9449503	1.31256915	5.13457624	1.038855	0.26562575	0.3652354
PWY.3001	0.65020765	0.53216224	0.27144501	0.28775783	0.47230668	0.48997779	0.1871428	0.26739967	0.3662871
PWY66.409	0.73510635	-0.6601179	3.01571562	-2.2297439	1.14176989	2.62180392	1.0075037	0.27164648	0.37070553
PWY.3841	-0.2536782	-0.1784016	-0.4285814	-0.2070354	-0.4910126	-0.9274433	0.14631256	0.27316159	0.371377
PWY.1269	1.13035999	0.08709958	-0.9964535	0.37682094	-0.3298314	0.174909	0.38156697	0.27529089	0.37287535
COA.PWY	-0.0875788	-0.0595471	0.11829861	0.11527828	-0.0046719	0.10088773	-0.1264731	0.28246414	0.38116908
PANTO.PWY	-0.1121357	-0.1697329	0.34384816	0.05196342	-0.0229043	0.11268265	-0.112037	0.28915167	0.38874836
P161.PWY	4.38685869	3.6241428	-1.6847934	1.47572699	3.12723721	3.2553477	1.12154185	0.29476335	0.39483061
PWY.7357	0.57394113	0.57423712	1.65971769	0.15438303	0.54420552	1.18595122	0.28944115	0.30210077	0.40317124
DAPLYSINESYN.PWY	0.71916497	0.61836218	1.87656159	0.55698807	2.25362074	4.49639326	-0.8053698	0.31009476	0.41232381
GOLPDLCAT.PWY	-2.5052119	-0.9435287	4.81832768	-1.649756	0.62804481	4.62871942	-0.9761488	0.31197051	0.413304
TEICHOICACID.PWY	0.5009874	1.68779097	2.27996544	-0.0845465	0.24670275	1.039404	0.96771413	0.31531073	0.41621016
PWY.5659	0.67655407	0.03266292	-0.3339158	0.29278902	-0.2587543	0.10770328	0.23700663	0.3206503	0.42172485
PWY.7115	4.29602481	2.76330419	5.09500992	1.99037613	2.92201334	4.21828471	1.26260639	0.32596657	0.42716919
PWY.6124	-0.0566629	-0.1725541	-0.5682168	0.04783002	-0.0854927	-0.11162	-0.1435837	0.32960174	0.43006319
PWY66.400	-0.0358414	0.16920323	0.50085694	0.05007474	0.28373572	0.84258964	-0.1282092	0.33054444	0.43006319
PWY0.1298	3.10750549	2.69637879	-0.6224581	0.78560599	1.67060254	3.80497273	1.03237299	0.33785582	0.43800594
PWY0.1061	4.28625193	2.98691549	5.18682663	1.89001315	3.5628202	3.67114707	1.26396166	0.3464835	0.44759256
PWY0.1261	2.08800807	1.99820719	6.99202221	1.28515728	1.64079181	2.07129231	1.15225573	0.354	0.45568085

GLCMANNANAUT.PWY	1.53297008	0.47945906	0.97613007	0.81129188	0.57813478	1.16451543	0.31033608	0.35568361	0.45623022
TCA	2.33301926	2.1064392	4.40778895	1.5639307	1.54945455	3.15982806	0.73828295	0.36321545	0.46287001
PWY.7392	0.02356202	1.06892166	2.81673231	-0.3908135	3.22365953	4.37442854	-0.684025	0.36341034	0.46287001
P42.PWY	1.71846224	1.61368487	3.44851819	1.35282964	1.02546649	1.67332275	0.60581822	0.36701364	0.465825
PWY.922	0.46335682	-0.3044815	3.56843662	-2.9162617	1.4346121	5.49247923	0.95723319	0.36869637	0.46633025
PWY.4041	0.69772728	0.16949396	0.32104696	-0.1704895	0.23357018	1.48748388	0.28470906	0.37440825	0.47071404
PWY.6545	0.04062009	0.69724848	0.81189751	0.18219182	0.23491686	0.16453446	0.17056957	0.37475581	0.47071404
PWY.5667	0.16265132	0.27523068	0.88328101	0.34644935	0.37985251	0.84933061	-0.1325954	0.38138674	0.47575047
PWY0.1319	0.16265132	0.27523068	0.88328101	0.34644935	0.37985251	0.84933061	-0.1325954	0.38138674	0.47575047
P162.PWY	-0.5037515	-0.0139923	1.4511508	-0.1825324	1.05009574	1.85109029	-0.5794003	0.39544432	0.49159688
PHOSLIPSYN.PWY	2.09035125	1.05428824	3.82875058	0.13327656	1.50807047	5.06527256	0.70463987	0.40031704	0.49595592
THISYN.PWY	0.68389752	0.32917749	1.13256769	0.32156363	0.39593216	0.97416329	0.18159037	0.40217091	0.49655796
PWY.5910	0.19466228	-0.330809	3.65473739	-3.0275496	1.35878506	5.63406284	0.88434532	0.40962615	0.50404845
PWY4LZ.257	3.86158232	3.53856468	-1.9289541	1.49321065	2.852109	3.19504155	0.87514146	0.41135342	0.50446382
PWY.7242	1.67086576	0.8216354	0.49573981	0.58608905	0.82284226	2.25697233	0.34287272	0.41490894	0.50711092
PYRIDNUCSYN.PWY	-0.1181778	-0.2283334	-0.5377855	-0.143488	-0.5255981	-0.5310016	0.11104989	0.44768697	0.54329552
PWY.5484	-0.0074	0.11868118	0.69085144	-0.0220362	0.38192218	0.95924993	-0.1164569	0.44831673	0.54329552
CALVIN.PWY	0.68118869	0.2539041	0.3018384	0.72044309	0.39279978	0.82030695	-0.1427764	0.44900456	0.54329552
PWY.7456	0.07827972	-0.2199605	-0.1193103	-0.2423525	-0.0356983	-1.1754953	0.24557002	0.45449123	0.54810736
PWY.5913	1.18933838	0.57460987	3.45223984	-0.4500899	1.6999481	3.1333799	0.52342382	0.46175743	0.55502631
RHAMCAT.PWY	0.70793211	0.36662524	0.03696166	0.62734987	0.56773657	1.1779665	-0.1753152	0.47155965	0.5649378
GLUCUROCAT.PWY	1.45193652	0.85949455	1.42114039	0.52844835	1.0003779	2.59007053	0.28436445	0.47589121	0.56825168
P23.PWY	3.28081924	1.5232079	7.55188204	1.834285	2.74459488	5.06060418	0.62526569	0.48936553	0.5820677
PWY.5177	0.54581635	0.62586839	2.46691015	-0.9758811	1.4031265	3.68044494	0.39390807	0.49066864	0.5820677
PWY.621	3.41102001	0.3200348	3.94105374	0.96413854	1.99662145	3.61190658	0.73072895	0.49927175	0.59034412
X1CMET2.PWY	-0.0687318	-0.1593115	-0.1621227	0.04874056	-0.129573	-0.1221601	-0.0800886	0.50234849	0.59205358
PWY.7663	0.3602921	0.14842549	-0.5262251	0.22548602	-0.1847314	0.03703034	0.11036523	0.50460279	0.5927858
CRNFORCAT.PWY	-0.2550311	2.38979917	-1.3601656	0.37940327	-0.3531378	0.76706626	0.35619519	0.51150064	0.59748816
PANTOSYN.PWY	-0.0914766	-0.0978471	0.37321076	0.06927965	-0.0214366	0.040707	-0.0725591	0.51189757	0.59748816
UDPNAGSYN.PWY	0.72438313	0.6579061	2.7962151	-0.1127288	1.20984743	2.49691358	0.29109881	0.52393886	0.60958271
PWY.241	1.91587821	0.87653203	2.2974466	1.09938834	2.3068909	5.52122254	-0.4815544	0.52755317	0.61182684
PWY.5690	1.94586047	2.04088064	4.06772962	1.58427928	1.52484403	3.19219728	0.46362233	0.54016223	0.62445506
PWY.7237	-0.6232769	0.49158763	0.94045237	0.32073306	-0.1918459	1.63467849	-0.3619565	0.5480391	0.63154982
PWY.7234	0.33293865	-0.0516765	-1.1717048	0.09300169	0.15747783	0.23593318	-0.1206267	0.55903838	0.64218649
PWY4FS.7	1.49713326	1.2557428	5.23690742	-0.3611207	2.39441361	5.95651076	0.49787141	0.57018406	0.64969526
PWY4FS.8	1.49713326	1.2557428	5.23690742	-0.3611207	2.39441361	5.95651076	0.49787141	0.57018406	0.64969526
P108.PWY	0.34712633	0.22620731	-1.4937806	-0.1039633	-0.4447359	0.5152697	0.21303755	0.57241817	0.64969526
PWY.6126	0.11343173	0.1809001	0.34816687	0.19360127	0.2442914	0.32257021	-0.0610607	0.57391287	0.64969526
NONOXIPENT.PWY	1.22699127	0.44661048	0.99628668	1.07109994	0.91502727	1.60527977	-0.1677763	0.57452391	0.64969526
ARGORNPROST.PWY	-0.1148966	1.09438463	3.46375409	0.82684049	1.73948112	1.60869629	-0.4767227	0.58110256	0.65509388
P164.PWY	0.718684	0.84690868	0.80132455	0.88876447	0.75026727	1.99960158	-0.2150125	0.58825311	0.6611018
PWY.5384	3.06417185	0.93239044	6.04117979	0.75297059	2.53605222	6.01128325	0.64574296	0.60114671	0.67350696
PWY.7117	1.93416866	0.96128435	2.40525762	1.07615567	2.29480363	5.35582381	-0.3905015	0.60640464	0.67730734
COLANSYN.PWY	0.77514088	0.09987304	1.05155244	0.06744294	0.47970941	1.75500421	0.14558133	0.62412106	0.69495689
PWY.7323	0.79616732	-0.0553726	0.76144398	0.07000204	0.31445659	1.5449377	0.14642845	0.62836814	0.69682923
PWY.6507	1.77741274	0.02474705	0.50196053	0.44180705	0.76075279	2.27276596	0.21343446	0.62964184	0.69682923
PWY.7221	-0.0358638	0.00472707	0.14094405	0.05261166	0.02257634	0.12778519	-0.0524517	0.64465068	0.7105292

GALACT.GLUCUROCAT.PWY	1.18445792	0.61633498	1.22158669	0.47250395	0.80595397	2.29908505	0.16604548	0.64593563	0.7105292
PWY.6305	1.93921608	-0.2191219	-0.2987607	0.81592792	-0.0251333	1.44259866	0.27798211	0.65354991	0.71673298
GLUDEG.I.PWY	0.06769459	0.35955729	1.31749999	-1.6210859	1.31052899	3.78866671	0.27358709	0.67247645	0.73526793
PPGPPMET.PWY	0.09042913	-0.716413	-1.8628313	-0.2396907	-1.2409166	-0.4369494	0.15913773	0.68103551	0.73978692
PWY.7383	0.66259825	-0.6554518	-0.7789355	-0.2911713	-0.1831913	0.23713797	0.20544068	0.68212526	0.73978692
GLYCOGENSYNTH.PWY	0.1956483	0.51774146	1.40338254	0.15888987	0.68475936	1.79919894	-0.0889882	0.68272346	0.73978692
PWY.6936	1.60075421	1.25972763	2.51897592	0.44239856	1.95818965	3.56670274	0.24275511	0.68914269	0.74452023
P124.PWY	-1.8219444	-1.3291689	2.31252476	-2.4068169	0.17826023	3.59817088	-0.3701388	0.69273226	0.74617748
PWY.7371	0.08647361	-0.0995334	-0.3944625	0.55722698	-0.5918748	0	-0.1456131	0.70577879	0.75798136
PWY.7282	2.76022029	2.12216782	4.06315667	2.86920683	3.68092277	3.0759586	-0.4686007	0.71466911	0.76526515
PWY66.367	0.20751885	0.78911972	0.01698545	-0.5539062	0.79076361	1.59966782	0.2203224	0.72073572	0.76949137
ANAGLYCOLYSIS.PWY	-0.0276804	0.06210355	0.47227376	-0.0542886	0.19679182	0.54316589	-0.0411074	0.72630145	0.77303907
PENTOSE.P.PWY	1.2792789	0.64408203	2.12416148	-0.2795429	1.70011474	3.95662314	0.2319109	0.72831781	0.77303907
P122.PWY	2.55644257	2.22275034	-0.715457	0.84288269	1.70331992	4.57219067	0.42241743	0.74814159	0.79176501
PWY.5941	0.08477817	1.07268022	-0.1708626	0.89867212	0.06461287	0.94156342	-0.2274597	0.75190509	0.79343474
PWY.6969	1.31703148	1.60312327	4.0860414	1.28928385	1.26983087	3.13345203	0.23264653	0.75489541	0.79428125
PWY.6892	1.25618872	0.60270532	0.7028422	0.8980577	0.58692423	1.46640605	0.08622168	0.78190234	0.82031951
LACTOSECAT.PWY	1.63179785	1.64379111	4.89280842	0.51967771	2.21952525	6.11618112	0.2236714	0.79499245	0.83164916
PWY66.399	0.393354	-1.1106145	-0.3729697	-0.2995157	-0.0939131	0.63306032	-0.1236572	0.80857355	0.84342586
PWY.4321	-1.7457547	-1.1028684	2.12134006	-3.102649	0.68612424	4.75622284	-0.215887	0.81311457	0.84573234
PYRIDOXSYN.PWY	3.52318541	2.01268232	4.10521715	2.80157369	3.57124416	4.45005055	-0.2109447	0.83465395	0.86565539
PWY0.845	2.78132724	2.02225775	3.93230706	2.59581303	3.31695114	2.86131451	-0.2136046	0.85078566	0.87987235
PWY.3781	1.41091736	-0.1628	0	1.01483755	0.34400026	-0.7833591	0.12186882	0.86479468	0.89181951
GALACTUROCAT.PWY	0.80823412	-0.2170314	-0.3725765	0.18138187	0.12283514	0.89897431	0.04392795	0.88205392	0.90704128
FERMENTATION.PWY	0.94716861	0.46768494	2.88980119	-0.019832	1.90853286	3.69446729	-0.0848509	0.900691	0.92358993
OANTIGEN.PWY	0.35244437	0.4638424	2.06013842	-0.016726	1.07293922	2.27481334	-0.040322	0.91282993	0.93340075
PWY.6590	-0.1561469	0.23275581	1.22823067	-1.1278409	1.04410903	3.41470436	-0.0296473	0.96049629	0.9771767
NAGLIPASYN.PWY	1.50138651	-0.0395457	0.90448444	1.57451674	-0.7532077	1.73938547	0.04951405	0.96102502	0.9771767
PWY.5723	3.47597484	-1.6565032	4.81332064	1.27838394	1.99841374	4.01631705	-0.0520967	0.96916896	0.98270484
PWY.6901	0.847795	0.4392057	1.81280758	-0.4026297	1.66350419	3.94278595	-0.0242015	0.97217094	0.98300293
PWY0.1241	2.68946468	0.12161553	2.88212305	1.69038095	1.11287589	4.20508752	-0.0277386	0.9793741	0.98753555
PWY.5973	0.20596022	0.04496616	-0.8738323	0.16272055	-0.2151973	-0.0260417	0.00257796	0.98643378	0.99119946
P185.PWY	0.01920335	-1.2543463	-1.0836345	0.19738676	-1.2415204	-2.1000355	0.00909903	0.98846889	0.99119946
CENTFERM.PWY	-0.1330723	0.22565223	1.22458393	-1.138123	1.01834279	3.39276327	-0.0036464	0.99500652	0.99500652

Supplementary Table 7. Linear model result for ECs. A positive coefficient indicates BDL mice > ShamOP mice, negative indicates the opposite. P-values and FDR-adjusted P-values are shown.

feature	mean normalized abundance BDL day1	mean normalized abundance BDL day3	mean normalized abundance BDL day7	mean normalized abundance shamOP day1	mean normalized abundance shamOP day3	mean normalized abundance shamOP day7	coef	P-value	FDR
EC_3.2.1.89	-1.7916189	-1.0736678	0.39555765	0.22505441	0.55969869	1.80384261	-1.8113778	7.43E-09	6.95E-06
EC_3.5.1.44	-1.4567886	-0.1444726	-0.0569534	0.67435635	0.91618663	2.35584713	-1.7948827	8.65E-09	6.95E-06
EC_2.6.1.1	1.15226367	0.58897503	0.4641598	-0.8096291	-1.189338	-2.8767144	2.07402234	1.89E-08	7.02E-06
EC_1.1.1.40	0.37489689	0.2436872	0.85798325	-0.8125845	-0.7756462	-1.2806079	1.249573	1.90E-08	7.02E-06
EC_3.2.1.17	0.30928725	0.02902991	0.74640558	-0.9725532	-1.0318868	-1.9084752	1.37852382	2.22E-08	7.02E-06
EC_4.1.1.8	0.28735312	0.21257614	0.48934965	-1.6917057	-1.2038235	-2.5624538	1.93171471	2.67E-08	7.02E-06
EC_4.2.1.126	1.65319559	1.18425312	1.09241627	0.17072741	-0.4087828	-0.3009271	1.49960036	3.06E-08	7.02E-06
EC_4.1.1.15	0.38546338	0.36427465	0.09222252	-1.3287274	-1.3576204	-3.6426331	1.98411338	5.12E-08	1.03E-05
EC_2.1.1.174	10.5127508	6.07370679	8.15917378	0.1054959	1.33587818	2.06580839	7.88558065	6.24E-08	1.11E-05
EC_1.6.5.5	11.878019	7.77443919	9.77892245	-0.0176	2.22519221	1.43915502	9.25812459	9.31E-08	1.41E-05
EC_3.4.17.14	-3.8352142	0.91297674	3.91493477	4.79570036	3.92040238	5.64503204	-5.8237328	9.64E-08	1.41E-05
EC_4.2.1.45	-3.8067632	-1.2395662	-0.3138183	1.01530604	2.10449442	4.85361159	-4.345893	2.03E-07	2.72E-05
EC_1.11.1.6	0.76906434	0.03881393	0.64106306	-0.418746	-0.7214609	-1.4045153	1.14219215	2.54E-07	3.14E-05
EC_3.6.3.33	10.4830074	3.57172748	4.83181538	0.13239143	0.84220493	2.74937533	6.63354762	2.81E-07	3.23E-05
EC_4.2.1.2	0.52784135	0.12003698	0.37062892	-0.28532	-0.5453994	-1.018163	0.83097506	3.02E-07	3.24E-05
EC_2.7.11.5	8.2686159	5.42289722	8.04112912	-0.2904126	0.92206451	1.01395375	6.95431736	3.49E-07	3.51E-05
EC_3.1.1.5	10.8246445	5.65001045	7.29165465	1.2176584	1.44978706	2.01932272	7.15970434	4.52E-07	4.27E-05
EC_2.7.4.6	1.0517988	0.39884055	0.986638	-0.3319483	-0.6390767	-1.9875274	1.46262415	5.59E-07	4.45E-05
EC_3.1.1.61	-0.9478318	0.00472763	0.15814141	0.77594309	1.01151144	2.3693494	-1.5373342	5.60E-07	4.45E-05
EC_1.1.1.218	-3.3640706	-0.5960436	1.52507443	2.58890131	3.30479291	7.40858675	-5.2410194	5.67E-07	4.45E-05
EC_4.1.3.36	0.73516507	0.1617321	1.08725237	-0.2880776	-0.423167	-0.9197138	0.9905383	5.81E-07	4.45E-05
EC_1.2.4.2	8.79142034	5.98910711	7.94706015	-0.2071993	-1.5553063	-0.12438	8.33371484	6.59E-07	4.81E-05
EC_1.6.5.11	0.73034435	0.03738386	0.99450862	-0.5237993	-0.7654907	-1.9213636	1.30387351	8.60E-07	6.00E-05
EC_4.1.2.50	0.54437753	0.13104407	0.17573207	-0.3125921	-0.5986303	-1.4990294	0.91499456	1.04E-06	6.95E-05
EC_1.2.1.8	9.53826195	6.81957942	8.10675614	-0.087235	1.38085578	1.68736622	7.77263578	1.34E-06	8.60E-05
EC_6.1.1.23	-1.3797786	-0.159981	0.52612639	0.73928748	1.13731199	2.37079347	-1.7981973	1.40E-06	8.64E-05
EC_3.2.2.21	10.0611945	5.06204503	7.2024459	1.79376478	0.7834883	1.92614032	6.46022658	1.61E-06	8.69E-05
EC_2.7.7.27	-0.6601416	0.166266	0.12308895	0.76767032	0.91309611	2.39627992	-1.3006777	1.61E-06	8.69E-05
EC_2.3.1.35	-1.1401482	-0.075333	0.25641046	0.83925276	1.31474798	2.45118387	-1.7986875	1.62E-06	8.69E-05
EC_4.2.1.130	10.3465102	5.31542014	7.07773689	0.53016079	1.97318788	2.99434146	6.83689888	1.62E-06	8.69E-05
EC_3.4.24.78	-1.9695253	-0.3652733	0.10897823	0.47449948	0.86691033	2.25835638	-1.9839957	1.73E-06	8.95E-05
EC_6.3.2.3	11.3785964	6.23679037	5.25489673	0.93555324	1.57575865	2.96585134	7.39468413	1.83E-06	9.17E-05
EC_4.1.1.70	-0.6608408	0.03886418	-0.1232329	0.83808279	0.92234193	2.52703165	-1.4346408	1.99E-06	9.26E-05
EC_1.4.99.5	-4.3070321	-2.2173084	-2.5195471	2.4768251	2.18654054	7.78675319	-6.4267838	2.00E-06	9.26E-05
EC_2.8.1.10	0.36973732	-0.0073795	0.51987825	-0.2402223	-0.5821264	-1.0271155	0.71024303	2.02E-06	9.26E-05
EC_2.4.2.14	-0.1216182	-0.0725644	-0.1736828	0.27505012	0.17919375	0.44898739	-0.3770878	2.44E-06	0.00010899
EC_1.7.99.1	0.19875937	-0.009798	-0.0643703	-0.6901698	-1.0823747	-1.9292	1.07413751	2.51E-06	0.00010904
EC_4.3.3.6	-0.8648658	-0.7155722	-0.0401569	0.83531068	1.12319002	2.20481414	-1.8204724	2.81E-06	0.00011894
EC_3.5.2.6	0.35913152	0.03928893	0.5398601	-0.5946465	-0.7416194	-1.9976666	1.09509814	3.55E-06	0.00014609
EC_6.3.4.3	-0.4337427	-0.070646	-0.0745961	0.71930648	1.1701302	2.27508085	-1.3325716	4.03E-06	0.00016181
EC_3.1.3.2	0.42988032	0.04839732	0.26939613	-0.511705	-1.0269354	-2.5921747	1.22897417	4.23E-06	0.00016585
EC_2.10.1.1	10.2459326	4.27470791	7.47304753	0.7025495	0.69183782	0.70557961	7.09162802	4.50E-06	0.00017209
EC_1.3.99.24	-2.7130932	-0.9599639	0.82450343	1.18921132	1.90559132	3.99563752	-3.4512336	5.05E-06	0.00018431
EC_1.8.1.2	9.39324235	6.00185277	8.36030054	-0.0880397	0.15745429	-0.499875	8.12704002	5.05E-06	0.00018431
EC_1.1.1.395	-2.6320959	-0.74147	0.65284063	1.77940523	3.6922771	9.12588218	-4.9312884	5.36E-06	0.00019117
EC_1.4.3.16	0.22946674	-0.1435143	-0.2605905	-0.5388823	-1.0060331	-2.0299436	0.92517968	5.56E-06	0.0001942
EC_1.3.3.4	5.24515929	6.58317719	0	-0.3237531	-1.8800034	-2.8655854	6.24407462	6.07E-06	0.00020699
EC_2.6.1.77	-2.1908478	-3.380962	-5.1757088	1.92337331	1.60917191	3.76301576	-5.0401871	6.19E-06	0.00020699

EC_1.8.5.3	8.85987305	6.53124947	8.45144868	-0.1043068	2.06322265	3.12314108	6.95472045	6.60E-06	0.00021628
EC_6.2.1.30	-0.3135143	0.3241252	0.26950916	0.82295644	1.26558444	2.4594287	-1.1992214	7.84E-06	0.00025192
EC_1.1.1.154	9.6715287	4.92664491	6.84399472	1.03136287	1.97457769	0.51903867	6.37097551	8.25E-06	0.00025553
EC_4.2.1.12	7.62143264	4.40168318	4.75270014	0.48873963	0.800533	0.6970054	5.51105833	8.27E-06	0.00025553
EC_1.14.13.107	9.82414736	2.72796999	4.58197018	-0.0514549	-0.1481764	-0.8826976	6.86050664	8.57E-06	0.00025972
EC_4.1.99.3	10.7466637	4.59694165	4.99110463	0.7879611	1.3832543	1.74799518	6.75375114	9.73E-06	0.00028944
EC_1.4.1.1	0.22478051	0.16219404	-0.4297257	-0.5408328	-1.3344242	-2.844595	1.22600418	1.03E-05	0.00029987
EC_3.5.1.9	-3.7726569	-1.1730094	2.09737204	2.81454351	2.22098936	6.60713982	-5.2504075	1.06E-05	0.00030347
EC_4.2.99.20	10.4810657	3.53984465	4.8696945	0.09500373	1.35374662	2.94351541	6.452869	1.14E-05	0.00032078
EC_2.4.1.25	-0.5230112	0.11574465	0.47357896	0.95317265	1.55879535	2.54471994	-1.5353948	1.17E-05	0.00032208
EC_6.2.1.17	7.57830558	6.02042702	7.40255607	0.14150815	1.01245621	0.82298845	6.48837288	1.18E-05	0.00032208
EC_1.15.1.1	0.8471707	0.15163902	0.62353339	-0.2210011	-0.5541152	-0.8719575	0.9891245	1.33E-05	0.00035438
EC_2.4.1.12	7.74645717	4.73653658	11.0081962	-0.2754842	0.7762365	2.03774485	6.70694744	1.35E-05	0.00035438
EC_2.8.1.1	9.18672184	3.99887263	3.41715941	1.30028409	-0.8597627	2.10565112	5.94424734	1.71E-05	0.00044097
EC_1.1.1.405	-2.5942883	-4.2899853	-0.2636084	1.42438779	2.23543774	6.5297745	-5.2700808	1.73E-05	0.00044097
EC_2.7.8.31	-1.1358338	0.40721211	0.11756115	0.72006947	0.84623177	2.20359196	-1.391938	1.87E-05	0.00046878
EC_3.1.2.14	9.62214255	2.82702357	4.47110659	0.44307123	1.15650146	1.74755055	5.73564084	1.91E-05	0.00047128
EC_2.7.1.17	-0.5753787	0.33723339	0.59500755	0.8477496	1.23600981	2.79759444	-1.339822	1.96E-05	0.00047305
EC_2.1.1.186	6.84885285	4.50760047	7.03527146	0.40789802	0.76686148	0.75026789	5.47186489	1.99E-05	0.00047305
EC_2.1.1.35	6.61041665	3.50360597	7.41135408	0.02344015	0.75398764	0.93020686	5.22607971	2.00E-05	0.00047305
EC_4.1.1.82	-2.6398521	-0.9757681	0.27471152	1.02411221	2.37268949	5.63626255	-3.7652462	2.08E-05	0.00048482
EC_2.3.1.200	8.09121102	1.74904119	2.80719125	0.37628059	0.38641245	0	4.86698632	2.13E-05	0.00048777
EC_2.1.3.3	-0.0864727	0.10056818	0.34149587	0.8563834	1.11180586	2.23583486	-1.0890733	2.17E-05	0.00048962
EC_1.3.1.54	-1.4532775	-1.2682347	1.72728087	2.28424578	2.69356506	8.68942459	-4.2575845	2.21E-05	0.00048962
EC_2.3.1.40	7.09622942	5.47693799	7.52851345	0.0924456	0.79765916	1.94178447	6.00909571	2.23E-05	0.00048962
EC_3.2.1.28	8.5912405	5.8108196	8.04577082	-0.539948	1.09535916	4.50210234	6.88110017	2.26E-05	0.00048962
EC_1.1.99.1	8.46888334	4.71057829	7.38113752	-0.0592369	0.71349825	1.50592926	6.60467035	2.56E-05	0.00054834
EC_2.5.1.18	8.36833562	6.35194525	7.4831102	0.2486023	2.1053263	3.31469427	6.28553064	2.65E-05	0.00055918
EC_3.2.1.67	-2.4166494	0.55544557	2.07574297	3.17855124	2.98118831	7.41966567	-4.4841559	2.72E-05	0.00056778
EC_5.99.1.2	-0.0330611	0.03102198	0.19525788	0.47477306	0.47703206	1.11106045	-0.5414587	2.91E-05	0.00060005
EC_7.1.3.1	-1.2518945	0.89246698	-2.9150777	4.10317765	2.69502594	7.6489496	-4.803344	3.04E-05	0.00061806
EC_1.18.1.3	7.77163898	4.50007286	7.27736678	0.35656087	0.74036614	1.96165536	5.85905647	3.28E-05	0.00065868
EC_1.7.2.3	8.42652264	6.44716914	8.40041987	0.46610342	2.00366047	2.14169932	6.53190916	3.44E-05	0.00068175
EC_2.3.1.122	-3.2429381	-1.2721553	0.64805464	0.92798191	1.86031898	3.98390747	-3.7030213	3.54E-05	0.00069305
EC_5.3.3.3	-4.646488	-0.1997664	-1.2173411	2.8127654	1.59889348	5.17151278	-5.3756851	3.84E-05	0.00074231
EC_1.5.1.30	10.1898758	2.09547801	4.52763282	0.79734051	1.080266	2.96162446	5.45448326	3.92E-05	0.00074896
EC_2.6.99.2	0.37248076	-0.1038434	-0.140573	-0.3973459	-0.760024	-1.3638278	0.78408296	3.96E-05	0.00074912
EC_2.1.1.148	-1.1135806	-0.1148279	0.35745203	0.5141107	0.80761066	2.46313031	-1.4458446	4.13E-05	0.00076296
EC_3.5.4.5	-0.2921502	-0.1282228	-0.4791812	0.53512059	0.59591556	1.49114365	-0.9376248	4.27E-05	0.00076296
EC_4.99.1.4	-1.8024186	-2.035838	0.3558117	1.19866777	1.5699272	3.45306402	-3.2370503	4.31E-05	0.00076296
EC_1.3.5.3	7.42735073	4.32752872	6.75942421	0.03313573	1.3076239	1.87146886	5.5550557	4.32E-05	0.00076296
EC_3.2.1.24	-1.2136071	-2.7931168	-3.3132446	3.88865277	3.52836508	0.75835567	-5.3898148	4.32E-05	0.00076296
EC_3.4.19.11	-4.7629728	-0.4975236	3.56006369	3.8937486	5.49550387	11.2791451	-7.6209393	4.32E-05	0.00076296
EC_1.12.1.2	-2.5092224	-1.6165306	1.03300356	2.27277708	2.88591998	7.73524583	-4.9501063	4.42E-05	0.00077079
EC_1.13.11.2	7.76673193	2.27734173	2.87129078	0.20291556	0.12386636	-0.5434931	5.14340616	4.57E-05	0.0007889
EC_1.12.1.3	-1.7969652	0.89686631	0.44425837	5.19327713	2.87315855	8.82510631	-5.477611	4.63E-05	0.00079027
EC_1.3.1.34	8.15085258	6.3766865	8.13755913	0.43282829	2.61063328	0.76689504	6.33001466	4.68E-05	0.00079164
EC_3.1.1.32	7.61619269	4.7265676	6.97984596	0.01725186	1.34121451	2.85735657	5.69031421	4.75E-05	0.00079535
EC_1.1.5.4	9.42045194	3.86070228	5.14281886	0.0111985	1.27030327	0.49473155	6.43933375	4.85E-05	0.00080311
EC_2.4.1.21	-0.0742565	0.14316102	0.08373899	0.74988239	0.99461511	2.23677079	-1.0032915	4.99E-05	0.00081007
EC_2.1.3.6	7.32722571	2.54524785	5.08385946	-0.4771598	-0.3615735	0.10933113	5.7207214	4.99E-05	0.00081007
EC_6.3.5.11	-1.4123027	-0.290298	0.93686234	0.40946103	0.4243865	2.17635713	-1.3733682	5.13E-05	0.00082361
EC_2.4.1.1	-0.142649	0.20811474	0.3049846	0.90513675	1.16081673	2.4247308	-1.1520921	5.38E-05	0.00085474

EC_2.3.1.41	7.43292448	4.00990665	4.96673187	1.08296815	-1.8127535	-3.8235813	6.42914979	5.54E-05	0.00086093
EC_4.1.2.13	0.44437653	0.44485568	-0.0900628	0.23200229	-0.2786274	-0.3658046	0.39298615	5.57E-05	0.00086093
EC_5.3.3.6	7.40777726	3.62848215	6.95824353	-0.0417491	0.64057049	0.48476855	5.76292292	5.58E-05	0.00086093
EC_2.3.3.9	8.29292982	6.74226364	7.41085346	0.50329883	1.76720975	2.18076521	6.49752985	5.90E-05	0.00090262
EC_3.5.1.19	7.05258848	4.34100008	4.72548248	-1.3666996	-0.9204925	1.72329026	6.62148597	5.97E-05	0.00090473
EC_1.13.11.16	8.00725807	4.35975233	4.8571812	-0.2923558	0.92806958	2.98013446	5.79160825	6.14E-05	0.00092131
EC_1.1.3.21	7.58448134	1.65004393	2.6437106	0.34175454	0.31992956	0	4.58721647	6.28E-05	0.00093442
EC_1.14.14.5	8.4580937	4.50935489	7.30517837	1.04383077	1.46778352	1.96196815	5.61471589	6.45E-05	0.00095055
EC_1.1.1.262	1.2253684	1.08251717	0.63150784	0.11633482	-0.355166	-1.3492311	1.33273117	6.63E-05	0.00096742
EC_2.8.1.2	7.77150677	4.49646878	7.03665325	0.45801	1.39721429	2.02133225	5.55013016	6.72E-05	0.0009683
EC_2.6.1.11	-0.3397696	0.23962161	0.55406979	0.89112976	1.30419097	2.28393085	-1.2344884	6.75E-05	0.0009683
EC_4.1.1.17	8.48945117	5.92697517	7.972897	0.81759841	1.89836781	2.24671152	6.15883901	6.90E-05	0.00098013
EC_1.2.1.22	8.24180782	4.95778427	9.53435441	0.90524165	2.14835239	2.02244878	5.77744944	7.07E-05	0.00099645
EC_7.3.2.1	-0.3974363	0.07551909	0.42259166	0.57387483	1.19999989	2.16095595	-1.1170253	7.31E-05	0.00100839
EC_3.4.11.19	7.50382267	1.68431853	2.91185968	0.35352456	0.34982556	0	4.57385177	7.41E-05	0.00100839
EC_1.14.12.17	7.74304096	5.26935523	5.02397045	0.15806431	0.9696838	1.95279838	5.8736914	7.44E-05	0.00100839
EC_3.6.3.2	7.29776562	4.8674075	7.61807812	0.45407138	0.3530858	2.02021194	5.84954709	7.49E-05	0.00100839
EC_3.4.11.4	-0.0277814	-0.1910261	-0.0065233	-0.4209255	-0.6443296	-1.1623416	0.50855556	7.50E-05	0.00100839
EC_2.5.1.141	8.92248395	3.63313543	4.7898583	0.81097133	1.18544616	0.55836974	5.64188985	7.63E-05	0.00100839
EC_1.11.1.5	7.75808394	5.18418911	6.84996483	1.04787321	1.37587658	1.90716299	5.46682704	7.65E-05	0.00100839
EC_1.7.1.6	9.26872366	1.0630295	4.96205417	0.76579342	0.95508426	2.31823934	4.8026637	7.66E-05	0.00100839
EC_2.7.6.2	-1.1476112	-0.5217061	-0.3014997	0.19392834	1.08301893	3.11313002	-1.6910611	7.90E-05	0.0010199
EC_2.7.8.12	7.11109249	2.86682857	0.94677032	1.04778455	-3.0278824	-2.9286737	5.66309579	7.95E-05	0.0010199
EC_1.16.1.3	7.72805997	4.74114405	7.31070724	0.52209129	1.50322577	2.06803053	5.5721041	7.98E-05	0.0010199
EC_1.1.1.83	7.31741274	3.56352298	4.8497154	0.46426613	1.3001646	0.66504393	4.920604	8.00E-05	0.0010199
EC_4.4.1.15	6.62175474	5.85601744	6.77728621	0.46038107	1.870252	1.78418633	5.27305846	8.27E-05	0.00104518
EC_4.1.3.4	-3.3188107	-1.2804817	-0.1294264	1.4235253	1.80524885	4.929892	-4.2151201	8.51E-05	0.00106097
EC_4.2.1.11	0.07496312	-0.0724083	-0.0033129	-0.2107189	-0.3178527	-0.7690848	0.33235063	8.56E-05	0.00106097
EC_4.2.2.n1	7.89451414	4.25386196	7.18332082	0.58101264	1.53412175	1.92800147	5.44738714	8.59E-05	0.00106097
EC_4.4.1.16	8.5192491	2.49988076	6.54352639	0.43989688	1.15852464	1.69361828	5.30484311	8.66E-05	0.00106129
EC_1.4.1.16	-0.2054604	-0.1947244	0.08429342	0.55388887	0.44601841	1.03056743	-0.7472661	8.87E-05	0.00107951
EC_1.1.1.261	-1.8270626	-0.6529061	-0.0907492	0.90765614	1.80109158	2.78436889	-2.6454505	9.23E-05	0.00111481
EC_3.2.2.4	8.14057982	6.78539243	5.40039002	2.63905636	0.23786086	-0.3269347	5.86254253	9.49E-05	0.00113745
EC_4.1.1.25	7.67661385	1.70502366	2.73562409	0.71514024	0.86726448	0	4.28477778	0.00010269	0.00122165
EC_1.14.14.9	7.4479976	5.26663158	7.2321381	0.77485943	1.37895782	1.98707192	5.51675446	0.00010477	0.00123717
EC_2.7.1.73	7.50017287	4.58389692	7.41094264	0.13926575	1.53995592	1.9779277	5.61626434	0.00010916	0.00127966
EC_3.1.2.20	7.71241236	0.95746933	2.94353681	0.23203299	0.17348275	-1.471643	4.74622425	0.00011352	0.00132108
EC_1.18.6.1	-4.01241	-3.4833547	0.69937988	2.4088952	2.30141857	3.81549633	-5.8058975	0.00012125	0.00138894
EC_2.4.1.157	8.2610061	1.02193339	2.9743118	0.36165305	0.35504442	1.12670485	4.58899151	0.00012173	0.00138894
EC_2.7.1.168	-2.1456309	-2.490501	0	1.8765975	1.32007274	6.72087545	-4.3351347	0.00012194	0.00138894
EC_4.2.1.81	6.28499717	3.58081924	6.30992343	0.35167902	1.14837051	1.6347236	4.5488136	0.00012339	0.00139549
EC_6.2.1.20	6.80890502	3.98914829	6.76639743	0.43056574	1.23625218	1.74146747	4.93802358	0.00012477	0.00140131
EC_1.4.4.2	0.36748042	-0.1725985	0.24506058	-0.254294	-0.6281122	-0.9949027	0.63531555	0.00012693	0.00141561
EC_3.6.1.26	7.60511429	4.57770299	7.02544651	0.39659406	1.5046877	1.90751126	5.50338773	0.00012866	0.001425
EC_1.4.99.6	7.37051875	4.40980829	6.91213108	0.79209102	1.82763928	1.84391886	4.99586725	0.00013097	0.00143104
EC_2.7.7.33	-0.4558343	0.47103542	0.00922551	1.02991207	1.38375845	3.04753902	-1.4814577	0.00013099	0.00143104
EC_2.7.11.1	-0.2397512	0.33817279	0.39585035	0.90201898	0.89318473	2.1402304	-1.0188889	0.00013215	0.00143402
EC_2.3.1.169	-2.1222229	0.15225322	-0.0971407	2.05240713	3.03769073	7.90360412	-4.2123387	0.00013409	0.0014369
EC_3.1.11.1	7.50878648	5.3656723	7.47162366	0.80673017	1.85377921	2.95457322	5.31300079	0.00013421	0.0014369
EC_2.8.1.12	7.37501506	3.63459224	4.71738066	-0.0418955	0.67704702	0.49394398	5.46020237	0.00013668	0.00145374
EC_2.4.2.7	0.02538662	0.03307127	0.3077037	-0.3729229	-0.2141464	-0.9451542	0.45872538	0.00013863	0.00146475
EC_3.4.11.7	8.96406976	1.68474394	3.19411284	0.76798513	0.96890421	2.08982696	4.67105986	0.00014712	0.00154431

EC_3.1.3.15	0.94463177	0.6948501	0.86293429	-0.2610681	-0.5718988	-1.381383	1.3562704	0.00014904	0.00155426
EC_2.3.1.15	9.07120386	5.36098518	8.37833588	0.92168226	1.69372033	4.58535141	6.01612587	0.00015114	0.00156596
EC_2.6.1.13	7.10153743	4.27106987	6.80584938	0.21733087	0.52145783	-0.8306843	5.88333255	0.00015429	0.00157141
EC_2.4.1.15	7.56176546	4.86018606	4.73798635	0.47080836	1.88649526	1.83531964	5.14454899	0.00015431	0.00157141
EC_1.1.1.301	-2.8311964	0.85644068	2.70345644	2.15609047	4.09884457	9.45046281	-4.5977876	0.0001546	0.00157141
EC_3.4.11.23	7.70338966	4.77720958	7.92967157	0.49239931	1.46696279	3.1668003	5.53147558	0.00015678	0.00158357
EC_2.3.1.1	-0.2583943	0.33803371	0.6941613	0.98016783	1.4016241	2.46330157	-1.2435366	0.00015838	0.0015897
EC_5.4.99.19	7.99486717	5.53467378	7.29625288	1.27999852	2.03816612	2.78763298	5.31123659	0.00016237	0.00159949
EC_4.2.1.8	-0.1107168	-0.0484602	0.26615436	0.39317532	0.59209184	1.30134574	-0.6201471	0.00016306	0.00159949
EC_4.99.1.12	-3.1548709	-1.8093642	0.07146782	-0.1401629	0.62777565	1.72210458	-2.6390722	0.00016332	0.00159949
EC_4.2.3.3	0.09956685	0.0277	-0.3003308	-0.0863385	-0.5470714	-0.9483249	0.37571754	0.00016334	0.00159949
EC_2.7.4.23	8.26727304	4.27166782	7.81991761	0.475279	2.06232875	2.11355539	5.58636963	0.00016707	0.00162618
EC_4.1.99.11	-4.7339018	-1.17796	2.52950051	1.72924805	1.89970263	6.70374617	-5.0225973	0.00016917	0.0016367
EC_4.2.1.79	7.47209024	4.64474164	7.99472505	0.48078292	1.59009821	1.96514648	5.49522976	0.0001724	0.00165793
EC_2.1.1.130	-2.8430275	-1.3307757	-0.3369503	1.6102131	1.1643034	6.90614437	-4.1597643	0.00017968	0.00171763
EC_2.6.1.81	7.94372644	5.19667468	7.73829566	0.86093902	2.52502194	2.98192068	5.26039727	0.0001853	0.00175301
EC_2.3.1.189	-3.8832984	-2.770818	-0.7153865	0.32998488	1.72187301	4.72163109	-4.4641325	0.00018622	0.00175301
EC_1.2.1.71	6.92111335	4.74688352	4.3492672	0.77367618	1.17100167	2.7194778	4.67626957	0.00018665	0.00175301
EC_2.4.1.58	7.9572515	4.82654254	5.21686119	0.44880392	0.98108615	1.94879404	5.69213594	0.0001934	0.00180582
EC_3.2.1.172	-1.5504157	-0.5518801	0.49196704	0.88675667	1.79769249	3.30132858	-2.4489726	0.00020426	0.00188073
EC_1.2.99.2	-2.3970227	-1.0683146	0.25083642	0.41156623	1.06071825	2.17453783	-2.457815	0.00020552	0.00188073
EC_3.6.1.45	7.4269649	4.20334203	7.38007061	0.83192025	1.88854091	1.81743022	4.97159648	0.00020585	0.00188073
EC_3.5.4.23	8.14657955	0.98132558	2.81965173	0.36498265	0.89226686	1.13191857	4.31714099	0.00020611	0.00188073
EC_3.1.3.26	8.382405	4.81516538	7.71331627	1.33628081	1.52016415	2.10739934	5.53903202	0.0002261	0.00205151
EC_3.4.23.51	7.36337221	3.65850399	7.57832061	0.03541853	1.44917361	2.03940151	5.31527787	0.00022803	0.00205738
EC_1.6.5.2	7.71313556	5.68666248	7.37110362	1.14622785	1.50996728	2.10665074	5.56180418	0.00023305	0.00208259
EC_2.1.1.220	-1.9271948	-2.8977706	-3.3547085	1.22409926	2.38350384	3.73197023	-4.3795734	0.00023342	0.00208259
EC_2.3.1.81	-2.0132609	-0.5515933	1.15562019	0.25784563	1.01756728	2.79412325	-1.9471954	0.00023931	0.0021139
EC_4.1.1.68	7.19957926	3.57288079	7.62227517	0.45139659	1.38120208	1.86332534	5.02510054	0.00023993	0.0021139
EC_3.4.21.116	-2.4823453	-0.7904928	-0.314438	0.4062853	0.67029992	1.99129036	-2.3183986	0.00024087	0.0021139
EC_6.3.1.20	3.00006318	1.1055274	2.96211829	-0.3547259	0.23183843	0.90719585	2.32102783	0.00025025	0.00218025
EC_4.1.3.1	7.04138096	4.55248176	7.57458446	0.45639317	1.46306676	3.04903503	5.09818514	0.00025196	0.00218025
EC_5.4.99.21	7.75110058	4.94259464	7.24923508	0.78327695	2.00161784	2.03218277	5.34802459	0.00025305	0.00218025
EC_2.1.4.2	7.71529788	1.04276916	2.8056274	0.72249419	0.89110962	0	4.06723287	0.00025387	0.00218025
EC_3.1.1.4	7.17219977	3.38986214	6.96885034	0.36323148	1.29149788	1.86566409	4.94264469	0.00026467	0.00224924
EC_2.3.1.31	-0.1823889	0.07879787	0.16502655	0.55913561	0.83072576	1.33997416	-0.7980353	0.0002647	0.00224924
EC_3.1.1.45	6.85293813	4.09911903	6.15954922	0.40611714	1.75736768	1.76122319	4.76538904	0.0002674	0.0022602
EC_2.7.7.59	7.10189116	4.65304616	10.7531968	1.05343696	1.39489639	4.58569977	5.05273296	0.00027184	0.00228575
EC_1.17.99.7	7.59706858	3.5701041	7.15388996	0.79175259	1.84820566	1.842906	4.84018689	0.00027443	0.00228985
EC_1.6.6.9	6.52444026	4.47713341	7.09301883	0.4667856	1.6790511	1.75941798	4.83492249	0.00027752	0.00228985
EC_1.7.1.13	0.18343013	-0.1813865	0.17900897	-0.2883234	-0.4856023	-1.0796049	0.51060352	0.00027808	0.00228985
EC_2.3.1.109	7.65356956	2.86877405	4.59634288	-0.3150273	1.73855549	1.7270957	4.95754333	0.00027953	0.00228985
EC_3.5.4.32	-1.2459527	0.00385437	0	2.58821339	2.74478131	4.42386302	-3.5311944	0.00028056	0.00228985
EC_1.1.1.30	5.35410704	3.76103902	2.72044429	-0.7577682	-0.2949726	0.04183798	4.9680456	0.00028116	0.00228985
EC_4.2.1.80	8.11082376	3.10816016	8.23862751	-0.1279273	1.61921168	3.21603571	5.47041445	0.00028234	0.00228985
EC_3.1.27.6	7.88060122	5.35801108	4.96502358	1.12022586	2.05786311	1.94743433	5.09343136	0.00028471	0.00228985
EC_1.6.1.1	8.19694713	4.68818226	7.89215271	0.515736	2.61398786	3.18504084	5.3624672	0.00028516	0.00228985
EC_3.6.3.27	7.80050499	5.22871188	9.77444351	0.57787879	1.95466854	3.05764159	5.77651394	0.00028774	0.00229904

EC_3.2.1.183	-3.7507938	-1.1117394	1.83855198	1.56347172	1.64146531	8.76014548	-4.6651886	0.00029196	0.00232126
EC_2.7.1.184	6.92626347	4.32125623	7.10430079	0.79088423	1.85074687	1.82149322	4.75162756	0.00029518	0.00233529
EC_3.1.1.10	7.52992283	0.93951385	2.84386337	0.71531807	0.90036889	0	3.93919646	0.0003022	0.00235956
EC_3.2.2.27	0.00649382	-0.1383738	0.12925034	0.43701585	0.52463287	1.07298938	-0.5790428	0.0003026	0.00235956
EC_3.4.24.30	7.33731324	0.99095121	2.6438275	0.70570835	0.85942854	0	3.85073885	0.00030266	0.00235956
EC_3.4.21.83	7.77089567	4.5322192	7.61361737	0.56567169	2.14053712	2.04821027	5.32770623	0.00030574	0.0023641
EC_3.1.26.11	-0.2461651	-0.2538334	-0.222081	0.32236033	0.34717001	0.96345428	-0.6606224	0.00030618	0.0023641
EC_3.5.1.94	7.41417166	3.59292738	7.33320482	0.51108339	0.92715588	1.90353783	5.2203751	0.00030834	0.00236934
EC_3.5.1.96	7.00631819	3.39196729	4.51274602	0.40536724	1.16610831	1.70435265	4.59507306	0.00033323	0.00253974
EC_2.3.1.129	0.57806673	0.18493369	0.84455078	-0.1565367	-0.4245098	-0.7721163	0.79673258	0.00033368	0.00253974
EC_2.7.8.40	-4.1142113	-1.3768528	1.03938785	1.10308715	3.55859584	6.99652577	-5.1952323	0.00033719	0.00255434
EC_2.7.3.3	-2.0071597	-1.1820432	0.65750261	0.11936891	1.04977206	2.82722997	-2.1683851	0.00034076	0.00256909
EC_1.1.99.28	-2.8917012	0.63838849	0.04095386	2.82755814	2.1695457	8.3927069	-4.6245909	0.00034233	0.00256909
EC_1.7.1.4	8.16706505	5.10488229	7.34732525	1.18533124	1.48381331	2.16900467	5.56957526	0.00034553	0.00257331
EC_2.1.1.187	8.79294015	5.24583875	4.56426304	0.56968829	2.8597144	3.72830943	5.28284397	0.00034701	0.00257331
EC_4.1.1.49	0.25911408	0.01483732	-0.3069765	-0.1290425	-0.5645892	-0.9295215	0.4801949	0.0003477	0.00257331
EC_1.5.3.1	-4.3307785	0.65427601	0.809766	1.76368833	2.22417862	4.14431991	-4.1623537	0.00035764	0.00263472
EC_4.3.99.4	-2.7379033	-0.5418412	0.91689305	1.60719004	1.39902717	5.89400672	-3.6140418	0.0003678	0.00268057
EC_3.6.3.24	10.8921988	5.76959299	9.07330847	3.54729776	2.05708001	5.83632552	5.49555111	0.00036794	0.00268057
EC_1.1.5.2	8.12464418	4.79499444	7.55571866	0.87283323	2.59239588	3.03476644	5.15494237	0.00036887	0.00268057
EC_4.1.2.57	5.47715348	3.3254395	5.95518998	0.63591132	0.86976048	1.33225179	3.96956555	0.00037136	0.00268653
EC_3.6.3.29	4.72258716	2.98698475	7.61418412	-0.4866893	0.30380223	-0.5270483	4.69504169	0.00037514	0.00269715
EC_4.1.1.47	7.29922602	4.47149823	7.62839385	0.57394685	0.96571287	3.05719897	5.30944888	0.00037619	0.00269715
EC_1.2.1.19	7.42670067	4.14157437	4.95854737	0.42888573	1.39248425	1.90381885	5.02203931	0.00037905	0.00270554
EC_5.4.99.27	7.42603025	4.27984485	4.98036222	0.43047214	1.3806208	1.99702223	5.06412896	0.00038165	0.00271205
EC_5.4.99.22	6.87808424	1.99494197	9.6433645	-0.0901632	1.55825341	1.68330183	4.80414419	0.00039542	0.00279755
EC_5.1.3.23	-3.2989654	0.4548269	-0.1803024	2.16313339	1.23324986	6.19110261	-3.9743662	0.00039844	0.00280656
EC_3.4.11.2	8.06035367	4.59591372	7.81925746	1.23795247	1.58111223	3.13046168	5.2046749	0.00040127	0.00281418
EC_5.1.3.15	6.91257314	2.83519382	6.9218704	0.37826302	1.06994703	1.70005572	4.69084541	0.00040721	0.00284338
EC_4.1.1.85	8.98430265	3.08242487	7.83684688	0.83420264	1.54637512	2.09623905	5.51974888	0.00041276	0.00286968
EC_2.4.1.109	-0.8807231	-0.0318596	0	1.55658458	2.51501322	6.08217509	-2.9373251	0.00042343	0.00293116
EC_2.8.3.5	6.75019383	4.04580221	6.32084077	0.74497242	1.84415384	0.3771306	4.68259392	0.00042664	0.0029407
EC_6.4.1.6	7.02264225	3.31745237	3.10703957	0.77927107	-0.3031614	1.24271093	4.7451321	0.00043002	0.00294993
EC_2.5.1.64	7.55882466	3.35873816	7.01286403	0.8182908	1.82701044	1.83864722	4.72118371	0.00043165	0.00294993
EC_1.2.1.5	7.37777187	4.22552812	6.81154257	0.43826091	1.89951947	2.9209703	4.94837831	0.00043436	0.00295588
EC_3.1.21.1	7.25937827	4.16588204	6.91070869	0.8291155	1.91550589	1.93394037	4.79048611	0.00043825	0.00296611
EC_2.7.2.15	7.02987808	4.00837047	6.53613722	0.78361956	1.90898688	1.71295227	4.62446695	0.00043956	0.00296611
EC_3.5.99.4	8.82123113	1.87921691	3.27369174	0.79599236	1.00317105	3.66745734	4.44137937	0.00044394	0.00297542
EC_6.3.4.5	-0.152401	0.26189562	0.63590288	0.50949159	0.83924608	1.6350743	-0.6744822	0.00044465	0.00297542
EC_3.1.3.11	0.53609654	0.27975369	0.55351677	0.01499866	-0.5125679	-1.0644332	0.74719083	0.00044766	0.00298315
EC_3.5.1.10	0.80809493	0.72939505	0.89059344	-0.284135	-0.5358059	-1.3942302	1.30278203	0.00045438	0.00301542
EC_2.7.1.202	7.73084395	3.41824758	4.87094992	0.5930564	1.438733	1.93100662	4.80672485	0.00046671	0.00308449
EC_1.1.1.399	4.73904329	3.13506	3.54025673	0.59667846	1.20479469	0	3.30626747	0.00047511	0.00312716
EC_3.6.1.66	-0.2160569	-0.2105778	0.24097587	0.30107349	0.61502849	1.16207298	-0.6751964	0.00048324	0.00316766
EC_7.1.1.2	-2.1112736	-1.9535642	-1.0825299	-0.2832734	0.201814	1.44880057	-2.0325894	0.00049016	0.0032
EC_1.2.99.6	7.10896362	4.18494867	4.85794912	0.50319824	1.46539143	1.91083333	4.79380864	0.00049793	0.00322504
EC_1.2.1.18	7.67881983	1.71113701	2.77280789	0.71523385	0.86446521	2.31316858	3.98972355	0.00049801	0.00322504
EC_2.3.2.10	7.51185983	1.01868154	2.79521089	0.69379738	0.85887736	1.04006806	3.84046924	0.00050242	0.00324048
EC_2.7.1.197	6.74143149	3.12993487	6.31717375	0.28823309	1.07977399	0.3335203	4.85651578	0.00051022	0.00327767

EC_1.17.7.1	7.53502611	3.77347535	7.04074973	0.8159132	2.42695336	1.77223313	4.67233745	0.00052098	0.00333021
EC_4.2.1.68	-2.3314433	-2.4415142	-0.403278	1.6942771	1.53666795	5.4831796	-4.2743114	0.00052255	0.00333021
EC_4.1.2.52	7.53837573	2.91158355	6.6562192	0.74224762	1.2971052	1.8467723	4.72219855	0.00052583	0.00333495
EC_1.2.7.4	-0.3280334	0.4494353	0.29078205	0.8700502	1.1324546	2.40500552	-1.1344923	0.00052745	0.00333495
EC_1.11.1.9	6.69565081	2.75498511	6.81211285	0.77147125	1.03150374	1.68626783	4.3372637	0.00053556	0.003373
EC_2.4.1.4	-1.1550325	0.45970458	0.45995039	1.33380671	1.61073563	4.33540462	-2.2039737	0.00054619	0.00342648
EC_3.2.1.185	-2.7296298	0.09784336	3.89582638	2.27155166	2.17778187	5.0695085	-3.52435	0.00054968	0.00343499
EC_5.3.1.n1	7.57301447	1.02438613	2.72801201	0.71211734	0.85625683	1.11962232	3.8467558	0.00055744	0.00346512
EC_2.7.1.58	7.57629651	5.07654298	4.85094433	1.53582265	1.84085731	1.93354123	4.66876961	0.00055882	0.00346512
EC_2.3.2.17	7.57146075	0.94450836	2.82368703	0.71329581	0.86870078	1.08262489	3.82951818	0.00056983	0.00351977
EC_6.2.1.27	6.93952374	3.57303073	4.64802251	0.43344506	1.3445606	1.7900317	4.55588673	0.0005741	0.00353259
EC_2.7.1.22	6.88497351	3.39105241	4.57760657	0.42167809	1.17572743	1.79114467	4.51583394	0.00057906	0.003536
EC_2.7.7.1	6.88497351	3.39105241	4.57760657	0.42167809	1.17572743	1.79114467	4.51583394	0.00057906	0.003536
EC_3.1.1.41	7.55003567	1.69146778	2.74783522	0.71139697	1.44837924	1.17473873	3.87243757	0.00058562	0.00354487
EC_3.4.19.5	7.07205311	3.58485146	6.8007997	0.39245498	1.76393705	2.93205004	4.62794432	0.00058685	0.00354487
EC_3.5.1.78	7.66672997	4.05125486	7.41769704	0.92923073	1.39873253	3.07838941	4.99016942	0.00058713	0.00354487
EC_1.14.11.17	7.03187778	3.76113749	7.08139616	0.63811425	0.8302995	0.76065765	5.16222689	0.00060659	0.00364865
EC_3.1.1.85	7.39208749	4.36456154	6.96782313	0.8295589	1.94514449	3.19221916	4.76208307	0.00061638	0.00368113
EC_3.6.1.40	6.77518369	4.07436237	5.50093935	0.03284411	1.37908052	2.05081726	4.92307255	0.00061695	0.00368113
EC_2.3.1.267	7.12538014	3.12192801	6.48964483	0.79564919	1.71034932	1.80583596	4.40118958	0.00061887	0.00368113
EC_2.4.1.246	7.58881458	1.10947828	2.94806706	0.70995762	0.90319926	1.23432906	3.8828447	0.00063129	0.00374115
EC_2.7.1.29	-4.6578248	-2.0059759	-3.2314729	0.27150776	-1.5050579	0	-3.1970352	0.00063644	0.00375781
EC_3.1.3.90	7.52268862	1.01878459	3.13398581	0.72492978	0.88192585	1.16871306	3.84640591	0.00064645	0.00379335
EC_2.1.1.242	7.36354134	3.53884457	6.96020255	0.47153715	2.53145408	1.93930808	4.61965168	0.00064718	0.00379335
EC_2.6.1.37	-2.002648	-0.8852263	-1.8309019	0.17132814	0.35587732	2.53116782	-2.1275768	0.00066761	0.00387409
EC_3.1.1.24	-3.0769582	-1.2264989	-1.0243129	1.75355171	0.66785574	6.64673479	-4.2101374	0.00066803	0.00387409
EC_2.1.1.197	1.12720757	0.75691732	0.56800203	0.03213901	-0.4940014	-1.4604688	1.26392265	0.0006682	0.00387409
EC_6.3.1.11	8.37932644	3.00117014	7.12757726	0.84087806	1.90444365	2.91319246	4.8591779	0.00067202	0.00388225
EC_2.6.1.17	6.98484715	2.91357284	4.27508359	0.78498408	1.20699026	1.69084836	4.16834584	0.00067881	0.0039074
EC_3.1.3.12	6.64990271	3.18082422	6.85514511	0.76519845	1.68591652	1.68857023	4.25794249	0.00068364	0.00392119
EC_6.3.3.1	0.07742344	0.13207313	-0.3505959	0.58942947	0.63103595	1.33961137	-0.6572776	0.00070621	0.0040362
EC_1.14.12.19	7.50534456	3.91420306	8.17689749	0.53135896	2.1998268	3.19247901	4.88773203	0.00071477	0.00407064
EC_2.1.1.227	7.60220676	1.63289152	3.03747131	0.72853129	1.5135894	1.24062465	3.87518635	0.00072472	0.00411274
EC_1.5.5.1	7.65725696	3.50947411	7.51871863	0.90132974	1.44701193	2.0809639	4.93594978	0.00074354	0.00419587
EC_5.4.99.29	6.23071331	3.68182813	4.47321943	0.4424018	1.13311931	1.7488622	4.27201888	0.0007446	0.00419587
EC_3.6.3.15	-2.1321206	-0.6180125	3.9072999	2.11060977	2.19135397	4.88906231	-3.3575821	0.00075168	0.00420564
EC_2.7.8.20	7.28607944	3.67943237	7.29966262	0.86104951	1.60359288	2.00008851	4.75614886	0.00075413	0.00420564
EC_2.6.1.83	-0.3074431	0.0862134	1.10779463	0.51383399	0.66485947	1.48221531	-0.6850853	0.00075419	0.00420564
EC_2.7.7.19	6.06802599	3.61725662	10.9303651	0.13630717	0.19781339	2.0524975	5.38779066	0.00077127	0.00428603
EC_4.3.2.7	7.2714159	2.84897581	6.63095672	0.78527442	1.84543292	1.70145162	4.37326701	0.00077534	0.00429379
EC_1.16.1.9	7.2167594	4.5169507	5.12708037	0.48903454	1.5136402	1.99953296	4.9809819	0.00079114	0.00436624
EC_2.6.1.107	5.98640048	2.93499274	5.9662924	0.67910565	1.58377781	1.54697326	3.81340232	0.00080174	0.00438656
EC_5.4.99.61	-2.3320125	-0.7799486	0.32544829	0.24402135	1.24956124	2.87529623	-2.3758673	0.00080176	0.00438656
EC_2.6.1.88	7.38330849	3.66473344	7.22381202	0.83385038	1.96779776	2.90976988	4.56833327	0.00080302	0.00438656
EC_1.13.11.15	7.08706947	3.45453679	7.14762867	0.80625588	1.90949727	1.86884493	4.50029005	0.00081346	0.00442851
EC_5.3.1.27	-0.8206606	2.01307913	-2.1901752	3.55815515	3.93064847	4.11088011	-3.7376953	0.0008261	0.00448213
EC_3.1.4.37	-3.1155832	-2.2963579	-2.6829047	0.78915472	0.7381842	5.62841685	-4.180385	0.00083291	0.00450388
EC_1.8.99.2	-1.6954278	-2.3415589	-0.2365766	1.2840005	1.29321384	0	-2.8638681	0.00084846	0.00457257
EC_3.7.1.20	-1.8886429	-6.068216	-6.9666459	0.7495714	-1.2822991	-0.6731856	-3.8879108	0.00085194	0.00457599
EC_3.4.21.89	-0.09824	-0.002365	0.44984079	0.48461501	0.90170126	1.49086562	-0.7506363	0.00087588	0.00466455

EC_5.4.2.10	0.09468707	0.22605957	0.66976148	0.76517196	1.16977313	2.31552057	-0.8897495	0.00087747	0.00466455
EC_3.5.1.53	-3.3244053	-0.6723115	1.14256438	0.94589554	0.50778354	5.9993911	-3.3034689	0.00087768	0.00466455
EC_4.3.2.10	-0.1901086	0.12478141	0.79896442	0.60314945	0.92653854	1.59126209	-0.796308	0.00088167	0.00466455
EC_4.2.2.n2	4.84667808	1.93493959	3.24303591	0.56135467	0.72772809	0	3.07660497	0.00088295	0.00466455
EC_5.1.3.24	6.76522433	3.19805006	4.50292161	0.47823339	1.33227612	0.89637922	4.41126014	0.000889	0.00468109
EC_5.1.3.29	6.52910169	4.24951712	6.98385488	1.15477569	1.27145816	1.81814277	4.49725314	0.00089383	0.00469115
EC_1.1.1.305	6.4201212	3.07999263	6.25759368	0.75015877	1.68120122	1.58726293	4.05174275	0.00092581	0.00482742
EC_2.1.2.13	6.4201212	3.07999263	6.25759368	0.75015877	1.68120122	1.58726293	4.05174275	0.00092581	0.00482742
EC_2.2.1.9	1.12234887	0.09218601	2.41833784	-0.2772116	-0.1001147	-0.059037	1.10679936	0.00093505	0.00485985
EC_6.2.1.14	-3.1152319	0.58007452	4.06446891	2.23994551	2.99001883	7.21077433	-4.0650946	0.00095147	0.00492923
EC_1.8.4.12	1.11807871	0.70249646	2.13031962	-0.4300773	0.16161223	0.68070885	1.18512638	0.00096584	0.00498756
EC_2.7.1.30	0.1221176	0.28618233	0.79539978	0.81051959	0.8134985	1.88241577	-0.6895217	0.00097129	0.00499968
EC_1.2.98.1	7.02081497	3.35220722	4.8501412	0.48593044	1.34496025	1.84012764	4.50988575	0.00098745	0.0050666
EC_2.1.1.131	-1.1624489	-0.1897899	-0.2939152	0.42347432	1.05192674	3.05099949	-1.6861066	0.00101945	0.00521412
EC_5.1.2.3	7.06949518	3.01761658	4.71816057	0.47327879	1.41152826	1.82025844	4.38829215	0.00102402	0.00522089
EC_3.5.1.11	6.81017765	4.14867476	6.2600131	1.13980274	1.90797687	1.8416004	4.31618713	0.00103432	0.00525671
EC_2.8.3.19	6.87202176	3.01311099	4.28137251	0.73646643	1.72195632	1.69942652	4.00159307	0.00103909	0.00526427
EC_5.3.3.8	6.4812901	2.72278151	4.39845945	0.41112276	1.25756664	1.63325376	4.04556412	0.00105028	0.00530424
EC_5.4.3.2	7.271155	4.70467067	7.14521196	0.83771651	2.4266312	4.17085161	4.55019788	0.00105398	0.00530624
EC_2.1.3.15	-0.0390619	0.13532977	0.64497725	0.70085835	0.96750793	2.60849705	-0.9322825	0.00105936	0.00531468
EC_1.2.1.72	7.2063562	4.64847545	7.14721077	0.8677891	2.04949032	4.12855522	4.60958992	0.00106254	0.00531468
EC_3.2.1.196	6.10820723	2.80710352	5.93765645	0.71545745	1.53884669	1.58132797	3.81889051	0.00106558	0.00531468
EC_1.1.1.85	-0.2644362	0.16570963	0.66622391	0.42739905	0.78797971	1.40493912	-0.6720277	0.00106915	0.00531594
EC_1.1.1.29	-0.1579588	0.3976142	0.84041852	1.60142178	3.33058432	7.54483773	-2.7877918	0.00107698	0.00533837
EC_2.7.1.60	7.52024931	3.85049169	7.49307193	0.84560839	2.07414012	2.04595813	4.81100359	0.00112378	0.00555321
EC_2.7.1.48	0.51447591	-0.0331883	0.42263291	-0.113065	-0.2865087	-0.2399982	0.49584403	0.00113156	0.0055745
EC_2.3.1.193	6.57064064	3.1308155	4.64900995	0.46613473	1.27497954	1.7476026	4.21707371	0.0011384	0.00559105
EC_1.7.5.1	7.32450549	3.20880699	4.6583199	0.80063973	1.83900522	1.79093333	4.26876352	0.00115785	0.00564345
EC_2.4.1.329	-1.9015593	1.52356532	3.21668437	2.37855778	3.2103113	8.6358362	-3.5341372	0.001158	0.00564345
EC_3.11.1.1	3.77266305	4.57258571	2.31926681	1.366872	-1.9242005	-1.2991305	3.94025735	0.00115961	0.00564345
EC_3.6.1.15	6.25986401	4.15511644	4.54336643	0.79126537	1.79637414	1.79884274	4.05080963	0.00117148	0.00567144
EC_3.5.1.105	6.58524075	3.35428925	4.32422814	0.75477323	1.63607496	1.59434466	4.00969896	0.00117594	0.00567144
EC_2.6.1.82	8.17712271	5.35951191	7.26317393	1.91016371	2.60650914	3.10514664	4.77167017	0.00117596	0.00567144
EC_1.1.1.282	7.53460129	3.27886214	5.00150304	0.508458	1.48597018	1.95949884	4.69719254	0.00119498	0.00574134
EC_1.1.1.22	0.07845373	-0.1446105	-0.1906337	-0.1865069	-0.479629	-1.2165746	0.38436748	0.0011976	0.00574134
EC_3.1.4.17	6.24938619	3.84184701	6.2293318	0.70485479	1.78475382	1.75899159	4.1967834	0.0012115	0.00579067
EC_2.7.1.41	7.74599429	1.060405	2.96643846	0.71531865	1.4968775	1.22323592	3.75384853	0.00122953	0.00585944
EC_2.7.1.165	6.97849533	2.65602495	4.67091817	0.36293985	1.2584915	1.80558862	4.31858685	0.00123387	0.00586271
EC_5.1.3.41	6.7475949	2.65823242	4.52131771	0.40185327	1.28068531	1.7486306	4.15937794	0.00124084	0.00587845
EC_2.3.1.242	6.09406527	2.50864829	4.10406068	0.69886967	0.9471365	1.46876988	3.69881304	0.00124633	0.00588646
EC_4.1.1.98	6.14984833	2.99974562	4.4183313	0.49211452	1.39871388	1.70997565	3.8725231	0.00125341	0.00588646
EC_2.4.1.44	5.51523461	2.18888358	3.79696697	0.32274195	0.82501168	0.51319053	3.61399213	0.00125353	0.00588646
EC_1.1.1.37	0.04474674	-0.289935	-0.2572807	-0.4494164	-0.8892689	-1.3316843	0.59869912	0.00126961	0.0059446
EC_3.5.4.30	-2.6370114	-0.0286362	0.88018197	0.75815931	2.33502093	3.96524941	-2.9784437	0.00132842	0.00618746
EC_1.17.4.1	0.86911434	0.13063207	0.6291858	-0.1098561	0.01105199	-0.3830975	0.68423353	0.00133324	0.00618746
EC_2.4.99.13	5.91734044	2.72922423	3.92036914	0.66055974	1.5598466	1.51185848	3.47610967	0.00133509	0.00618746
EC_2.1.1.195	-1.5345946	-0.179101	-0.4625227	0.17890785	0.78273392	2.76602727	-1.6397574	0.00133689	0.00618746
EC_3.1.3.70	7.29086279	2.98266111	4.86139811	0.48371549	1.43808762	1.92676296	4.48095378	0.00137253	0.00633415
EC_3.5.3.24	-2.6909548	-5.1640271	-6.6430017	1.47786429	-1.1050979	1.37464693	-4.6686679	0.00142152	0.00654144
EC_7.2.4.2	-2.5490801	-1.2178692	-0.2110649	0.10470599	0.57678006	1.8930222	-2.2853675	0.00142569	0.00654186
EC_1.5.1.34	6.23145137	3.83186813	7.11493442	-0.0250515	1.45478779	3.11684239	4.61698308	0.00144603	0.00661482
EC_4.1.1.19	0.94554609	0.11749485	1.28566037	0.10467861	-0.1595249	-0.5445678	0.761159	0.001451	0.00661482

EC_1.1.1.34	6.94187178	0.98736715	2.50773328	0.67851648	0.83570074	2.16430742	3.36411733	0.00145394	0.00661482
EC_7.1.1.3	6.82375899	2.24721553	4.41292355	0.53530565	1.4351249	1.70107899	3.9238	0.00146327	0.0066166
EC_2.3.2.2	7.41849907	5.31220622	7.92555003	0.90006002	2.77837949	3.26126285	4.90853527	0.00146452	0.0066166
EC_2.4.2.15	8.05870199	2.15170209	4.68656318	0.48719053	1.8913138	1.8166991	4.43092278	0.00146995	0.0066166
EC_4.2.1.135	-3.230281	-0.3693897	2.99008918	0.87521912	2.53028889	8.37138384	-3.8569588	0.00147081	0.0066166
EC_3.2.1.82	-3.0554736	-3.3408825	4.62785105	1.78833155	1.60301754	5.87813457	-4.4795421	0.00148322	0.00665379
EC_1.5.1.20	0.06959443	-0.0676539	0.17557353	0.53212381	0.53344056	1.10153135	-0.5733677	0.00150934	0.0067509
EC_3.6.3.16	5.40601799	2.07780959	2.79168942	0.71136747	0.87568637	0	3.23491727	0.00151328	0.0067509
EC_2.7.2.7	-0.2399481	-0.0844914	-1.0835768	0.371944	0.24407201	0.76719855	-0.6702121	0.00157992	0.00702866
EC_6.3.5.7	-0.4586445	0.12197876	1.08054698	0.54070442	1.22308475	2.44137284	-1.0784122	0.00158767	0.00703336
EC_1.1.1.284	7.32904869	2.48632918	6.61403049	1.12572949	1.80069895	1.75586659	4.09631294	0.00159449	0.00703336
EC_3.6.1.3	-1.5580833	0.16713312	0.51446236	0.4592558	1.08406437	2.88419866	-1.675594	0.00159988	0.00703336
EC_3.6.1.12	6.36144048	2.81263036	4.42781721	0.76274294	1.01027621	1.65887817	3.90585314	0.00160506	0.00703336
EC_4.2.1.55	-3.8554346	-1.2635042	-1.1868903	0.60999473	0.24627156	2.20335357	-3.310292	0.00160703	0.00703336
EC_2.3.1.241	6.88142991	2.54131918	4.34037735	0.74427735	1.68968933	1.61427069	3.86417959	0.00160725	0.00703336
EC_2.7.7.70	0.28166222	-0.6056324	0.18541151	1.02840719	0.62691369	1.21468648	-0.9656376	0.00163138	0.00710247
EC_3.1.6.6	1.36215229	1.18377619	2.2844449	0.40584295	-1.0474653	-0.0460966	1.5420628	0.00163189	0.00710247
EC_2.5.1.7	0.04095527	0.04168521	0.15893806	0.42151552	0.54351081	1.14216512	-0.5004889	0.00164325	0.0071326
EC_2.7.7.61	7.36632081	4.1501899	4.95550982	0.83693058	2.04372875	2.98639486	4.41674729	0.00166485	0.00720688
EC_1.14.13.127	7.63093911	3.9973794	7.28621969	0.97242731	2.61822712	2.00393537	4.65412907	0.00168942	0.00729356
EC_7.3.2.3	6.11550402	2.70565568	5.66958671	0.74155982	1.58149999	1.58785317	3.72496091	0.00171162	0.00736961
EC_3.2.1.98	6.20581461	2.3946522	5.69375352	0.71206685	1.56825994	1.52407995	3.69368505	0.00171873	0.00738045
EC_2.7.4.3	-0.041921	-0.1420184	0.50775759	0.33661958	0.58104217	1.28226457	-0.5509638	0.00173819	0.00740018
EC_1.2.7.8	-0.7652325	-0.1188835	-0.6680326	0.16948916	-0.0705474	-0.0572075	-0.5853912	0.00173955	0.00740018
EC_2.5.1.56	-3.0599345	-0.9417646	0.0439861	0.24239819	-0.0907999	2.88980096	-2.4100795	0.00173956	0.00740018
EC_2.5.1.84	-1.8766237	-6.1578218	-7.4232981	0.23409047	-1.520177	-1.0565426	-3.5622276	0.00174176	0.00740018
EC_1.1.1.67	6.84722689	4.94455972	7.86666582	0.7835244	2.55006939	1.9755256	4.77893168	0.00176156	0.00744585
EC_2.4.1.211	-2.4875975	-0.9072357	-0.1308542	0.02927945	0.84605719	2.39544768	-2.2468331	0.00176633	0.00744585
EC_1.1.1.264	4.32090928	1.48259661	2.64786816	0.10832144	0.51182052	-0.1877811	2.9087811	0.00176642	0.00744585
EC_4.1.1.64	-2.8050558	2.5497515	5.08837791	2.35245836	4.09881384	7.03034084	-3.4803614	0.00179427	0.00754343
EC_3.1.1.84	-1.9473744	2.24583857	7.52208576	2.86300341	5.7373642	6.80717498	-3.625001	0.00180685	0.00757652
EC_2.3.1.245	-1.6755167	0.24481778	0.9863256	1.18658996	1.79597286	3.67719347	-2.3825879	0.00181352	0.00758466
EC_3.4.22.34	1.07160577	2.58098897	0	-1.0166578	-1.233773	-1.41213	2.61467348	0.00184512	0.00768639
EC_1.1.1.42	-0.1172258	0.33930099	0.60509867	0.7527364	1.23777191	2.1478856	-0.9620005	0.00184741	0.00768639
EC_4.3.2.2	-0.0344567	-0.0929655	0.11823142	0.25625571	0.34304477	0.80399574	-0.3931167	0.00189949	0.00785228
EC_6.1.1.14	0.36780731	0.17210888	0.30188066	0.06888136	0.06203159	0.07374116	0.22313698	0.00189979	0.00785228
EC_3.7.1.14	7.44531836	3.07658059	7.32225811	0.50880226	2.63889998	3.21966342	4.31954483	0.00190195	0.00785228
EC_1.1.2.3	7.37235519	4.64178006	5.45332805	0.78980651	2.00834634	4.10436249	4.54529922	0.00191088	0.00786892
EC_1.14.14.51	6.05490194	2.69567331	3.86676182	0.68297011	1.57519365	1.46105643	3.51915322	0.00194398	0.00798474
EC_2.1.1.61	6.8585475	3.99297054	10.2309405	1.24395062	1.31354597	2.04014843	4.88556686	0.00198098	0.00811597
EC_1.5.5.2	5.41403143	1.50737571	3.29470732	0.56585532	1.21332591	1.1651385	2.91367055	0.00199728	0.0081619
EC_1.1.99.14	3.06260851	1.9515132	2.37206347	0.45409248	0.58106843	0	2.14833135	0.00200467	0.00817133
EC_2.7.7.53	-3.0740669	-1.7754869	-1.3819066	0.26937336	3.8806361	5.39793777	-4.5337619	0.00201435	0.00819001
EC_6.2.1.33	5.40119101	3.13498613	4.17610626	0.46919235	1.30475601	1.65105262	3.54995667	0.00205383	0.00832942
EC_2.7.7.49	-0.2811855	-0.2532353	0.51727972	0.22125103	0.47526057	1.12498338	-0.5966513	0.00206335	0.00834695
EC_3.5.1.32	-1.8485214	0.43045614	-0.5574214	1.92896462	2.15516859	5.35261916	-3.3358422	0.00208003	0.00839328
EC_3.1.3.97	6.11151004	2.76697249	4.08256663	0.70411271	1.63053728	1.49627927	3.5662709	0.00209093	0.00841614
EC_1.8.4.2	1.31445895	1.09503112	-0.5605096	-0.0954661	-2.5640259	-1.5296045	2.10018094	0.0021141	0.00848811
EC_3.1.3.6	7.31991726	5.54417413	5.19160056	1.02856796	2.35000791	0.35907082	5.05615136	0.0021277	0.00849663
EC_1.97.1.9	4.61852005	2.98207286	3.1897129	1.01476928	1.18207471	0.73580131	2.83077625	0.00212852	0.00849663
EC_2.1.1.200	7.08819414	3.09971824	7.55090547	0.82502799	2.02277592	1.89628797	4.37373104	0.00213209	0.00849663
EC_1.5.1.41	6.80258099	2.62973751	4.59069374	1.02219159	1.0664359	1.7514088	3.92098671	0.00214062	0.0085095

EC_3.1.2.2	6.58105546	2.81780854	4.45079781	0.75844377	1.51479221	1.73482662	3.85233443	0.00221104	0.00876773
EC_2.6.1.90	-2.4528866	-0.5035166	0.47217113	2.59512205	2.19771653	5.31021912	-4.2253042	0.00221731	0.00877092
EC_3.1.4.14	6.46048796	4.12124274	4.81491749	0.82383916	1.9005736	1.89345101	4.11286101	0.00230267	0.00908621
EC_2.3.2.6	-0.1522022	-0.2030783	0.87192702	0.85548434	1.22259775	2.66517655	-1.2582327	0.00231341	0.00910621
EC_2.7.7.40	-0.8159862	-0.5197294	0.32399819	0.83207491	1.86719413	3.07066804	-2.0368199	0.00235189	0.0091998
EC_6.4.1.8	-2.1816763	-1.6322507	2.11746641	1.75448549	1.8747797	3.0168224	-3.4198546	0.00235278	0.0091998
EC_4.2.1.33	-0.0159028	0.18673481	0.8509394	0.59463556	0.96997476	1.68419723	-0.6986876	0.00235437	0.0091998
EC_1.1.5.12	5.31021698	1.58175331	3.52811451	0.39599483	0.86093966	1.31251357	3.10235022	0.00238628	0.00930185
EC_1.1.1.65	5.20447956	1.08131829	3.5624788	0.6127239	0.83042815	0	2.93916561	0.00240999	0.00935658
EC_2.3.1.201	-0.7933635	-0.7017286	0	2.83529855	0.66280736	2.41341019	-2.7312017	0.00241197	0.00935658
EC_3.1.3.68	6.03353963	2.39453649	4.00752361	0.7032421	1.443567	1.43427905	3.45428038	0.00241871	0.0093601
EC_2.7.2.8	0.0102499	0.20445845	0.90313516	0.64471911	0.98711561	1.68479647	-0.7051112	0.00242971	0.00938009
EC_1.11.1.21	8.32620925	5.18646836	7.94675419	2.48125004	2.81250851	2.23084575	4.61062356	0.00244583	0.00941967
EC_1.4.7.1	-2.6386015	-0.656655	0.87605881	0.72947434	0.25424442	2.65416053	-2.3322404	0.00246045	0.00943125
EC_4.1.1.104	6.53481662	2.04765737	6.7242942	0.74368422	1.76571567	1.61466931	3.7754944	0.00246157	0.00943125
EC_1.2.5.1	7.2782326	3.63001747	7.73797047	0.80393369	1.96830688	4.44154465	4.37891293	0.00247526	0.00943125
EC_3.1.3.22	6.22812333	2.41257091	4.12038111	0.71982618	1.46924773	1.52242404	3.54734821	0.0024782	0.00943125
EC_3.1.3.50	6.22812333	2.41257091	4.12038111	0.71982618	1.46924773	1.52242404	3.54734821	0.0024782	0.00943125
EC_3.2.1.26	0.00121643	0.10415822	0.88039519	0.619945	1.08811197	2.43515514	-0.8664186	0.00250987	0.00952921
EC_2.8.1.4	-0.0480616	0.31326194	0.83832772	0.69833738	1.1039781	2.5434085	-0.8851061	0.00252325	0.00954015
EC_3.1.3.10	7.21917086	3.73705133	7.41442238	1.23577972	2.03059043	3.00627553	4.2808139	0.00252464	0.00954015
EC_4.2.2.17	-0.4535341	0.63698331	1.81965624	2.81427183	2.48118211	4.16609907	-2.671249	0.00254177	0.00958234
EC_3.5.1.91	-0.8115875	-0.5163138	1.87941635	1.55708989	2.50675429	3.56617843	-2.5095182	0.00255384	0.00960529
EC_4.1.1.11	0.03727146	-0.2529781	0.05078871	-0.278084	-0.6025863	-1.071241	0.42477823	0.00256805	0.00962169
EC_2.5.1.101	-3.2485628	0.05649222	0	0.945987	0.84524646	5.63992696	-3.2091771	0.00257018	0.00962169
EC_3.5.2.17	5.77540733	3.73163861	4.58222175	0.40986866	1.24324368	0.59420407	4.19792022	0.00257729	0.00962587
EC_2.7.1.194	6.45859296	3.89539387	4.69828788	0.79566943	1.90643061	1.76836796	4.04731946	0.00259077	0.00963002
EC_2.1.1.45	0.35496813	0.12170875	0.04747514	-0.0082547	-0.1468315	-0.3923277	0.33879496	0.00259682	0.00963002
EC_1.1.1.414	6.54576438	2.75283732	4.34604064	0.74082125	1.72128493	1.6818554	3.74679457	0.00259825	0.00963002
EC_4.2.1.104	7.07500525	2.83074896	7.48156479	0.54510634	1.84606362	3.10979874	4.31300301	0.00260238	0.00963002
EC_4.1.1.4	-1.3263432	-1.5025117	-2.3512068	0.74236818	0.59112212	3.5267569	-2.5728175	0.00261359	0.00964926
EC_2.3.1.118	7.64688869	3.67993114	5.05225864	1.22019602	2.03978038	1.92820008	4.34185689	0.00262534	0.0096704
EC_1.14.99.46	6.33553134	2.56510892	4.2505547	0.72789245	1.5921946	1.55486632	3.62459481	0.00264202	0.00970956
EC_4.1.2.20	7.18526814	3.51728702	7.83475804	0.84936423	2.07378322	1.97377735	4.56715459	0.00268918	0.00984044
EC_4.6.1.12	-0.110737	-0.1245293	0.22704856	0.20868402	0.21743666	0.35731817	-0.3052956	0.00268988	0.00984044
EC_2.3.1.n5	7.31872453	2.17849346	4.57458395	0.77804195	1.80236669	1.7741364	3.91697873	0.00270731	0.00988169
EC_1.5.1.42	5.91092532	1.84777608	4.24182254	0.35765883	0.8938489	1.56810825	3.57473478	0.00273023	0.00994275
EC_2.7.1.100	-1.0546422	-1.6682997	-0.4619542	1.49940508	1.59013804	8.11194617	-3.5969656	0.00274022	0.00994925
EC_6.1.1.12	0.09877662	-0.1196581	-0.2513765	-0.1499276	-0.4204585	-0.5824829	0.27393333	0.00274441	0.00994925
EC_3.2.2.28	6.82471802	2.79221582	6.47644997	1.11602938	1.38729844	1.85052723	4.05227871	0.00284562	0.01029294
EC_1.16.1.1	7.24441353	2.36261073	4.40443925	0.79285464	1.68516873	2.71676229	3.82907636	0.0028608	0.01032461
EC_6.1.1.6	0.27227154	0.06628012	0.32160488	0.01908731	-0.016223	-0.1262908	0.21694011	0.00287226	0.01034271
EC_6.3.2.14	7.24991743	2.10030905	7.05740354	0.81784865	1.91646426	1.86836255	4.08628196	0.0028889	0.01037935
EC_1.1.1.271	-0.1536318	0.05501435	-0.1096436	-0.3090406	-0.4659639	-0.9016138	0.36398272	0.0028965	0.01038344
EC_2.7.8.37	6.60054233	2.04544851	4.30332858	0.74331241	1.53548926	1.71270214	3.57518792	0.00293292	0.01048173
EC_3.6.1.67	5.56833676	1.80265715	4.02289331	0.417488	0.50007787	1.42880759	3.46772423	0.00293697	0.01048173
EC_5.4.2.7	-0.017096	0.34648671	1.19190919	0.86198208	1.29488823	2.89757751	-1.0114056	0.00294692	0.0104939
EC_3.1.3.74	6.82346495	2.55741728	4.56909879	0.77989505	1.69781064	1.7655948	3.82578812	0.00298682	0.01061245
EC_2.1.1.207	-0.1377742	0.20055874	1.07543873	0.63055491	1.17295637	2.32961886	-0.9007894	0.00300648	0.01065117
EC_1.11.1.15	0.93028905	0.34345938	0.75132183	0.10352117	0.1612482	-0.036099	0.59638596	0.00301098	0.01065117

EC_3.2.1.73	1.27912056	2.89770105	2.56150683	-0.6695722	-3.1035478	-0.217631	3.4179109	0.0030224	0.01066809
EC_3.4.11.18	-0.0809127	-0.0470543	-0.0999848	0.20735291	0.15506533	0.40109252	-0.2869103	0.00311625	0.01096706
EC_2.3.1.12	7.0759834	2.69992436	4.77957241	0.51517567	1.28099449	1.86758597	4.30061712	0.00312076	0.01096706
EC_7.5.2.6	6.39594197	2.53218839	4.30659971	0.74153638	1.65407335	1.61378223	3.61600672	0.00313338	0.01098734
EC_2.1.1.217	-1.354214	-0.8663295	-2.4470502	0.36768088	1.25117059	2.38582617	-2.2377781	0.00318603	0.01112898
EC_2.3.1.251	5.61041106	2.79957806	10.6118116	0.66252165	0.72047291	-0.2673888	4.6684278	0.00318763	0.01112898
EC_2.4.2.17	-0.0265257	0.17143758	0.88188918	0.60024699	0.91126748	1.62030579	-0.6812641	0.00325255	0.01124901
EC_6.3.1.1	0.00730902	0.32223836	0.7621292	0.52759247	0.74769878	1.62605528	-0.5326578	0.00326224	0.01124901
EC_3.5.1.46	-2.9845029	-0.893597	3.22623083	2.08477211	2.17146447	7.34892282	-4.2872359	0.00326695	0.01124901
EC_5.4.4.2	5.46153611	2.69822655	7.41652508	0.39835853	1.29077588	1.24751469	3.91988695	0.00327293	0.01124901
EC_2.7.11.33	6.11393146	2.66327002	10.0017419	0.86665044	0.74255887	1.83367172	4.41526202	0.00327542	0.01124901
EC_2.7.4.28	6.11393146	2.66327002	10.0017419	0.86665044	0.74255887	1.83367172	4.41526202	0.00327542	0.01124901
EC_3.2.2.9	-0.0152015	-0.0518489	0.97552411	0.47178586	0.94689675	1.81117817	-0.7102335	0.0032797	0.01124901
EC_2.6.1.85	3.39081167	2.15078384	5.27027738	0.55195487	0.94457873	1.07126549	2.43305635	0.00328214	0.01124901
EC_4.1.1.76	-1.3553893	-0.2410151	-2.4260591	2.9868251	-1.233033	6.33826113	-3.1352099	0.00328505	0.01124901
EC_1.14.13.239	6.93351984	2.8300547	4.74795867	0.80976051	1.86540045	1.82411106	3.92307511	0.00330455	0.0112763
EC_1.7.1.15	6.39248446	2.55595905	4.36752233	0.73353117	1.68596332	1.67579531	3.61581217	0.00330706	0.0112763
EC_2.7.7.12	-0.0566606	0.37453864	1.04040237	0.6841572	1.14364358	2.6437246	-0.8616465	0.00331959	0.01129504
EC_3.4.23.49	6.58832844	3.73988723	4.85112093	0.83514696	2.46013434	1.87377218	3.86210381	0.00335683	0.01139762
EC_6.3.5.3	0.61159673	0.19048383	0.90505384	0.04482609	-0.0311956	0.23807615	0.45312779	0.00339232	0.01149382
EC_2.3.1.174	7.67468945	2.98763919	6.7459943	0.84358342	3.07283751	2.87185918	4.05977811	0.00348757	0.01179166
EC_3.2.1.22	-0.0208919	0.4879156	0.30171134	0.74664354	0.95079805	1.17653234	-0.6711765	0.00352623	0.01189732
EC_4.1.3.40	6.47838298	3.12300895	7.05351904	0.80768643	1.89739108	1.85740166	4.0575421	0.00355486	0.01196879
EC_1.2.1.39	6.99364982	4.02327418	5.00206704	1.59564956	1.89010479	1.95625458	3.95788109	0.00356986	0.01199414
EC_2.4.1.301	-3.5409621	-1.5718231	-0.0444495	0.58279566	-0.2142444	5.98820527	-3.446301	0.00363569	0.01218981
EC_3.1.11.5	3.55863232	1.78693106	3.63748938	0.32043233	0.46144376	1.46380411	2.42392815	0.00364591	0.0121986
EC_3.4.11.1	5.5673935	6.32522239	9.23256405	1.44509397	1.72462002	5.0438271	4.27565807	0.00376523	0.01257164
EC_2.8.3.16	1.06950744	0.65690555	2.32766763	-0.7118043	0.6866756	0.88271194	1.12140435	0.00387139	0.01289928
EC_5.1.1.20	4.93024109	1.72716269	5.98697013	0.65653868	0.86055598	1.28379767	3.11941622	0.00396243	0.0131703
EC_3.1.12.1	0.0362346	-0.4546588	1.6884716	0.79597691	0.78855337	2.07738786	-0.8969658	0.00396913	0.0131703
EC_1.1.1.159	6.72551591	3.493934	7.64606013	1.20144408	2.04518133	2.9590137	3.98478666	0.00402072	0.01331398
EC_4.3.99.2	3.10479303	4.32029415	0	1.27326665	-1.831849	-1.4058518	3.25153127	0.00404823	0.01337748
EC_3.4.19.13	4.48169633	2.34684327	3.45464756	0.5906399	1.19112185	1.25140278	2.72533261	0.00408223	0.01346215
EC_2.1.2.1	0.07523769	-0.0176124	-0.0439403	-0.0676098	-0.2897188	-0.1482172	0.17876825	0.00413721	0.0136155
EC_1.1.1.6	3.03210074	1.78895962	7.03018282	-2.6972895	1.72714618	4.74133097	3.35139497	0.00415927	0.01366011
EC_2.5.1.16	0.14950612	-0.2092355	0.48182243	0.6541143	1.46828912	3.22676644	-1.1957869	0.00418229	0.01370766
EC_4.1.1.77	5.76000471	2.8654717	7.05540393	0.44168514	1.90266199	1.92363806	3.77816841	0.00422299	0.01378063
EC_3.5.3.23	7.26939034	4.43010779	4.78139794	1.57738621	2.47394074	1.84429704	4.05235296	0.00422369	0.01378063
EC_7.3.2.5	6.1050351	2.65063052	4.39283945	0.75090639	1.78321835	1.62095205	3.46796186	0.00423229	0.01378063
EC_5.4.99.9	-1.3181226	-0.1570535	-0.4604089	0.15758736	0.79267453	2.6567432	-1.495801	0.00423888	0.01378063
EC_1.1.1.237	5.25535435	2.48805345	3.8238151	0.6612448	1.35452238	1.37861314	3.11693408	0.00426779	0.01383612
EC_1.3.1.87	5.95452726	2.14235649	6.89774645	0.45838673	1.3106334	1.7089321	3.81778975	0.00427317	0.01383612
EC_1.21.4.2	-0.510783	0.48981772	1.11986933	1.37780838	1.36691111	2.99314939	-1.5428914	0.00433632	0.01401235
EC_3.5.4.1	2.23236938	1.11447924	2.71411695	-0.4207132	0.88801123	2.23395259	1.53839631	0.00437532	0.01410997
EC_2.5.1.54	0.15754336	0.18324111	0.44699105	0.88000042	0.99232449	1.8468915	-0.841695	0.00439719	0.01415206
EC_1.1.1.298	6.6831377	3.10877645	4.87655466	0.83576681	1.84038122	1.86876309	3.89482759	0.00444275	0.01427011
EC_1.2.2.1	4.51258372	2.41583575	3.38829308	0.95392529	1.08074056	1.10778013	2.61640139	0.00449924	0.01440775
EC_4.2.1.99	6.13519994	2.15388506	4.35983094	0.74560606	1.46951431	1.6495327	3.40572776	0.00450354	0.01440775
EC_1.3.99.2	-2.8475345	-0.6257549	-0.1561445	-0.0938283	-0.2479661	2.2314988	-1.8917311	0.00454576	0.01451391
EC_3.5.3.9	5.76612364	1.74833392	3.90821036	0.66825391	1.46531259	1.24587337	3.10983654	0.00463437	0.01476745

EC_1.14.12.10	5.46927648	1.87264482	3.94798851	0.69223244	0.91987968	1.41574128	3.14699236	0.00467053	0.0148532
EC_1.14.13.149	5.08086723	1.66758916	3.85455054	0.3260721	0.80570297	0.33786516	3.23668891	0.00468138	0.01485828
EC_1.17.7.3	-0.0963444	0.0715411	-0.5613622	0.34722419	0.30483784	0.54158934	-0.452728	0.00469308	0.01486606
EC_3.2.2.10	5.74700781	1.84557307	5.79543007	0.7005169	1.45310031	1.63837742	3.30059585	0.00471759	0.0148714
EC_4.3.1.4	0.359588	0.55670083	-2.342885	-0.3599299	-1.2928092	-1.8750718	0.96298008	0.00471935	0.0148714
EC_1.5.1.43	-2.3276742	-0.4544626	2.05821235	0.75959525	1.95322195	5.52680856	-2.9036998	0.00472255	0.0148714
EC_2.6.1.66	6.41499489	1.33418671	4.33424644	0.74222569	1.0022837	1.58099617	3.42268621	0.0047582	0.01495434
EC_3.7.1.22	-2.6878129	0.57409361	1.4927136	0.67269218	2.56896234	6.52589293	-3.0832534	0.00477079	0.01496462
EC_5.1.3.13	-0.1456951	-0.0549634	0.0508415	0.13967593	0.20950037	0.66913243	-0.3215761	0.00479106	0.01499891
EC_4.1.1.37	6.26944849	5.36286492	8.40989952	1.36943417	2.12951619	3.32794752	4.33480242	0.00480181	0.01500333
EC_3.6.1.65	6.02904312	1.37995693	7.07456915	0.76981173	1.03822549	1.7309964	3.52642542	0.00482877	0.01504484
EC_2.7.7.85	-0.1843415	-0.2506904	0.09793818	0.16598715	0.17034707	0.72426775	-0.4139823	0.00483839	0.01504484
EC_4.4.1.13	7.01109138	2.07507223	4.66411711	0.78963469	1.76759197	1.80318884	3.73167485	0.0048432	0.01504484
EC_4.2.1.119	7.57144268	3.28975178	3.07247562	1.81406044	3.17338208	2.28771001	3.18145929	0.00485742	0.01505988
EC_4.4.1.28	6.95725685	2.06573451	4.6390025	0.78465749	1.76173824	1.80318884	3.70173212	0.00489764	0.01515532
EC_1.1.1.336	2.79114457	0.51881986	1.73292162	0.91615996	-1.6748386	-5.5053299	2.63336957	0.00494246	0.01526459
EC_2.4.1.227	-0.0049178	-0.1023451	-0.0257338	0.2497096	0.31355475	0.62158078	-0.3614616	0.00498924	0.01537951
EC_6.5.1.1	-1.962313	-1.8734891	-0.2573724	0.11320461	1.44810955	2.62855895	-2.6031513	0.00501579	0.01540418
EC_1.1.5.3	1.99156716	0.44988779	3.21284791	0.41764062	0.47498685	0.47251322	1.15154287	0.00501643	0.01540418
EC_3.5.2.12	8.04675088	0.89003275	0.45969452	0.41589813	0.45490414	3.35795534	3.76683993	0.00510448	0.01564465
EC_2.7.1.192	6.17190078	1.24799364	4.21356115	0.71740942	0.97182382	1.60510341	3.27041113	0.00515026	0.01575489
EC_2.4.1.352	5.26796518	1.69401722	3.58781481	0.61861414	1.40564898	1.24875575	2.8367031	0.00516551	0.0157715
EC_4.2.3.5	0.06787604	-0.0263524	0.53683717	0.47562976	0.59602058	1.21877409	-0.5207265	0.00518639	0.01580519
EC_2.4.2.9	-0.1627526	0.0283321	0.43591542	0.29626105	0.85487396	2.04286466	-0.7305115	0.00524625	0.01595735
EC_1.1.1.8	6.70000472	3.45559924	4.82317415	0.76254393	1.94132731	2.87848811	3.89383976	0.00527317	0.0160089
EC_3.2.2.31	6.11553745	1.83469123	4.18575351	0.71542482	1.51272211	1.5652043	3.27427376	0.00530818	0.0160848
EC_2.6.1.33	-1.6937892	-1.4296298	-2.8473956	1.37744088	0.7788395	4.70783395	-3.3601743	0.00533023	0.0161024
EC_3.2.1.131	-0.341305	1.14996246	0.19195968	0.92090128	1.26563709	2.10170189	-0.9367571	0.00533405	0.0161024
EC_3.1.21.5	-0.8716546	-0.2096354	2.4935554	1.47999501	1.36930208	7.37086509	-2.4420767	0.00544913	0.01641895
EC_1.3.1.n3	6.34896717	1.9993142	4.34994001	0.75085908	1.6523623	1.55140897	3.41170762	0.00546833	0.01644595
EC_2.7.7.58	6.59331745	1.35818888	6.26698981	0.76126051	1.74095478	1.68098382	3.49574907	0.00550575	0.01651262
EC_3.5.1.124	4.06700221	0.89693344	4.90166396	0.47966547	0.22339515	1.0230706	2.57946451	0.00551106	0.01651262
EC_2.1.1.266	6.30990492	1.21873693	4.04971769	0.69638196	1.49604087	1.54522156	3.15952099	0.00553275	0.01653933
EC_1.3.1.1	2.75173755	1.31562414	1.93936565	0.25702249	0.27180069	0.71550855	1.82141358	0.00554057	0.01653933
EC_5.3.3.18	6.28899133	2.04344153	4.31881779	0.75136051	1.67600986	1.63033937	3.37375017	0.00556782	0.01658112
EC_3.5.1.14	5.09086955	2.00256557	3.90446057	0.64783104	0.87512067	1.57574351	3.00735344	0.00558064	0.01658112
EC_4.1.1.23	0.10542601	0.0634514	0.1828521	0.43107848	0.56606784	1.01598857	-0.4521115	0.00558555	0.01658112
EC_1.8.4.13	6.03358823	2.4643079	4.44585398	0.74601423	1.78754872	1.6699243	3.36587478	0.00562529	0.0166683
EC_1.17.1.9	7.33515698	4.17419225	3.82705234	1.01036859	1.50579751	0.9001014	4.62722994	0.00564324	0.01669068
EC_1.2.99.7	-1.5577505	-0.2521482	2.21544946	2.44172586	1.82000298	5.32338659	-3.2491623	0.00566522	0.01670621
EC_3.6.1.61	-3.0909788	-0.6587725	2.04687976	0.15253504	1.74872464	4.46667755	-2.8422772	0.00566929	0.01670621
EC_2.2.1.10	-2.4772749	-0.0163518	1.47979811	1.51493402	1.67006805	4.66409155	-3.100005	0.00576923	0.01696957
EC_6.1.2.1	-0.8675565	-1.2594137	0	0.32371153	1.65766096	1.10770565	-1.764094	0.00581685	0.01707836
EC_2.7.1.218	6.08852273	2.79860412	4.43875628	0.75400454	1.80959197	1.70056047	3.49658355	0.00583143	0.01708993
EC_2.7.7.39	-0.3865821	0.44875523	0.44168153	1.76377773	1.94525747	4.52632911	-2.1752906	0.00584926	0.01711096
EC_2.7.1.89	6.35541558	4.17589461	4.71351207	0.77650569	2.40870969	2.91989048	3.78898536	0.00592931	0.01731359
EC_2.1.1.265	6.33095488	2.03623053	4.13927548	0.73505264	1.7479941	1.65457849	3.35317868	0.00599399	0.01746951
EC_2.1.1.297	-0.0283185	0.03130601	-0.2089566	0.45500094	0.74015422	1.53977057	-0.7203094	0.00600446	0.01746951
EC_6.1.1.9	0.15712748	0.041842	0.27365209	0.48633867	0.60438458	1.09517746	-0.4744316	0.0060592	0.01759687
EC_7.4.2.1	6.19487348	2.12366428	6.24223798	0.7826871	1.79417922	1.75871961	3.5161188	0.00611915	0.01773892

EC_6.3.2.1	0.349611	-0.1658373	0.08489908	-0.0385921	-0.436502	-0.5454555	0.37022886	0.00619552	0.01792795
EC_6.3.4.2	0.05224789	-0.0744469	-0.1217058	0.18192999	0.17986998	0.42812717	-0.227537	0.00631366	0.01823695
EC_6.3.2.34	-5.0101363	-1.2349579	1.35069834	0.71598617	0.23707973	4.80267038	-3.989017	0.00633267	0.018259
EC_5.4.99.28	4.41563034	1.90665844	4.57260425	-0.2845692	-0.12668	2.05318406	3.4719638	0.00640661	0.01843911
EC_1.12.7.2	0.1742936	-0.0611841	-0.196542	0.89246817	0.27436904	1.22388658	-0.6878863	0.0064414	0.01850606
EC_2.7.1.31	2.99891948	2.03407521	6.33949453	-2.4268048	1.7670937	6.10774984	2.99737165	0.00650646	0.01865959
EC_3.1.2.12	9.01498383	5.62090755	5.16570988	3.2543997	2.81921034	4.08550659	4.12015426	0.00654395	0.01873365
EC_4.2.1.20	-0.1567199	0.0504345	-0.1767567	0.21171266	0.13796205	-0.0549563	-0.2383089	0.0065677	0.01873441
EC_2.4.2.18	-0.1781641	0.47240007	0.44134546	0.64377751	1.17000418	1.52677792	-0.8040974	0.00657493	0.01873441
EC_2.3.1.46	5.58456781	3.83882866	5.82712563	1.93865802	2.6308394	0.41085342	3.03098805	0.0065801	0.01873441
EC_1.2.1.79	6.9617792	3.3038965	5.16102569	1.29004069	0.87135929	0.83667898	4.35223165	0.00659087	0.01873441
EC_1.1.1.81	6.11639852	3.01033609	4.37176661	1.07932872	1.69413815	1.6751301	3.44322573	0.00670782	0.01903314
EC_2.7.7.n1	6.27764201	2.12675697	6.1511382	1.11027969	1.80160718	1.71276493	3.37580388	0.00672162	0.01903866
EC_2.1.1.133	-1.3430068	0.35561282	0.24738179	0.31692322	1.09249799	3.00721205	-1.470987	0.00681509	0.01926943
EC_4.2.1.53	-0.4752362	0.35277803	0.18006207	0.88491209	1.51592823	2.15887766	-1.3607722	0.0068622	0.01936853
EC_1.3.1.10	5.80711219	2.46307174	4.45548316	0.75016681	1.64778908	1.65529117	3.29362043	0.00687475	0.01936992
EC_4.2.1.115	-2.1744063	-0.0157878	-0.2404932	0.00195857	0.64552284	1.99187437	-1.6471167	0.00689836	0.01939992
EC_4.2.1.75	5.18251803	2.64173877	5.4837978	-0.0098758	1.0572959	0.56130408	3.90443935	0.00691677	0.01939992
EC_2.7.8.42	5.40802662	1.62312265	3.91677321	0.65191445	1.35325025	1.51633284	2.88922817	0.00692164	0.01939992
EC_4.1.1.3	-1.2576226	-0.4931658	-0.6582573	-0.1228715	0.55534017	1.98664982	-1.2899551	0.00704205	0.01970301
EC_3.5.3.6	0.45065548	-0.0935361	0.55787375	0.79794268	0.73949655	1.65934945	-0.6208662	0.00707537	0.01976183
EC_3.1.26.12	6.36355682	5.62760898	8.17730945	1.73337346	2.73493092	3.25278459	4.0638581	0.00713018	0.01985855
EC_5.3.2.3	-2.2845586	-0.3515618	-2.8466623	0.46943987	3.00284348	1.3816179	-3.0887246	0.00713474	0.01985855
EC_3.4.23.36	-0.0170709	-0.0385971	0.1159759	0.38804036	0.49223094	1.02674724	-0.5144767	0.0072407	0.02011863
EC_5.3.1.4	-0.019261	0.47662486	0.71024946	0.50047073	0.96073803	1.60609948	-0.5508028	0.0074028	0.02051671
EC_2.7.2.3	0.0651593	-0.039083	-0.2824187	-0.1182284	-0.3342969	-0.473959	0.22143152	0.0074196	0.02051671
EC_4.7.1.1	4.68882833	1.53071225	3.78703469	0.67699747	0.96714377	1.19100299	2.62005251	0.0074223	0.02051671
EC_1.1.1.281	-2.0019674	-0.4304134	0.07563139	1.53228198	0.56358611	0	-2.2040861	0.00750842	0.02071911
EC_2.1.1.222	6.71430649	4.07991062	4.72012623	1.69976656	1.8079999	1.82427656	3.78517539	0.00752626	0.02072528
EC_6.5.1.4	6.83573916	1.97866768	7.56161601	0.84377409	2.04723777	1.98457523	3.8161219	0.00753646	0.02072528
EC_2.3.1.210	5.0735937	1.75797755	4.00308064	0.67002692	1.43575221	0	2.93488549	0.00756061	0.02075613
EC_2.7.7.82	-3.1958695	-0.4776791	-0.3683513	0.55688567	1.89525752	5.26173743	-3.5001705	0.00769681	0.02109399
EC_1.14.11.47	4.49990264	1.54938905	3.37321616	0.58797957	1.24064521	0.92697498	2.47022859	0.00778263	0.02129285
EC_2.1.1.11	-3.0753959	-0.0984121	2.44128446	0.97088197	0.88030797	8.30209922	-3.2488101	0.00781126	0.02133485
EC_1.17.99.6	0.2094861	0.48293956	0.68870947	0.88452034	0.95822749	2.29551881	-0.7289212	0.00786764	0.02145233
EC_3.5.3.1	3.92107881	4.67313745	5.66980275	0.3808331	0.79450327	2.0402878	3.66737559	0.00788666	0.02146775
EC_2.7.1.199	4.34195673	1.09047857	3.44728723	0.59222132	0.79301751	0	2.50034474	0.00795285	0.02161129
EC_4.1.1.112	7.07190494	1.34585282	4.51273726	0.73293202	1.76255063	2.85624069	3.38066528	0.00801561	0.02174505
EC_2.7.4.1	0.11615676	0.57421895	1.00496344	0.90274905	1.51609332	2.49203944	-0.925251	0.00805481	0.02181453
EC_2.8.3.9	2.60493279	2.66740395	4.68318102	0.11389643	-0.5266211	0.04590773	2.98684182	0.00810907	0.02190743
EC_1.1.1.373	5.17182943	1.96060229	4.11190919	0.69489239	1.59072705	0	3.00713177	0.00811639	0.02190743
EC_6.3.4.20	0.42683001	-0.0547821	0.54928493	0.04832861	-0.322179	-0.689814	0.44135563	0.00815747	0.02198136
EC_4.4.1.24	-3.46152	-0.940588	2.73129864	1.04386355	0.87476261	4.19039925	-3.2112355	0.00822225	0.02210236
EC_1.1.1.125	-1.9167284	0.19842945	0.89587569	0.93916359	1.35549887	1.96732612	-2.0335198	0.00822989	0.02210236
EC_2.1.1.37	-0.1131655	0.01471802	0.36915918	0.18622919	0.25491759	0.45533443	-0.2528468	0.00827139	0.02217671
EC_7.6.2.2	5.54652735	1.15872774	3.8055438	0.66325497	1.31117915	1.28923615	2.82106287	0.00831226	0.02222387
EC_4.2.1.19	-0.051005	0.46608724	0.55129331	0.63034231	0.98567524	1.3910285	-0.6407886	0.00831665	0.02222387
EC_3.5.1.4	8.57343487	4.63440987	3.2480346	2.80473158	3.0711335	4.08584997	3.46517628	0.0083418	0.02225404
EC_2.6.1.22	5.40430225	1.73585729	4.10070097	0.69329695	1.53851912	1.42236636	2.87896697	0.00836866	0.02228866

EC_5.4.99.20	6.89658971	2.14628228	4.68508336	1.13405381	1.79520948	1.82636303	3.50120595	0.00843421	0.02242606
EC_1.1.1.27	-0.6006179	0.05853789	1.51107811	0.17008823	1.3528573	2.67744474	-0.9955383	0.00848936	0.02253538
EC_2.7.13.3	0.28478249	0.34763566	0.86339722	0.80501544	1.10449089	2.19602174	-0.7079355	0.00852058	0.02254891
EC_3.1.3.4	4.90202853	1.87608606	4.01962679	0.68513542	0.90878845	1.28782362	2.88446329	0.00855472	0.02254891
EC_3.1.3.81	4.90202853	1.87608606	4.01962679	0.68513542	0.90878845	1.28782362	2.88446329	0.00855472	0.02254891
EC_2.1.1.156	0.69274966	0.00038463	-0.2649798	2.11885086	3.8296135	7.70316487	-3.0830901	0.00857662	0.02254891
EC_1.1.1.381	4.78050601	2.20778863	3.67970535	0.64112527	1.3462989	1.23475085	2.78401531	0.00858162	0.02254891
EC_2.7.6.3	2.07045208	1.45103803	2.49564601	0.02198433	0.84730756	1.04885286	1.4757017	0.00858277	0.02254891
EC_7.2.2.8	5.68675437	2.15064928	4.23910256	0.72410114	1.6533338	1.60464558	3.11239306	0.00859273	0.02254891
EC_2.3.1.191	5.60987653	4.2180637	8.24048057	0.75418505	1.82955182	2.06232867	4.16072302	0.00864557	0.02263608
EC_2.4.2.4	6.54427007	3.52955308	5.25727781	0.4943447	1.40376104	2.35107675	4.28232292	0.00865414	0.02263608
EC_2.4.2.21	0.04451299	-0.2560399	1.28309138	0.58927171	0.46952641	1.59495447	-0.592222	0.00873893	0.02280734
EC_2.7.7.73	6.86815272	2.57817545	7.87154483	1.21847966	1.56672057	0.85493235	4.1867068	0.00875473	0.02280734
EC_4.2.2.7	-2.2025419	0.46489958	-0.5006778	0.8606406	1.2645862	4.30432212	-2.4945101	0.00876222	0.02280734
EC_3.5.3.26	5.15319871	1.18579061	3.78684258	0.64233207	0.87262867	1.40215983	2.76283702	0.00887437	0.02306188
EC_2.7.1.195	5.17906167	1.17178274	3.80512897	0.65621641	0.87137395	1.3785013	2.7696595	0.00890618	0.02309019
EC_2.4.1.64	4.65163089	1.02850363	2.96249851	0.49077918	1.26632214	1.19144064	2.32419006	0.00891553	0.02309019
EC_2.7.1.196	4.48420726	1.21618024	3.44984017	0.64375592	0.83652509	0	2.57747705	0.0089284	0.02309019
EC_2.6.1.98	-3.7037798	0.68419121	-0.147198	-0.0748122	1.06473914	2.84914269	-2.3965024	0.00897319	0.02316418
EC_2.7.2.2	6.41421274	3.02899349	10.5432372	1.95964744	3.47254377	4.06041249	2.98962686	0.00898586	0.02316418
EC_2.1.1.298	6.54973022	1.3426752	4.5067184	0.73672374	1.80972928	1.56045411	3.25824393	0.00904049	0.02326766
EC_2.7.4.29	5.53125741	1.24228268	4.05970223	0.44424218	1.1560112	1.59726071	3.0026798	0.00910774	0.02340325
EC_2.6.1.9	-0.0555594	0.41782772	0.79433246	0.65499032	0.996349	1.48240179	-0.6593058	0.00927962	0.02375734
EC_1.3.8.7	5.68590594	1.85657595	4.13280999	0.70059786	1.59201472	1.44349163	3.04874178	0.00928992	0.02375734
EC_1.3.8.8	5.68590594	1.85657595	4.13280999	0.70059786	1.59201472	1.44349163	3.04874178	0.00928992	0.02375734
EC_2.6.1.50	-0.7276693	0.41242163	0	1.08386098	3.47167616	6.1563887	-2.7676785	0.00931653	0.02377773
EC_1.14.12.18	5.71867602	2.05972027	6.68716101	0.73745282	2.26732123	1.66059662	3.18104641	0.0093275	0.02377773
EC_4.4.1.11	-1.6561905	-0.0966007	0.27308654	0.11046455	0.83191103	2.46098167	-1.5230475	0.00935997	0.02382267
EC_1.17.5.3	5.73146895	1.19678889	3.99007529	0.67521188	1.36409056	1.53724058	2.89754227	0.00942091	0.02391597
EC_2.1.2.5	0.55727069	0.48450746	-1.0762136	0.02597868	-0.6343466	-1.1055265	0.67043254	0.00942641	0.02391597
EC_6.1.1.22	0.1507507	0.00241477	-0.0329543	-0.0175401	-0.2435106	-0.2691382	0.20037274	0.00950072	0.02404974
EC_3.5.1.n3	6.46771382	1.35681881	4.32717291	0.74430606	1.70613481	1.63447968	3.21847951	0.00950908	0.02404974
EC_2.4.1.325	6.11941564	1.26770659	4.14470816	0.70204469	1.6331761	1.62288974	3.03060934	0.00986219	0.02490359
EC_4.3.3.7	-0.0571914	-0.0472945	0.46563258	0.29165504	0.59872413	1.34172843	-0.519858	0.00997697	0.02515387
EC_1.3.1.25	5.77677142	2.52679566	4.26482806	0.73061968	1.73087754	2.70742696	3.12209242	0.01013395	0.025461
EC_1.4.1.14	-3.0923967	-0.5630881	0.77774019	0.70689263	0.66429237	7.38946763	-3.2927414	0.01013794	0.025461
EC_6.1.1.15	0.10459736	0.01528161	0.15238877	0.28261085	0.34249966	0.73592283	-0.2829643	0.01014635	0.025461
EC_3.6.3.31	0.22685319	0.43392311	0.58420004	0.7232486	1.17557537	2.33082834	-0.7386997	0.01020685	0.02557285
EC_3.6.3.8	0.48044718	-0.2660276	-0.6442371	0.8689157	1.30270757	1.55359453	-1.02288	0.01038139	0.02596322
EC_3.1.31.1	5.91692626	3.16337029	5.16674077	0.75120005	0.86420773	2.06898811	3.88834407	0.01039499	0.02596322
EC_1.2.1.91	6.20187672	1.3256789	4.25265535	0.74340992	1.70858345	1.5591883	3.06879157	0.01053859	0.02624027
EC_3.3.2.12	6.20187672	1.3256789	4.25265535	0.74340992	1.70858345	1.5591883	3.06879157	0.01053859	0.02624027
EC_4.1.3.27	0.2997301	-0.2237146	0.5418284	0.93185486	1.39428332	2.67914139	-1.1689633	0.01061937	0.02640049
EC_1.3.98.1	1.14447597	0.93181602	1.9854032	-0.9987552	0.74228455	1.72411976	1.24248723	0.0108205	0.02685891
EC_2.3.1.202	-1.4432663	-0.6430112	2.13739334	0.0634675	3.47369525	7.09179744	-2.8256797	0.01087973	0.02696427
EC_5.4.99.2	0.22605132	-0.2699783	-0.938465	-0.2969077	-0.9846102	-1.1656305	0.54412335	0.01093049	0.02704832
EC_1.2.1.21	5.49172353	1.92334804	4.08788432	0.70827738	1.56412476	1.50172222	2.96262783	0.01098626	0.02713151
EC_2.7.7.2	-0.1620312	-0.1519764	1.04991148	0.31739287	0.52179672	1.26434443	-0.5187092	0.01099789	0.02713151
EC_2.7.4.16	0.29293933	-0.2643788	-0.2090946	-0.2164598	-0.6302582	-0.8231774	0.46583083	0.01102864	0.02714576
EC_3.4.22.71	-2.0269424	-0.6390804	0.7210272	0.14386679	0.75934915	2.77217311	-1.8894207	0.01105099	0.02714576
EC_7.5.2.1	6.54986308	1.600575	4.55190261	0.76740196	1.85264688	1.73995392	3.30096266	0.01105438	0.02714576

EC_3.1.3.104	5.23340591	1.79533862	4.22232354	0.71640322	0.97049149	1.65700821	2.96846671	0.01126204	0.0276135
EC_1.5.99.8	3.48461907	2.24076854	2.92108493	0.89062893	0.9754051	0.93291885	2.05099609	0.01128162	0.02761932
EC_3.5.4.42	4.73775678	1.24082638	4.05387574	-0.0792548	0.3643531	1.52556438	3.1383532	0.01137133	0.02779659
EC_1.2.1.46	4.80485579	0.50690205	3.66258982	-0.4161732	0.28449342	2.52255928	2.94298799	0.01151016	0.02809318
EC_6.3.2.12	1.34997692	1.07354316	5.75373202	-4.3927523	1.61130668	5.65832856	2.8958028	0.01155735	0.02816556
EC_2.7.7.81	-1.78749	0.102543	-1.0796617	0.08983013	0.4438959	2.03275967	-1.4923857	0.01165443	0.02835843
EC_2.7.8.8	2.26290027	1.39137782	1.21030097	0.29478371	-0.3824704	0.57758875	1.71533766	0.01167181	0.02835843
EC_4.2.1.149	5.84697437	2.36525281	4.40564112	0.99470907	1.79134499	1.65490328	3.09445991	0.0117271	0.02844224
EC_4.2.3.12	6.47464887	0.88034736	6.62471894	0.71840385	1.65355228	1.69280995	3.35853367	0.01175032	0.02844224
EC_2.5.1.97	-1.7661979	0.05058371	-0.2744885	0.1227798	0.58766763	2.22838141	-1.4916135	0.01176197	0.02844224
EC_2.3.1.n3	-0.1910256	-0.0672767	-1.125864	0.30760831	0.14363696	1.12933093	-0.6235371	0.01177714	0.02844224
EC_4.4.1.21	0.18656521	-0.0349935	0.97976376	0.44084009	0.79755331	1.55885244	-0.4993297	0.01184535	0.02856401
EC_1.8.1.7	2.1671688	0.89832749	4.43468706	-3.8913784	1.69553501	5.70448712	2.7948447	0.01187067	0.02858216
EC_3.1.2.28	5.7090734	2.40640472	4.41706108	1.11753678	1.03247637	1.62949657	3.22402789	0.01195145	0.02873358
EC_1.3.1.28	7.99719287	0.94599355	7.47474883	0.93413563	2.63725898	2.0121266	3.80146388	0.01201391	0.02883775
EC_4.2.99.18	0.03021781	-0.0416913	0.30082798	0.2415568	0.43678488	0.95381158	-0.3611996	0.01203069	0.02883775
EC_2.3.1.n2	0.10597159	0.1019589	-0.7607417	0.5498518	0.2429231	1.31993719	-0.5520295	0.01207031	0.02888959
EC_1.5.1.12	-2.0585693	-1.208308	0.17395566	0.67943054	0.86669164	5.27414976	-2.8280507	0.01209547	0.02890674
EC_3.5.1.2	0.0469305	0.14245602	0.25621193	-0.1500365	-0.0571749	-0.527216	0.27554067	0.0122608	0.02922397
EC_2.7.1.208	5.74263665	1.27286914	4.10952256	0.70789299	1.62850947	1.44703226	2.85244571	0.0122646	0.02922397
EC_1.2.4.1	2.45054688	1.65079499	6.30749575	-2.9822566	1.98015155	5.72743153	2.8512234	0.01236609	0.02933999
EC_1.17.1.8	0.11150247	0.19281586	0.07748409	0.5460345	0.69856827	1.42540985	-0.5754081	0.01236658	0.02933999
EC_4.2.1.46	0.2250649	0.20352716	0.46706819	0.54414267	0.92241422	1.60281987	-0.5601198	0.0123681	0.02933999
EC_2.3.3.5	7.88338112	3.01186866	7.21759948	2.19088948	1.68572947	2.10837939	4.06187841	0.01255505	0.02973955
EC_3.3.2.1	6.88295789	1.45345616	6.83658957	0.81908893	1.91016359	3.05945501	3.48306083	0.01274838	0.03015302
EC_2.1.1.33	-0.0153341	-0.1542546	0.47731079	0.18569834	0.44987723	0.8535892	-0.3641967	0.01289785	0.03045532
EC_4.3.1.14	-0.7045174	-1.2229715	-0.3260825	2.20710972	-0.1166395	2.38305891	-2.3100361	0.01291412	0.03045532
EC_4.1.3.17	6.68590551	1.34585282	4.51273726	0.73293202	1.76255063	2.85624069	3.17869749	0.01297031	0.03054299
EC_3.5.4.13	6.16208813	3.89614463	7.50951015	2.16087311	1.41112714	1.98282649	3.63299198	0.0130454	0.03067483
EC_3.4.21.92	-0.0221389	0.02165742	-0.0485248	0.16601256	0.17702334	0.35246186	-0.2039705	0.01307181	0.03069199
EC_3.6.3.21	0.10764686	-0.4956802	0.00017681	2.49994399	1.60803231	7.27872004	-2.9543295	0.01319873	0.03090748
EC_2.1.1.185	4.52455553	2.29690473	6.43755463	0.41829075	1.42183316	3.69952212	2.79170492	0.01320207	0.03090748
EC_3.1.4.46	2.4053892	1.92435111	5.94234247	-0.4000536	1.37461692	3.33431085	1.99621266	0.01359834	0.03175863
EC_2.1.1.172	6.47381032	4.77535691	7.46757505	1.73692245	3.5132247	0.58631315	3.8257686	0.01360519	0.03175863
EC_2.2.1.6	0.19397794	0.3946861	1.06932016	0.79663055	1.1153757	2.11977319	-0.7025636	0.01367455	0.03187421
EC_3.5.2.5	9.43313077	4.0993225	4.7742468	3.14389015	1.96666369	6.59433172	3.76183051	0.01377318	0.03205758
EC_5.3.1.12	-0.255272	-0.2304734	0.04297354	0.15226753	0.08275539	0.85873661	-0.4321916	0.01393685	0.03239158
EC_4.2.1.44	0.53752028	0.43291938	0.6457571	1.12035482	1.07798309	2.28591292	-0.7458021	0.01404466	0.03259498
EC_2.4.1.305	-1.318412	0.92218628	2.67790896	0.4419087	3.49943555	6.77296149	-2.3109251	0.01408751	0.03264723
EC_3.5.3.12	0.15183829	0.91301853	2.23758535	1.7574762	2.61526519	5.31269374	-1.8285736	0.01443215	0.03339774
EC_2.4.1.293	-3.1852133	-3.6156899	-4.8890932	-0.1485084	-2.6725784	0.53097692	-2.6596223	0.01452533	0.033565
EC_2.4.2.3	0.13675374	0.2667433	0.03772583	0.84170105	0.803846	1.92915649	-0.8014503	0.01456301	0.03360372
EC_1.2.1.3	5.49186604	3.13379961	7.20823134	1.27353014	1.93574111	1.14591312	3.39310283	0.01465469	0.03376675
EC_6.3.4.15	0.16878399	0.12027535	0.49756572	0.70891782	0.82271163	2.06355621	-0.7322411	0.01474079	0.03387314
EC_6.3.1.8	3.82614298	1.65247198	1.44452896	0.48171311	1.20384853	1.06257781	1.96953516	0.01474304	0.03387314
EC_6.1.1.10	-0.0245075	-0.055753	0.00797184	0.14456497	0.1711099	0.27936093	-0.2025661	0.01479702	0.03394858
EC_5.4.99.18	0.18852214	0.05474495	-0.0647821	0.38716238	0.55603735	0.98549442	-0.4110261	0.01492582	0.03419524
EC_2.1.1.n11	5.13039735	1.1606939	3.72273261	0.63602849	1.41466514	1.22878447	2.58260603	0.01500851	0.03433571
EC_1.4.3.19	3.66805936	5.4354804	6.31343257	1.64728409	1.16164093	-0.9334271	3.46920798	0.01514374	0.0345498
EC_5.4.99.26	4.02325098	1.82647175	4.01306492	0.33626832	-0.4976172	1.82695502	2.98528663	0.01514512	0.0345498
EC_1.5.1.5	0.03237463	-0.0839705	-0.0896411	-0.1008914	-0.2143871	-0.5610273	0.17474814	0.01555767	0.03544059

EC_4.2.1.9	0.2028749	0.2789953	0.85894718	0.61361757	0.91262576	1.57467937	-0.5276325	0.01582757	0.03600436
EC_6.3.2.45	4.49194406	1.91778921	7.70651653	0.05268065	0.59418844	0.70121549	3.65876564	0.0161563	0.03670018
EC_3.4.13.22	0.80182298	0.66959198	1.31965386	0.04042106	0.1002514	0.58308864	0.68714563	0.0162538	0.03686949
EC_1.13.12.16	-0.0399607	0.13343357	-1.0262286	0.44587874	0.12302888	1.05504814	-0.5194504	0.01630003	0.0369222
EC_1.1.1.86	0.16251473	0.18824588	0.82657276	0.47177201	0.85389645	1.40649755	-0.466811	0.01646229	0.03723221
EC_1.3.8.13	4.67375231	1.20232634	3.84752716	0.65451319	0.88338667	1.33920509	2.52342298	0.01648325	0.03723221
EC_2.1.1.107	0.29410009	-0.8438198	0.92321684	1.28515073	1.93304512	3.75975313	-1.8544358	0.01665584	0.03756921
EC_1.2.99.5	-2.4879968	0.89928184	-2.6677579	0.61833204	1.45311297	4.49432244	-2.7113626	0.01669211	0.03759822
EC_1.1.1.350	4.65790692	1.07730941	3.65565878	0.62832084	1.47483935	0	2.4434462	0.01675428	0.0376854
EC_6.3.2.4	0.08974369	-0.0200699	0.17060547	0.26677198	0.37559743	0.64521447	-0.2913993	0.01683518	0.03777626
EC_7.2.2.6	4.74593063	1.18662268	3.84396867	0.65878326	0.89477832	1.39263613	2.54212896	0.01684172	0.03777626
EC_2.7.1.35	0.54873761	0.51105199	0.98755931	0.93480434	1.35592043	2.2631919	-0.6581758	0.01697894	0.03803094
EC_1.1.1.50	-1.5198577	2.55406813	2.36388575	2.46160762	2.23790543	6.39549582	-2.505767	0.01707154	0.03818508
EC_2.7.1.193	4.56226015	1.15833798	3.73082844	0.64461915	0.87447518	1.30309355	2.4482176	0.01714146	0.03828816
EC_4.3.1.2	-1.4815485	-1.9363604	-2.7854828	1.39830981	-0.5974912	2.71853866	-2.719745	0.01739255	0.03879504
EC_4.2.1.85	-0.1171468	0.62472852	0.98354695	0.87945567	1.28042443	3.16074296	-1.0300099	0.01753112	0.03904989
EC_4.2.2.9	5.42424097	2.83914309	4.73052102	0.82788391	1.92033969	1.88342104	3.0988474	0.01762203	0.03919803
EC_7.6.2.5	5.03515897	1.2281032	4.01205881	0.69514964	0.94599317	1.54399838	2.6724195	0.01764793	0.03920136
EC_3.2.2.20	0.77199451	-0.1836053	0.70321026	0.97728865	1.11352435	2.36679561	-0.7793592	0.0177255	0.03930436
EC_3.1.26.5	-0.0351895	0.13996772	1.20734289	0.3634763	0.97496296	1.61328623	-0.5489797	0.01774325	0.03930436
EC_2.1.1.64	5.26762107	5.23283114	4.76812256	2.45696472	1.87091895	-0.7621105	3.35204997	0.01782478	0.03943056
EC_3.2.1.1	0.73792018	0.14085123	0.02515991	0.01866475	-0.1694376	-0.5138763	0.5520322	0.01800207	0.03971343
EC_4.2.1.120	-1.610625	0.12281384	-0.2183279	0.65026943	0.88871577	3.27376398	-1.8990024	0.0180021	0.03971343
EC_1.13.11.24	7.08362028	1.70195071	0.70383911	0.41129955	1.86573913	2.08522976	3.28743289	0.01804108	0.03974481
EC_2.3.1.222	0.20202538	0.92513485	1.45777665	1.11140529	1.09082416	2.62461501	-0.6913291	0.01806937	0.03975261
EC_3.2.1.180	-1.8096108	0.23680318	-0.0828259	1.05510665	0.0874954	0	-1.4723824	0.01811167	0.03979116
EC_2.3.1.243	5.75869961	1.35786494	4.30998295	0.74279548	1.61076115	1.64675342	2.87624092	0.01834839	0.04025618
EC_3.5.4.43	-1.4582315	-0.0469014	2.72656966	0.283292	2.42980199	5.65836059	-2.138479	0.0184825	0.04048786
EC_2.7.7.42	6.61124764	6.19898155	11.7963116	1.8638858	5.33134082	4.71899967	3.72491068	0.01850441	0.04048786
EC_1.3.3.3	7.61088198	2.48595425	5.02337466	1.38388927	2.61705829	2.02853846	3.60558469	0.01876268	0.0409971
EC_7.2.1.1	0.18943389	-0.2640822	-0.2666704	-0.2331832	-0.5940562	-0.9718953	0.42142621	0.01880394	0.04103142
EC_1.5.1.7	-2.4133074	-0.1369267	0	0.20187518	2.18690193	3.00230963	-2.5317764	0.01895774	0.04123337
EC_5.4.99.12	0.02952498	0.01648667	0.45147827	0.36308485	0.53103807	1.12569247	-0.4416885	0.018968	0.04123337
EC_2.8.4.4	-0.0840236	-0.2012987	-0.3283022	0.28234639	0.02156001	0.53564709	-0.3856052	0.01897351	0.04123337
EC_3.2.1.68	-0.204211	1.19291472	3.79053793	1.69830117	4.83927126	5.51916546	-2.4497536	0.01940064	0.04210463
EC_6.3.5.1	-0.1782676	-0.1938192	-0.6475784	0.19989777	0.19806809	0.49589966	-0.479471	0.0197822	0.04287478
EC_5.3.3.14	6.42581796	1.41529549	4.57976093	0.7909435	1.85816416	1.72149489	3.1617314	0.01996745	0.04321795
EC_2.5.1.55	0.43974178	-0.1461294	-0.3627153	-0.0096256	-0.587915	-0.6264653	0.41412921	0.02007549	0.04339332
EC_2.4.2.8	0.03728029	0.08571467	0.23066774	0.27483845	0.58736175	1.25004167	-0.4272531	0.02034671	0.04392044
EC_1.3.1.84	4.75842728	1.6594732	3.9391117	0.65035618	1.50983143	1.54309613	2.51093455	0.02056166	0.04432487
EC_2.7.1.90	-0.4111186	-0.0130879	-0.397174	-0.4755744	-0.733057	-1.3969199	0.41257955	0.02072913	0.04459948
EC_4.1.2.22	-0.0959798	1.2084446	3.8400005	1.78498854	4.8912236	5.40257232	-2.4301604	0.02074459	0.04459948
EC_2.7.1.176	5.78034831	1.25990117	4.09243859	0.6961815	1.5480556	2.59769442	2.74708284	0.02114611	0.04540195
EC_1.1.1.244	-2.5460555	3.63055587	2.4992397	3.70922288	2.80699138	0.26266821	-2.7006477	0.02121017	0.04547868
EC_2.3.3.16	7.23944997	2.001377	2.61424702	1.2220401	1.97062912	1.88189139	3.26306754	0.02151881	0.04607894
EC_2.8.1.13	-0.1842529	-0.221447	0.16635285	0.1236075	0.11887374	0.33055687	-0.3036892	0.02174408	0.04649933
EC_1.3.8.1	0.46018596	-0.0839733	0.27729656	1.0936165	0.37409523	1.05282112	-0.6073006	0.02191798	0.04677639
EC_4.2.2.8	-2.334725	0.41717046	-0.0094625	0.49670194	-0.0595164	0	-1.3240895	0.02193189	0.04677639
EC_3.2.1.38	-0.1408074	1.25174431	3.9099361	1.73948814	4.95687244	5.50139044	-2.4396063	0.02200083	0.04686119
EC_2.4.1.54	-0.0096705	0.28979733	-0.8896773	0.55784178	0.86594586	1.42104523	-0.7836957	0.02219245	0.04720672

EC_3.4.21.107	2.49619054	0.68555652	1.41865704	0.66081498	0.26961619	0.57764409	1.20117604	0.02223993	0.04724515
EC_2.7.7.89	6.10068714	5.9273602	11.3715387	1.46566473	5.20616815	4.6796464	3.57105453	0.0222827	0.04727346
EC_2.3.3.13	0.16258036	0.31711432	1.15229316	0.62337652	1.02908116	1.68365205	-0.5570343	0.02282612	0.04836247
EC_3.5.1.110	4.6843672	1.92769045	4.16696523	0.72790225	1.52445548	1.49630108	2.55452978	0.02291064	0.04847759
EC_2.5.1.90	3.89202749	0.96441031	3.99390826	-0.0962452	-0.5214672	1.93495965	2.83646146	0.02310402	0.04882244
EC_2.4.2.19	0.07250654	-0.2886576	-0.8510645	-0.4229788	-1.1932531	-1.0206676	0.58542997	0.02337835	0.04929577
EC_1.2.1.11	0.38764594	0.08403834	0.30323228	0.00315041	0.04379955	-0.1446215	0.2726903	0.0233894	0.04929577
EC_3.6.3.25	0.71743501	-0.1965312	0.59679322	1.07477859	0.61021223	1.49150737	-0.5982573	0.0243436	0.05123962
EC_2.7.7.47	-0.555797	-0.5157489	0	1.19531964	1.13675402	4.15905746	-2.0388464	0.02443986	0.05137489
EC_1.1.1.157	-0.1352269	0.82000597	0.8174522	0.64286363	1.08590283	2.43029913	-0.7030036	0.0245141	0.05146359
EC_3.5.99.2	0.36253566	0.69708841	0.98992682	1.09379127	1.99833349	2.89579718	-1.0691222	0.02472367	0.0518358
EC_3.4.21.105	2.36557036	1.4143097	2.26149519	0.70121622	0.43390819	1.11506332	1.34795917	0.0247592	0.0518426
EC_1.2.1.27	6.3820809	2.84713218	2.77280789	1.84119278	1.93024867	2.31316858	2.75524501	0.02485898	0.05198375
EC_3.1.21.3	0.05541911	0.14814219	0.27687239	0.41377182	0.37592565	1.10175128	-0.376313	0.02492525	0.05205456
EC_2.8.1.7	0.21632309	0.11169307	0.35577922	0.45225312	0.5662177	1.20805904	-0.3925292	0.0252225	0.05260692
EC_4.2.1.28	4.48728448	3.8937886	5.55821911	1.17188253	1.1878247	2.71868619	3.02807139	0.02526458	0.05262634
EC_7.1.2.2	-0.1433246	-0.0898124	0.04508304	0.02578449	0.12582387	0.18252767	-0.180451	0.02563785	0.05333469
EC_1.3.7.9	7.03769639	3.31226333	8.18723472	2.68601677	1.20131887	3.02258842	3.61565392	0.02593344	0.05387982
EC_6.1.1.13	3.73970555	1.73808378	-1.0677267	0.90704894	0.28448445	-2.681476	2.20988433	0.02615081	0.05425754
EC_4.2.1.49	-0.1544761	-0.2319229	-0.4109337	-0.3564532	-0.969629	-1.6914302	0.51949946	0.02620449	0.05425754
EC_1.1.1.266	0	1.51914817	0	1.53529151	2.97877777	4.85391282	-1.9063747	0.02623841	0.05425754
EC_5.3.3.2	1.46851436	1.08416332	2.91357483	-0.3981974	1.0912599	2.12245726	1.08825354	0.02625038	0.05425754
EC_1.1.99.38	-0.6048969	1.51596789	-2.8337051	1.67283229	2.60462685	5.0761294	-2.5522015	0.02629374	0.05426774
EC_2.3.2.3	-0.2168271	0.01087919	1.63923874	0.40200267	1.23531353	3.09428355	-0.9375134	0.0263229	0.05426774
EC_4.1.3.38	4.89345134	3.15591695	7.23989045	1.41748285	0.09857712	4.17500055	3.21843989	0.02683649	0.05513564
EC_1.1.1.23	0.11439709	0.3024525	1.26474355	0.63573031	0.98385714	1.59643055	-0.5540524	0.02683815	0.05513564
EC_3.6.5.3	-0.0301873	-0.1483428	-0.823555	0.23361459	-0.0225287	0.81405532	-0.3951363	0.02684687	0.05513564
EC_2.1.3.2	-0.0942838	-0.0947557	0.12385264	0.10601947	0.10480518	0.21877823	-0.188145	0.02696974	0.05531724
EC_3.4.11.10	4.28274054	4.31164818	8.45575271	1.3545772	0.62847939	3.54606121	3.4001448	0.02708951	0.05549203
EC_5.3.1.35	5.11103461	1.24295192	4.05461123	0.69344886	1.51799088	1.52588368	2.53833725	0.0271652	0.05557619
EC_3.5.2.2	9.03313407	3.21524405	4.86931864	2.34018056	3.18880434	4.92314437	3.50441919	0.02741076	0.05600722
EC_2.7.1.40	0.26723256	0.03185887	0.2479918	0.47617699	0.56448173	1.1308648	-0.4096099	0.02758123	0.05628394
EC_3.2.1.55	0.04869973	0.95703994	0.61027427	0.85242053	1.78496152	2.29503736	-0.9083997	0.02792058	0.05690414
EC_2.7.4.25	-0.0451425	-0.1613557	0.50989574	0.19028739	0.48181837	1.06841873	-0.4194843	0.02818526	0.05734634
EC_2.5.1.17	2.10557399	1.46272406	1.87955035	0.76535634	0.43810549	1.34813022	1.11558396	0.02820897	0.05734634
EC_2.1.1.208	-1.9757285	-0.5670638	-1.94971	1.57390311	-0.426044	1.28550809	-2.3538401	0.02848362	0.05783148
EC_4.2.1.47	-0.1384656	0.00488224	0.11800886	-0.1300738	-0.3545712	-0.5617502	0.20412334	0.02857387	0.05794145
EC_6.3.2.8	0.12625127	0.10927438	-0.2250989	0.45924607	0.5200017	1.019057	-0.475516	0.02887174	0.05847164
EC_3.2.1.156	-1.4432075	0.44535156	2.84846953	0.77123409	2.27542208	7.32443354	-2.3733766	0.02908763	0.05883468
EC_1.1.1.411	4.45631677	1.11842807	3.62209152	0.62372236	1.40001048	1.2627712	2.20959511	0.02921236	0.05901264
EC_4.2.1.10	0.15631056	0.07634297	0.19044216	0.35973374	0.55068501	0.92718862	-0.3647031	0.02944325	0.05940218
EC_1.1.1.136	-0.6337838	0.0116348	-0.5551047	0.69039787	0.25600345	0.60876892	-0.9327736	0.0294855	0.05940218
EC_1.2.1.2	5.21918708	3.32241328	6.41643612	2.16362099	-0.0129517	2.88882375	3.14892189	0.02955188	0.05940218
EC_2.5.1.31	4.84359323	-0.2111794	5.84309844	-1.5799237	0.4973364	2.043535	3.59861189	0.02955314	0.05940218
EC_5.1.3.11	-1.1055091	-0.9663673	-1.2905137	-0.2006696	-0.7511906	-0.4610928	-0.663514	0.02970844	0.05963968
EC_7.1.1.7	5.64240105	1.37374962	4.52041611	0.77322179	1.75957781	1.70166257	2.7743465	0.02975922	0.05966706
EC_1.3.1.95	-0.9595213	1.08899816	1.62096549	1.3112646	2.80579247	5.81785462	-2.3108409	0.03011541	0.06030591
EC_3.1.3.73	1.25784545	0.5221279	4.94707068	-1.9276913	1.22097659	2.5499455	1.75661515	0.03027312	0.06054625
EC_1.19.1.1	4.64793606	1.185032	3.90262724	1.0020942	0.88693364	1.46730408	2.30459532	0.03040807	0.06074049
EC_3.2.2.8	5.57397981	3.80023702	4.59910496	1.12916773	1.14267566	3.92549731	3.3253822	0.03073104	0.06130937
EC_2.7.7.43	-2.3837491	-1.2265072	-0.1012478	0.41636763	0.65056939	2.84041964	-2.5056183	0.03082539	0.0614213
EC_2.1.1.14	2.49534613	1.61039709	6.39448098	-2.5544147	2.57380476	4.62054557	2.59036796	0.03111377	0.06191911

EC_2.6.1.52	0.11847591	0.00475826	0.13685802	0.28388996	0.25687949	0.52894529	-0.2267673	0.03156279	0.06269199
EC_3.6.4.13	2.26782485	1.52576377	3.67809369	0.30975287	1.13525956	2.96877046	1.24818471	0.03158021	0.06269199
EC_6.3.2.6	0.06023259	0.00369129	-0.0844738	-0.0386973	-0.18904	-0.2768264	0.14173833	0.03185728	0.06316394
EC_6.1.1.5	0.30246179	0.12145486	0.42994738	0.4657732	0.55857024	1.15396232	-0.3326093	0.03211147	0.0635894
EC_4.1.1.88	-2.2482525	-0.6690389	0.5993415	0.30850074	-0.2087907	0	-1.4382399	0.03217329	0.0635894
EC_3.5.1.3	4.36764932	2.54154798	4.5144467	0.76167015	1.7008627	1.75240594	2.53909077	0.03219065	0.0635894
EC_3.2.1.165	-0.7516112	1.14237709	4.9109026	2.36811279	3.16468699	5.18542248	-2.3860409	0.03436932	0.06780974
EC_4.1.2.29	-1.4429431	-0.2862171	-0.1433956	0.96521202	0.91529692	5.01917145	-2.3552188	0.03445808	0.06786184
EC_1.1.1.94	0.21572498	0.05108321	0.04270899	0.37321583	0.50545032	0.98827154	-0.3611503	0.03448024	0.06786184
EC_2.6.1.59	-0.1052323	0.6880229	0.84274582	0.75528117	0.92808349	2.41495185	-0.7379852	0.03557185	0.0699246
EC_2.7.4.7	1.39541598	0.93893859	2.41430537	0.12755925	0.59298584	1.42340737	0.90825326	0.03579943	0.07028592
EC_4.1.1.50	0.48381428	-0.2240485	0.93580499	0.70837702	1.39977878	3.07422173	-0.957314	0.03588063	0.07035932
EC_3.5.4.19	0.10683744	0.28058863	1.0828868	0.52083217	0.91919515	1.47419207	-0.4886518	0.03604276	0.07059106
EC_2.7.1.162	-1.1726433	0.68955204	0	0.87472573	1.98586489	5.42054852	-2.2054155	0.03613725	0.07068992
EC_3.6.3.42	-1.025342	-0.1113503	4.02037396	1.09102301	3.88151233	4.507308	-2.5378614	0.03667246	0.07164959
EC_2.4.1.83	-1.0856839	-0.2067102	2.64580135	1.07319374	1.74560186	5.19718226	-2.1522909	0.03686743	0.07189353
EC_1.2.1.88	6.76052839	2.94354944	7.58665792	1.22341048	2.2166374	5.51217366	3.39746318	0.03688684	0.07189353
EC_1.2.1.77	5.26605912	1.27081778	4.17346766	1.05745299	1.64206062	1.16915878	2.45321549	0.03750146	0.07300284
EC_4.2.1.118	-1.7436603	0.02868046	4.39856153	-0.4117507	1.98227721	6.25538419	-1.6119333	0.03856503	0.07498238
EC_1.5.1.38	2.08527528	2.03463084	1.71750246	0.86864869	-0.4406895	-1.2538938	1.86170319	0.03869719	0.07514835
EC_2.1.1.178	3.36871273	2.58898126	6.77262857	1.44427711	0.99821567	2.50251167	2.07274297	0.03893712	0.07552296
EC_2.4.1.289	-2.149524	-3.8531005	-9.5383237	0.77269716	-2.7099703	-3.9956671	-2.6630422	0.03914839	0.07584116
EC_2.7.1.63	-0.0537979	1.26050876	3.8882759	1.73966383	4.87751881	4.34947822	-2.21642	0.03934315	0.07612663
EC_4.1.1.44	-0.6428735	-0.4706399	0.19529024	1.7991683	0.54119655	2.42801331	-1.9484085	0.03961609	0.0765625
EC_2.7.1.56	-0.7862787	0.62079508	0.33040558	0.96609335	0.5203149	1.61866249	-1.0566535	0.0400175	0.07724533
EC_1.10.3.10	-0.7210503	-0.5599962	-6.0491125	-0.6490437	-6.1770292	-7.7595181	2.07845664	0.04009073	0.07729378
EC_1.4.3.21	5.615113	0.6162675	4.32351212	0.72211017	1.54728161	1.61776393	2.57590443	0.04022512	0.07745989
EC_2.7.1.33	0.02369828	0.09347824	1.17986584	0.35185468	0.79250045	1.47641746	-0.4539128	0.04056103	0.07801318
EC_1.12.99.6	6.52242495	4.11957948	8.04037109	1.04839804	3.45204159	6.91679762	3.25011875	0.04064401	0.07807927
EC_1.3.99.22	-1.912935	-0.7964026	1.91774023	2.1604686	-0.044482	5.9056367	-2.9772851	0.04098264	0.07859453
EC_5.99.1.3	0.10829115	0.0294938	0.3264878	0.20753475	0.37076599	0.74468178	-0.2244789	0.0410101	0.07859453
EC_2.7.7.72	0.03646126	-0.1207297	0.11675531	0.26282221	0.33109579	0.58285113	-0.3366141	0.0411775	0.0788213
EC_3.8.1.7	4.42082986	0.89716955	8.45749729	0.6234701	0.19461717	2.82213371	2.8959031	0.04124405	0.0788547
EC_1.1.1.276	-0.5988473	-0.9731106	0.50273023	1.12940853	0.06179961	0	-1.227406	0.04152771	0.07930261
EC_3.4.21.53	0.21719617	-0.1110579	0.05320182	0.36419937	0.32273483	0.65782416	-0.3083379	0.04179303	0.07971449
EC_3.5.2.7	0.07399711	-0.1384931	-0.4690483	-0.1445985	-0.7976067	-1.392047	0.45472492	0.04257699	0.08111346
EC_2.7.1.95	-0.9034169	-0.0063216	2.0034658	0.77225087	3.08235085	1.27772485	-1.8254912	0.04338624	0.08255723
EC_2.7.1.36	1.32711339	0.5283235	4.56723604	-3.423502	0.74231204	4.69104664	2.43313703	0.04344539	0.08257194
EC_2.5.1.19	0.01474549	-0.205026	0.95801194	0.34086126	0.5933245	1.12429771	-0.4740196	0.04369701	0.08295202
EC_3.1.26.3	0.40490335	0.20496769	0.44359848	0.61834748	0.89954327	1.5653383	-0.4960944	0.04393148	0.08320585
EC_5.3.1.9	0.05361121	-0.0193101	0.09460248	0.18513581	0.17989322	0.42448961	-0.1820455	0.04393435	0.08320585
EC_5.1.3.2	0.29894262	0.60070311	0.65939391	0.80411044	0.89726817	2.13074989	-0.5588845	0.04401519	0.08322991
EC_3.4.17.13	2.07125015	1.29556892	1.37900832	0.76442039	-0.0923546	0.48329984	1.26509302	0.0440507	0.08322991
EC_5.1.1.7	-0.0023311	0.2387741	1.39345879	0.43475447	0.9075847	1.61713091	-0.4909349	0.04412022	0.08326331
EC_2.7.7.87	0.01425824	0.21548078	1.06435553	0.44520755	0.88285066	2.18220854	-0.6025991	0.0442443	0.08330241
EC_1.8.99.3	-1.6696606	-0.3237937	2.03801839	1.44864556	0.69560671	3.94372744	-2.2679318	0.04426097	0.08330241
EC_2.3.1.16	1.99854473	1.21932092	4.37311477	-2.972302	2.24865731	5.47771267	2.16417259	0.04429655	0.08330241
EC_2.4.2.45	-1.9125409	1.40678554	2.23717165	1.04660109	1.85962979	7.4259958	-2.379244	0.04468375	0.08385362
EC_2.4.1.248	1.07086026	1.11891033	-0.6769258	0.03136224	-1.5599063	0	1.36818838	0.04469409	0.08385362
EC_3.1.3.3	0.84414481	0.11453683	-1.4050739	0.0963618	-0.7915323	-0.7180129	0.61176981	0.04484954	0.08404709
EC_2.7.1.51	4.93156821	2.82932964	7.82693984	1.46870823	0.69627112	5.23378365	2.83192166	0.04513672	0.08448667

EC_2.7.1.144	0.25113995	0.34513947	1.34861025	0.62621941	1.30560749	2.8653758	-0.7241191	0.04534034	0.08476901
EC_1.1.1.193	0.02122207	-0.122484	1.91236697	0.49666204	0.7657426	1.59487278	-0.5270122	0.04594119	0.08579251
EC_3.5.4.26	0.02162237	-0.1221208	1.91236697	0.49635315	0.7657426	1.59487278	-0.5265071	0.04609634	0.08598226
EC_4.1.3.34	3.55672139	2.61730552	4.13634297	1.10969264	0.9559411	1.59432635	2.17209629	0.04661541	0.08684958
EC_1.1.1.292	-0.7645712	1.16175072	1.49255173	1.70168918	1.42326395	3.70336124	-1.6789025	0.04668872	0.08688537
EC_3.1.1.2	4.89737996	2.04919206	1.88874287	1.07259268	1.59424317	-2.4245826	2.73545201	0.0467498	0.08689836
EC_2.7.6.5	0.34263565	0.18927635	0.8208361	0.58105254	0.97726232	1.58818528	-0.4963125	0.04705186	0.08735871
EC_3.4.21.88	-0.0673704	0.06566705	0.30250166	0.26251303	0.6418406	1.52332265	-0.5273958	0.04768041	0.08842349
EC_2.1.1.199	0.18976945	0.04623712	0.25616639	0.35809252	0.45951414	0.86998624	-0.3115092	0.04781233	0.08856586
EC_2.1.1.170	5.30670113	2.80813892	7.68459411	1.40027208	1.87701147	2.98085125	2.94898474	0.04823598	0.08924767
EC_2.7.1.66	4.90239552	2.33246189	2.69234442	1.34969074	0.34004221	1.13318365	2.73105213	0.04900551	0.09056714
EC_6.3.4.13	0.11313249	0.01622413	0.42513593	0.31711714	0.34061015	0.67879174	-0.2552718	0.04919957	0.09080267
EC_3.5.1.111	-2.7064506	0.13168584	2.20564538	0.72244768	0.34308082	3.61578139	-2.0771712	0.04924603	0.09080267
EC_2.5.1.47	0.28025467	0.3624482	1.00090867	0.63967365	0.83058066	1.75801823	-0.4514527	0.04936673	0.09092083
EC_3.2.1.37	0.07447325	0.87411438	0.56192779	0.85630323	1.76970928	2.3152594	-0.9299663	0.04948338	0.09103129
EC_3.2.1.78	-0.1992162	-0.0424008	-0.554622	-0.5474898	-1.174888	-4.2606217	1.05364077	0.05013848	0.09202822
EC_3.1.3.5	0.90282831	0.25240395	1.26342715	0.08669532	0.17301841	0.62511143	0.53093283	0.05013991	0.09202822
EC_1.17.7.4	0.06693147	-0.0982184	0.73433917	0.30238527	0.47462765	0.9401495	-0.3523858	0.05088385	0.09328705
EC_3.6.3.12	7.17302829	4.09457279	8.64916185	3.51843764	2.01341189	4.59575397	3.09713413	0.05099242	0.0933795
EC_3.1.1.3	4.13926174	1.46105482	4.2593847	0.65877604	0.21591084	1.95169079	2.52199942	0.05106957	0.09341427
EC_5.4.2.6	1.16285157	0.69327428	4.24539726	-1.3380071	1.37069286	2.83177614	1.27654108	0.05238075	0.09570362
EC_2.3.1.30	0.42787628	0.51027759	0.67268512	0.71469047	0.91947115	2.348574	-0.5093544	0.05285016	0.09645154
EC_3.2.1.170	2.93059934	1.74043027	4.75005811	3.55940636	5.2114282	10.405829	-2.2682233	0.05293476	0.09649628
EC_1.1.1.41	-2.1458859	2.70298699	2.46114437	2.32655757	2.02661198	3.10432044	-2.1941003	0.05342714	0.09728343
EC_2.5.1.6	0.10218709	0.07366412	0.37315899	0.277736	0.28411069	0.43400811	-0.1750537	0.05374719	0.09773846
EC_2.6.1.53	2.41418822	0.75761005	2.32030809	0.39861847	0.76674151	0.96430977	1.22230252	0.05379875	0.09773846
EC_2.7.1.6	0.18157643	0.41511374	0.82075166	0.5838976	0.69041422	1.89157279	-0.4479298	0.05412234	0.09819542
EC_1.7.2.2	5.20576706	4.67780667	10.6331767	2.6548245	4.08519539	5.51550076	2.18444059	0.05417256	0.09819542
EC_2.7.7.3	0.12125336	0.02125137	0.43787339	0.31770108	0.55878913	0.95737306	-0.3561243	0.05484451	0.09930134
EC_3.5.2.3	0.15282476	0.03192628	0.46792812	0.37071352	0.43329072	0.99506055	-0.3246068	0.0550584	0.09957634
EC_4.2.1.24	-0.0147391	0.57947875	0.99471971	0.77795137	0.88905804	1.90634815	-0.6431213	0.05535546	0.10000098
EC_1.1.1.205	0.06862359	-0.0633796	-0.1714382	0.12419784	0.09402938	0.22360923	-0.1349174	0.05555671	0.10025176
EC_4.1.1.18	5.96602566	3.97496039	7.87235159	0.61464805	3.10995064	5.46110458	3.42508587	0.05694896	0.10264875
EC_2.4.1.303	-2.4600163	-3.0272174	-0.4573433	1.96591495	-1.546741	-1.5688642	-2.7653424	0.05810861	0.10462156
EC_1.1.1.79	3.71029919	5.28314324	6.17244777	1.37290279	1.3300718	1.62867765	3.17672752	0.05823768	0.10464953
EC_1.1.1.369	-2.2750783	0.04088476	0.48782956	0.1842908	-0.4357333	1.58820645	-1.2814925	0.05825447	0.10464953
EC_3.2.1.23	0.26001202	0.32922445	0.90540824	0.60660653	0.87135451	1.49209097	-0.44608	0.05885298	0.10560658
EC_1.2.1.38	0.14321317	0.2283425	0.89681121	0.5494019	0.67283571	0.97898015	-0.3805617	0.0590547	0.10585028
EC_2.4.1.247	-2.4979788	-0.3133616	-0.345527	0.75982467	0.11530037	2.38612575	-2.2224446	0.0594827	0.10649857
EC_2.3.1.61	3.53805051	1.26825469	5.44589036	0.91333794	0.03617875	1.15865902	2.30998956	0.05973879	0.10683797
EC_6.3.2.10	0.34691903	0.28044868	0.67586871	0.6126787	1.03869067	1.68435485	-0.5294213	0.06012347	0.10740633
EC_3.6.1.9	2.07356336	1.78620602	6.63727341	-2.0590087	2.13211232	6.8367073	2.0475789	0.06033958	0.10767263
EC_4.1.1.96	-1.5663662	-1.7687392	-1.1810441	-0.2677137	-0.6521234	1.52394223	-1.4323641	0.06068751	0.1081733
EC_1.14.13.48	4.60635818	2.35867345	6.14369581	1.5632002	2.09555976	2.56133934	2.12980663	0.06077036	0.10820089
EC_3.5.4.2	1.12745999	0.57760855	0.68766512	1.28888238	2.04329826	3.56565307	-0.9564733	0.06085905	0.10823879
EC_2.4.2.22	0.08670693	0.67490617	1.8322329	0.49743674	1.62199975	2.76936889	-0.6555702	0.06096183	0.10830166
EC_1.4.1.2	0.55319512	0.93369645	0.32844118	-0.058984	-0.5924273	-0.0869653	0.89799478	0.06232783	0.11052952
EC_1.3.1.91	4.53158976	1.37366421	7.08043088	0.54692959	1.69593003	1.67828367	2.65318819	0.06235351	0.11052952
EC_1.3.1.76	0.31925039	0.21239702	0.93943337	0.90592582	1.07456577	1.78058658	-0.7186785	0.06310773	0.11174312
EC_3.1.1.1	2.09937235	1.3152848	6.12227374	-2.3670315	1.58629706	5.7587508	2.31827409	0.06359916	0.11248926

EC_3.2.1.91	0.77496965	0.96078806	-0.8781056	-0.8058238	-2.1574814	0	1.78137059	0.06376006	0.1126498
EC_2.1.1.80	2.70674395	-0.3288125	2.11928476	3.99976452	2.83009525	6.30403023	-2.359314	0.06412661	0.1131729
EC_1.1.1.317	-0.1011754	1.29870527	3.99272084	1.84873034	4.54340624	3.55426889	-2.0598654	0.06475455	0.11415566
EC_2.7.2.1	0.13270497	0.0485068	0.45025589	0.2038917	0.34549793	0.76796727	-0.1826033	0.0650321	0.11451925
EC_6.2.1.26	2.51327998	2.23174482	6.74005107	-2.375323	2.97562694	7.17750569	2.29776776	0.06612158	0.11631025
EC_6.3.1.5	1.0752207	0.92099615	2.59806583	-0.3048836	0.9118252	2.40154689	0.75772541	0.06632661	0.11654326
EC_2.7.2.4	0.28386669	0.14574554	0.58497953	0.41614542	0.58018361	0.86980418	-0.2576634	0.06673384	0.11713064
EC_4.99.1.1	2.80659312	2.51060838	7.46675595	-0.5523001	2.74574174	6.43756335	1.82580864	0.06699123	0.11745405
EC_1.1.1.60	3.24354977	1.64992398	3.74959231	1.40895914	1.48994124	2.5156013	1.16025925	0.06870645	0.12032995
EC_2.4.2.1	0.13641102	-0.0089965	0.30986549	0.29896685	0.19233147	0.60487113	-0.1980096	0.06989048	0.12223021
EC_2.7.9.2	2.49538055	2.05905662	3.61152922	0.49074681	0.02689046	2.66430644	1.85352534	0.06994369	0.12223021
EC_3.1.1.96	0.20591282	-0.0195224	0.18018348	0.2956059	0.58595708	0.93947782	-0.3524637	0.07044286	0.12296873
EC_2.7.1.130	0.42348178	-0.2056668	0.23161565	-0.0784277	-0.426777	-0.4914709	0.42401854	0.07080124	0.12346013
EC_3.2.1.n1	0.16237294	0.26767367	0	-0.372864	-2.6773887	-2.3622596	1.56955701	0.07099537	0.12366439
EC_2.3.3.14	-2.8381707	-2.4246511	-2.3479324	-1.0953406	-1.1129332	1.30125841	-1.8452619	0.07158606	0.12455819
EC_3.5.1.108	0.48879838	-0.1348035	0.04998077	0.02377715	-0.3386007	-0.2102723	0.33991872	0.07204367	0.12521875
EC_3.6.1.27	0.29505119	0.07480771	0.43005152	0.46072764	0.50696073	0.9143505	-0.301876	0.07218248	0.12532438
EC_3.4.21.62	-0.9150293	-0.1820153	-0.5377243	0.43015053	-0.1113647	0	-0.8052464	0.07329584	0.12712
EC_3.6.1.41	0.31419164	-3.1899373	-0.3226726	0.05539473	2.22001616	3.82097969	-2.2647475	0.07357971	0.12747467
EC_3.5.4.9	-0.0048317	-0.1095472	-0.2249873	-0.1114956	-0.2546007	-0.5797155	0.15100708	0.07427416	0.12853912
EC_3.4.21.72	4.12984828	1.30189197	4.21695979	0.72613635	1.65862018	1.55150391	1.99856975	0.07449693	0.12878587
EC_4.3.1.18	2.83903555	0.65143118	2.14061365	3.38830534	3.93951521	8.62327885	-2.2779942	0.07527109	0.12998426
EC_3.6.1.31	0.10546063	0.48654305	1.28910309	0.5401852	0.93162623	1.47265634	-0.4054879	0.07544019	0.13013636
EC_3.5.2.10	-1.6121383	0.06456977	0.24597092	1.18363894	0.61581301	2.42164487	-1.950549	0.07606467	0.13107282
EC_2.7.1.55	0.31023685	0.23708552	2.36350949	2.26923172	1.99297302	7.58284108	-2.3405165	0.07644261	0.13158289
EC_1.1.1.127	5.32775682	3.56872046	0.63122173	1.32108148	2.10234493	1.99975172	2.46267778	0.0773459	0.13299519
EC_2.7.7.6	0.1788141	0.05325858	0.00179287	0.01203364	0.00183349	-0.0143197	0.10741749	0.07825997	0.13442301
EC_2.4.1.187	0.88534272	0.77286798	3.1744678	-0.4891634	1.01829592	2.65681838	0.70777113	0.07847248	0.13464401
EC_2.1.1.198	0.16175451	-0.0067337	0.22054694	0.24465035	0.36603919	0.51535519	-0.2107242	0.07865614	0.13481511
EC_2.5.1.49	0.12954453	0.15289161	0.31537726	0.41434563	0.5195133	1.01557878	-0.3672014	0.07876395	0.13485597
EC_5.4.99.62	1.56495914	1.52409416	3.92996626	0.30942562	1.08975932	3.15029896	0.90195725	0.0788551	0.13486825
EC_4.2.1.36	6.20418661	2.86688891	4.16722891	1.78111509	3.54894378	2.83351656	2.27345498	0.07895224	0.13489074
EC_4.2.1.3	3.32950575	2.74029556	7.05021877	1.32323704	2.56207007	3.25413752	1.59380362	0.07958934	0.13583473
EC_2.3.1.47	2.04191623	0.65219	1.07060878	0.96931995	0.37732132	0.81651684	0.67548881	0.08046024	0.13717531
EC_3.6.1.57	-2.101287	-1.1388811	-0.4533063	-0.0118821	-0.9805564	1.03770602	-1.366754	0.08059371	0.13725716
EC_2.1.2.2	0.26439146	0.2538874	-0.3438798	0.51771022	0.31709273	0.95103863	-0.3227222	0.08072367	0.13733285
EC_6.1.1.2	0.18945411	0.04405688	0.66656662	0.32262674	0.57029922	1.11749097	-0.3117982	0.08145478	0.13843002
EC_4.2.2.23	-2.0373907	-0.515399	-0.0404132	0.41229761	0.61751864	0	-1.6789785	0.0821797	0.13946789
EC_1.17.98.1	-0.576605	0.85651239	1.02690715	0.0575994	3.62982803	4.14829282	-1.638631	0.08223916	0.13946789
EC_4.1.1.48	0.14908761	0.66332945	1.00957532	0.83263465	1.34469094	1.70977979	-0.6790367	0.08249072	0.13974695
EC_3.7.1.8	-1.2935812	1.58884173	0	1.41317766	1.91142431	1.40972094	-1.6901754	0.08260075	0.13978589
EC_4.3.99.3	0.59167602	0.06425775	0.44925861	0.04360672	-0.0899065	0.13721429	0.37528706	0.08292093	0.14018001
EC_2.7.1.2	1.35543653	1.10666863	1.4967169	0.32730656	0.73982671	1.47026225	0.67195906	0.0831287	0.14038348
EC_2.7.1.113	-0.8980652	-2.6603863	2.2644016	-0.9207492	0.70392595	2.56704692	-1.2047097	0.0836078	0.14104425
EC_1.8.7.1	-0.398919	2.11333619	0	2.07242092	1.74677048	1.1491053	-1.3045629	0.08369656	0.14104583
EC_2.1.1.181	4.64538965	2.66299673	7.48469045	1.05344579	1.82899703	6.05105735	2.32712634	0.08384639	0.14115021
EC_2.8.3.1	-1.2125189	2.61031583	2.57203671	2.31968352	1.9901528	2.67615323	-1.6530586	0.08478374	0.14257872
EC_2.1.1.190	0.43487107	0.85455303	1.11323323	1.02839601	1.48807802	2.42149818	-0.6954198	0.0850346	0.14285102
EC_3.4.25.1	0.8801122	-0.8867794	-5.0802353	1.70624851	0.48469989	2.82992375	-1.9295452	0.0859877	0.14430119
EC_1.1.1.3	0.75172967	0.5277217	0.51147899	0.95112371	1.16084735	1.91923375	-0.5040984	0.08742416	0.14655867

EC_3.6.1.1	0.50732404	0.34719045	0.47580307	0.49648487	1.29287238	2.66778771	-0.5960438	0.08775613	0.14696179
EC_1.1.1.267	0.10701502	-0.0612894	0.43797314	0.29069075	0.37733078	0.74155786	-0.2903479	0.08857214	0.14817381
EC_1.1.1.38	1.27799654	0.71971374	1.6666552	1.70580061	2.53445685	4.6615482	-1.2378165	0.0886799	0.14819971
EC_1.2.7.7	0.62759095	1.56788647	5.51423608	-0.313682	-0.6431009	2.75708504	1.57798533	0.08881736	0.14821007
EC_5.3.1.23	-0.7938233	0.875805	0.29661923	0.41862325	0.71536951	2.24287267	-0.8243017	0.08887067	0.14821007
EC_3.1.3.23	1.4747112	0.69487748	4.06587543	-1.4439968	1.67677643	3.58741353	1.27599143	0.0911265	0.15181449
EC_3.2.1.135	1.27393825	1.0078489	3.19162981	-0.2012909	1.29792532	2.41554612	0.78075617	0.09171815	0.15254548
EC_2.5.1.9	0.14565677	-0.0273082	0.22152419	0.45355732	0.57181906	1.49275409	-0.5351258	0.09175525	0.15254548
EC_1.1.1.313	-2.3559302	-0.5969162	2.6018986	-0.0160445	1.49910364	3.10741656	-2.0162085	0.09191279	0.15264936
EC_5.1.3.9	0.52231469	0.25406367	1.26354629	0.62329335	1.26038874	2.52608558	-0.5639466	0.09270928	0.15381313
EC_2.6.1.92	-1.0301532	0.21054832	0.18516097	0.05378595	0.24036205	2.02457691	-0.8155362	0.09502429	0.15749124
EC_3.5.1.16	3.12158783	2.15428307	5.10261583	1.56414136	1.39333266	-0.1153949	1.73422498	0.09537218	0.15790486
EC_2.8.1.6	0.06784796	-0.1361768	0.65620964	-0.0136322	-0.2384347	-0.5345529	0.22345542	0.0959699	0.15873086
EC_2.7.11.32	1.41135284	0.48583977	3.46606771	-1.1765078	1.22600195	3.21395986	1.14748819	0.09684753	0.15985318
EC_2.7.4.27	1.41135284	0.48583977	3.46606771	-1.1765078	1.22600195	3.21395986	1.14748819	0.09684753	0.15985318
EC_4.6.1.1	3.81586113	1.49547656	4.35654598	1.19543293	0.4352994	1.91875683	2.01906786	0.09698561	0.15991673
EC_2.7.9.3	0.94975837	1.17553596	1.43798452	1.65228438	1.43120967	2.92096624	-0.6568469	0.09721917	0.16013742
EC_4.2.3.4	0.1137468	-0.024975	1.07242499	0.39232416	0.66130859	1.2459177	-0.4105157	0.09751311	0.16037604
EC_3.1.11.3	3.51524362	2.20662925	4.62810109	0.78343506	1.79006397	1.70309548	1.95384339	0.09756376	0.16037604
EC_2.7.1.12	0.59873134	0.63211293	5.63703616	-3.6062869	1.41484864	5.50445259	1.9914006	0.09833053	0.1614712
EC_1.1.1.18	0.38501728	0.6305176	1.24880306	0.91239004	1.08372013	2.58807261	-0.6107984	0.09900016	0.16240476
EC_3.2.1.184	-0.4670143	1.52187106	0	0.98399159	2.18528236	5.26928033	-1.6460091	0.09963691	0.16328253
EC_1.2.1.76	1.70703462	3.10544543	6.52478715	3.08755726	6.55038662	8.70911144	-2.1684652	0.09980532	0.16339179
EC_7.3.2.2	1.10002278	0.61464905	3.92498775	-1.00845	1.39601203	2.88414307	0.98315266	0.10074724	0.16476586
EC_3.2.1.54	2.14912346	1.49527419	2.61365804	2.34991918	3.82035912	7.95924747	-1.5943036	0.10108177	0.16514478
EC_1.2.1.16	2.18851064	1.20954938	4.34012982	-2.5585856	2.35971276	6.64110204	1.84313274	0.10171051	0.16598048
EC_2.1.1.201	3.96652915	2.5728522	5.02460107	0.82232459	2.35532484	1.75769177	2.15473662	0.10179998	0.16598048
EC_1.20.4.1	5.69976741	3.95524475	7.38720221	3.25849357	2.96284779	1.86406422	2.31026671	0.10203936	0.16620205
EC_1.8.98.1	-0.6533899	-3.1174016	-2.6337675	0.99275363	-0.3260515	-0.9834707	-2.0696452	0.10293932	0.16744778
EC_3.5.5.1	-0.6614799	0.09716236	0	0.73074039	0.63205399	0	-0.9141035	0.10301271	0.16744778
EC_4.1.2.21	3.76776592	3.81994094	7.51338206	1.48127921	1.84946213	6.27790971	2.01336746	0.10432835	0.1694149
EC_3.2.1.8	-0.0071286	0.0585903	0.93900551	0.28832282	0.89558264	1.02910117	-0.4526177	0.10459546	0.16967708
EC_1.17.1.2	0.10156856	1.52916533	3.89087793	1.35852759	4.2756914	4.21572415	-1.6186395	0.10660285	0.17275901
EC_2.4.1.18	0.08871309	0.02699917	0.12218241	0.21526626	0.16211322	0.32853183	-0.1417569	0.10705185	0.17327251
EC_6.4.1.7	-1.4537958	-3.0650196	-2.299352	-0.7961152	-1.2857117	0.72584958	-1.3713614	0.10713575	0.17327251
EC_3.2.1.96	3.12204462	1.54130947	2.73845497	0.99274586	0.20431744	2.18211451	1.62470641	0.10724338	0.17327251
EC_2.4.1.129	1.77409264	0.52059149	1.66967947	0.61960238	0.41528815	1.41084	0.66078055	0.10736445	0.17329377
EC_2.1.1.223	1.47766162	0.73357775	-1.0754333	0.76744236	-0.000302	-0.5123679	0.55356438	0.10776606	0.173509
EC_5.1.1.3	0.09650102	-0.006761	0.45613101	0.20927285	0.33572338	0.55581082	-0.1922436	0.10777346	0.173509
EC_6.3.4.21	-0.2180206	0.06550611	0.7948467	0.02359528	0.34181743	0.59615953	-0.1965883	0.10782191	0.173509
EC_1.14.13.81	-0.9113156	-0.5417059	1.04040104	0.37710059	0.22437783	2.65243778	-1.167037	0.10892259	0.17510479
EC_2.7.1.71	0.16500125	0.07038477	0.60944304	0.37450219	0.48098274	0.57215138	-0.249413	0.11539213	0.18529823
EC_6.3.2.7	5.67702727	3.65268562	5.19468006	3.59873278	1.71118821	1.27473463	2.22629592	0.1154941	0.18529823
EC_1.1.1.306	0.46165639	2.93641165	2.89377783	4.09608214	2.94765061	2.38191397	-1.8580734	0.1164378	0.18643266
EC_3.4.17.19	1.08598842	1.0039587	1.42809924	2.01918521	2.14351486	3.74118681	-1.1872456	0.11655796	0.18643266
EC_1.5.1.39	0.48854005	0.98368413	0.08846721	0.28213853	-1.0759878	-0.1154199	0.83028504	0.1165622	0.18643266
EC_4.1.2.40	2.13256052	1.44084812	4.72173292	-0.559498	2.12407066	4.13072918	1.26847776	0.11666552	0.18643266
EC_1.1.1.69	-0.1628126	-0.2786466	-0.7656903	0.22060509	-0.2140666	0.93420727	-0.4495317	0.11693663	0.18668014
EC_6.3.1.2	0.39915993	0.77599597	1.02863579	0.66211675	1.23042086	2.0814023	-0.4250752	0.11784805	0.18794832
EC_1.2.7.1	0.45291015	0.52874236	0.72358278	0.89480071	0.99576523	2.38523313	-0.6107063	0.1201855	0.19148603
EC_4.1.1.21	4.48174544	3.55953353	6.31735317	1.55675143	2.36480177	4.53061736	2.16780341	0.12034957	0.19155739

EC_1.14.12.7	3.73379729	1.30471781	4.27086629	0.74030353	1.76731944	1.55532603	1.75514536	0.12140174	0.19304078
EC_2.7.6.1	0.18796903	0.0732118	0.4449257	-0.1890939	0.18639231	0.27987281	0.18319793	0.12176684	0.19342982
EC_4.1.1.41	1.10792321	0.10335631	-1.4860151	0.22312477	-0.6310983	0.08160077	0.50973455	0.12231571	0.1939806
EC_6.1.1.17	0.11383485	-0.0507643	0.32255769	0.15103578	0.2889793	0.64971093	-0.1806439	0.12235513	0.1939806
EC_1.8.4.14	4.80101018	2.32113518	3.34990861	0.9972264	1.34317759	4.48052727	2.17664217	0.12449915	0.19703327
EC_6.1.1.20	0.06164661	-0.0315228	0.1273539	-0.0420026	-0.0800999	-0.1238459	0.10194304	0.12458667	0.19703327
EC_4.3.2.1	0.59966384	0.48710453	1.02778549	0.81852291	1.05041788	1.78382164	-0.4090686	0.12464869	0.19703327
EC_2.7.8.7	1.43639742	1.0296057	3.78709495	-0.3666173	1.40679627	2.59782576	0.97621604	0.1253274	0.19791131
EC_3.6.3.5	6.34651685	1.22734418	2.89221599	1.02130223	2.36816563	4.13049085	2.23864581	0.12623423	0.19914752
EC_6.2.1.5	4.51640058	3.47564963	6.06071031	2.1131963	2.7589792	3.22696701	1.86582523	0.12707587	0.20027855
EC_1.1.1.133	0.05822439	0.14414469	0.34858884	0.19461037	0.47975629	0.72181537	-0.2331027	0.12724629	0.20035054
EC_3.6.1.55	0.79689329	0.99091094	2.0845291	2.30185631	2.29280619	5.46663234	-1.6910967	0.13001364	0.20450725
EC_5.3.1.15	-1.0792621	-0.1565321	0.45911069	0.43676865	0.83348783	0	-1.0733669	0.13074713	0.20545978
EC_2.7.1.69	1.39805271	0.64436285	3.80994527	-0.5069251	1.36225426	2.85254809	0.87952603	0.1327347	0.2082855
EC_3.5.4.33	-0.0206766	-0.0237524	0.07053633	0.08149775	0.18490714	0.2114925	-0.1430646	0.1328047	0.2082855
EC_1.21.4.1	-2.7413282	0.23870211	1.37333914	0.53769138	0.58938421	-1.6515078	-1.4486043	0.13449712	0.21066513
EC_3.1.3.82	0.29161888	1.41860451	4.63061199	2.9889297	2.67487929	7.71503884	-2.2840396	0.13458432	0.21066513
EC_4.2.1.113	2.003896	1.23519528	6.29932065	-1.7128524	2.48193204	5.82887867	1.60500423	0.13515973	0.21135981
EC_4.1.3.30	5.18796192	2.35807317	4.4330785	1.38762254	2.13972113	3.04117593	2.24169049	0.1358802	0.21227976
EC_1.17.4.2	0.4544715	0.73305531	1.11342354	0.81334988	1.29049965	2.09055864	-0.503636	0.13649071	0.21302632
EC_1.4.1.4	0.09052222	-0.5338085	-1.293634	-0.4021273	-1.4325783	-0.843914	0.49776411	0.13739832	0.21422475
EC_1.9.6.1	2.75690853	1.30167686	6.39881463	0.64800639	2.03183976	3.47419123	1.21490662	0.13752535	0.21422475
EC_2.6.1.19	2.06370378	0.75822251	3.90913278	-1.8423604	1.85648912	5.63838854	1.47452872	0.13786975	0.21455312
EC_7.2.4.1	-1.5732466	-0.698962	-2.3813031	0.08772448	0	0	-1.4100551	0.13852138	0.2153585
EC_3.3.1.1	0.02370719	0.33198902	-1.0841296	0.36845558	0.14815724	0.73836221	-0.3476856	0.14305943	0.22219869
EC_3.6.3.30	2.99722668	1.59776509	4.32470952	1.48845679	0.75666701	2.39437091	1.29676135	0.14460188	0.22437741
EC_2.2.1.7	-0.0197371	0.00547421	0.30133283	0.13323767	0.2085794	0.43366497	-0.1688532	0.14534268	0.22530921
EC_1.21.4.4	-0.6663115	-0.8660312	-0.4598404	-0.216193	1.22376391	4.95239521	-1.6465244	0.14548492	0.22531224
EC_5.3.1.22	6.18234262	4.19488924	3.01864952	3.26811816	1.27296086	3.24375132	2.47991227	0.1465877	0.22680139
EC_2.1.2.11	0.40305815	-0.0403668	0.39177698	0.14385641	-0.1011923	-0.0125969	0.2016567	0.14676771	0.22685138
EC_3.4.16.4	2.79492913	1.51778651	3.66434584	1.18194012	1.60351223	3.05700012	0.8826952	0.14690252	0.22685138
EC_2.7.1.21	0.15481584	-0.2364011	-0.2941803	-0.1202657	-0.3345553	-0.3113879	0.17684651	0.14787999	0.22814146
EC_2.5.1.72	0.1144439	-0.2127093	-0.4327803	0.36459706	-0.0692082	0.20496848	-0.2698939	0.14881254	0.22935982
EC_4.3.1.3	-0.1818767	-0.380006	-0.2750716	-0.2527272	-0.8381249	-1.3438621	0.3245046	0.14994596	0.23075152
EC_2.1.2.9	0.27319156	0.06418726	0.302027	0.05596242	0.03667581	-0.0033465	0.16084752	0.15007763	0.23075152
EC_2.3.1.266	0.95037948	0.41429762	1.20419585	1.06155855	1.17440963	2.53079117	-0.4999138	0.15014654	0.23075152
EC_2.1.1.163	0.21709094	-0.2126797	1.3554337	-0.2975983	-0.0531504	0.24473264	0.35060746	0.15039119	0.23090655
EC_1.1.1.35	2.46601609	1.4483602	4.23901803	-0.3418189	2.21614184	4.6456167	1.17092245	0.15215939	0.23287959
EC_1.1.1.28	0.7356464	0.38573144	2.3773828	-0.5664238	0.73362122	2.32964542	0.57271818	0.15231708	0.23287959
EC_2.6.1.42	0.67935773	0.68096583	0.53038527	0.73605131	1.16352779	2.04755649	-0.3867973	0.15232248	0.23287959
EC_3.4.11.6	0.75058074	1.76017696	4.50129448	2.46104694	3.1724556	7.53267092	-1.7925015	0.1523377	0.23287959
EC_1.17.1.4	2.88549909	1.32138183	2.5827811	0.98943028	1.20625797	3.01169079	0.96826631	0.15243689	0.23287959
EC_3.1.3.1	1.07622827	0.72613724	1.71613735	0.05631252	0.64167921	1.65877439	0.57194677	0.15254628	0.23287959
EC_1.21.4.3	-0.6849045	-0.8050348	-0.4598404	-0.216193	1.15661239	4.95239521	-1.6126291	0.1531134	0.23352339
EC_1.2.1.68	-1.7394343	-0.7528995	-2.8569949	-0.2565866	0.14023404	3.00053496	-1.8282823	0.1547673	0.23549877
EC_1.1.1.122	0.85022707	1.36669202	-0.4540944	0.507254	-0.7757667	0.15074769	0.83355881	0.15483391	0.23549877
EC_3.5.4.3	3.3139568	1.91530814	3.86530725	1.16107451	1.59331176	2.6400416	1.38909019	0.15484851	0.23549877
EC_5.1.1.4	-3.1665649	2.42580318	7.29318958	1.77466126	1.54535198	4.17574255	-1.9185451	0.15665643	0.23802292
EC_2.1.1.74	0.32483669	0.41924034	1.14555053	0.46645634	0.85526244	2.66448888	-0.4237553	0.15773268	0.23943165
EC_4.2.1.151	0.25778662	-0.3951189	-0.4197234	0.59980532	0.64389432	0	-0.5919996	0.15833895	0.24012498
EC_3.2.1.14	2.09617614	1.15506346	-0.4695932	0.43337208	-0.3242421	1.21280266	1.16103739	0.15888945	0.2407325
EC_2.4.1.250	1.17012691	1.31301334	-0.949736	0.14336223	0.13360246	0.18280719	0.81556933	0.1593685	0.24123074
EC_2.3.1.51	0.11118649	0.34311456	0.0628634	0.50955293	0.26362572	0.7818232	-0.2772599	0.1611455	0.24369084
EC_2.7.7.9	1.69228025	1.14480475	3.43018099	0.31636519	1.35175353	2.77343848	0.73280933	0.16233039	0.24502123
EC_2.4.2.43	4.55430747	0.45905925	2.11646025	0.806169	0.56564242	2.08038904	1.91032281	0.16253496	0.24502123

EC_2.7.8.26	0.13759141	-0.1274121	1.87212607	0.44201381	0.59174595	1.86328594	-0.4214484	0.16259727	0.24502123
EC_2.7.1.15	1.15821234	1.40575761	3.3782634	0.30662247	1.38875707	2.41719414	0.57935547	0.16263551	0.24502123
EC_4.2.1.59	0.27232135	-0.0755983	0.17310819	0.30567549	0.19164923	0.64164283	-0.1754825	0.16380375	0.24654997
EC_3.1.26.4	0.15882298	0.02876838	-0.0289806	0.22525485	0.29796553	0.40129915	-0.182478	0.16440689	0.24716329
EC_2.1.1.264	3.18090316	0.60460225	4.69313141	3.44765765	4.10024321	7.36919739	-1.722944	0.16466285	0.24716329
EC_3.2.1.122	0.76831186	1.16899797	2.08949748	1.85846682	3.47996238	4.91584346	-1.7195215	0.16467293	0.24716329
EC_1.1.1.290	0.66809728	-0.1507791	1.14589539	-0.0079362	0.01397531	0.55155994	0.36388433	0.16723003	0.25076697
EC_3.2.1.n2	0.14213967	0.24207187	0	0.05612699	-1.8720675	-2.1893612	1.03114713	0.16774791	0.2513089
EC_2.1.1.193	0.29895436	0.00048664	0.26511343	0.38755353	0.46426178	0.81752177	-0.2800861	0.17055355	0.25487662
EC_3.6.1.8	2.39372668	-0.1043724	-0.5780182	0.65794209	-0.5534616	0.79246322	0.86627006	0.17055649	0.25487662
EC_3.6.3.20	1.60829889	1.230946	3.51366334	0.25858226	1.3727285	2.69939557	0.76370476	0.17060546	0.25487662
EC_3.5.1.87	0.02315541	-0.0051674	1.97412087	0.50496465	1.43707095	4.26207109	-1.0517897	0.17101972	0.25525806
EC_2.6.1.57	5.13067195	1.42332679	7.30453935	1.47530779	1.97279455	3.14655457	2.22605187	0.17225769	0.25686708
EC_5.4.3.8	0.24854099	0.7106329	1.10939304	0.8504107	0.94205639	1.74799207	-0.4806186	0.17455519	0.26005161
EC_3.4.13.21	1.89490366	-0.7575115	-0.6381682	-0.1167441	-1.6005519	0.66664784	1.14534305	0.17537855	0.2610361
EC_3.1.21.7	-0.4863669	-4.7892524	-0.3123402	-0.5395755	-0.7539945	1.6784485	-1.6705369	0.176719	0.2627877
EC_4.1.3.6	2.29396753	0.36139033	1.02427714	0.92931147	0.82302147	0.65183701	0.59581004	0.17737158	0.26334834
EC_3.6.5.n1	0.06142034	0.01175402	0.03576319	0.13487614	0.15225999	0.3694411	-0.1306989	0.17742398	0.26334834
EC_2.3.1.274	0.62119511	-0.7231606	3.6785805	-2.245615	0.72615946	2.38173731	1.18320651	0.17854705	0.26477061
EC_2.7.1.121	1.01204863	1.48320336	2.17042641	2.40809206	3.23910336	4.41035093	-1.6226337	0.18001957	0.26670797
EC_2.7.7.15	-0.8539862	-0.7233181	0	0.69108196	-0.4299373	1.75686405	-1.1665093	0.1814192	0.26853386
EC_3.1.2.29	-1.3890593	-1.4991068	-0.805795	-0.1434863	-0.4689769	0.04745446	-1.1314123	0.18163321	0.26860307
EC_1.8.1.9	0.28007467	-0.0349282	0.30996969	0.31598068	0.41545133	0.78003507	-0.2373782	0.18253541	0.26968892
EC_2.7.1.50	0.57066	0.75187249	1.52711851	0.69337064	1.60143259	2.42900351	-0.4669945	0.18294951	0.2700523
EC_2.5.1.78	0.10529029	-0.241577	-0.2014642	-0.0195691	-0.3721601	-0.5251064	0.14640933	0.18411265	0.27148892
EC_1.8.4.11	0.92039124	0.28365099	2.70939861	-0.0619281	0.8380726	1.34026204	0.49679577	0.18426085	0.27148892
EC_5.2.1.8	0.49551526	0.17009778	0.67916549	0.17926514	0.24486955	0.2191381	0.19674864	0.18494268	0.27213986
EC_3.1.4.16	2.26804629	0.0532612	1.57083133	1.23447597	-1.0012139	0.59715256	0.98041868	0.18504155	0.27213986
EC_3.5.4.16	0.25971985	0.04236072	0.31346245	0.36272744	0.2637231	0.85443646	-0.205524	0.18532292	0.27230431
EC_2.3.3.10	1.01945608	-0.4105142	4.67377026	-4.090288	2.01178753	6.21876573	1.69635868	0.18573829	0.27266517
EC_2.1.2.3	0.57022124	0.22140104	0.11119386	0.24219056	0.14258154	0.22333953	0.18335443	0.18842097	0.27635076
EC_2.1.1.192	0.0670581	-0.0730455	0.0375771	0.17189892	0.16111716	0.26191271	-0.1663654	0.18921368	0.27726019
EC_1.1.1.130	0.28819978	0.57095953	2.14147024	1.33911348	1.5430502	3.34287823	-1.053644	0.18995575	0.27809384
EC_5.4.2.11	0.60273513	-0.0341577	1.56605408	-0.2823223	0.46265681	0.86687555	0.38233544	0.19279982	0.28200046
EC_3.4.22.40	-0.3452514	-1.4486844	1.9853445	-0.3786367	0.58265834	1.98045168	-0.6968316	0.19386741	0.28330397
EC_5.1.1.1	0.26346134	0.00962005	0.82493439	0.34239958	0.57492559	1.12617108	-0.2792115	0.19555265	0.28523941
EC_2.7.3.9	0.55870426	0.64913846	1.61782569	0.75587264	1.41545826	2.50162952	-0.4808777	0.19563843	0.28523941
EC_6.2.1.22	1.89966627	-0.6748341	1.22482621	1.56581417	2.12624569	4.0090452	-1.1653923	0.19577597	0.28523941
EC_2.7.7.60	0.18231925	-0.0122428	1.27030015	0.32941765	0.76181368	1.06527534	-0.3210866	0.19590228	0.28523941
EC_3.4.21.102	1.11208646	0.63653207	1.10484378	0.13463094	0.72067382	1.31323395	0.4611735	0.19614704	0.2853371
EC_2.7.1.170	3.37920873	2.69225398	7.30237988	1.5331522	1.98229347	3.10915344	1.72933357	0.19678216	0.28600195
EC_4.1.2.5	1.89377778	0.07077592	0.63045756	1.04218997	-0.3803466	-0.7456979	0.75229741	0.19720235	0.2863535
EC_6.1.1.19	0.18756353	0.03011369	-0.1247901	0.0490586	-0.0096124	-0.1287306	0.08703993	0.19845633	0.28791407
EC_2.7.1.25	0.73934698	0.53776342	0.65748333	1.02635285	0.74400316	1.67261083	-0.3607346	0.19941764	0.28904759
EC_5.1.3.32	0.88160857	0.12167166	0.1267662	0.62726418	0.97630637	2.08133796	-0.4127637	0.19967163	0.28915477
EC_2.3.1.179	0.03238549	-0.0928854	-0.3519316	0.0191628	-0.3590116	-0.4666093	0.10954825	0.20112274	0.29099381
EC_3.5.1.49	-1.8343451	0.67520489	0	0.03488211	0.58719718	0	-0.932037	0.20329011	0.29386492
EC_2.7.14.1	-0.310472	0.26740845	-1.1949419	0.01954321	0.38125977	2.32246481	-0.6547959	0.20357059	0.29400573
EC_3.6.3.41	3.39628008	2.0153136	6.06242908	1.24608368	1.91751358	4.54494027	1.33442722	0.20382567	0.29410964
EC_3.6.1.23	0.15055268	0.09469821	0.66245221	0.03024089	0.18347014	0.0697131	0.10784263	0.2048156	0.29527276

EC_2.7.1.23	0.20469546	0.07401751	0.59762006	0.27596413	0.54598429	0.97397689	-0.2496072	0.20506596	0.29536855
EC_2.7.1.83	3.07261573	1.25251782	2.0178822	0.52850503	0.58228301	1.20722906	1.66338378	0.20610107	0.29659347
EC_1.11.1.1	0.23819601	0.33386085	-0.6840396	0.35275348	0.32472853	0.83254517	-0.2475326	0.20744021	0.29825334
EC_6.5.1.2	0.23580945	0.0241898	0.63930665	0.28427354	0.35870557	0.89835212	-0.1790199	0.20765933	0.29830132
EC_2.2.1.2	1.30682945	0.19588754	1.49208887	1.20268447	1.55845905	2.01336482	-0.4897713	0.20818509	0.29878933
EC_1.16.3.1	0.17175889	-0.1722468	-0.4086048	0.14254231	0.52553156	1.0979067	-0.414817	0.20947362	0.30037021
EC_3.2.1.177	1.39632302	0.63628276	0.48400524	1.37670498	1.98766371	3.89240419	-0.8921377	0.21045279	0.30150507
EC_1.1.1.274	1.52194602	0.72605653	3.47464278	-1.9990107	1.98055106	5.05841361	1.24661811	0.21105007	0.30195143
EC_UNGROUPED	0.12819791	0.11534575	0.46736143	0.02813254	0.10467579	0.1339278	0.09809052	0.21114038	0.30195143
EC_4.1.3.3	1.41732928	0.85645163	1.68162991	0.58691773	0.73022507	1.86378698	0.44945652	0.21227003	0.30329685
EC_2.7.1.156	3.62990932	1.47249035	3.69210832	1.58558517	1.09791291	1.77037222	1.42342855	0.21303868	0.30404752
EC_2.7.7.62	3.62901201	1.47249035	3.69210832	1.58558517	1.09791291	1.77037222	1.42295905	0.21317404	0.30404752
EC_3.2.1.179	2.3974743	2.1542956	5.10253116	3.50856997	3.8655362	6.24041213	-1.3415117	0.21360621	0.30439359
EC_2.8.3.10	0.9764693	-0.1586436	-0.3855936	0.8237105	0.72879131	0.79295815	-0.3810791	0.21670453	0.308535
EC_4.2.1.30	-2.3807321	0.29505139	3.23254485	0.09013187	0.47743166	1.91876705	-1.2022571	0.21731501	0.30913012
EC_2.1.1.144	4.41041682	2.25503369	4.76241072	1.26955562	1.31937752	5.60020922	1.83546813	0.2185262	0.31057795
EC_4.3.1.12	-1.9807746	-0.2330201	-0.3420225	-0.0111735	-0.0948056	-0.0987846	-1.1107611	0.21901445	0.31099665
EC_3.1.21.2	0.153794	-0.1389561	0.11173862	0.31908854	0.061376	0.47363215	-0.2099347	0.22004374	0.3121822
EC_2.1.4.1	0.67616357	0.35869761	3.111561	0.91178443	2.1282995	3.85677627	-0.8352387	0.22199224	0.31466862
EC_3.1.1.11	-0.1771601	-0.3454893	-0.6143903	-0.3590669	-0.9895214	-0.8931849	0.34556722	0.22396643	0.31718703
EC_3.1.11.6	0.29278185	0.1375264	0.95570232	0.37841406	0.70944607	1.05018443	-0.2561426	0.2260463	0.31985054
EC_6.3.2.5	0.36917075	0.13755817	0.54079666	0.17521213	0.0647834	0.28852558	0.1542846	0.22641683	0.3200928
EC_1.3.99.5	0.87475023	1.10477593	0.10574337	0.3337951	0.00103531	0.66426197	0.59388468	0.22721784	0.3209427
EC_1.1.1.291	4.19675954	3.35992772	3.42606141	2.27477829	2.27222814	2.40052808	1.51543451	0.22831113	0.32220358
EC_UNMAPPED	-0.2787733	-0.1558512	-0.557905	-0.1058678	-0.1567258	-0.1325295	-0.1434011	0.22864694	0.3223217
EC_5.1.3.1	0.45383953	0.15046471	0.26190252	0.22868491	0.09816319	0.19430244	0.1414876	0.22879623	0.3223217
EC_5.3.1.1	0.14515044	0.10354747	0.22507859	0.04248087	0.05160257	0.16476133	0.07867977	0.23094594	0.32488197
EC_3.6.3.40	0.66310207	1.51788826	2.91232273	1.74380762	2.49919537	5.2975043	-1.2166727	0.23101819	0.32488197
EC_3.2.1.141	-0.0776027	1.37109902	5.18157454	1.44327957	2.59054608	5.20808579	-1.2337853	0.2317359	0.32553952
EC_4.3.1.1	0.88029708	-0.0193118	0.2482855	0.72603004	0.74961307	0.96602865	-0.2810907	0.23189117	0.32553952
EC_5.4.2.9	-1.1671796	-0.8520095	-1.5169534	-0.6594166	-0.8665807	0.92207312	-0.578776	0.23276897	0.32648643
EC_1.6.5.3	1.08061835	1.10714492	0.3500488	0.51408335	0.25490533	0.73042498	0.54295768	0.23302633	0.32656221
EC_2.4.1.52	-1.0559937	0.8179247	4.58267774	-2.9515038	0.09799832	4.94665259	1.21314264	0.23398634	0.32762167
EC_3.4.14.5	0.56076116	-0.4173866	-0.8039486	0.12987298	-1.7509997	-3.1682096	0.96704546	0.23513038	0.32893676
EC_3.5.4.10	0.41575352	-0.047329	-0.3019522	0.03679498	-0.0396476	-0.1327005	0.17429377	0.23570414	0.32945244
EC_2.3.1.19	-0.5106995	0.15374009	-0.5825286	0.22451207	0.10026665	1.27135152	-0.6054175	0.23597743	0.32954761
EC_3.5.4.4	1.43232119	-0.4963026	4.45538917	-2.3194891	1.84808038	3.94945583	1.24935716	0.23781776	0.33172676
EC_2.7.1.107	1.32832494	0.85439991	1.83932598	0.35333046	0.93236766	1.8145848	0.48798451	0.23795095	0.33172676
EC_2.3.3.1	0.60762222	0.89363293	2.26201016	1.08869443	1.41789342	3.69888048	-0.6277446	0.23953617	0.33364709
EC_2.1.1.182	0.07402188	0.05711249	0.41571959	0.18419426	0.29973748	0.49643012	-0.1531744	0.24009997	0.3341426
EC_6.1.1.16	0.14855833	0.07486683	0.14685947	0.18392903	0.24578206	0.44736037	-0.1165595	0.24205848	0.33657655
EC_3.4.19.3	-0.0767774	0.26243407	2.88135906	0.11540692	1.59285895	3.08734216	-0.5842359	0.2427672	0.33723406
EC_2.5.1.29	-2.245357	-2.2764521	2.6582494	-3.0172185	1.70111156	6.75428624	-1.4685954	0.24295131	0.33723406
EC_5.3.3.10	1.89545233	-0.1557655	0.62063335	0.20441125	0.42526222	1.39512484	0.57945169	0.24571052	0.34076952
EC_6.3.2.31	-1.2072594	0.83949396	0	0.46420802	0.80042353	1.28496654	-1.0165762	0.24604413	0.34093776
EC_2.7.7.56	2.10421279	1.47042363	0.5798913	0.858933	1.42336877	1.63295881	0.547227	0.24856482	0.34413371
EC_1.3.5.1	0.91727034	0.10873536	-1.1911141	0.32290021	-0.5529626	-0.0172358	0.38143378	0.24916776	0.34467134
EC_2.3.1.9	0.67719359	0.46959733	1.78437499	0.68497324	1.83039814	4.45497256	-0.8163188	0.24979812	0.34524594
EC_2.6.1.87	2.00908492	1.59376453	2.75014988	1.33040512	0.99329148	2.39838071	0.5926805	0.25182276	0.3476836
EC_3.1.3.45	1.57613052	0.33611627	-0.3202679	0.53702072	-0.0829703	0.71981658	0.5467475	0.25215925	0.3476836
EC_2.6.1.39	2.49246636	3.65511257	0	2.17959447	-0.7849779	2.28709753	1.37714365	0.25241589	0.3476836
EC_2.3.1.50	0.25338977	1.1316826	-1.201622	-0.5251338	-0.4713205	0	0.82466458	0.25242782	0.3476836

EC_4.1.1.65	0.2137941	-0.3443239	0.49705069	-0.0783049	-0.2846726	-0.2546127	0.21995452	0.25315286	0.34838345
EC_2.4.1.182	0.4105528	-0.4176879	0.79354262	-0.0452364	-0.3204196	0.01341778	0.29189725	0.25504223	0.35068307
EC_1.18.1.1	2.99651351	1.26228889	3.42415292	1.83396323	0.68222936	0.96846084	1.09202421	0.25674662	0.35272461
EC_3.1.3.89	2.74879894	1.64123205	3.94736961	3.27692679	3.25862833	9.39882257	-1.5715376	0.25741128	0.35333548
EC_5.3.1.26	2.45402509	2.2126781	6.60048448	-0.7884527	2.75581064	8.23234698	1.31831555	0.25855179	0.35459793
EC_2.7.8.6	4.85615074	0.94425911	5.94648804	2.08632389	0.80768605	3.59109248	1.73951049	0.25972588	0.35590424
EC_2.3.1.39	0.22919413	0.01790521	0.38887745	0.16428521	-0.0631848	0.08040798	0.09524523	0.26144812	0.3579588
EC_3.5.1.54	-1.38546	0.55164863	0.06400452	0.45436476	-0.0010057	0	-0.7670565	0.26751677	0.36595565
EC_1.97.1.4	0.76251257	0.67034057	1.02715319	0.76576177	1.37352724	2.27706941	-0.4012318	0.27007377	0.36913913
EC_3.1.22.4	0.29901458	0.16892913	0.22373676	0.29615864	0.33695147	0.75485935	-0.1260659	0.27150154	0.37077506
EC_3.2.2.15	-0.6882576	0.40838015	-0.3249813	0.01733402	0.16608403	1.32910008	-0.4936921	0.27330886	0.37292611
EC_3.5.3.3	-0.3663819	3.25218273	0.5082037	0.42637439	-0.4913438	1.14308917	0.80292133	0.27692564	0.37754039
EC_3.2.1.40	1.25409549	1.92635733	2.11045307	1.23231316	-0.5730074	1.04837988	0.98097544	0.27938307	0.38041447
EC_2.7.1.180	-0.0787844	-0.2018133	0.75817839	0.01421216	0.30333134	0.89374547	-0.2443037	0.27950752	0.38041447
EC_6.4.1.1	0.92223302	-0.5472361	0.41455071	0.88198588	0.17446584	0.83067908	-0.3037308	0.28272924	0.38447346
EC_3.1.21.4	-0.4677118	0.23977827	0.00987938	-0.2130352	-0.57609	-0.9266451	0.26721929	0.28362184	0.38536098
EC_3.2.2.1	-0.1669752	-0.9980035	3.46032982	-3.188342	0.3295444	3.8638482	1.09190354	0.28395818	0.38549183
EC_3.1.3.48	0.85130322	0.33332213	2.08509711	-0.4196919	0.82037311	2.48014047	0.4512869	0.28483642	0.38635751
EC_4.1.99.1	1.49355775	0.24042459	0.25731324	0.91740597	0.12338535	-0.0642535	0.37005282	0.28577607	0.38730495
EC_3.2.1.41	0.31435493	0.71869757	-0.2809326	0.58369045	0.8961295	1.64629901	-0.4410852	0.28622236	0.38758272
EC_2.7.9.1	-0.1317665	-0.186028	-0.5101669	0.02570582	-0.2800818	0.15897554	-0.140307	0.28789885	0.38952448
EC_4.2.1.22	-1.288703	-1.1880753	4.42950392	-4.8923492	0.33480017	5.23135533	1.29659212	0.28882562	0.39029766
EC_2.6.1.2	5.30840496	3.7394673	6.5109712	2.23848074	4.13048976	4.45219977	1.7504796	0.28895636	0.39029766
EC_3.4.25.2	0.9240208	0.16914136	0.26028324	0.41992624	1.19422831	3.08370165	-0.4483729	0.29216587	0.39403777
EC_3.4.11.5	-0.5672394	-0.6635212	3.43713237	-0.4167001	1.10492254	3.43705898	-0.6989056	0.29224566	0.39403777
EC_3.1.4.55	-0.1566546	-1.3503267	-4.9872489	0.79050291	-1.1742704	0.24941697	-1.2467198	0.29246141	0.39403777
EC_2.3.1.89	0.3442793	0.29861695	3.985232	-1.5117587	1.16194915	3.53243487	0.74694685	0.2934495	0.39503764
EC_2.7.2.11	0.03485013	0.38120605	0.48876378	0.1897334	0.53889097	0.69811069	-0.1578246	0.29600483	0.39814385
EC_5.3.1.16	0.88542795	0.55245229	2.04611678	0.3164168	0.88682248	1.13618865	0.29863467	0.30257616	0.40664211
EC_2.7.7.23	1.4536341	1.02069039	3.65052106	0.00013786	1.69532513	3.2156869	0.59125378	0.30691176	0.41212398
EC_4.4.1.1	0.96621381	-0.5036722	5.11050071	-2.7715424	1.33230797	5.5110319	1.29164688	0.30764301	0.41276079
EC_6.3.2.13	0.04811863	-0.1328284	0.05434305	0.07556148	-0.3081945	-0.414383	0.09940793	0.30892366	0.41413306
EC_2.1.1.13	0.80328279	0.00319031	0.01639466	0.38144686	-0.0940961	0.07299076	0.23843725	0.31261286	0.41872915
EC_1.3.1.31	-0.425209	1.25119944	0	0.3709673	0.16720615	5.31555767	-0.7328585	0.31492808	0.42138007
EC_2.1.3.9	0.51563101	0.83533983	0.45359312	0.16235276	0.30900458	0.57314523	0.35687721	0.31511673	0.42138007
EC_1.1.1.132	1.8240072	2.08434624	4.00254324	2.6579336	2.9797781	7.45066672	-1.2096803	0.31675224	0.42319803
EC_2.7.1.85	-0.5366582	-1.5709195	0	0.11631058	0.25132687	0.47369709	-1.0447357	0.31700325	0.42319803
EC_3.6.3.4	1.16806342	0.73499202	2.05664329	1.22848447	1.65162294	4.09273501	-0.6198248	0.31834604	0.42463766
EC_2.4.99.17	0.26060116	-0.045539	0.06933836	0.14028669	-0.1372023	-0.112284	0.11259643	0.31863209	0.42466651
EC_1.3.1.26	0.82114231	0.97935819	0.45508512	0.30969356	0.26183859	0.69884231	0.4872051	0.32043866	0.42672013
EC_3.6.1.19	4.33215386	1.02749042	0.71741833	3.44284072	3.98055805	7.11939472	-1.3834189	0.3216974	0.42804145
EC_2.7.7.80	4.03737211	3.50787057	4.71490134	3.17379577	2.89485794	-1.0735534	1.40835595	0.3219905	0.42807678
EC_2.7.1.45	1.1541292	-0.2353522	-0.1972629	0.59517112	0.8779658	2.64454872	-0.4607132	0.32308184	0.4291724
EC_1.1.1.95	1.13602353	0.51773576	0.08447953	0.55147582	0.12339327	0.8601336	0.33649566	0.33074781	0.43899255
EC_1.2.3.3	-0.8059793	-3.826339	3.46970662	-1.6440316	0.4076191	3.96160134	-1.1166431	0.33150956	0.43964025
EC_2.1.1.228	0.16163905	0.00190101	0.26821114	0.15076111	0.22721282	0.41042859	-0.0918106	0.33292504	0.44115315
EC_4.1.2.17	2.79868949	1.68320655	2.86208689	1.45091148	1.32334359	2.08076851	0.91955221	0.33455738	0.44295066
EC_3.1.4.52	4.52818458	2.55047471	8.22349392	1.39735216	3.58550995	7.03070374	1.42590728	0.33596412	0.44444676
EC_3.2.1.51	0.7575127	0.44320202	-1.1464983	0.63126419	-1.7301212	0.36239528	0.59115507	0.33734365	0.44590444
EC_2.7.8.36	-1.3204638	1.23222499	0	0.12457232	0.75247679	2.37595662	-0.8806021	0.33959387	0.44830941
EC_3.6.3.44	3.18971293	2.74185282	4.79244914	1.32884804	2.65558319	4.51502165	1.0451883	0.33972139	0.44830941
EC_1.14.13.7	0.2874306	3.15269312	-0.8632748	2.72992217	0.21306805	6.01316128	-1.1745873	0.34068009	0.44920544
EC_3.5.1.59	1.20710765	0.96418587	4.6135695	0.69599628	0.55438777	1.06860136	0.84008587	0.34133123	0.44969479
EC_2.3.1.8	0.10251203	0.76686788	1.777458	0.50430056	0.8966472	2.10339956	-0.299847	0.3440491	0.45290397
EC_3.1.4.58	2.16726379	-1.8935135	7.4902954	2.48716627	2.03608228	5.80853884	-1.3941006	0.34534301	0.45423495
EC_3.1.1.47	0.38216155	-0.5920656	-0.3557656	0.16828948	-1.5392456	-2.8418708	0.73358987	0.34584503	0.45452301
EC_1.2.1.10	5.56709242	4.82504686	-2.4551302	1.94549201	4.14027341	4.36682088	1.3378406	0.34768659	0.45656964

EC_1.1.1.49	1.20464283	0.88049073	2.15760479	0.27704694	1.25603815	2.05428654	0.37516159	0.34998209	0.45920852
EC_1.1.1.251	5.79710036	5.17219826	2.24564345	2.49950939	5.22749922	5.75607148	1.31875937	0.35248015	0.46210868
EC_2.3.1.28	0.70630515	1.10975877	0.98760477	0.63500528	0.37842924	0.47267496	0.35413125	0.35295302	0.46235118
EC_3.5.4.28	0.0224764	-0.017717	-1.3538411	0.23007167	-0.1397277	0.349065	-0.2848946	0.35375506	0.46302414
EC_2.5.1.3	0.16352398	0.21243133	1.29343301	0.24919203	0.68051552	1.09698568	-0.1826298	0.3543056	0.4633671
EC_3.6.1.13	1.45334584	0.83628943	3.23073269	0.03856968	1.59941107	3.06559558	0.50471543	0.35559824	0.46467923
EC_6.1.1.11	0.40548477	0.41222321	0.88041256	0.36869591	0.91682594	1.72042759	-0.2598732	0.35615634	0.46503015
EC_2.5.1.48	2.34466428	1.48419849	2.86366017	1.32598595	1.32482599	2.49546952	0.62430048	0.35731464	0.46616354
EC_2.4.2.53	1.36588039	0.73881008	0.61821315	0.68572673	0.60895307	1.0439163	0.34453755	0.35827479	0.46703678
EC_4.3.1.7	5.54833345	2.694856	1.30210593	2.16769586	3.10722197	5.78411872	1.06646048	0.35974242	0.4685696
EC_2.1.1.104	0.55666585	-1.2784999	-0.1297041	0.24875414	-2.3833301	-2.6986794	0.82392395	0.36611698	0.47648611
EC_1.15.1.2	-0.7575371	0	0	1.10443518	0.32359254	-1.0741503	-0.9586443	0.36706905	0.47733837
EC_2.7.8.5	0.50446006	0.81894675	1.55127731	0.60845197	1.22864873	2.48566666	-0.3141994	0.36756697	0.47759916
EC_5.4.2.8	0.8332761	-0.1549106	-1.1347619	0.05697702	-0.5022624	0.49367561	0.31108685	0.3681166	0.47792664
EC_1.17.1.5	5.91273362	3.51504274	5.77008917	3.16251365	3.70853359	4.79649528	1.48983884	0.36851353	0.47805551
EC_3.5.4.31	0.03320141	-0.0204798	-1.3994998	0.22901903	-0.1556226	0.31554884	-0.2756748	0.36936589	0.47877451
EC_2.7.7.65	5.5628056	2.93729406	11.668523	2.50144433	3.92172147	9.6473617	1.47555604	0.37015435	0.47940958
EC_2.1.1.151	-0.5320209	1.1473101	0.57276985	-0.1233268	-0.2195721	0	0.33689674	0.37174841	0.48108618
EC_3.5.1.88	0.05384114	0.04303016	0.34755329	0.05219248	0.28463848	0.37851703	-0.0852573	0.3752613	0.48524126
EC_3.1.3.16	1.91036421	1.16737315	3.89091236	0.77359443	1.59532229	3.20392399	0.52772423	0.37762762	0.48790825
EC_3.1.1.81	-1.5714363	1.17456177	1.70414974	-0.8112959	-2.5367462	1.01488587	0.93990039	0.37846344	0.48859508
EC_3.5.1.1	1.1895764	0.31080301	1.19134489	0.28824146	0.67663026	1.5503894	0.29717539	0.37910538	0.48903072
EC_6.3.5.9	1.26318151	1.40733844	-2.6776062	0.18171915	-0.7577469	0	0.9814265	0.38210896	0.49250962
EC_1.1.1.1	1.63468022	1.12227195	3.34638965	0.01171511	1.9815066	3.54211174	0.54171717	0.3835027	0.49324081
EC_3.6.4.12	0.27428016	0.10296125	0.47618779	0.24397594	0.36060323	0.66103521	-0.0986457	0.38355856	0.49324081
EC_4.2.1.17	3.20220955	1.47532765	2.20790989	1.38669593	1.66106979	3.41978893	0.7250029	0.38359762	0.49324081
EC_2.4.1.7	2.04874289	1.99131646	6.06444006	-0.5071319	2.85977574	6.35373809	1.02265756	0.38466699	0.49422015
EC_6.3.2.2	1.18751279	0.84109493	3.13143514	-0.3257461	1.62269825	3.40951633	0.49936009	0.38626507	0.49587666
EC_6.3.5.5	0.03707062	-0.0062464	0.18056546	0.10468571	0.08589841	0.09738504	-0.0582511	0.38713981	0.49660266
EC_4.1.3.16	-0.0769703	-0.4200838	0.39603985	-0.2663517	-0.5244933	-0.0703003	0.18472194	0.38765927	0.49687213
EC_1.1.1.100	1.14568402	0.58883734	0.1127233	0.57441815	0.42389124	0.9331129	0.24967251	0.38887079	0.49802751
EC_5.1.3.21	0.32258748	0.62328623	5.99157555	-2.1535853	1.81749363	5.8544303	0.92367635	0.39022786	0.49936728
EC_3.6.3.34	1.17671576	-0.2721426	-0.1201058	0.38190878	-0.1627103	0.11674301	0.33607907	0.39069737	0.49957004
EC_6.3.4.19	0.31654001	-0.0369727	0.55444078	0.19299075	0.52201993	0.9560393	-0.1806084	0.39189766	0.50070616
EC_1.4.3.5	3.84340354	2.50465448	4.7950444	2.33774603	2.25648122	2.42252582	1.16362507	0.39483653	0.50405999
EC_4.6.1.17	0.85258306	0.81172048	2.72500733	0.91565034	1.56155595	3.45777395	-0.3937024	0.39636275	0.5056065
EC_5.3.2.6	-0.1480579	1.18208018	2.68965303	-0.590518	1.09030574	0	0.6327109	0.40069168	0.51072288
EC_1.8.1.14	1.32749584	0.82610148	0.97224499	1.60253755	1.30695626	3.5125007	-0.6501009	0.40410026	0.51465902
EC_1.1.1.131	-0.6113363	0.00525045	0.88138794	-0.1202065	0.12417698	0.16139532	-0.2068929	0.40782431	0.51899036
EC_5.4.2.2	0.99644436	0.91267179	2.33040707	0.26125021	1.11739988	2.38716624	0.30908655	0.40858971	0.51949883
EC_3.1.3.27	1.99577903	1.59537617	6.40694734	-0.3490441	2.31065054	7.14884198	0.88682688	0.40887081	0.51949883
EC_2.4.99.12	0.69177181	-1.303067	-5.9690738	0.54548503	-1.5872912	1.9869827	-0.8666706	0.41393872	0.52552221
EC_2.7.7.22	1.32367648	-1.090879	-0.8996129	0.81362823	0.31727792	2.49350639	-0.675562	0.41593072	0.52763408
EC_6.2.1.1	5.2411545	4.40479702	8.66799167	3.64715685	4.47436828	5.4231967	1.21328186	0.41727342	0.52846527
EC_6.1.1.18	-0.024246	-0.1616895	-0.5366028	-0.0561413	-0.4306919	-0.3391015	0.08039146	0.41739888	0.52846527
EC_3.2.1.49	-0.419341	0.66285134	1.90657879	0.03906258	1.37905486	6.33657973	-1.0564712	0.4176985	0.52846527
EC_4.1.3.32	-1.3253129	1.3448706	2.00834229	0.03496246	1.14478427	3.71381776	-0.8529693	0.41790217	0.52846527
EC_2.1.1.10	-0.2069143	0.98102987	0.1656824	0.21583966	-0.7724739	-1.5216085	0.59593577	0.41896149	0.529388
EC_3.4.13.9	1.59612497	0.49286166	4.1952938	-0.8441693	2.11098692	4.3600191	0.71561393	0.4209853	0.53152703
EC_1.1.1.58	0.27719379	0.24464277	0.85841772	0.18212465	0.0904043	0.33760316	0.1644337	0.42388069	0.53455818
EC_5.1.3.22	3.95790994	3.09844368	2.35555976	1.99033861	2.09789237	5.55266666	0.96363062	0.42405175	0.53455818
EC_3.2.1.85	1.68339241	1.94940682	7.80541779	-0.2482472	2.09567094	6.98476743	1.05771185	0.42679175	0.53759023
EC_3.4.14.12	0.22333328	0.21324555	-1.2333917	0.43333606	0.10894252	0.88264695	-0.3447645	0.42844933	0.53912984
EC_4.2.1.136	0.06761053	-0.0088973	1.53254575	0.03110346	0.77157842	1.09353997	-0.1933735	0.4289282	0.53912984

EC_2.1.1.177	0.32821388	0.11713105	0.66806188	0.26147367	0.53186818	1.04656471	-0.1581422	0.42902113	0.53912984
EC_3.2.1.25	-0.1818038	1.14881017	5.48905709	1.01994111	2.06958142	4.94579493	-0.8951554	0.43160974	0.54195875
EC_1.3.1.9	0.42342415	0.0500469	-0.7120613	0.14715743	-0.293494	-0.0261538	0.17209382	0.43312373	0.54343493
EC_4.1.2.48	3.88748068	0.88236015	1.38078876	2.4494025	3.72372536	7.18973953	-0.9739887	0.43429158	0.54436695
EC_3.4.11.9	1.05039226	0.2808244	-0.5645557	0.55699069	1.16740943	1.45078209	-0.2946588	0.43482907	0.54436695
EC_5.5.1.4	-0.0468106	-0.7202177	1.27641291	-0.1789573	1.08221594	-0.0370176	-0.3763674	0.43488343	0.54436695
EC_3.11.1.3	-0.9013186	-1.4003549	-2.9297731	-0.993687	-3.4983197	-1.2640011	0.5237528	0.43634677	0.5457733
EC_1.5.1.2	0.30624771	0.63607259	1.07455887	0.5111112	0.844935	1.2160516	-0.195913	0.43874063	0.54834043
EC_4.3.1.17	1.248387	0.37348152	0.76800384	0.37877507	0.6103785	1.53664939	0.27283073	0.43926867	0.54857347
EC_3.5.1.26	0.80002063	-0.7138107	-0.3109397	1.73364915	0.17499077	1.31418063	-1.0398231	0.43999126	0.54904893
EC_4.1.1.83	2.61008594	1.79975564	3.787169	2.25788523	3.55606971	6.22080759	-0.7366858	0.44129784	0.55005952
EC_2.7.1.49	1.66634952	0.55955441	2.85495345	-0.1119962	1.59271956	3.7417322	0.46690384	0.44164017	0.55005952
EC_1.1.1.2	1.44490195	1.97097786	2.49437922	0.77875867	0.76927001	1.93461342	0.83241832	0.44190143	0.55005952
EC_2.8.3.12	-0.5534168	0.01579504	2.05059844	-0.2427499	1.36749782	2.37594211	-0.6618065	0.44217114	0.55005952
EC_1.1.1.202	-2.3863396	-1.8575048	4.75824908	-2.9101947	1.16679488	6.67430106	-1.0087495	0.44418903	0.55214209
EC_5.3.1.8	1.47579064	0.27160477	2.57113051	0.11013691	1.21463503	2.5947329	0.38490908	0.4476326	0.55588699
EC_3.2.1.86	1.63735592	1.03392412	3.70309175	0.46622399	1.74438807	3.19443313	0.43317325	0.44791629	0.55588699
EC_1.3.1.14	1.83562577	1.36490359	-0.1734742	0.77737471	0.81573686	1.80923712	0.49717411	0.44824013	0.55588699
EC_1.1.3.15	2.5666208	2.44626155	10.2437198	4.27635199	4.01520232	7.85819953	-1.1961923	0.44906863	0.5561353
EC_3.5.1.28	1.54572944	1.13817274	1.42534461	0.91998359	1.03986687	1.77143162	0.31550039	0.44913293	0.5561353
EC_2.1.1.171	1.53026292	1.10982306	1.42217649	0.95414888	0.80869343	1.88637292	0.33920802	0.45306059	0.56000859
EC_6.1.1.1	-0.098507	-0.083834	0.10594099	-0.0465807	-0.0214989	0.08213087	-0.046799	0.45325433	0.56000859
EC_1.18.1.2	1.10139887	0.67262443	3.31751672	-0.1362398	1.43054172	3.34979577	0.38580978	0.45330708	0.56000859
EC_2.3.2.29	2.25866316	1.31631693	3.28044165	1.20557047	0.79151711	2.58734458	0.79884313	0.45609753	0.56302278
EC_6.3.3.3	0.7412133	0.25081748	1.33413306	0.56698368	0.2881437	0.58912934	0.16172656	0.45732986	0.56411041
EC_4.2.1.106	-0.1452153	3.04660299	0	2.5342542	1.01857298	1.43788079	-0.8901505	0.46152086	0.56884305
EC_3.4.21.108	0.58044007	-0.955984	-0.8971403	0.2198461	-1.5867422	-2.7461517	0.61720402	0.46336396	0.57067679
EC_2.3.1.79	1.65168062	0.82763061	2.97705218	0.1955904	1.55257987	3.59233898	0.43559552	0.46710876	0.57484802
EC_2.7.10.1	-0.5399191	-2.8471057	2.54753648	-0.9767973	-0.1884038	3.13754086	-0.8007723	0.46824236	0.5757318
EC_2.3.1.180	0.03960661	-0.2043366	-0.1029334	0.02157249	-0.3189462	-0.3043486	0.06861872	0.46860711	0.5757318
EC_4.1.1.20	0.86478549	0.51108152	1.3099013	0.37591156	0.77687223	1.09760187	0.1918919	0.46890236	0.5757318
EC_2.5.1.74	0.63945746	-0.0715823	0.62617011	0.1318215	0.26205177	0.36794313	0.18044434	0.46964341	0.57603099
EC_5.3.1.17	0.13942857	-0.0880432	0.2119037	0.16611773	0.1092538	0.74600174	-0.1558896	0.46986339	0.57603099
EC_2.3.1.157	1.11466438	0.85467567	3.3582282	-0.0660327	1.5722517	3.18219203	0.39980314	0.47107232	0.57695718
EC_6.4.1.2	1.22267193	1.04189296	1.44085319	0.8024197	0.82677707	2.05567586	0.21037989	0.47133737	0.57695718
EC_2.7.1.167	0.59242972	0.26689826	1.07553891	0.69316124	0.7652287	1.96190953	-0.3470824	0.48093415	0.58825609
EC_4.1.2.25	0.18127095	-0.1764549	0.53402441	0.16080179	0.13883665	0.72031958	-0.1292828	0.48600911	0.59401114
EC_4.4.1.25	0	1.40464363	2.15267103	1.79653811	0.51594882	1.35665556	-0.5574935	0.48983862	0.59779506
EC_4.2.1.40	1.43761322	0.8937065	1.99298954	1.56664441	1.60584583	2.80381129	-0.4289485	0.49008245	0.59779506
EC_3.1.3.18	2.69548796	0.36728065	0.74081954	1.89517185	2.09965669	4.57137898	-0.6824399	0.49022173	0.59779506
EC_4.1.3.39	2.95294149	0.92350315	0.88035575	0.40965391	1.08305992	4.01933238	0.87554757	0.49062849	0.59783714
EC_5.1.3.4	1.08899433	0.90984884	1.46083753	0.53879009	1.15599104	1.24083184	0.23774579	0.49128212	0.59817975
EC_3.1.2.6	6.57931074	4.16743852	-0.136611	3.0692074	3.67700601	8.21800052	0.95715917	0.49355616	0.60010408
EC_1.1.1.44	0.97273539	0.86472905	2.03748696	0.23187725	1.23808162	2.07663299	0.26089397	0.49360989	0.60010408
EC_3.1.1.31	1.88631201	0.97315552	3.10267544	-0.439221	2.10270266	5.18487156	0.57905208	0.49912038	0.60634442
EC_2.7.7.75	6.90745643	4.08920566	7.84367293	5.45955425	3.67454941	7.88689211	0.83093423	0.50207916	0.60947781
EC_3.6.3.14	0.73663019	0.3225905	-0.0461301	0.52701503	0.66987647	1.31472104	-0.1832749	0.50726752	0.61492311
EC_2.7.7.18	0.5469128	0.30230753	1.19680676	0.15111901	0.53520994	0.83731413	0.1727468	0.50733071	0.61492311
EC_3.4.14.11	-0.1916176	0.27090425	3.36638058	-0.8545283	2.12144659	5.7652519	-0.5823907	0.50775155	0.61496907
EC_6.3.4.4	0.16062185	0.06814165	0.27897795	0.10756979	0.23698547	0.47685326	-0.0561325	0.50908651	0.61612128
EC_4.4.1.5	1.40025246	0.47603983	0.42579159	0.64349803	0.44659671	1.19516283	0.30207284	0.50968916	0.61638614
EC_4.99.1.3	-0.4451572	1.69143712	2.81974586	0.43598585	0.08030227	-1.3999088	0.63355117	0.51031767	0.61668185
EC_2.7.7.38	0.66564241	-0.0281552	0.20331835	0.25325732	0.01486296	0.50848102	0.15462586	0.51140651	0.61753297
EC_5.4.99.1	0.31915067	0.34669517	2.29520986	0.99240141	0.92472764	4.36282635	-0.8417573	0.51218026	0.61800263

EC_2.7.1.26	0.34881495	0.1536955	1.5007313	0.38259592	0.56396947	1.30730149	-0.1426228	0.5129419	0.61845698
EC_5.3.1.5	0.18230594	-0.002472	0.80974522	-0.1498581	0.20072211	0.48734845	0.14497767	0.51538754	0.62093953
EC_5.4.2.12	0.0120102	-0.0131019	-0.3616008	0.05078926	-0.2856074	-0.2741747	0.05951644	0.51680132	0.6221761
EC_6.3.5.2	0.01831679	0.10235982	0.09360069	0.09891242	0.08946901	0.09300302	-0.0377277	0.519032	0.62439355
EC_4.1.1.36	0.27267678	0.08951159	0.42486843	0.16384025	0.06954778	0.32052377	0.07295353	0.52022692	0.62521248
EC_2.3.1.181	0.59519995	0.0627631	-0.2115077	0.31397506	-0.1212268	0.13956457	0.15936794	0.52049932	0.62521248
EC_2.7.7.24	-0.0051887	-0.076425	-0.1653952	-0.0282358	0.03144745	0.09160341	-0.0571298	0.52090571	0.62521248
EC_2.5.1.61	0.55563673	0.77247387	1.67890236	0.89084433	0.95644965	1.76448196	-0.2569791	0.52128677	0.62521248
EC_2.1.1.77	5.56379324	1.4933123	1.43074579	2.0969093	3.32841307	3.07632992	0.9861072	0.52165923	0.62521248
EC_1.1.1.346	2.60091213	2.14415034	6.41741664	0.79518079	2.3819737	5.11178401	1.02450028	0.52216265	0.62534916
EC_2.7.4.9	0.39813103	0.14431158	-0.3867969	0.2233838	0.3884302	1.0152711	-0.1694822	0.52477023	0.62713339
EC_4.1.1.33	-0.5196826	-1.3090921	2.31430316	-3.9791737	0.75221211	5.35251271	0.75025464	0.52486887	0.62713339
EC_1.1.1.272	1.05572702	0.13051909	-2.5739977	0.7231933	0.44141496	-0.1100418	-0.2382324	0.52491031	0.62713339
EC_1.7.99.4	2.35716707	1.32471915	4.67822255	-0.2388249	2.76599038	6.4103536	0.65624687	0.52521445	0.62713339
EC_3.6.3.36	3.58637516	1.14840498	5.87133243	2.57979327	1.50919048	3.2982829	0.67980016	0.52872668	0.63085814
EC_3.2.1.15	0.12958655	1.41418133	-0.212412	0.88039868	0.86642143	2.10777327	-0.4943404	0.52955904	0.63138219
EC_2.7.7.7	0.37852608	0.20977186	0.60526864	0.35334929	0.41988479	0.79501677	-0.0866277	0.5300541	0.63150362
EC_5.3.4.1	-4.0519716	-1.9224323	6.32176482	-4.5641472	1.24102197	7.56060853	-0.9579624	0.53262885	0.63359291
EC_1.2.7.3	0.17288643	-0.9157203	-0.6726997	0.06950808	0.06665282	-0.2859803	-0.3382226	0.53295409	0.63359291
EC_3.6.1.11	1.32465022	0.87681969	3.82371061	-1.6493621	2.599597	6.40260012	0.67097049	0.53338125	0.63359291
EC_1.16.3.2	0.22255335	-0.1211625	-0.2254325	0.2355245	-0.1008421	0.40144671	-0.1011209	0.53338581	0.63359291
EC_3.6.3.3	0.67953413	0.68866732	1.69156492	0.4982663	1.2291716	2.68573323	-0.2184073	0.53502342	0.63490229
EC_4.3.1.19	1.21731371	1.06534893	1.89222311	1.1508781	1.64903705	2.56775018	-0.2540977	0.53527876	0.63490229
EC_6.3.5.4	0.20776728	0.81603543	1.59713084	0.33670456	0.91618558	2.42951944	-0.2080677	0.53666955	0.63608214
EC_4.2.2.2	0.4093965	0.95157583	-0.1998042	0.70125991	1.059723	1.70694194	-0.4241115	0.53799383	0.63718148
EC_2.4.1.8	-0.0600692	-0.901989	-1.9649913	-0.0640188	-0.7361408	0.08775008	-0.327284	0.53951214	0.63850884
EC_2.7.1.92	1.27413469	0.88686212	1.57831565	1.14960235	1.33198206	2.34864026	-0.1926336	0.54186195	0.64081759
EC_5.1.99.6	0.01931004	-0.0316234	1.43047924	0.10529658	0.44049902	0.79648085	-0.1317526	0.54608751	0.64533961
EC_2.4.1.212	0.89580704	0.83826157	1.83605747	0.92487322	1.20050224	2.74097912	-0.2631003	0.55003429	0.649207
EC_4.1.1.5	0.13422699	0.29117315	1.2258826	-0.2882881	1.04039071	4.23466314	-0.4177832	0.55016857	0.649207
EC_3.5.1.100	1.00683851	0.36204342	-0.7868068	0.39006752	0.09041537	0.69661214	0.22653839	0.55204371	0.65094141
EC_4.1.99.22	1.1464323	0.93827421	3.33903671	0.67623312	2.35512093	4.93518559	-0.4446394	0.55407532	0.65240707
EC_4.2.1.32	2.28478	1.44305411	2.16923611	1.30192069	1.12011879	3.435449	0.44996529	0.55409915	0.65240707
EC_2.1.1.166	5.02791887	3.01163064	5.04496274	2.27018578	3.82383467	7.73291365	0.81877199	0.5581733	0.65652728
EC_2.1.1.191	1.09447227	0.56618159	-0.0403863	0.91104447	0.81865123	1.7516627	-0.2228954	0.55841611	0.65652728
EC_3.4.13.3	-0.5055638	-0.6235253	0.06410876	-0.5615142	-0.361541	1.02203989	-0.1895542	0.5588538	0.65656123
EC_2.1.1.226	1.88804795	1.99029016	2.48538491	2.21053057	2.2442648	6.01080873	-0.7232232	0.5610917	0.65870854
EC_3.1.5.1	1.02149475	0.3215649	1.79129277	0.38906328	0.79608036	1.6107809	0.18495507	0.56261696	0.66001668
EC_4.1.2.9	-2.0888274	-1.4050138	3.09190474	-2.7057732	0.83439278	4.80997576	-0.6537488	0.56578828	0.66325254
EC_2.7.8.33	3.1171794	2.70774525	4.61444792	2.47928705	2.23459128	2.62947527	0.73689706	0.56687733	0.66404449
EC_3.2.2.24	0.42930516	1.28914689	0.68859075	0.53269913	0.38057179	0.55297635	0.28016055	0.5682741	0.66519548
EC_2.5.1.10	1.18937765	0.21919785	0.96499678	0.6208427	0.41401899	1.22066584	0.1864356	0.56896981	0.66552477
EC_4.2.1.90	-0.4191888	3.10126143	7.17096765	2.34918285	2.37694355	2.21631425	-0.582779	0.57017863	0.66645334
EC_5.3.1.28	2.49647976	1.83655784	1.41803061	1.62085247	1.38770151	2.3111615	0.49548922	0.57087329	0.66678
EC_3.4.13.20	0.51355168	0.11920666	-0.4340274	0.79499582	0.16477918	-0.2783534	-0.1896488	0.57139594	0.66690543
EC_4.1.1.31	1.81756093	0.90139962	4.39739532	-0.3640242	2.80705563	4.3428318	0.51532247	0.5744686	0.67000478
EC_1.1.1.88	0.3227248	0.58762768	3.96424086	-1.0029498	0.98491635	4.5712927	0.48192678	0.576378	0.67174388
EC_2.7.1.4	3.99701531	0.47246573	4.6284945	1.1626586	2.51871633	4.50841155	0.77930093	0.5781283	0.67325755
EC_5.1.3.14	1.0259737	0.94792948	1.32565983	0.84579712	0.62820107	1.591972	0.16261581	0.5785152	0.67325755
EC_2.1.1.72	0.5425424	0.30886088	1.0660919	0.47052172	0.59991203	1.50754066	-0.1252023	0.58042113	0.67498648
EC_3.5.1.77	2.6122571	2.3200728	8.30090338	0.83762227	3.90605978	6.18595916	0.65983017	0.58333677	0.67788629
EC_2.7.1.24	0.32366865	0.1854474	1.14851308	0.28211861	0.66932703	1.01184202	-0.1291116	0.58415397	0.6780938
EC_2.7.7.41	0.84334668	0.51280539	2.20752714	0.46545977	0.83686868	1.59408526	0.15800151	0.58435979	0.6780938

EC_3.4.23.43	1.9861927	-1.1890184	6.47490969	-0.4970917	1.23019209	4.62165439	0.6701554	0.58638747	0.67995544
EC_2.6.1.62	0.84400484	0.5600767	0.53223325	0.79761411	0.25187623	0.55866992	0.11793831	0.58992259	0.68356109
EC_5.1.3.3	0.10870154	-0.1796939	0.43039981	0.19576685	-0.0046804	0.31437828	-0.0996368	0.59202649	0.68550435
EC_2.4.2.2	1.86113751	1.43469749	2.02676694	1.59792636	2.10473154	4.38228285	-0.3993949	0.59257103	0.68564054
EC_1.2.7.5	1.00206834	2.17561606	6.3465466	-0.2672261	2.04195973	6.27798735	0.72323347	0.59313653	0.68580077
EC_1.1.1.26	0.84447755	-1.6387877	0.18034852	0.39023861	-1.4648474	-2.6991967	0.50729451	0.59457381	0.68696802
EC_1.8.1.4	0.70279341	0.0204801	0.63039058	0.28167137	0.33287794	0.55731643	0.11758059	0.59637685	0.68798042
EC_2.1.1.63	0.6336272	0.40344002	-0.0646623	0.54940084	0.68207178	0.70157243	-0.146381	0.59668386	0.68798042
EC_1.1.1.14	1.35007065	1.17082006	1.97781195	1.27607671	1.59271252	3.13387886	-0.2603736	0.5967352	0.68798042
EC_1.1.1.140	2.60568733	1.90320713	2.23945819	1.740883	1.27057143	3.68056136	0.4705661	0.59846367	0.68947823
EC_2.7.8.13	0.38489421	0.18576047	0.49370164	0.24848464	0.27437108	0.33400743	0.05949251	0.60726599	0.69911769
EC_4.2.1.6	2.66605339	2.57209453	4.89945578	2.28968999	4.26294475	6.88016048	-0.6330873	0.60878086	0.70035965
EC_2.7.1.53	4.40047577	1.89245896	7.13605634	1.89652779	3.46432502	6.98538832	0.76848235	0.60999126	0.70118211
EC_1.3.1.2	-0.9591273	-0.2536128	5.49191199	-3.7831509	1.91920687	6.43052141	0.65340576	0.61036899	0.70118211
EC_2.1.1.173	2.15749424	1.1108938	2.48911889	1.8362013	2.02789664	4.01556369	-0.3578479	0.61485471	0.70583035
EC_4.2.1.1	2.70644894	1.92023203	1.99843356	2.25724988	2.31047644	5.84093039	-0.4041713	0.61608997	0.70674321
EC_3.1.11.2	0.47478843	0.84820064	2.08971992	0.4563096	1.20533951	2.68373288	-0.1894637	0.61823722	0.70870019
EC_3.2.2.23	1.61991272	0.96859674	4.05345012	0.05313386	2.05031148	4.64905173	0.3758828	0.62471708	0.71561742
EC_6.3.4.14	1.20548308	0.99522848	1.5554705	0.86576003	0.8298967	2.20256011	0.14433958	0.62563928	0.71616299
EC_2.5.1.145	0.41374362	-1.0183617	3.63372286	-2.0079591	0.85410422	4.45096897	0.52237206	0.62725012	0.7174955
EC_2.3.1.117	0.80010092	0.2634138	0.39377946	0.3503468	1.4285981	1.55708423	-0.302184	0.62932994	0.7193622
EC_1.2.1.12	0.49066406	0.56747918	1.53069373	0.28714144	1.1711311	2.10437417	-0.1703193	0.62999178	0.71960654
EC_3.4.21.26	-0.0552099	0.63272361	5.65582213	0.25665508	-0.9243914	1.68856004	0.83098915	0.63116103	0.72042971
EC_3.5.1.47	0.32992825	-0.0247464	2.04501031	0.03665483	0.81313393	2.39270957	-0.1861762	0.63483839	0.72411254
EC_2.7.1.5	1.14216086	0.58099693	0.73239902	0.66680309	0.56011885	1.35486271	0.17098607	0.63558711	0.72445203
EC_4.2.1.7	1.49852851	0.04916475	0.42991449	0.43855223	0.52591173	1.65294054	0.2274995	0.64009241	0.72906271
EC_5.3.1.13	1.32139912	0.15374862	-0.3312642	0.54722846	-0.0451231	0.97193985	0.29647874	0.64054016	0.72906271
EC_4.2.1.109	0.01383584	1.3941104	0	2.10666538	-0.6675428	0.84224735	-0.529915	0.64293377	0.73126886
EC_1.1.1.283	-1.2781046	-2.0619101	3.36826718	-3.1138094	1.34975956	6.41635385	-0.5876582	0.64453999	0.73257695
EC_2.6.1.16	0.16178233	0.07511093	0.64074371	0.10561623	0.36319718	0.67354218	-0.0748764	0.65066497	0.7387136
EC_2.7.7.71	0.83766754	1.58266216	-0.4467572	1.66633518	0.48406067	2.20394209	-0.4051604	0.65085912	0.7387136
EC_2.1.2.10	0.33677496	-0.237339	0.0653694	0.12891455	-0.1898295	0.02847879	0.08895018	0.65286145	0.74046292
EC_2.7.1.191	2.61427435	1.57478037	2.21207277	1.60192483	1.64338516	4.03959286	0.26112018	0.65445243	0.7410967
EC_3.5.2.15	-1.3349887	0.82838707	0.35432111	0.24361553	0.16976607	-0.9751117	-0.4210079	0.65470878	0.7410967
EC_1.5.1.36	0.89032082	-0.6501749	4.45520187	-1.659809	1.91689304	4.31482103	0.48395761	0.65480462	0.7410967
EC_1.1.1.31	1.4285536	3.32151311	7.24012019	3.20579335	3.2966893	5.23737582	-0.6910689	0.65585348	0.74176105
EC_2.2.1.3	0.4437136	-1.3997352	-0.4123635	0.20787547	-1.363499	-2.3698401	0.33175513	0.65663433	0.74212155
EC_2.7.7.8	0.09533612	-0.0128589	-0.2106299	0.11972436	-0.0093504	-0.0675874	-0.0332884	0.65738724	0.74217344
EC_1.2.1.24	-2.147665	-1.4305117	2.34557096	-3.6554321	1.00086325	6.28787766	-0.5201813	0.65760449	0.74217344
EC_3.5.1.104	0.71446727	1.07020835	-0.097965	0.95647003	0.91432858	1.78814074	-0.3109858	0.65876971	0.7429664
EC_4.1.2.19	1.18630766	0.96691668	0.85289776	1.05711616	1.11607023	2.13153439	-0.1498207	0.66586231	0.75043851
EC_1.8.4.8	4.69138828	2.11737331	5.28835798	2.44021283	3.23757641	5.99879978	0.68381091	0.66874045	0.75315369
EC_5.4.99.23	2.9042842	1.78303118	5.41357038	1.88076726	2.36407414	4.42965488	0.44069373	0.67041564	0.75402426
EC_2.7.7.4	1.02014899	0.48659458	0.90507614	1.0828981	0.55685395	1.34835184	-0.1282126	0.67069777	0.75402426
EC_3.4.24.64	0.42968239	-1.4126378	-0.2484755	0.26409905	-1.3476259	-2.5793116	0.33183291	0.67092196	0.75402426
EC_3.2.1.4	3.01000841	1.56916363	4.17801415	1.7110651	1.53554329	5.37940746	0.50752948	0.67298412	0.75581294
EC_2.7.1.16	1.79486997	0.62769617	1.41716546	0.74335497	1.0869954	2.81587133	0.20693691	0.67477346	0.75729292
EC_1.3.1.98	0.3955375	0.16111297	0.89455004	0.22435192	0.62264354	1.18865895	-0.1079305	0.67754479	0.75987215
EC_5.1.3.20	2.7998596	0.4579361	5.26477621	2.36090595	2.48109448	5.50404115	-0.5406482	0.67843895	0.76021858
EC_2.5.1.30	0.24510047	0.12692797	2.85595102	-0.7344495	0.98522022	2.58100165	0.2583893	0.67920006	0.76021858
EC_1.1.1.77	1.5271997	1.21692492	1.89235895	1.07541717	2.27743253	2.91577724	-0.2492007	0.67938885	0.76021858

EC_3.2.1.99	-0.1071924	0.3891491	3.19262456	0.08250633	1.31886876	2.62411222	-0.3486464	0.67974712	0.76021858
EC_2.7.1.59	2.52378478	-1.0588887	-2.0235129	0.58257768	-0.0842794	-0.4994214	0.47564134	0.68331117	0.76321058
EC_5.4.99.5	0.60262535	-0.2753848	-0.2414065	0.86156861	-1.3460968	-1.2862195	0.33258206	0.68337286	0.76321058
EC_3.1.3.25	0.42008682	0.28068516	0.37720044	0.58517054	0.24485802	0.35508469	-0.0779103	0.68701623	0.7667464
EC_4.1.2.42	-0.7337194	2.55573582	0	1.53900981	-1.126914	3.67318534	-0.4238165	0.69073634	0.77036289
EC_3.5.4.12	-0.1175392	-0.1464061	-1.7327482	-0.3190753	-0.8311622	0.01925067	0.11681825	0.6917572	0.77096604
EC_4.1.99.2	0.89827926	0.17498642	-2.3254993	0.63558643	0.13112621	0.17644193	-0.1629135	0.69276495	0.77155375
EC_6.1.1.21	0.30619679	0.08447552	0.61071597	0.19517669	0.38136643	0.78513089	-0.0687219	0.69334512	0.77166477
EC_3.4.24.71	0.16736506	-0.5244062	-1.3834716	-0.1358079	-1.0261849	-0.9113961	0.26188026	0.69424695	0.77213338
EC_2.7.7.13	1.09241913	0.34676767	0.13444158	0.78421198	0.24750542	0.62486931	0.12359321	0.69484508	0.77226381
EC_2.4.2.6	-0.67003	-0.828301	3.66005344	-2.2233371	0.50645567	3.4373019	0.38944421	0.69825474	0.77551667
EC_2.3.1.275	-0.1377322	-2.2572566	3.81007387	-2.762759	0.89791604	3.05143759	0.39423276	0.70104761	0.77808049
EC_1.1.1.25	0.18219059	0.01723157	0.48611797	0.15310102	-0.0241501	0.21644077	0.05771419	0.70271933	0.77939727
EC_1.4.1.13	1.24320774	0.75179	0.95071955	1.05417566	1.11359183	1.68967908	-0.1238232	0.70429564	0.78060648
EC_2.1.1.189	1.74715836	1.84521263	5.38780201	2.30473399	2.461014	5.42436593	-0.5375806	0.70652759	0.78254022
EC_1.3.99.1	0.24703879	-0.1973127	-1.3788158	0.14792921	-0.3696846	0.16856691	-0.0905419	0.70980331	0.78562654
EC_7.1.1.1	4.76911195	3.7763283	6.79036527	3.06044392	5.07121806	5.87319091	0.5760252	0.71029558	0.78562996
EC_3.1.7.2	0.22871355	-0.7684288	-1.7726305	-0.2471519	-1.1764779	-0.1095815	0.16403524	0.71182807	0.78678313
EC_4.2.1.96	0.51874807	0.59124142	0	0.74835467	0.52342128	1.10090406	-0.2400577	0.71274167	0.78725111
EC_2.5.1.129	1.11175651	0.07435375	2.76605539	-0.2271505	1.59642665	2.5562818	0.21071505	0.71512264	0.78933811
EC_1.6.99.1	0.58253125	0.94430927	0.31569663	0.45219108	0.48957387	0.80987831	0.16654228	0.71653377	0.79035249
EC_4.1.99.19	1.74128491	0.89419989	1.19136931	1.12848589	0.99321584	2.13465857	0.15757001	0.71764381	0.7910336
EC_2.7.1.11	-0.0515697	0.01002311	-0.1056904	0.04623746	-0.1776457	-0.1736708	0.01966784	0.72664382	0.80040464
EC_2.3.1.29	0.70300552	0.09564354	-0.2040163	0.48511264	0.01926276	0.23932989	0.07662532	0.7293263	0.8028088
EC_3.5.2.9	1.67160418	1.50352387	1.93603505	1.30661524	1.30764135	2.58454748	0.16969258	0.73149928	0.80464921
EC_1.2.1.70	0.66016069	0.96583063	1.55145862	0.94182522	0.93977526	1.55977507	-0.1446385	0.7328679	0.80524595
EC_6.3.2.17	1.32686132	0.64070572	1.42703331	0.80821512	1.63935428	2.04007019	-0.1464255	0.73342149	0.80524595
EC_2.9.1.1	2.76276749	1.9057355	5.6003323	2.55139183	3.31314159	5.23128676	-0.3461309	0.73354597	0.80524595
EC_1.7.1.7	1.0014408	0.5999472	4.01546172	-0.1953386	2.66440249	6.016559	-0.3295477	0.73539931	0.80672902
EC_6.1.1.4	0.15053969	0.11418406	0.58716434	0.18136528	0.23600223	0.31989716	-0.0262963	0.73862975	0.80971971
EC_5.4.99.25	0.3439777	0.07244556	0.45651318	0.20600757	0.39451904	0.64624198	-0.0640437	0.74064753	0.81137785
EC_5.1.1.13	-0.8249744	-1.3202189	3.18165912	-1.7974441	0.78769754	3.45792821	-0.2497372	0.74115906	0.81138476
EC_2.1.1.176	0.6907547	0.86534049	2.10039415	0.59717504	1.11599329	2.70144153	-0.1182886	0.74284154	0.8126727
EC_3.4.13.18	0.81021066	-0.142485	-1.0750335	0.21207449	-0.146272	0.44065859	0.11710295	0.74655007	0.81617387
EC_1.8.1.8	3.15785477	0.96162637	0.24886171	2.55325172	0.53698981	0.81380945	0.35972922	0.75313391	0.82212128
EC_3.5.1.5	-0.4071349	-0.8460916	-0.601581	-0.3050316	-0.907015	-0.3683532	-0.0757091	0.75321257	0.82212128
EC_3.2.2.n1	0.26090175	0.22520638	1.49134837	-0.177178	0.83279402	0.98079484	0.09299963	0.75375503	0.82212128
EC_3.2.1.93	1.05589381	0.80569784	1.5915759	1.01550755	1.21744708	2.15463047	-0.1983976	0.75456882	0.82212128
EC_2.3.2.16	2.04113079	-0.7837133	0	1.61436236	-1.0221187	-1.0802805	0.39442957	0.75493116	0.82212128
EC_4.4.1.8	4.05139396	1.63690598	2.01217671	2.71465308	2.71224134	7.02962182	-0.3343235	0.75570065	0.82212128
EC_1.3.1.43	1.93114161	2.30262381	0	2.91885138	-0.1500956	-1.0954314	0.44068839	0.75578968	0.82212128
EC_2.5.1.75	0.36967538	0.08301076	1.3221321	0.27606381	0.55515864	0.89464838	-0.063941	0.75608539	0.82212128
EC_3.2.1.10	-0.0441405	0.38723805	3.08678995	-0.3959636	1.28224189	3.34373887	-0.1530649	0.76220645	0.82821621
EC_3.2.1.20	-0.1916101	-0.1731547	3.09743026	-0.7632528	0.41108767	2.51523825	0.16566563	0.76413795	0.82975358
EC_3.5.1.25	1.12512911	1.07435288	1.11101908	0.97328354	1.19505767	2.28112083	-0.112352	0.76615229	0.83137876
EC_2.7.1.39	2.08580058	1.44324734	2.60589541	1.07215856	2.13576478	3.66756185	0.16017683	0.76995517	0.83494125
EC_1.4.1.21	-1.7853594	0.40946386	1.94878544	0.25686037	-0.3901861	-1.158631	-0.4090635	0.77393841	0.83819768
EC_2.5.1.1	-0.47007	-1.2531104	2.7564242	-3.6139032	1.10049059	6.69021267	0.36281947	0.77400197	0.83819768
EC_3.6.3.19	2.73395145	1.03099852	4.89488188	1.05615904	5.35262205	4.16876353	-0.4781257	0.7760244	0.83922644
EC_3.2.1.31	0.84991747	0.54971535	3.18118191	0.27591681	1.79903986	3.36381812	-0.1515706	0.77606548	0.83922644
EC_5.3.1.25	1.01169819	0.71619765	2.36662086	0.78029535	0.88651061	2.07662351	0.0877254	0.77651961	0.83922644
EC_3.4.24.55	1.72853365	1.02253577	0.41604437	1.09887955	0.75613723	1.97714888	0.21575943	0.77759157	0.83980273
EC_3.5.3.11	1.68613763	-0.4244	-1.1117095	0.99319914	0.06112744	2.04421432	-0.2299473	0.77809867	0.83980273
EC_5.3.1.14	0.34857948	0.02160842	0.21223803	0.21253571	0.12496061	0.93758057	-0.0629879	0.78109818	0.84209674

EC_1.6.99.3	1.10619648	0.5090194	3.61134179	-1.3953438	2.11715152	7.23450556	0.31193404	0.78127281	0.84209674
EC_3.2.1.50	-0.4366682	-1.2361155	-0.3383145	-1.3050032	-0.1286413	1.55532026	-0.164159	0.78283646	0.8432162
EC_4.3.2.3	3.26126927	1.21729341	0.8269317	1.35901478	1.80897097	4.6844138	0.29563076	0.78704544	0.84718162
EC_5.4.2.1	-0.0179118	-1.5316404	3.85372287	-1.6735311	1.41598849	5.25892596	-0.3295238	0.79278969	0.85279319
EC_1.3.1.12	1.56468455	0.14412563	0.76756984	1.20179857	0.41644838	0.71928372	0.08290987	0.79614918	0.85583372
EC_2.4.1.11	0.32355883	-0.7687605	-1.4131426	-0.2819598	-1.1032934	0.55977484	0.16452185	0.79956793	0.85893384
EC_2.1.1.34	0.30962971	-0.3604383	-2.1149099	0.2765347	-0.7586624	-0.3581575	-0.0786159	0.80133232	0.85994318
EC_5.3.2.n1	-0.855925	1.7821388	0	0.34570513	-0.5990444	0	0.19286314	0.80157842	0.85994318
EC_1.9.3.1	1.97298246	-0.1814053	0	1.90691192	0.48114725	0	-0.2208874	0.80620605	0.86433038
EC_6.2.1.3	0.83812098	-0.1623031	-0.5428001	0.58258488	0.0061545	0.46354972	-0.0634255	0.81113975	0.86903965
EC_3.6.1.22	2.17363943	1.21462201	1.1585809	1.66880325	1.7712572	3.07727941	-0.1778359	0.81236416	0.86931563
EC_1.1.1.47	2.56265915	1.57782208	2.35408582	-0.0067428	3.57014022	5.51113311	0.2974707	0.81262545	0.86931563
EC_3.1.1.29	0.21875804	0.01372953	0.29422631	0.14923061	0.18724863	0.27779336	-0.0231944	0.81302122	0.86931563
EC_6.3.3.2	0.13859788	-0.0705723	1.41484422	-0.1653259	0.51996731	0.66837074	0.05206256	0.81379249	0.86956137
EC_1.3.5.4	4.2588859	2.13653426	9.57042776	3.38896156	3.5450961	6.78628786	0.27182658	0.82032587	0.87595968
EC_3.1.13.1	0.4396956	0.06707516	0.57860953	0.30257303	0.19941021	0.47531496	0.03330674	0.82405051	0.87901915
EC_2.3.1.18	0.90857004	0.93629659	2.37947432	1.06367478	1.05655003	3.03271314	-0.2212658	0.82510832	0.87901915
EC_5.1.99.1	-0.4446002	0.50114485	-2.2399524	-0.0672736	-0.8252223	0.79754222	-0.1274457	0.82573599	0.87901915
EC_2.7.1.198	1.84746421	1.94848757	0	2.05806161	-1.0842339	4.71561273	0.28007008	0.82597732	0.87901915
EC_3.1.1.27	-1.2897301	1.8552335	0	1.52040531	-0.8065781	-2.8629316	-0.1994172	0.82612933	0.87901915
EC_6.3.4.18	1.01357956	0.2260253	-0.8632389	0.27937358	0.54248666	0.83886524	0.06546384	0.82647504	0.87901915
EC_2.6.1.51	0.51667653	-2.3508091	-0.607888	0.12575827	-1.6191544	-2.8696717	0.20297722	0.82776074	0.87924292
EC_3.6.3.6	-1.1900784	-3.5032193	3.42896919	-2.6275737	-0.4493127	2.76274333	-0.2378556	0.82778038	0.87924292
EC_1.6.99.5	0.70754618	-0.2669641	-0.5355298	0.12439925	0.27344528	0.95546938	-0.0724043	0.82920515	0.87990349
EC_2.8.4.3	0.0433409	-0.0213	-0.3788785	0.08083616	-0.0753634	-0.2222808	-0.0211141	0.82981705	0.87990349
EC_5.3.1.6	0.69599153	0.86111386	1.75516995	0.43964753	1.25260542	2.37281954	-0.0758083	0.83004594	0.87990349
EC_1.2.1.41	0.34642459	0.48219313	0.60889057	0.29499859	0.58769582	0.84896068	-0.0370442	0.83218801	0.88159232
EC_3.6.1.7	0.61313866	0.25717016	1.98706853	0.20273323	1.04005388	2.1510455	-0.0777157	0.83595541	0.8849996
EC_2.1.1.79	1.59210242	0.68619269	3.24858227	-0.6791914	2.69373527	5.74389897	0.20390369	0.8373401	0.8856969
EC_3.1.3.7	0.58425809	-0.0095934	-1.1291011	0.44322017	-0.1860844	0.29678098	-0.0537255	0.83771705	0.8856969
EC_6.1.1.3	0.04314107	-0.0316393	-0.0787269	0.01273276	-0.064587	0.02518952	0.01270111	0.83847861	0.88591885
EC_3.4.14.4	-0.0363293	-0.0080748	-1.696325	0.15322035	-0.6463529	-0.2374512	-0.069516	0.84036834	0.88733173
EC_2.5.1.15	0.62125354	0.8438548	1.51467544	0.74152612	0.76865804	1.66665343	-0.0623207	0.8425397	0.88903993
EC_2.7.4.2	-0.487958	-0.6069254	2.22758556	-3.0237499	1.98758512	8.00089294	-0.2646295	0.84379867	0.88978376
EC_2.4.1.280	0.35610282	0.21235577	1.65808326	0.41718509	-0.4393686	3.92300371	-0.1319901	0.84929101	0.89498777
EC_3.5.1.18	2.35017619	1.27783174	4.0843017	0.61919069	2.7500983	5.87313855	0.17496477	0.8512467	0.89646046
EC_6.1.1.7	0.14204629	0.0255624	0.48675824	0.11189375	0.169564	0.33816469	-0.0174828	0.85477021	0.89911223
EC_2.4.2.52	3.22839827	0.39981839	5.56451813	2.10806591	2.81077385	4.8694754	-0.1883022	0.85488442	0.89911223
EC_3.5.3.7	-0.9944865	2.33969251	4.04666347	0.1092221	0.19067521	6.38847529	-0.1562551	0.8578307	0.90162049
EC_1.5.1.3	0.48107708	0.08339668	0.71721657	0.20294799	0.305381	0.90688974	0.0401745	0.86016747	0.90348526
EC_2.7.10.2	3.30766923	2.98644541	5.23076386	1.46282179	5.06556482	9.39671982	-0.2565462	0.86172273	0.90452726
EC_1.3.7.8	1.6015093	2.93854156	5.69521561	2.70166703	2.77810556	3.17347255	-0.2118562	0.86413322	0.90625344
EC_2.3.1.54	2.01768572	0.96871287	2.30175975	1.33619464	2.3222123	2.4304846	-0.1222672	0.86488586	0.90625344
EC_2.4.2.29	0.09102593	-0.0276786	0.14462927	0.06413849	0.05482376	0.12345672	-0.0133239	0.8650601	0.90625344
EC_3.2.1.52	0.93553937	0.44456192	2.69468054	0.61281979	1.07997417	1.73445631	0.06394013	0.86576206	0.90639757
EC_4.1.1.32	0.84838198	-0.6690326	-0.8410634	-0.0855599	0.15510192	1.52064035	-0.1004578	0.87096402	0.91124965
EC_5.4.99.24	1.47038522	-0.608635	0.05334946	0.8601033	-0.1069182	1.84346201	-0.1118528	0.87283717	0.91261491
EC_3.4.24.70	2.64025948	0.70317672	0.2603987	2.14488305	0.53579005	1.14962924	0.17589882	0.87608873	0.91487172
EC_4.1.2.14	-0.2957619	-0.5141619	0.23538507	-0.2908867	-0.5382441	-0.0315373	0.02991518	0.8761791	0.91487172
EC_3.2.1.3	0.46315426	1.33481862	0.13539715	1.10299233	0.28356259	1.3057024	-0.1292009	0.87670459	0.91487172
EC_3.6.3.28	0.27756962	-0.7099228	-0.2760336	0.19894145	-1.053691	3.76E-05	0.09786336	0.87972612	0.91742867
EC_1.1.1.57	3.36551604	3.02293982	5.43819085	3.19652489	3.42179824	6.18685517	-0.1649073	0.8855473	0.92290005
EC_2.7.4.22	0.09106618	-0.0389332	-0.1576486	0.03731935	-0.0314317	-0.0411909	0.01007128	0.88861137	0.9245252
EC_4.1.99.17	0.5536115	0.11621942	1.34216943	0.4606379	0.34500451	0.82796489	0.024374	0.88871131	0.9245252
EC_2.7.1.201	0.5342142	1.6894942	4.76174042	1.22937395	1.29359732	2.06090088	0.10192864	0.88883369	0.9245252
EC_2.4.2.10	0.19006619	-0.0019722	0.31696663	0.12158615	0.09717503	0.22225442	0.01079708	0.8897952	0.92492627
EC_1.1.1.17	1.49553856	0.87181043	1.37413586	0.81238063	1.17951299	2.93265834	0.04850655	0.89099274	0.92557202

EC_3.1.4.1	-1.6291392	-1.2881586	4.85843534	-3.7728587	1.03209367	6.31642106	0.15905598	0.89176682	0.92577732
EC_2.7.1.19	3.75202904	-1.9530288	5.83713257	1.51049412	2.3285599	4.80027963	-0.2239327	0.89258577	0.9260289
EC_5.3.1.24	0.48078503	0.62904691	0.93155661	0.45387379	0.51135344	1.02719212	0.04053143	0.89364155	0.92652571
EC_2.7.1.148	0.24814341	0.15404909	0.59412694	0.20113967	0.33953253	0.44625775	-0.0214894	0.89564074	0.92799938
EC_6.3.2.9	0.19591449	-0.0292724	0.36897559	0.08456082	0.18324397	0.37745534	-0.0180209	0.89639173	0.92817867
EC_1.3.5.2	1.9990614	0.31505902	-1.8123041	0.9738237	0.94269695	0.3191965	0.05779838	0.89744977	0.92867548
EC_3.1.26.8	0.3447411	0.26631749	3.49307603	-1.1570593	1.81901138	4.73656932	0.11002248	0.90080566	0.93125032
EC_6.3.4.6	-0.331466	0.50788561	0.20720451	0.24661446	-0.2635347	0.28464439	-0.0524772	0.90236186	0.93125032
EC_1.6.1.2	5.48685807	3.61100141	5.41137199	3.41647703	6.56884434	4.66573578	0.19897706	0.90239409	0.93125032
EC_4.1.99.12	-0.0205954	-0.2371425	0.29929771	0.03924857	-0.1758466	-0.0694335	-0.0125336	0.90245328	0.93125032
EC_2.4.1.320	0.25087096	0.91806499	-0.6706726	0.24045756	0.39232671	0.35851387	0.06847994	0.90298206	0.93125032
EC_4.2.3.1	1.06191129	1.06906599	0.63337409	0.89470389	0.97790688	1.8624376	-0.0371617	0.90341718	0.93125032
EC_3.5.3.18	1.87153014	0.72522578	8.46483535	1.14517993	3.37266697	5.3918395	-0.1596682	0.90637733	0.93268152
EC_2.5.1.120	-0.577685	-2.9043658	-7.0430241	-0.5816439	-4.4473523	-4.3724684	0.15283411	0.90648551	0.93268152
EC_3.1.13.5	3.69034747	0.61222779	4.36881434	2.44198033	1.00759987	6.61617537	0.16337082	0.90752554	0.93268152
EC_3.5.1.24	-0.444165	0.05360389	3.90980101	-0.7769294	0.69100333	4.31164623	-0.1069098	0.90767591	0.93268152
EC_2.8.1.8	0.4395081	-0.0681835	-0.4562642	0.29052073	-0.1967435	0.21245399	0.02939666	0.90770935	0.93268152
EC_2.8.3.8	2.1361673	0.50504552	-1.5551228	1.06835008	0.99689194	2.19380775	-0.0867363	0.91015698	0.93459854
EC_1.2.4.4	2.00235489	1.05995124	0.08273057	1.50635631	1.27092089	2.2201808	-0.0896341	0.91189653	0.935451
EC_3.1.6.1	1.12975586	0.60449052	0.18950379	0.86995883	0.63706497	1.42850635	-0.0384868	0.91251918	0.935451
EC_3.1.4.53	3.69421101	4.56569218	2.8516271	3.62680551	3.77920146	6.41650381	-0.1435429	0.91273457	0.935451
EC_3.2.1.18	2.19770058	1.01667791	4.79888505	2.21269413	1.63112247	3.63578275	-0.1106962	0.91761513	0.93985325
EC_3.6.3.17	1.69766307	1.50275931	2.29838476	1.44768569	1.66151453	3.24944841	-0.0521761	0.92016696	0.94186624
EC_1.4.1.11	-0.7067371	3.80475787	2.23769719	0.19191568	0.8971241	5.58559935	0.1205951	0.92244234	0.9430807
EC_4.2.1.42	2.03742936	0.70039543	2.20116943	1.24202987	1.62436637	2.4402913	0.05915167	0.92252788	0.9430807
EC_3.4.24.75	1.13653666	0.87382764	0.7989313	1.50004147	-0.4853487	1.94404916	0.09475567	0.92420763	0.94419686
EC_2.2.1.1	1.81398695	0.6957082	1.36640779	1.28885028	1.08426256	2.07628802	0.03785055	0.92930727	0.94866981
EC_3.6.1.54	2.14604122	0.25710396	1.64507252	2.13381006	-0.4548553	2.30329614	0.10832599	0.9297673	0.94866981
EC_4.1.2.4	-0.1909679	-0.208068	-0.1057401	-0.0671289	-0.3262326	-0.2751982	-0.0071636	0.93466631	0.95264509
EC_3.8.1.8	-2.153566	0.06047955	0	0.47445243	-2.3785284	-3.7692358	-0.0870205	0.93515106	0.95264509
EC_2.3.1.57	0.89618037	0.40695166	1.87349927	0.19685463	1.147176	2.49692993	0.0321412	0.93544291	0.95264509
EC_3.5.4.25	0.04694341	-0.2484134	0.29976363	0.06016587	-0.1694552	-0.08842	0.00795637	0.93824843	0.95489669
EC_2.7.4.8	0.43150589	0.22434222	0.7519661	0.23904018	0.41536732	0.89143969	0.01486657	0.94038801	0.95646811
EC_4.2.1.70	2.11606887	0.89403083	1.25732823	1.01252996	1.50430074	3.67495431	0.05452195	0.94317091	0.95869144
EC_2.7.1.189	1.40507291	2.19821532	3.6090126	1.7075443	1.976838	3.40564574	-0.0627301	0.94380932	0.95873356
EC_2.7.7.77	3.68110115	1.40523398	6.24545672	1.93859448	3.56418115	6.83810374	0.0742011	0.94943679	0.96384038
EC_2.7.1.76	-0.8814459	-2.4394802	3.29340981	-2.4957858	0.20101221	3.1406818	-0.0503509	0.95972968	0.97367395
EC_3.1.1.72	2.80356375	2.31953537	4.77708579	1.85411092	3.08298393	6.9554271	-0.0530237	0.96151357	0.97486792
EC_2.4.1.230	0.39816948	-0.4046264	-0.8150544	0.01925979	-0.4794658	0.6407408	0.02776753	0.96255793	0.97531106
EC_1.4.1.3	0.72084354	1.0497009	-0.2112089	0.70357538	0.49467627	1.13796186	0.03095799	0.96328915	0.97543656
EC_2.4.1.281	-1.2679848	-0.6630404	-1.3957596	-0.7251026	-1.2187269	-2.0164033	-0.0128217	0.96421592	0.97575978
EC_3.1.1.83	2.9932312	2.5522648	3.67871442	1.81869568	3.58178089	5.4185634	0.05146527	0.96621684	0.97716892
EC_2.4.1.20	-0.119352	-0.0550925	-1.3649011	-0.1307095	0.0090041	-1.2061844	-0.0231101	0.96688671	0.977231
EC_3.5.99.6	0.77171683	0.34970081	0.86644158	0.5922083	0.58583819	0.98330936	-0.0076355	0.97003057	0.97908824
EC_1.1.1.169	0.82941742	1.15232875	1.56574085	0.68036649	1.24146305	1.87363542	0.01297552	0.97140974	0.97908824
EC_1.21.98.1	0.84401968	-0.2608081	-4.6221146	0.30833309	-1.2801749	0.01888583	0.03327136	0.97148671	0.97908824
EC_5.1.3.8	0.07041123	0.75794138	5.69541017	0.82839259	0.36730313	3.63625144	-0.0425971	0.97150292	0.97908824
EC_2.1.3.1	-0.13272	-0.0829572	-1.9773812	-0.4009508	-0.5948155	0.65882219	-0.0158548	0.97177251	0.97908824
EC_3.5.99.10	2.16849954	1.14530771	4.41033913	1.58531227	1.49894012	5.26397334	0.04073543	0.97432302	0.98104249
EC_1.1.1.103	1.42865291	1.14108286	2.23590642	1.21243723	1.27577356	2.56257977	0.01521323	0.97576912	0.98188296
EC_4.3.1.15	2.21680627	1.9290692	4.81636487	1.91582977	2.41531334	4.79714816	-0.0245152	0.97812605	0.98363834

EC_2.4.1.57	0.11236208	5.53682123	0.69117526	0.86007729	4.19482961	2.32828564	-0.0417143	0.97898327	0.98388431
EC_2.3.1.128	0.45893351	-1.2594479	2.223499	-2.6689352	1.86504907	6.64418538	0.02568089	0.98360034	0.98790628
EC_6.4.1.3	0.28999847	-0.0326268	-1.1845423	0.18243944	-0.243669	-0.2145375	0.00441183	0.98441766	0.98810923
EC_2.3.1.234	0.1365485	0.0109212	0.03629571	0.10961054	0.01793139	0.10690277	0.00057014	0.99416104	0.99726585
EC_1.3.98.3	1.04861163	1.0618216	1.49349932	0.90648994	0.94569534	2.32827329	0.00174484	0.99665047	0.99913899
EC_3.4.15.5	0.85405165	0.11742181	-0.6243599	0.4802644	0.10729712	0.89629611	-0.0006755	0.99843382	0.99955496
EC_4.2.1.51	0.82545717	0.29439717	0.42398025	0.73728557	0.21100727	0.91247845	0.00041908	0.99890527	0.99955496
EC_3.2.1.21	1.14386513	1.01696693	0.33556529	0.86841994	1.15371174	1.15235934	0.00058946	0.99893257	0.99955496
EC_3.1.3.71	1.53713435	-1.129492	-0.0865954	0.40091648	-1.0697958	4.01826199	-0.0001546	0.99990485	0.99990485
