# Supplementary material for: Impaired flux of bile acids from the liver to the gut reveals microbiome-immune interactions associated with liver damage
Source: NPJ Biofilms Microbiomes. 2023 Jun 7;9:35. doi: 10.1038/s41522-023-00398-0 (PMC10247725; doi:10.1038/s41522-023-00398-0)
Supplement: Supplementary file 2 — Reporting Summary [file 41522_2023_398_MOESM2_ESM.pdf]

nature portfolio

Corresponding author(s): Adrian Press, Gianni Panagiotou

Last updated by author(s): Mar 24, 2023

Reporting Summary

Nature Portfolio wishes to improve the reproducibility of the work that we publish. This form provides structure for consistency and transparency in reporting. For further information on Nature Portfolio policies, see our Editorial Policies and the Editorial Policy Checklist.

Statistics

For all statistical analyses, confirm that the following items are present in the figure legend, table legend, main text, or Methods section.

n/a	Confirmed	
☐	☒	The exact sample size (*n*) for each experimental group/condition, given as a discrete number and unit of measurement
☐	☒	A statement on whether measurements were taken from distinct samples or whether the same sample was measured repeatedly
☐	☒	The statistical test(s) used AND whether they are one- or two-sided<br>*Only common tests should be described solely by name; describe more complex techniques in the Methods section.*
☐	☒	A description of all covariates tested
☒	☐	A description of any assumptions or corrections, such as tests of normality and adjustment for multiple comparisons
☒	☐	A full description of the statistical parameters including central tendency (e.g. means) or other basic estimates (e.g. regression coefficient) AND variation (e.g. standard deviation) or associated estimates of uncertainty (e.g. confidence intervals)
☐	☒	For null hypothesis testing, the test statistic (e.g. *F*, *t*, *r*) with confidence intervals, effect sizes, degrees of freedom and *P* value noted<br>*Give P values as exact values whenever suitable.*
☒	☐	For Bayesian analysis, information on the choice of priors and Markov chain Monte Carlo settings
☒	☐	For hierarchical and complex designs, identification of the appropriate level for tests and full reporting of outcomes
☐	☒	Estimates of effect sizes (e.g. Cohen's *d*, Pearson's *r*), indicating how they were calculated

Our web collection on statistics for biologists contains articles on many of the points above.

Software and code

Policy information about availability of computer code

Data collection	No software was used.
Data analysis	We used the following softwares for our analyses. For metagenomics profiling: mOTUs v2.6, HUMAnN3, .For statistical analysis and data visualization: R v3.6.3 (R packages: vegan, ggplot2, ComplexHeatmap)

For manuscripts utilizing custom algorithms or software that are central to the research but not yet described in published literature, software must be made available to editors and reviewers. We strongly encourage code deposition in a community repository (e.g. GitHub). See the Nature Portfolio guidelines for submitting code & software for further information.

Data

Policy information about availability of data

All manuscripts must include a data availability statement. This statement should provide the following information, where applicable:

- Accession codes, unique identifiers, or web links for publicly available datasets
- A description of any restrictions on data availability
- For clinical datasets or third party data, please ensure that the statement adheres to our policy

Raw metagenomic sequencing data for all samples of this study have been deposited in European Nucleotide Archive under accession ID PRJEB57214.

Human research participants

Policy information about studies involving human research participants and Sex and Gender in Research.

Reporting on sex and gender	N/A
Population characteristics	N/A
Recruitment	N/A
Ethics oversight	N/A

Note that full information on the approval of the study protocol must also be provided in the manuscript.

Field-specific reporting

Please select the one below that is the best fit for your research. If you are not sure, read the appropriate sections before making your selection.

☒ Life sciences ☐ Behavioural & social sciences ☐ Ecological, evolutionary & environmental sciences

For a reference copy of the document with all sections, see nature.com/documents/nr-reporting-summary-flat.pdf

Life sciences study design

All studies must disclose on these points even when the disclosure is negative.

Sample size	Sample sizes were estimated based on previous studies used for BDL mouse model (Fouts et al., Journal of Hepatology, 2012)
Data exclusions	All data collected were used in the analysis.
Replication	Number of mice used in the statistics as described in the Methods and manuscript.
Randomization	Mice were randomly selected to receive the BDL or Sham surgery.
Blinding	The group information is necessary for our case-control study.

Reporting for specific materials, systems and methods

We require information from authors about some types of materials, experimental systems and methods used in many studies. Here, indicate whether each material, system or method listed is relevant to your study. If you are not sure if a list item applies to your research, read the appropriate section before selecting a response.

Materials & experimental systems

n/a	Involved in the study	
☒	☐	Antibodies
☒	☐	Eukaryotic cell lines
☒	☐	Palaeontology and archaeology
☐	☒	Animals and other organisms
☒	☐	Clinical data
☒	☐	Dual use research of concern

Methods

n/a	Involved in the study	
☒	☐	ChIP-seq
☒	☐	Flow cytometry
☒	☐	MRI-based neuroimaging

Animals and other research organisms

Policy information about studies involving animals; ARRIVE guidelines recommended for reporting animal research, and Sex and Gender in Research

Laboratory animals	For all experiments, male and female FVB/NRj mice (Janvier Laboratory, France) were housed under specific pathogen-free conditions and bred in the Jena University Hospital (Germany) animal facility. All animals were given LASQ diet (Rod 16, Auto) from Altromin company, which was composed of cereals, vegetable by-products, minerals, oils, fats and yeast, and provided mice crude nutrients with 16.9% crude protein, 4.3% crude fat, 4.3% crude fibre and 7.0% crude ash.

Wild animals	Study did not involve wild animals.
Reporting on sex	Sex-based analysis was not performed.
Field-collected samples	No field-collected samples.
Ethics oversight	All experimental animal procedures in this study were evaluated through the Independent Ethical Advisory Committee and approved by the Local Government Authority of Thuringia, Thüringer Landesverwaltungsamt, per European and German laws and regulations under the license UKJ-19-010.

Note that full information on the approval of the study protocol must also be provided in the manuscript.
